# Partially Saturated Bicyclic Heteroaromatics as an sp^3^‐Enriched Fragment Collection

**DOI:** 10.1002/anie.201606496

**Published:** 2016-09-06

**Authors:** David G. Twigg, Noriyasu Kondo, Sophie L. Mitchell, Warren R. J. D. Galloway, Hannah F. Sore, Andrew Madin, David R. Spring

**Affiliations:** ^1^ Department of ChemistryUniversity of CambridgeLensfield RdCambridgeCB2 1EWUK; ^2^ Shionogi & Co. Ltd.1-1, Futaba-cho 3-chome, ToyonakaOsaka561-0825Japan; ^3^ AstraZeneca UK Ltd.310 Cambridge Science Park, Milton RdCambridgeCB4 0FZUK

**Keywords:** drug design, drug discovery, fused-ring systems, nitrogen heterocycles, synthetic methods

## Abstract

Fragment‐based lead generation has proven to be an effective means of identifying high‐quality lead compounds for drug discovery programs. However, the fragment screening sets often used are principally comprised of sp^2^‐rich aromatic compounds, which limits the structural (and hence biological) diversity of the library. Herein, we describe strategies for the synthesis of a series of partially saturated bicyclic heteroaromatic scaffolds with enhanced sp^3^ character. Subsequent derivatization led to a fragment collection featuring regio‐ and stereo‐controlled introduction of substituents on the saturated ring system, often with formation of new stereocenters.

Fragment‐based drug discovery (FBDD) is a well‐established method for generating high‐quality hits and leads.^1^ The approval of the B‐Raf kinase inhibitor vemurafenib (Zelboraf) in 2011^2^ and the Bcl‐2 inhibitor venetoclax (Venclexta) in 2016,^3^ coupled with the ongoing evaluation of over 20 candidates in clinical trials,^4^ validates this approach as a complementary strategy to other hit‐discovery techniques such as high‐throughput screening.^5^ While the growing prevalence of fragment‐based approaches is encouraging, evaluation of many existing fragment libraries shows a predominance of (hetero)aromatic, “flat” compounds, with a deficiency of chiral, sp^3^‐rich examples.^6^, ^7^

Studies by Ritchie et al.^8^ and Lovering et al.^9^ demonstrate improvements in project progression by, for example, increasing the fraction of sp^3^ centers within molecules or restricting the number of aromatic rings. Furthermore, computational analysis demonstrates that greater 3D conformational character is observed in compounds that have been clinically evaluated in humans, compared to those found in commercial libraries.^10^ This indicates the importance of sp^3^‐richness in both the design of screening collections and the subsequent development of hits to leads.

Examples of previous studies aiming to synthesize collections of sp^3^‐enriched fragments have been limited. Diversity‐oriented synthesis^7^, ^11^ and natural product derivatives^12^ have been used to generate 3D fragment collections, but there remains an unmet need to access new scaffold types. Recently there have been calls^13^ for new approaches and methodologies for designing fragments with multiple synthetically accessible growth vectors in three dimensions, to allow rapid and efficient elaboration of hits to leads after initial screening, with some early success.^14^

With these points in mind, the study described herein was aimed at developing efficient synthetic routes to a series of partially saturated bicyclic heteroaromatic (PSBH) fragments with enhanced sp^3^ content relative to existing fragment libraries. Compounds featuring PSBHs have been shown to display bioactivity against a range of targets (Figure 1),^15^ and so a series of related fragments might be expected to serve effectively as a screening collection for FBDD applications.

**Figure 1:**
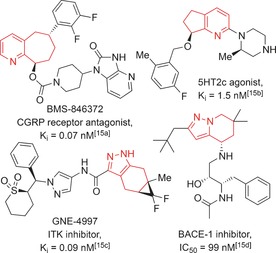
Selected examples of bioactive compounds containing functionalized partially saturated bicyclic heteroaromatics (highlighted in red).

The targets of this study featured a variable aromatic heterocycle fused to a partially saturated carbocycle. The heterocycle could bear either a polar (e.g., amino) group, which should greatly enhance aqueous solubility, a property necessary for fragment screening at higher concentrations,^7^, ^14^ or alternatively a hydrophobic (e.g., chloro) group, which can forge key interactions with protein targets.^16^ The synthetic route (Scheme 1) employed a modular and divergent approach, using simple cross‐coupling and alkylation reactions to install a pair of terminal olefins that could be reliably cyclized through ring‐closing metathesis (RCM).^17^ This allowed excellent control of the carbocycle ring size and the position and orientation of the resultant endocyclic olefin growth vector, which could undergo subsequent functionalization to produce a range of fragments suitable for screening and/or further elaboration.

**Scheme 1:**
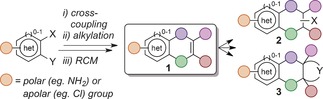
General synthetic strategy toward PSBH scaffolds **1** and subsequent incorporation of new functionalities (**2**) or rings (**3**).

We selected pyrazole and pyridine as representative aromatic heterocycles. Whilst previous studies have shown the synthesis of related structures,^18^ they have incorporated less control over the position of the olefin and do not feature the amino group found in many of our compounds. Furthermore, there are only very few examples where the olefin is used as a branch point and further functionalized beyond simple reduction.15a, 19

Starting from readily available 3‐nitro‐1*H*‐pyrazole (**4**), 2‐(trimethylsilyl)ethoxymethyl (SEM) protection, selective iodination, and subsequent Suzuki coupling with potassium vinyltrifluoroborate gave vinyl derivative **7 a** (Table 1). Deprotection followed by N‐alkylation with an alkyl bromide of varying C‐chain length provided metathesis precursors **9 a**–**c**, which upon treatment with either Grubbs’ or Hoveyda‐Grubbs’ 2^nd^ generation catalysts yielded the desired scaffolds **10 a**–**c**. Inclusion of further heteroatoms in the formation of medium‐sized partially saturated rings was achieved through treatment of vinyl intermediate **8 a** with either tosylate **11** (leading, after RCM, to O‐containing fragment **10 d**), or 3‐Boc‐1,2,3‐oxathiazolidine 2,2‐dioxide **12**, which gave access to the N‐containing scaffold **10 e** after allylation and metathesis. Use of a different Suzuki coupling partner gave methyl‐substituted product **7 f**, which could be elaborated to PSBH fragment **10 f**. Alternatively, direct allylation at the C‐5 position of SEM‐protected intermediate **5** could be achieved upon treatment with lithium diisopropylamide (LDA) and allyl bromide. This led, in an analogous way, to scaffolds **10 g**–**h** with non‐conjugated olefins.

**Table 1:** Synthesis of pyrazole‐based PSBH scaffolds. 
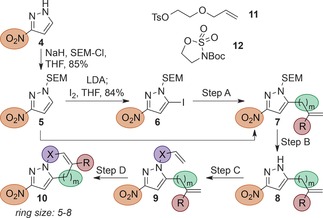

				Step			
	m	R	X	A (**7**)	B (**8**)^[c]^	C (**9**)^[d]^	D (**10**)^[g]^
**a**	0	H	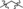	85 %^[a]^	80 %	55 %	45 %^[h]^
**b**	0	H	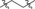	–	–	86 %	84 %
**c**	0	H	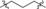	–	–	55 %	89 %
**d**	0	H	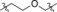	–	–	59 %^[e]^	65 %
**e**	0	H	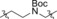	–	–	29 %^[f]^	83 %
**f**	0	Me	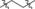	76 %^[a]^	69 %	44 %	90 %
**g**	1	H	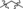	61 %^[b]^	82 %	70 %	93 %
**h**	1	H	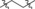	–	–	36 %	59 %^[h]^

Reaction conditions: [a] R′‐BF_3_K (1.5 equiv), Pd(dppf)Cl_2_⋅CH_2_Cl_2_ (5 mol %), K_2_CO_3_ (3.0 equiv), THF/H_2_O, 70 °C. [b] **5** (1.0 equiv), LDA (1.2 equiv), CuBr (20 mol %), allyl bromide (1.2 equiv), THF, −78 °C to RT. [c] TFA, CH_2_Cl_2_, RT. [d] NaH (1.5 equiv), alkyl bromide (1.5 equiv), THF or DMF, 70 °C. [e] NaH (1.5 equiv), **11** (1.5 equiv), THF, 70 °C. [f] **12** (2.0 equiv), K_2_CO_3_ (3.0 equiv), DMF, RT, 43 %; *then* NaH (1.5 equiv), allyl iodide (1.5 equiv), DMF, RT, 68 %. [g] Grubbs II (10 mol %), CH_2_Cl_2_, 40 °C. [h] Hoveyda‐Grubbs II (10 mol %), toluene, 110 °C or CH_2_Cl_2_, RT. THF=tetrahydrofuran, TFA=trifluoroacetic acid, DMF=*N*,*N*‐dimethylformamide.

A similar approach was used to generate PSBH scaffolds from pyridine **13** (Table 2). Attempts to mask the 2‐amino group as a nitro group proved ineffective since, despite successful cross‐coupling reactions, the 2‐nitropyridines were unstable to strong base and did not undergo the desired alkylations at the 4‐methyl position. Mono‐Boc protection was also unsuitable due to poor yields in the cross‐coupling step, possibly due to catalyst chelation. The 2‐amino group could be rendered synthetically tractable, however, either through bis‐Boc protection or through substitution with a 2‐chloro group, which itself can serve as a synthetic handle.^20^

**Table 2:** Synthesis of pyridine‐based PSBH scaffolds. 
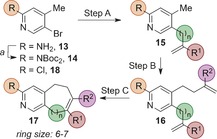

					Step		
	R	n	R^1^	R^2^	A (**15**)^[b]^	B (**16**)^[e]^	C (**17**)^[f]^
**a**	Boc_2_N	0	H	H	85 %	60 %	95 %
**b**	Boc_2_N	0	H	Me	–	69 %	94 %
**c**	Boc_2_N	0	H	CF_3_	–	77 %	54 %
**d**	Boc_2_N	0	Me	H	87 %	60 %	94 %
**e**	Boc_2_N	0	Ph	H	92 %	68 %	83 %
**f**	Boc_2_N	1	H	H	78 %^[c]^	41 %	65 %
**g**	Cl	1	H	H	73 %^[d]^	79 %	91 %

Reaction conditions: [a] Boc_2_O (2.5 equiv), DMAP (0.1 equiv), THF, 70 °C. [b] R′‐BF_3_K or R′‐B(MIDA) (1.5 equiv), Pd(dppf)Cl_2_⋅CH_2_Cl_2_ (10 mol %), K_2_CO_3_ (3.0 equiv), THF/H_2_O, 70 °C. [c] Allyltributyltin (1.1 equiv), Pd(PPh_3_)_4_ (10 mol %), KF (2 equiv), toluene, 110 °C. [d] *i*‐PrMgCl.LiCl (1.5 equiv), allyl bromide (1.2 equiv), THF, −15 °C to RT. [e] LDA (1.2 equiv), alkyl bromide (1.5 equiv), THF, −78 °C to RT. [f] Grubbs II (5 mol %), CH_2_Cl_2_, 40 °C. DMAP=4‐dimethylaminopyridine.

Bis‐Boc substrate **14** (prepared in one step from **13**) was functionalized at the 5‐position using either Suzuki coupling (for vinyl substituents) or Stille coupling (for allyl substituents) to produce intermediates **15 a**,**d**–**f**. Treatment with LDA and trapping of the resultant anion with a variable alkyl bromide electrophile gave a range of metathesis substrates (**16 a**–**f**), which under standard ring‐closing metathesis conditions yielded PSBH scaffolds **17 a**–**f**. The 2‐chloro substrate **18** could be allylated in the 5‐position by using an excess of *i*‐PrMgCl⋅LiCl and trapping the resultant organometallic intermediate with allyl bromide. Allylation at the 4‐methyl position and RCM gave scaffold **17 g** in superior yields.

Following PSBH synthesis, a series of simple one‐, two‐, or three‐step functionalizations were performed on selected pyrazole and pyridine scaffolds to demonstrate the synthetic utility of the olefin π‐bond as a growth vector and to generate a variety of new stereocenters (Scheme 2).

**Scheme 2:**
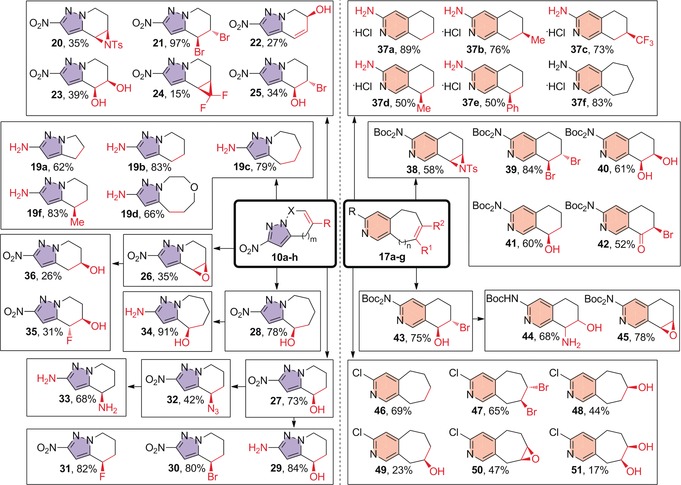
Functionalization of PSBH scaffolds. For reaction conditions, see the Supporting Information.

Catalytic hydrogenation of nitropyrazoles **10 a**–**d**,**f** served to reduce both the olefin π‐bond and the nitro group in moderate to good yields, revealing the latent amino functionality and, in the case of **19 f**, creating a new stereocenter. Other one‐step reactions include aziridination, dibromination, allylic oxidation dihydroxylation, difluorocyclopropanation, hydroxybromination, epoxidation, and hydroboration (**20**–**28**) to introduce functionalities at the 4‐, 5‐ and 6‐positions of the fused pyrazole systems. Demonstrating that these initial products can serve as intermediates to other fragments, the products of hydroboration can react further to incorporate Br, F, and N substituents (**30**–**33**), whilst epoxide **26** can be opened by nucleophiles such as fluoride and hydride to form fluorohydrin **35** and alcohol **36**. Whilst the yields of some reactions were modest, sufficient material was obtained for full characterization and future screening campaigns.

The pyridine‐based scaffolds **17 a**–**g** can undergo a similar range of transformations. Catalytic hydrogenation and subsequent acid‐mediated deprotection of bis‐Boc compounds **17 a**–**f** generated novel fragments, many of which include new stereocenters (**37 a**–**f**). Aziridination, dibromination, dihydroxylation, hydroboration, α‐bromoketone formation, and hydroxybromination were also carried out (**38**–**43**). Further reactions included amino‐alcohol (**44**) and epoxide (**45**) formation, both from bromohydrin **43**. 3‐Chloro fragments **46**–**51** could also be readily accessed using similar conditions.

Calculation of a range of physicochemical properties was carried out on all of the PSBH products. Almost all fragments were shown to conform to the so‐called “Rule of Three”, a set of criteria commonly associated with greater hit rates in fragment screening collections.^21^ Of particular note are the low mean values for molecular weight (190), Slog*P* (1.45), and “fraction aromatic” (0.43) and the high mean number of chiral centres (0.88), especially when compared to existing commercial libraries (Table 3).

**Table 3:** Mean physicochemical properties of fragment collections.

Property^[a]^	Ideal Range^[b]^	This work	Chembridge	Maybridge
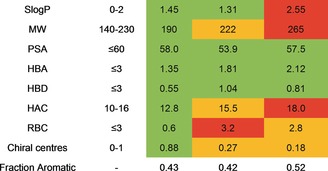				

[a] MW=molecular weight, PSA=polar surface area, HBA=number of hydrogen‐bond acceptors, HBD=number of hydrogen‐bond donors, HAC=heavy atom count, RBC=rotatable bond count. [b] Based on the guidelines used by Astex Pharmaceuticals.^14^, ^21^ Green: within ideal range, orange: at extreme of ideal range, red: outside ideal range. See the Supporting Information for further details.

In conclusion, we have developed simple, scalable routes to a series of partially saturated pyrazole‐ and pyridine‐based scaffolds that can readily undergo a range of synthetic transformations to generate a collection of sp^3^‐rich fragments, which are suitable for use either as screening members in a library or as intermediates to “higher‐content fragments”. The compounds adhere to recognized guidelines for fragment physicochemical properties, whilst displaying enhanced sp^3^ character and greater chirality, and providing a range of three‐dimensional growth vectors for synthetic development. It is envisioned that the strategy could be applied to a vast range of analogous scaffolds with varied heterocycles and substituents and that several of the functionalization reactions detailed in Scheme 2 could be rendered asymmetric based on related precedents.^19^, ^22^

*Dedicated to Professor Stuart L. Schreiber on the occasion of his 60th birthday*

## Supporting information

As a service to our authors and readers, this journal provides supporting information supplied by the authors. Such materials are peer reviewed and may be re‐organized for online delivery, but are not copy‐edited or typeset. Technical support issues arising from supporting information (other than missing files) should be addressed to the authors.

Supplementary

## References

[1a] C. W. Murray , D. C. Rees , Nat. Chem. 2009, 1, 187–192; 21378847 10.1038/nchem.217

[1b] R. E. Hubbard , J. B. Murray in Methods in Enzymology, Vol. 493 (Ed.: L. C. Kuo), Academic Press, New York, 2011, pp. 509–531; 10.1016/B978-0-12-381274-2.00020-0 21371604

[1c] D. Erlanson in Fragment-Based Drug Discovery and X-Ray Crystallography (Eds.: T. G. Davies, M. Hyvönen), Springer, Berlin, 2012, pp. 1–32;

[1d] E. R. Zartler , ACS Med. Chem. Lett. 2014, 5, 952–953; 25221648 10.1021/ml5003212 PMC4160759

[1e] M. Congreve , G. Chessari , D. Tisi , A. J. Woodhead , J. Med. Chem. 2008, 51, 3661–3680; 18457385 10.1021/jm8000373

[1f] G. M. Keserű, D. A. Erlanson, G. G. Ferenczy, M. M. Hann, C. W. Murray, S. D. Pickett, *J. Med. Chem* **2016**, DOI: 10.1021/acs.jmedchem.6b00197. 10.1021/acs.jmedchem.6b00197 27124799

[2a] G. Bollag , J. Tsai , J. Zhang , C. Zhang , P. Ibrahim , K. Nolop , P. Hirth , Nat. Rev. Drug Discovery 2012, 11, 873–886. 23060265 10.1038/nrd3847

[3] A. J. Souers , J. D. Leverson , E. R. Boghaert , S. L. Ackler , N. D. Catron , J. Chen , B. D. Dayton , H. Ding , S. H. Enschede , W. J. Fairbrother , D. C. Huang , S. G. Hymowitz , S. Jin , S. L. Khaw , P. J. Kovar , L. T. Lam , J. Lee , H. L. Maecker , K. C. Marsh , K. D. Mason , M. J. Mitten , P. M. Nimmer , A. Oleksijew , C. H. Park , C. M. Park , D. C. Phillips , A. W. Roberts , D. Sampath , J. F. Seymour , M. L. Smith , G. M. Sullivan , S. K. Tahir , C. Tse , M. D. Wendt , Y. Xiao , J. C. Xue , H. Zhang , R. A. Humerickhouse , S. H. Rosenberg , S. W. Elmore , Nat. Med. 2013, 19, 202–208. 23291630 10.1038/nm.3048

[4] M. Baker , Nat. Rev. Drug Discovery 2013, 12, 5–7. 23274457 10.1038/nrd3926

[5] S. Barelier , I. Krimm , Curr. Opin. Chem. Biol. 2011, 15, 469–474. 21411360 10.1016/j.cbpa.2011.02.020

[6] P. J. Hajduk , W. R. J. D. Galloway , D. R. Spring , Nature 2011, 470, 42–43. 21293363 10.1038/470042a

[7] A. W. Hung , A. Ramek , Y. Wang , T. Kaya , J. A. Wilson , P. A. Clemons , D. W. Young , Proc. Natl. Acad. Sci. USA 2011, 108, 6799–6804. 21482811 10.1073/pnas.1015271108 PMC3084099

[8] T. J. Ritchie , S. J. F. MacDonald , Drug Discovery Today 2009, 14, 1011–1020. 19729075 10.1016/j.drudis.2009.07.014

[9] F. Lovering , J. Bikker , C. Humblet , J. Med. Chem. 2009, 52, 6752–6756. 19827778 10.1021/jm901241e

[10] A. D. Morley , A. Pugliese , K. Birchall , J. Bower , P. Brennan , N. Brown , T. Chapman , M. Drysdale , I. H. Gilbert , S. Hoelder , A. Jordan , S. V. Ley , A. Merritt , D. Miller , M. E. Swarbrick , P. G. Wyatt , Drug Discovery Today 2013, 18, 1221–1227. 23906694 10.1016/j.drudis.2013.07.011

[11] V. S. B. Damerla , C. Tulluri , R. Gundla , L. Naviri , U. Adepally , P. S. Iyer , Y. L. N. Murthy , N. Prabhakar , S. Sen , Chem. Asian J. 2012, 7, 2351–2360. 22887684 10.1002/asia.201200385

[12] B. Over , S. Wetzel , C. Grutter , Y. Nakai , S. Renner , D. Rauh , H. Waldmann , Nat. Chem. 2013, 5, 21–28. 23247173 10.1038/nchem.1506

[13] C. W. Murray , D. C. Rees , Angew. Chem. Int. Ed. 2016, 55, 488–492; 10.1002/anie.201506783 26526786

[14] N. Palmer , T. M. Peakman , D. Norton , D. C. Rees , Org. Biomol. Chem. 2016, 14, 1599–1610. 26741115 10.1039/c5ob02461g

[15a] G. Luo , L. Chen , C. M. Conway , R. Denton , D. Keavy , M. Gulianello , Y. Huang , W. Kostich , K. A. Lentz , S. E. Mercer , R. Schartman , L. Signor , M. Browning , J. E. Macor , G. M. Dubowchik , ACS Med. Chem. Lett. 2012, 3, 337–341; 24900474 10.1021/ml300021s PMC4025799

[15b] K. K.-C. Liu , P. Cornelius , T. A. Patterson , Y. Zeng , S. Santucci , E. Tomlinson , C. Gibbons , T. S. Maurer , R. Marala , J. Brown , J. X. Kong , E. Lee , W. Werner , Z. Wenzel , C. Vage , Bioorg. Med. Chem. Lett. 2010, 20, 266–271; 19914063 10.1016/j.bmcl.2009.10.112

[15c] J. D. Burch , K. Barrett , Y. Chen , J. DeVoss , C. Eigenbrot , R. Goldsmith , M. H. A. Ismaili , K. Lau , Z. Lin , D. F. Ortwine , A. A. Zarrin , P. A. McEwan , J. J. Barker , C. Ellebrandt , D. Kordt , D. B. Stein , X. Wang , Y. Chen , B. Hu , X. Xu , P.-W. Yuen , Y. Zhang , Z. Pei , J. Med. Chem. 2015, 58, 3806–3816; 25844760 10.1021/jm501998m

[15d] C. Sund , O. Belda , N. Borkakoti , J. Lindberg , D. Derbyshire , L. Vrang , E. Hamelink , C. Åhgren , E. Woestenenk , K. Wikström , A. Eneroth , E. Lindström , G. Kalayanov , Bioorg. Med. Chem. Lett. 2012, 22, 6721–6727. 23010268 10.1016/j.bmcl.2012.08.097

[16] Y. S. Tan , D. R. Spring , C. Abell , C. Verma , J. Chem. Inf. Model. 2014, 54, 1821–1827. 24910248 10.1021/ci500215x

[17] RCM has been used in many diversity-generating libraries and applications. Selected examples:

[17a] J. W. Herndon , Coord. Chem. Rev. 2015, 286, 30–150;

[17b] S. Collins , S. Bartlett , F. Nie , H. F. Sore , D. R. Spring , Synthesis 2016, 1457–1473;

[17c] A. Grossmann , S. Bartlett , M. Janecek , J. T. Hodgkinson , D. R. Spring , Angew. Chem. Int. Ed. 2014, 53, 13093–13097; 10.1002/anie.201406865 25257387

[17d] D. Morton , S. Leach , C. Cordier , S. Warriner , A. Nelson , Angew. Chem. Int. Ed. 2009, 48, 104–109; 10.1002/anie.200804486 PMC2633658 19016294

[18a] S. Fustero , R. Román , A. Asensio , M. A. Maestro , J. A. Aceña , A. Simón-Fuentes , Eur. J. Org. Chem. 2013, 7164–7174;

[18b] A. J. Eberhart , C. Cicoira , D. J. Procter , Org. Lett. 2013, 15, 3994–3997; 23855635 10.1021/ol401786d PMC3819180

[18c] T. A. Moss , Tetrahedron Lett. 2013, 54, 993–997;

[18d] S. Ahamad , A. K. Gupta , R. Kant , K. Mohanan , Org. Biomol. Chem. 2015, 13, 1492–1499; 25476791 10.1039/c4ob02365j

[18e] J. Velcicky , D. Pflieger , Synlett 2010, 1397–1401;

[18f] J. Luo , Z. Huo , J. Fu , F. Jin , Y. Yamamoto , Org. Biomol. Chem. 2015, 13, 3227–3235; 25633240 10.1039/c4ob02567a

[18g] A. van den Hoogenband , J. A. J. den Hartog , N. Faber-Hilhorst , J. H. M. Lange , J. W. Terpstra , Tetrahedron Lett. 2009, 50, 5040–5043.

[19a] P. Fackler , S. M. Huber , T. Bach , J. Am. Chem. Soc. 2012, 134, 12869–12878; 22823342 10.1021/ja305890c

[19b] P. Mukherjee , S. J. S. Roy , T. K. Sarkar , Org. Lett. 2010, 12, 2472–2475. 20443572 10.1021/ol100557f

[20] G. Pelletier , e-EROS Encycl. Reagents Org. Synth. 2011, 1–5.

[21] M. Congreve , R. Carr , C. Murray , H. Jhoti , Drug Discovery Today 2003, 8, 876–877. 10.1016/s1359-6446(03)02831-9 14554012

[22a] J. E. Aaseng , S. Melnes , G. Reian , O. R. Gautun , Tetrahedron 2010, 66, 9790–9797;

[22b] H. Doucet , E. Fernandez , T. P. Layzell , J. M. Brown , Chem. Eur. J. 1999, 5, 1320–1330;

