# Supplementary material for: Partially Saturated Bicyclic Heteroaromatics as an sp3‐Enriched Fragment Collection
Source: Angew Chem Int Ed Engl. 2016 Sep 6;55(40):12479–83. doi: 10.1002/anie.201606496 (PMC5091628; doi:10.1002/anie.201606496)

Angewandte Chemie
Eine Zeitschrift der Gesellschaft Deutscher Chemiker

Supporting Information

Partially Saturated Bicyclic Heteroaromatics as an sp^3-Enriched Fragment Collection

*David G. Twigg, Noriyasu Kondo, Sophie L. Mitchell, Warren R. J. D. Galloway, Hannah F. Sore, Andrew Madin, and David R. Spring**

anie_201606496_sm_miscellaneous_information.pdf

Contents

1. General Experimental Details 2
2. Procedures and Analytical Data 3
3. Computational evaluation of physicochemical properties 39
4. References 43
5. NMR Spectra 44

1. General Experimental Details

All non-aqueous reactions were performed under a constant stream of dry nitrogen using glassware that had been oven-dried overnight. Standard practices were employed when handling moisture- and air-sensitive materials.[1]

Room temperature (rt) refers to ambient temperature. All temperatures below 0 ^{o}C are that of the external bath. Temperatures of 0 ^{o}C were maintained using an ice-water bath. Temperatures below 0 ^{o}C were maintained using an acetone-cardice bath.

All reagents and solvents were used as received unless otherwise stated. CH_2Cl_2, EtOAc, MeOH, MeCN and toluene was distilled from CaH_2. Tetrahydrofuran (THF) was dried over Na wire and distilled from a mixture of $LiAlH_4$ and CaH_2 with triphenylmethane as the indicator. Et_2O was distilled from a mixture of $LiAlH_4$ and CaH_2. Petroleum ether was distilled before use and refers to the fraction between 40-60 ^{o}C. *n*-Butyllithium in hexanes (Aldrich) was titrated with *N*-benzylbenzamide by the method of Chong *et al.* before use.[2]

Yields refer to chromatographically and spectroscopically pure compounds unless otherwise stated. Where possible, reactions were monitored by thin layer chromatography (TLC) performed on commercially prepared glass plates pre-coated with Merck silica gel F_{254}. Visualisation was by the quenching of ultraviolet (UV) fluorescence (λ_{max} = 254 nm) or by staining with potassium permanganate.

Flash column chromatography was carried out using slurry-packed Merck 9385 Keiselgel 60 SiO_2 (230-400 mesh) under a positive pressure of dry nitrogen. Additionally, Combiflash® (Teledyne ISCO), an automated chromatography system, was used for purification of some compounds.

Infrared (IR) spectra were recorded neat on a Perkin-Elmer Spectrum One spectrometer with internal referencing. Selected absorption maxima (ν_{max}) are reported in wavenumbers (cm^{-1}).

^{1}H, ^{13}C and ^{19}F nuclear magnetic resonance (NMR) spectra were recorded using an internal deuterium lock at ambient probe temperature (unless otherwise stated) on the following instruments: Bruker DPX-400 (400 MHz), Bruker Avance 400 QNP (400 MHz) and Bruker Avance 500 Cryo Ultrashield (500 MHz). For ^{1}H NMR, chemical shifts (δ) are quoted in parts per million (ppm), to the nearest 0.01 ppm, and are referenced to the residual non-deuterated solvent peak. Coupling constants (*J*) are reported in Hertz (Hz) to the nearest 0.1 Hz. Data are reported as followed: chemical shift, multiplicity (s = singlet; d = doublet; t = triplet; q = quartet; qn = quintet; m = multiplet; or as a combination of these, eg. dd, dt etc.; app = apparent; br = broad), integration and coupling constant(s). The internal standard used was tetramethylsilane. For ^{13}C NMR, chemical shifts (δ) are quoted in ppm, to the nearest 0.1 ppm, and are referenced to the solvent peak. Coupling constants (*J*) to ^{19}F nuclei are reported in Hertz (Hz) to the nearest 0.1 Hz. The internal standard used was tetramethylsilane. For ^{19}F NMR, chemical shifts (δ) are quoted in ppm, to the nearest 0.1 ppm, and are referenced to the internal standard, trichlorofluoromethane.

High resolution mass spectrometry (HRMS) measurements were recorded on a Bruker Bioapex 4.7e FTICR or a Micromass LCT Premier spectrometer. Mass values are quoted within the error limits of ±5 ppm mass units. ESI refers to the electrospray mass ionisation technique.

2. Procedures and Analytical Data

3-Nitro-1-((2-(trimethylsilyl)ethoxy)methyl)-1*H*-pyrazole, **5**

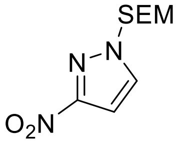

NaH (60 wt% in mineral oil, 2.92 g, 73.1 mmol) was added to a solution of 3-nitro-1*H*-pyrazole **4** (5.51 g, 48.7 mmol) in THF (55 mL) at 0 °C and the mixture stirred for 5 min. SEM-Cl (11.2 mL, 63.3 mmol) was added and the mixture stirred at rt for a further 3 h. The reaction was quenched with saturated aqueous NH_4Cl, the layers separated and the aqueous phase extracted with EtOAc (2x). The combined organic fractions were washed with brine, dried over $MgSO_4$ and evaporated. The residue was purified by flash column chromatography (petroleum ether/EtOAc, 12:1-5:1) to provide **5** as a yellow solid (10.0 g, 41.3 mmol, 85%).

^{1}H NMR (500 MHz, $CDCl_3$): δ 7.66 (d, 1H, *J* = 2.4 Hz), 6.98 (d, 1H, *J* = 2.4 Hz), 5.50 (s, 2H), 3.63-3.60 (m, 2H), 0.94-0.91 (m, 2H), -0.01 (s, 9H); **^{13}C NMR** (126 MHz, $CDCl_3$): δ 156.0, 131.7, 103.8, 81.8, 67.9, 17.8, -1.5; **IR** ν_{max}: 3141, 3114, 2953, 2930, 1539, 1504, 1374, 1295, 1247, 1179, 1090, 1058, 940, 921, 834, 822, 800, 751, 695; **HRMS** (ESI): $[M+Na]^+$ calcd. for $C_9H_{17}N_3NaO_3Si$: 266.0931, found: 266.0927.

5-Iodo-3-nitro-1-((2-(trimethylsilyl)ethoxy)methyl)-1*H*-pyrazole, **6**

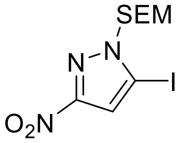

To a solution of *i*-Pr_2NH (6.92 mL, 49.4 mmol) in THF (40 mL) was added *n*-BuLi (1.6 M in hexanes, 30.8 mL, 49.4 mmol) at 0 °C. After 1 h, the resulting solution was added to a solution of **5** (10.0 g, 41.2 mmol) in THF (40 mL) at -78 °C. After stirring at this temperature for 1 h, a solution of I_2 (12.5 g, 49.4 mmol) in THF (20 mL) was added and the reaction allowed to warm to rt and stirred for 2 h. The reaction was quenched with 10% (w/v) aqueous $Na_2S_2O_3$ then the reaction mixture poured onto saturated aqueous NH_4Cl. The layers were separated and the aqueous phase extracted with EtOAc (2x). The combined organic fractions were dried over $MgSO_4$ and evaporated. The residue was purified by flash column chromatography (petroleum ether/EtOAc, 20:1-12:1) to provide **6** as a brown oil (11.2 g, 30.3 mmol, 84%).

^{1}H NMR (500 MHz, $CDCl_3$): δ 7.13 (s, 1H), 5.58 (s, 2H), 3.64 (m, 2H), 0.92 (m, 2H), -0.01 (s, 9H); **^{13}C NMR** (126 MHz, $CDCl_3$): δ 157.2, 113.0, 83.4, 81.7, 67.7, 17.7, -1.4; **IR** ν_{max}: 2951, 2897, 1541, 1484, 1448, 1382, 1295, 1247, 1235, 1092, 1033, 993, 857, 833, 822, 755, 744, 693; **HRMS** (ESI): $[M+Na]^+$ calcd. for $C_9H_{16}IN_3NaO_3Si$: 391.9898, found: 391.9893.

3-Nitro-1-((2-(trimethylsilyl)ethoxy)methyl)-5-vinyl-1*H*-pyrazole, **7a**

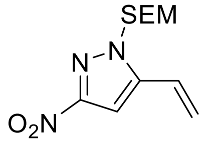

Potassium vinyltrifluoroborate (5.48 g, 41.2 mmol), K_2CO_3 (11.4 g, 82.4 mmol) and Pd(dppf)Cl_2·CH_2Cl_2 (1.00 g, 1.37 mmol) were added to a solution of **6** (10.1 g, 27.5 mmol) in THF (100 mL) and water (20 mL) and the reaction mixture heated to reflux for 17 h. After cooling, the crude mixture was poured onto brine, the layers separated and the aqueous phase extracted with EtOAc (3x). The combined organic fractions were dried over $MgSO_4$ and evaporated. The residue was purified by flash column chromatography (petroleum ether/EtOAc, 20:1-18:1) to provide **7a** as a yellow oil (6.94g, 23.5 mmol, 85%).

^{1}H NMR (500 MHz, $CDCl_3$): δ 7.03 (s, 1H), 6.75 (ddd, 1H, *J* = 17.7, 11.3, 0.4 Hz), 5.90 (dd, 1H, *J* = 17.6, 0.6 Hz), 5.59 (dd, 1H, *J* =11.3, 0.6 Hz), 5.54 (s, 2H), 3.62-3.58 (m, 2H), 0.91-0.89 (m, 2H), -0.03 (s, 9H); **^{13}C NMR** (126 MHz, $CDCl_3$): δ 155.2, 144.4, 122.1, 121.6, 100.1, 79.8, 67.4, 17.7, -1.5; **HRMS** (ESI): $[M+H]^+$ calcd. for $C_{11}H_{20}N_3O_3Si$: 270.1268, found: 270.1265.

3-Nitro-5-(prop-1-en-2-yl)-1-((2-(trimethylsilyl)ethoxy)methyl)-1*H*-pyrazole, **7f**

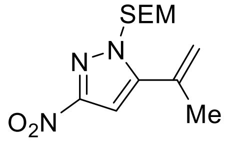

The compound was prepared in an analogous way to **7a**, using **6** (135 mg, 0.366 mmol), potassium isopropenyltrifluoroborate (81 mg, 0.549 mmol), K_2CO_3 (151 mg, 1.10 mmol), Pd(dppf)Cl_2·CH_2Cl_2 (27 mg, 0.037 mmol), THF (1.4 mL) and water (0.3 mL). After purification by flash column chromatography (petroleum ether/EtOAc, 20:1), **7f** was obtained as a yellow oil (79 mg, 0.279 mmol, 76%).

^{1}H NMR (400 MHz, $CDCl_3$): δ 6.85 (s, 1H), 5.64 (app t, 1H, *J* = 0.8 Hz), 5.52 (s, 2H), 5.49-5.48 (m, 1H), 3.75-3.72 (m, 2H), 2.13 (dd, 3H, *J* = 1.5, 0.9 Hz), 0.93-0.90 (m, 2H), -0.02 (s, 9H); **^{13}C NMR** (126 MHz, $CDCl_3$): δ 155.0, 147.5, 131.8, 120.5, 101.9, 79.7, 67.5, 23.2, 17.9, -1.5; **HRMS** (ESI): $[M+H]^+$ calcd. for $C_{12}H_{22}N_3O_3Si$: 284.1425, found: 284.1416.

5-Allyl-3-nitro-1-((2-(trimethylsilyl)ethoxy)methyl)-1*H*-pyrazole, **7g**

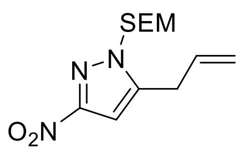

LDA (2.0 M in THF, 4.2 mL, 8.30 mmol) was added dropwise to a stirred solution of **5** (1.68 g, 6.91 mmol) in THF (17 mL) at -78 °C. After stirring for 1 h, CuBr (198 mg, 1.38 mmol) was added and stirring continued for 1 h at -78 °C. Allyl bromide (717 µL, 8.30 mmol) was then added and the reaction stirred at rt for 1 h. The reaction was quenched with saturated aqueous NH_4Cl, the layers were separated and the aqueous phase extracted with EtOAc (2x). The combined organic fractions were washed with

brine, dried over $MgSO_4$ and evaporated. The residue was purified by flash column chromatography (petroleum ether/EtOAc, 20:1) to provide **7g** as a yellow oil (1.20 g, 4.24 mmol, 61%).

^{1}H NMR (500 MHz, $CDCl_3$): δ 6.75 (s, 1H), 5.91 (ddt, 1H, *J* = 16.8, 10.1, 6.5 Hz), 5.48 (s, 2H), 5.24 (app dq, 1H, *J* = 10.1, 1.3 Hz), 5.17 (app dq, 1H, *J* = 16.9, 1.5 Hz), 3.60-3.57 (m, 2H), 3.54-3.52 (m, 2H), 0.92-0.87 (m, 2H), -0.02 (s, 9H); **^{13}C NMR** (126 MHz, $CDCl_3$): δ 155.1, 144.8, 131.9, 118.7, 102.8, 79.6, 67.3, 29.6, 17.8, -1.5; **HRMS** (ESI): $[M+Na]^+$ calcd. for $C_{12}H_{21}N_3NaO_3Si$: 306.1244, found: 306.1235.

3-nitro-5-vinyl-1*H*-pyrazole, **8a**

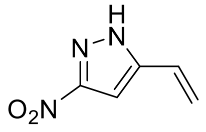

TFA (4.98 mL, 64.6 mmol) was added to a solution of **7a** (1.74g, 6.46 mmol) in CH_2Cl_2 (17 mL). After stirring at room temperature for 22 h, the reaction mixture was evaporated. The residue was purified by flash column chromatography on amine-functionalised silica ($CHCl_3$/MeOH, 95:5) to provide **8a** as a white solid (721 mg, 5.18 mmol, 80%).

^{1}H NMR (400 MHz, $CDCl_3$): δ 12.52 (br s, 0.2H), 6.98 (s, 1H), 6.71 (dd, 1H, *J* = 17.7, 11.3 Hz), 5.89 (d, 1H, *J* = 17.7 Hz), 5.58 (d, 1H, *J* = 11.2 Hz); **^{13}C NMR** (101 MHz, $CDCl_3$): δ 156.9, 144.8, 122.8, 120.6, 98.8; **IR** ν_{max}: 3350, 3147, 2968, 1634, 1540, 1524, 1482, 1386, 1339, 1292, 1206, 1126, 1083, 1062, 1006, 998, 976, 943, 832, 816, 777, 755, 679; **HRMS** (ESI): $[M+Na]^+$ calcd. for $C_5H_5N_3NaO_2$: 162.0274, found: 162.0271.

3-Nitro-5-(prop-1-en-2-yl)-1*H*-pyrazole, **8f**

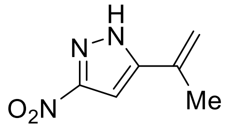

The compound was prepared in an analogous way to **8a**, using **7f** (75 mg, 0.265 mmol), TFA (0.20 mL, 2.65 mmol) and CH_2Cl_2 (0.75 mL). After purification by flash column chromatography (petroleum ether/EtOAc, 5:1), **8f** was obtained as a white solid (28 mg, 0.183 mmol, 69%).

^{1}H NMR (500 MHz, $CDCl_3$): δ 10.94 (br s, 1H), 6.90 (s, 1H), 5.54 (app q, 1H, *J* = 0.9 Hz), 5.31 (app q, 1H, *J* = 1.6 Hz), 2.14 (dd, 3H, *J* = 1.5, 0.9 Hz); **^{13}C NMR** (126 MHz, $CDCl_3$): δ 157.1, 146.3, 130.8, 115.5, 98.9, 20.2; **HRMS** (ESI): $[M+H]^+$ calcd. for $C_6H_8N_3O_2$: 154.0617, found: 154.0612.

5-Allyl-3-nitro-1*H*-pyrazole, **8g**

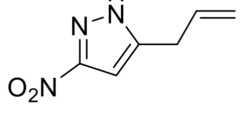

The compound was prepared in an analogous way to **8a**, using **7g** (954 mg, 3.37 mmol), TFA (2.60 mL, 33.7 mmol) and CH_2Cl_2 (9 mL). After purification by flash column chromatography on amine-functionalised silica ($CHCl_3$/MeOH, 95:5), **8g** was obtained as a yellow oil (422 mg, 2.76 mmol, 82%).

^{1}H NMR (400 MHz, $CDCl_3$): δ 12.61 (s, 1H), 6.75 (s, 1H), 5.97-5.87 (m, 1H), 5.21-5.17 (m, 2H), 3.58 (d, 2H, *J* = 6.5 Hz); **^{13}C NMR** (101 MHz, $CDCl_3$): δ 156.8, 145.7, 132.4, 118.8, 100.8, 30.2; **HRMS** (ESI): $[M+H]^+$ calcd. for $C_6H_8N_3O_2$: 154.0617, found: 154.0614.

1-Allyl-3-nitro-5-vinyl-1*H*-pyrazole, **9a**

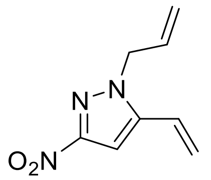

NaH (60 wt% in mineral oil, 65 mg, 1.63 mmol) was added to a solution of **8a** (151 mg, 1.09 mmol) in THF (14 mL) at 0 °C and the reaction mixture stirred for 5 min. Allyl bromide (141 μL, 1.63 mmol) was then added and the reaction heated to reflux for 15 h. After cooling, the reaction was quenched with saturated aqueous NH_4Cl, the layers were separated and the aqueous phase extracted with EtOAc (2x). The combined organic fractions were washed with brine, dried over $MgSO_4$ and evaporated. The residue was purified by flash column chromatography (petroleum ether/EtOAc, 10:1) to provide **9a** as a yellow oil (107 mg, 0.598 mmol, 55%).

^{1}H NMR (400 MHz, $CDCl_3$): δ 7.00 (s, 1H), 6.55 (dd, 1H, *J* = 17.4, 11.2 Hz), 5.96 (ddt, 1H, *J* = 17.1, 10.6, 5.4 Hz), 5.84 (d, 1H, *J* = 17.4 Hz), 5.56 (d, 1H, *J* = 11.2 Hz), 5.31 (app d, 1H, *J* = 10.6 Hz), 5.12 (dt, 1H, *J* = 17.0, 1.5 Hz), 4.86 (dt, 2H, *J* = 5.4, 1.6 Hz); **^{13}C NMR** (101 MHz, $CDCl_3$): δ 155.1, 143.8, 131.1, 121.9, 121.4, 119.0, 99.5, 53.5; **HRMS** (ESI): $[M+H]^+$ calcd. for $C_8H_{10}N_3O_2$: 180.0773, found: 180.0773.

1-(But-3-en-1-yl)-3-nitro-5-vinyl-1*H*-pyrazole, **9b**

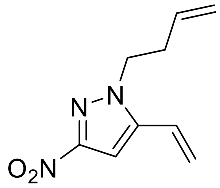

NaH (60 wt% in mineral oil, 314 mg, 7.85 mmol) was added to a solution of **8a** (728 mg, 5.24 mmol) in THF (14 mL) and the reaction mixture stirred for 5 min. 4-bromo-1-butene (1.11 mL, 7.85 mmol) was then added and the reaction heated to reflux for 17 h. The reaction was incomplete so, after cooling, further NaH (60 wt% in mineral oil, 314 mg, 7.85 mmol) and 4-bromo-1-butene (1.11 mL, 7.85 mmol) were added and the reaction heated to reflux for a further 23 h. After cooling, the reaction was quenched with saturated aqueous NH_4Cl and the crude mixture was poured onto brine. The layers were separated and the aqueous phase extracted with EtOAc (2x). The combined organic fractions were dried over $MgSO_4$ and evaporated. The residue was purified by flash column chromatography (petroleum ether/EtOAc, 10:1-4:1) to provide **9b** as a yellow solid (866 mg, 4.48 mmol, 86%).

^{1}H NMR (500 MHz, $CDCl_3$): δ 6.96 (s, 1H), 6.57 (ddd, 1H, *J* = 17.4, 11.2, 0.4 Hz), 5.84 (dd, 1H, *J* = 17.4, 0.6 Hz), 5.77-5.69 (m, 1H), 5.57 (dd, 1H, *J* = 11.2, 0.6 Hz), 5.10-5.06 (m, 2H), 4.26 (t, 2H, *J* = 7.3 Hz),

2.64-2.59 (m, 2H); ^{13}**C NMR** (126 MHz, $CDCl_3$): δ 155.0, 143.6, 132.8, 122.0, 121.4, 118.6, 99.2, 50.1, 34.3; **IR** ν_{max}: 3143, 2917, 1637, 1535, 1523, 1470, 1428, 1392, 1328, 1298, 1289, 1208 ,1087, 1000, 983, 921, 910, 829, 811, 756; **HRMS** (ESI): $[M+H]^+$ calcd. for $C_9H_{12}N_3O_2$: 194.0924, found: 194.0923.

3-Nitro-1-(pent-4-en-1-yl)-5-vinyl-1*H*-pyrazole, **9c**

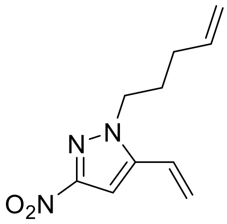

NaH (60 wt% in mineral oil, 14 mg, 0.36 mmol) was added to a solution of **8a** (50 mg, 0.36 mmol) in DMF (1 mL) at 0 °C and the reaction mixture stirred for 5 min. 5-bromo-1-pentene (64 μL, 0.54 mmol) was then added and the reaction stirred at rt for 17 h. The reaction was incomplete so further NaH (60 wt% in mineral oil, 29 mg, 0.72 mmol) and 5-bromo-1-pentene (64 μL, 0.54 mmol) were added and the reaction stirred for a further 24 h. The reaction was quenched with saturated aqueous NH_4Cl and the crude mixture was poured onto water. The layers were separated and the aqueous phase extracted with EtOAc (2x). The combined organic fractions were washed with brine, dried over $MgSO_4$ and evaporated. The residue was purified by flash column chromatography (petroleum ether/EtOAc, 12:1) to provide **9c** as a yellow oil (41 mg, 0.198 mmol, 55%).

^{1}**H NMR** (400 MHz, $CDCl_3$): δ 6.97 (s, 1H), 6.56 (dd, 1H, *J* = 17.4, 11.2 Hz), 5.84 (dd, 1H, *J* = 17.4, 0.5 Hz), 5.77 (ddt, 1H, *J* = 17.0, 10.4, 6.6 Hz), 5.57 (dd, 1H, *J* = 11.2, 0.5 Hz), 5.08-5.04 (m, 1H), 5.04-5.02 (m, 1H), 4.20 (t, 2H, *J* = 7.3 Hz), 2.12-2.07 (m, 2H), 2.02-1.94 (m, 2H); ^{13}**C NMR** (101 MHz, $CDCl_3$): δ 155.0, 143.5, 136.4, 121.9, 121.3, 116.3, 99.3, 50.1, 30.4, 29.1; **HRMS** (ESI): $[M+H]^+$ calcd. for $C_{10}H_{14}N_3O_2$: 208.1086, found: 208.1089.

1-(2-(Allyloxy)ethyl)-3-nitro-5-vinyl-1*H*-pyrazole, **9d**

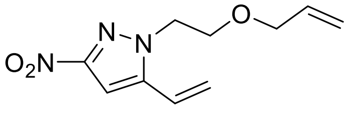

NaH (60 wt% in mineral oil, 34 mg, 0.860 mmol) was added to a solution of **8a** (80 mg, 0.573 mmol) in THF (1 mL) and the reaction mixture stirred for 5 min. A solution of (2-allyloxyethoxy)-*p*-toluenesulfonate **11** (220 mg, 0.869 mmol) in THF (0.5 mL) was then added and the reaction heated to reflux for 20 h. After cooling, the reaction was quenched with saturated aqueous NH_4Cl, the layers were separated and the aqueous phase extracted with $CHCl_3$ (3x). The combined organic fractions were evaporated. The residue was purified by flash column chromatography (petroleum ether/EtOAc, 8:1) to provide **9d** as a white solid (76 mg, 0.341 mmol, 59%).

^{1}**H NMR** (500 MHz, $CDCl_3$): δ 6.97 (s, 1H), 6.72 (dd, 1H, *J* = 17.5, 11.2 Hz), 5.83 (dd, 1H, *J* = 17.5, 0.7 Hz), 5.80-5.74 (m, 1H), 5.55 (dd, 1H, *J* = 11.2, 0.7 Hz), 5.20-5.13 (m, 2H), 4.37 (t, 2H, *J* = 5.2 Hz), 3.92 (dt, 2H, *J* = 5.5, 1.5 Hz), 3.83 (t, 2H, *J* = 5.3 Hz) ; ^{13}**C NMR** (126 MHz, $CDCl_3$): δ 155.3, 145.1, 133.8, 122.7, 121.1, 117.3, 99.0, 72.2, 68.5, 50.9; **IR** ν_{max}: 2985, 2878, 1734, 1541, 1524, 1471, 1391, 1341, 1306,

1241, 1101, 1045, 1004, 925, 829, 807, 757; **HRMS** (ESI): $[M+Na]^+$ calcd. for $C_{10}H_{13}N_3NaO_3$: 246.0849, found: 246.0838.

tert-Butyl (2-(3-nitro-5-vinyl-1*H*-pyrazol-1-yl)ethyl)carbamate, **52**

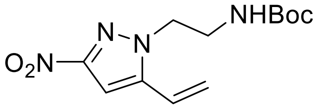

3-Boc-1,2,3-oxathiazolidine 2,2-dioxide **12** (70 mg, 0.317 mmol) was added to a suspension of **8a** (22 mg, 0.158 mmol) and K_2CO_3 (65 mg, 0.474 mmol) in DMF (0.5 mL) and the reaction mixture stirred at rt for 22 h. The reaction was quenched with 1 M aqueous HCl (2 mL). The resulting mixture was poured onto 0.1 M aqueous HCl, the layers were separated and the aqueous phase extracted with EtOAc (2x). The combined organic fractions were dried over $MgSO_4$ and evaporated. The residue was purified by flash column chromatography (petroleum ether/EtOAc, 5:1-3:1) to provide **52** as a white solid (19 mg, 0.067 mmol, 43%).

^{1}H NMR (400 MHz, $CDCl_3$): δ 6.98 (s, 1H), 6.61 (dd, 1H, *J* = 17.3, 11.3 Hz), 5.86 (d, 1H, *J* = 17.3 Hz), 5.57 (d, 1H, *J* = 11.2 Hz), 4.74 (br s, 1H), 4.35 (t, 2H, *J* = 5.4 Hz), 3.57 (app q, 2H, *J* = 5.8 Hz), 1.42 (s, 9H); **^{13}C NMR** (101 MHz, $CDCl_3$): δ 155.9, 155.5, 144.8, 121.8, 121.6, 99.1, 80.1, 49.6, 40.4, 28.3; **IR** ν_{max}: 3355, 3145, 2990, 1675, 1520, 1471, 1393, 1365, 1290, 1277, 1250, 1198, 1164, 1081, 1007, 982, 928, 856, 830, 816, 757; **HRMS** (ESI): $[M+Na]^+$ calcd. for $C_{12}H_{18}N_4NaO_4$: 305.1226, found: 305.1216.

tert-Butyl allyl(2-(3-nitro-5-vinyl-1*H*-pyrazol-1-yl)ethyl)carbamate, **9e**

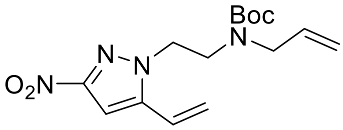

NaH (60 wt% in mineral oil, 3.3 mg, 0.082 mmol) was added to a solution of **52** (15.5 mg, 0.055 mmol) in DMF (0.3 mL) at 0 °C and the reaction mixture stirred for 5 min. Allyl iodide (7.5 μL, 0.082 mmol) was then added and the reaction stirred at rt for 2 days. The reaction was quenched with 0.1 M aqueous HCl, the layers were separated and the aqueous phase extracted with EtOAc (2x). The combined organic fractions were washed with brine, dried over $MgSO_4$ and evaporated. The residue was purified by flash column chromatography (petroleum ether/EtOAc, 8:1-7:1) to provide **9e** as a colourless oil (12 mg, 0.073 mmol, 68%).

^{1}H NMR (400 MHz, $CDCl_3$): δ 6.98 (s, 1H), 6.67-6.49 (m, 1H), 5.86 (d, 1H, *J* = 17.4 Hz), 5.65-5.53 (m, 2H), 5.12-4.98 (m, 2H), 4.41-4.33 (m, 2H), 3.63-3.48 (m, 4H), 1.45 (s, 9H); **^{13}C NMR** (101 MHz, $CDCl_3$): δ 155.5, 155.4, 144.8, 133.1, 121.9, 121.3, 116.7, 98.9, 80.4, 51.4, 48.5, 47.5, 28.3; **HRMS** (ESI): $[M+H]^+$ calcd. for $C_{15}H_{23}N_4O_4$: 323.1719, found: 323.1724.

1-(But-3-en-1-yl)-3-nitro-5-(prop-1-en-2-yl)-1*H*-pyrazole, **9f**

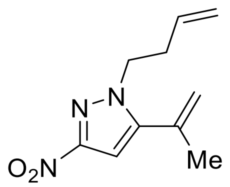

The compound was prepared in an analogous way to **9b**, using **8f** (27 mg, 0.176 mmol), NaH (11 mg, 0.265 mmol) x2, 4-bromo-1-butene (27 μL, 0.265 mmol) x2 and THF (1.0 mL). After purification by flash column chromatography (petroleum ether/EtOAc, 15:1-5:1), **9f** was obtained as a brown oil (16 mg, 0.077 mmol, 44%).

^{1}H NMR (500 MHz, $CDCl_3$): δ 6.75 (s, 1H), 5.76-5.67 (m, 1H), 5.48 (app qn, 1H, *J* = 1.3 Hz), 5.23-5.22 (m, 1H), 5.10-5.08 (m, 1H), 5.06 (app t, 1H, *J* = 1.2 Hz), 4.28 (t, 2H, *J* = 7.5 Hz), 2.68-2.63 (m, 2H), 2.10 (t, 3H, *J* = 1.2 Hz); **^{13}C NMR** (126 MHz, $CDCl_3$): δ 154.9, 147.0, 133.0, 132.8, 120.2, 118.2, 101.3, 50.5, 34.2, 23.6; **IR** ν_{max}: 3151, 2954, 1641, 1562, 1533, 1472, 1441, 1401, 1379, 1324, 1218, 1198, 1016, 1009, 999, 911, 828, 757; **HRMS** (ESI): $[M+H]^+$ calcd. for $C_{10}H_{14}N_3O_2$: 208.1086, found: 208.1096.

1,5-Diallyl-3-nitro-1*H*-pyrazole, **9g**

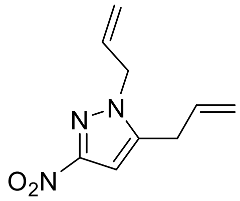

NaH (60 wt% in mineral oil, 6.7 mg, 0.167 mmol) was added to a solution of **8g** (17 mg, 0.111 mmol) in THF (1 mL) at 0 °C and the reaction mixture stirred for 1 min. Allyl bromide (14 μL, 0.167 mmol) was then added and the mixture heated to 60 °C for 4 h. The reaction was quenched with saturated aqueous NH_4Cl, the layers separated and the aqueous phase extracted with $CHCl_3$ (2x). The combined organic fractions were dried over $MgSO_4$ and evaporated. The residue was purified by flash column chromatography (petroleum ether/EtOAc, 6:1) to provide **9g** as a yellow oil (15 mg, 0.078 mmol, 70%).

^{1}H NMR (400 MHz, $CDCl_3$): δ 6.71 (s, 1H), 6.00-5.83 (m, 2H), 5.30 (dd, 1H, *J* = 10.3, 0.5 Hz), 5.25 (dd, 1H, *J* = 10.1, 1.2 Hz), 5.17-5.09 (m, 2H), 4.77 (dt, 2H, *J* = 5.5, 1.5 Hz), 3.40 (d, 1H, *J* = 6.2 Hz); **^{13}C NMR** (126 MHz, $CDCl_3$): δ 155.0, 143.9, 131.8, 131.2, 118.9, 118.8, 102.2, 53.2, 29.9; **HRMS** (ESI): $[M+H]^+$ calcd. for $C_9H_{12}N_3O_2$: 194.0924, found: 194.0919.

5-Allyl-1-(but-3-en-1-yl)-3-nitro-1*H*-pyrazole, **9h**

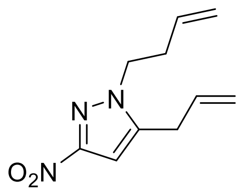

4-bromo-1-butene (78 μL, 0.77 mmol) was added to a suspension of **8g** (78 mg, 0.51 mmol) and Cs_2CO_3 (249 mg, 0.77 mmol) in DMF (0.8 mL) and the reaction mixture stirred for 30 min. Saturated aqueous

NH_4Cl was added and the crude mixture poured onto water, the layers separated and the aqueous phase extracted with EtOAc (2x). The combined organic fractions were washed with brine, dried over $MgSO_4$ and evaporated. The residue was purified by flash column chromatography (petroleum ether/EtOAc, 15:1) to provide **9h** as a colourless oil (38 mg, 0.18 mmol, 36%).

^{1}H NMR (400 MHz, $CDCl_3$): δ 6.66 (s, 1H), 5.89 (ddt, 1H, *J* = 16.8, 10.4, 6.4 Hz), 5.79-5.68 (m, 1H), 5.25 (dd, 1H, *J* = 10.1, 1.2 Hz), 5.17-5.06 (m, 3H), 4.15 (t, 2H, *J* = 7.4 Hz), 3.42 (d, 2H, *J* = 6.2 Hz), 2.66-2.60 (m, 2H); **^{13}C NMR** (101 MHz, $CDCl_3$): δ 155.0, 143.7, 133.0, 132.0, 118.7, 118.5, 101.8, 49.8, 34.1, 30.0; **HRMS** (ESI): $[M+H]^+$ calcd. for $C_{10}H_{14}N_3O_2$: 208.1086, found: 208.1095.

2-Nitro-6*H*-pyrrolo[1,2-*b*]pyrazole, **10a**

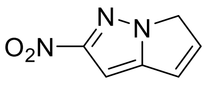

Hoveyda-Grubbs' catalyst, 2nd generation (34 mg, 0.055 mmol) was added to a solution of **9a** (98 mg, 0.547 mmol) in toluene (11 mL) and the reaction mixture heated to reflux for 24 h. After cooling, the solvent was evaporated and the residue purified by flash column chromatography (petroleum ether/EtOAc, 5:1-2:1) to provide **10a** as a brown solid (37 mg, 0.245 mmol, 45%).

^{1}H NMR (400 MHz, $CDCl_3$): δ 6.74-6.70 (m, 3H), 4.78 (app t, 2H, *J* = 1.4 Hz); **^{13}C NMR** (126 MHz, $CDCl_3$): δ 158.8, 149.6, 133.8, 121.4, 93.5, 55.0; **IR** ν_{max}: 3093, 1567, 1517, 1445, 1436, 1400, 1325, 1300, 995, 878, 829, 798, 756, 666; **HRMS** (ESI): $[M+H]^+$ calcd. for $C_6H_5N_3O_2$: 152.0460, found: 152.0455.

2-Nitro-6,7-dihydropyrazolo[1,5-*a*]pyridine, **10b**

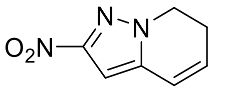

Grubbs' catalyst, 2nd generation (633 mg, 0.75 mmol) was added to a solution of **9b** (1.44 g, 7.46 mmol) in CH_2Cl_2 (30 mL) and the reaction mixture heated to reflux for 20 h. After cooling, the solvent was evaporated and the residue purified by flash column chromatography (petroleum ether/EtOAc, 8:1-3:1) to provide **10b** as a brown solid (1.03 g, 6.24 mmol, 84%).

^{1}H NMR (500 MHz, $CDCl_3$): δ 6.69 (s, 1H), 6.45 (dt, 1H, *J* = 9.9, 1.8 Hz), 6.21 (dt, 1H, *J* = 9.9, 4.5 Hz), 4.30 (t, 2H, *J* = 7.8 Hz), 2.74 (tdd, 2H, *J* = 7.8, 4.5, 1.8 Hz); **^{13}C NMR** (126 MHz, $CDCl_3$): δ 155.5, 139.3, 128.0, 116.9, 98.5, 46.1, 24.0; **IR** ν_{max}: 3147, 2956, 1633, 1524, 1467, 1449, 1409, 1365, 1336, 1323, 1289, 1235, 1203, 1097, 1040, 1000, 925, 885, 835, 811, 755, 657; **HRMS** (ESI): $[M+Na]^+$ calcd. for $C_7H_7N_3NaO_2$: 188.0430, found: 188.0430.

2-Nitro-7,8-dihydro-6*H*-pyrazolo[1,5-*a*]azepine, **10c**

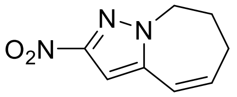

The compound was prepared in an analogous way to **10b**, using **9c** (39 mg, 0.188 mmol), Grubbs II (16 mg, 0.019 mmol) and CH_2Cl_2 (1.5 mL). After purification by flash column chromatography (petroleum ether/EtOAc, 5:1), **10c** was obtained as a brown solid (30 mg, 0.168 mmol, 89%).

^{1}H NMR (500 MHz, $CDCl_3$): δ 6.73 (s, 1H), 6.26 (dt, 1H, *J* = 12.3, 2.1 Hz), 6.07 (dt, 1H, *J* = 12.4, 4.5 Hz), 4.46-4.44 (m, 2H), 2.61-2.57 (m, 2H), 2.19-2.14 (m, 2H); **^{13}C NMR** (126 MHz, $CDCl_3$): δ 154.3, 142.2, 134.4, 115.5, 103.3, 54.9, 31.1, 24.5; **IR** ν_{max}: 3139, 2934, 1524, 1467, 1411, 1377, 1354, 1332, 1303, 1264, 1253, 1004, 831, 818, 197, 159; **HRMS** (ESI): $[M+H]^+$ calcd. for $C_8H_{10}N_3O_2$: 180.0773, found: 180.0774.

(*Z*)-2-Nitro-8,9-dihydro-6*H*-pyrazolo[1,5-d][1,4]oxazocine, **10d**

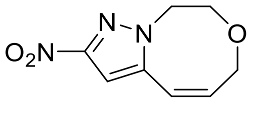

The compound was prepared in an analogous way to **10b**, using **9d** (37 mg, 0.166 mmol), Grubbs II (14 mg, 0.017 mmol) and CH_2Cl_2 (3.3 mL). After purification by flash column chromatography (petroleum ether/EtOAc, 3:1-2:1), **10d** was obtained as a brown solid (21 mg, 0.108 mmol, 65%).

^{1}H NMR (500 MHz, $CDCl_3$): δ 6.79 (s, 1H), 6.31 (dt, 1H, *J* = 12.5, 2.1 Hz), 5.96 (dt, 1H, *J* = 12.5, 3.3 Hz), 4.45 (t, 2H, *J* = 5.3 Hz), 4.39 (app t, 2H, *J* = 2.8 Hz), 3.92 (t, 2H, *J* = 5.3 Hz); **^{13}C NMR** (126 MHz, $CDCl_3$): δ 155.3, 142.9, 136.0, 114.5, 102.0, 69.8, 68.9, 50.1; **IR** ν_{max}: 3147, 2924, 1533, 1470, 1462, 1408, 1365, 1309, 1262, 1243, 1208, 1109, 1014, 834, 817, 758, 718; **HRMS** (ESI): $[M+H]^+$ calcd. for $C_8H_{10}N_3O_3$: 196.0722, found: 196.0725.

tert-Butyl (*Z*)-2-nitro-8,9-dihydropyrazolo[1,5-*d*][1,4]diazocine-7(6*H*)-carboxylate, **10e**

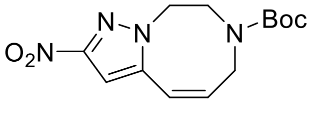

The compound was prepared in an analogous way to **10b**, using **9e** (12 mg, 0.037 mmol), Grubbs II (3 mg, 0.004 mmol) and CH_2Cl_2 (1.9 mL). After purification by flash column chromatography (petroleum ether/EtOAc, 3:2), **10e** was obtained as a brown solid (9 mg, 0.031 mmol, 83%). NMR spectroscopy revealed a 7:3 mixture of rotamers at room temperature.

^{1}H NMR (500 MHz, $CDCl_3$): δ 6.74 (s, 0.7H), 6.71 (s, 0.3H), 6.32-6.25 (m, 1H), 6.03-5.97 (m, 1H), 4.37 (t, 2H, *J* = 5.7 Hz), 4.12 (br s, 1.4H), 4.00 (br s, 0.6H), 3.78 (t, 2H, *J* = 5.0 Hz), 1.32 (s, 2.7H), 1.19 (s, 6.3H); **^{13}C NMR** (126 MHz, $CDCl_3$): δ 155.4, 155.3, 154.3, 154.2, 142.3, 141.7, 135.0, 134.6, 115.3, 114.3, 102.2, 102.1, 80.6, 80.5, 50.2, 49.1, 47.6, 47.2, 46.7, 45.4, 28.2, 28.0; **IR** ν_{max}: 3139, 2932, 1694, 1535, 1471, 1449, 1417, 1402, 1361, 1314, 1242, 1214, 1166, 1141, 1005, 940, 849, 831, 762, 655; **HRMS** (ESI): $[M+Na]^+$ calcd. for $C_{13}H_{18}N_4NaO_4$: 317.1226, found: 317.1224.

4-Methyl-2-nitro-6,7-dihydropyrazolo[1,5-*a*]pyridine, **10f**

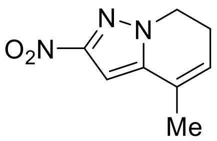

The compound was prepared in an analogous way to **10b**, using **9f** (85 mg, 0.411 mmol), Grubbs II (35 mg, 0.041 mmol) and CH_2Cl_2 (4.0 mL). After purification by flash column chromatography (petroleum ether/EtOAc, 7:1-4:1), **10f** was obtained as a green solid (66 mg, 0.369 mmol, 90%).

^{1}H NMR (500 MHz, $CDCl_3$): δ 6.73 (s, 1H), 5.90-5.87 (m, 1H), 4.25 (t, 2H, *J* = 7.8 Hz), 2.70-2.64 (m, 2H), 2.03 (app q, 3H, *J* = 1.8 Hz); **^{13}C NMR** (126 MHz, $CDCl_3$): δ 155.3, 142.1, 124.9, 122.8, 97.3, 46.1, 23.8, 17.8; **IR** ν_{max}: 3151, 2945, 2921, 1645, 1528, 1461, 1443, 1407, 1348, 1332, 1292, 1207, 1195, 1002, 829, 811, 756, 715, 695; **HRMS** (ESI): $[M+H]^+$ calcd. for $C_8H_{10}N_3O_2$: 180.0773, found: 180.0776.

2-Nitro-4,7-dihydropyrazolo[1,5-*a*]pyridine, **10g**

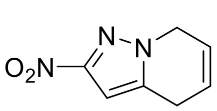

The compound was prepared in an analogous way to **10b**, using **9g** (10 mg, 0.052 mmol), Grubbs II (4 mg, 0.005 mmol) and CH_2Cl_2 (1.0 mL). After purification by flash column chromatography (petroleum ether/EtOAc, 3:1), **10g** was obtained as a white solid (8 mg, 0.048 mmol, 93%).

^{1}H NMR (400 MHz, $CDCl_3$): δ 6.70 (s, 1H), 6.06-5.97 (m, 2H), 4.80-4.77 (m, 2H), 3.54-3.51 (m, 2H); **^{13}C NMR** (101 MHz, $CDCl_3$): δ 155.8, 139.1, 121.0, 120.3, 99.3, 47.9, 24.0; **IR** ν_{max}: 3137, 2923, 1532, 1483, 1416, 1404, 1388, 1332, 1247, 1008, 893, 832, 816, 758, 672; **HRMS** (ESI): $[M+H]^+$ calcd. for $C_7H_8N_3O_2$: 166.0617, found: 166.0617.

2-Nitro-7,8-dihydro-4*H*-pyrazolo[1,5-*a*]azepine, **10h**

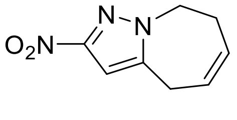

Hoveyda-Grubbs' catalyst, 2nd generation (6.6 mg, 0.011 mmol) was added to a solution of **9h** (21.8 mg, 0.105 mmol) in CH_2Cl_2 (1 mL) and the reaction mixture stirred at rt for 2 h. After cooling, 2 drops of DMSO were added then the solvent evaporated. The residue was purified with by flash column chromatography (petroleum ether/EtOAc, 5:1) to provide **10h** as a brown solid (11 mg, 0.061 mmol, 59%).

^{1}H NMR (500 MHz, $CDCl_3$): δ 6.65 (s, 1H), 5.78-5.71 (m, 2H), 4.55-4.53 (m, 2H), 3.52-3.50 (m, 2H), 2.53-2.50 (m, 2H); **^{13}C NMR** (126 MHz, $CDCl_3$): δ 154.1, 145.4, 128.8, 122.8, 101.4, 50.6, 27.9, 24.7; **IR** ν_{max}: 3123, 3025, 2913, 1655, 1547, 1521, 1471, 1450, 1429, 1413, 1395, 1347, 1322, 1303, 1245, 1182, 1119, 1001, 924, 844, 824, 807, 758, 710, 691; **HRMS** (ESI): $[M+H]^+$ calcd. for $C_8H_9N_3O_2$: 180.0883, found: 180.0777.

N,N-(bis-Boc)-5-bromo-4-methylpyridin-2-amine, **14**

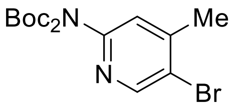

Di-tert-butyl dicarbonate (15.1 g, 68.9 mmol) and DMAP (337 mg, 2.76 mmol) were added to a solution of 2-amino-5-bromo-4-methylpyridine **13** (5.16 g, 27.6 mmol) in THF (335 mL) and the resulting solution heated to reflux for 18 h. After cooling, the solvent was evaporated and the residue purified by flash column chromatography (petroleum ether/EtOAc, 19:1-9:1) to provide **14** as a white solid (9.96 g, 25.7 mmol, 93%).

^{1}H NMR (500 MHz, $CDCl_3$): δ 8.50 (s, 1H), 7.15 (s, 1H), 2.41 (s, 3H), 1.46 (s, 18H); **^{13}C NMR** (126 MHz, $CDCl_3$): δ 151.4, 150.2, 149.1, 123.7, 121.7, 83.6, 28.1, 22.5; **IR** ν_{max}: 2985, 1756, 1718, 1593, 1460, 1394, 1367, 1316, 1286, 1249, 1160, 1114, 1061, 1041, 937, 904, 867, 855, 818, 806, 773, 753, 722, 691; **HRMS** (ESI): $[M+Na]^+$ calcd. for $C_{16}H_{23}N_2O_4NaBr$: 409.0739, found: 409.0758.

N,N-(bis-Boc)-4-methyl-5-vinylpyridin-2-amine, **15a**

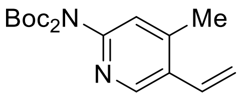

A flask charged with **14** (2.60 g, 6.71 mmol), potassium vinyltrifluoroborate (1.35 g, 10.1 mmol), $Pd(dppf)Cl_2 \cdot CH_2Cl_2$ (548 mg, 0.67 mmol) and K_2CO_3 (2.78 g, 20.1 mmol) was thoroughly de-gassed with nitrogen. THF (60 mL) and water (6 mL) were added and the solution heated to reflux for 20 h. After cooling, the reaction was filtered through Celite and evaporated. The residue was re-dissolved in 1:1 CH_2Cl_2:H_2O, the layers separated and the aqueous phase extracted with CH_2Cl_2 (3x). The combined organic fractions were dried over $MgSO_4$ and evaporated. The residue was purified by flash column chromatography (petroleum ether/EtOAc, 19:1-9:1) to provide **15a** as a white solid (1.90 g, 5.69 mmol, 85%).

^{1}H NMR (400 MHz, $CDCl_3$): δ 8.48 (s, 1H), 7.02 (s, 1H), 6.81 (dd, 1H, *J* = 11.4, 17.6 Hz), 5.71 (dd, 1H, *J* = 17.5, 1.2 Hz), 5.41 (dd, 1H, *J* = 11.1, 1.1 Hz), 2.35 (s, 3H), 1.46 (s, 18H); **^{13}C NMR** (101 MHz, $CDCl_3$): δ 151.7, 151.3, 146.4, 146.1, 132.2, 131.5, 122.7, 117.9, 83.2, 28.1, 19.5; **IR** ν_{max}: 2983, 2934, 1736, 1700, 1597, 1479, 1341, 1270, 1239, 1160, 1143, 1124, 1058, 994, 910, 863, 809, 774, 754; **HRMS** (ESI): $[M+H]^+$ calcd. for $C_{18}H_{27}N_2O_4$: 335.1971, found: 335.1971.

N,N-(bis-Boc)-4-methyl-5-(prop-1-en-2-yl)pyridin-2-amine, **15d**

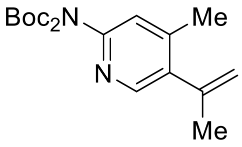

The compound was prepared in an analogous way to **15a**, using **14** (289 mg, 0.75 mmol), potassium isopropenyltrifluoroborate (166 mg, 1.12 mmol), $Pd(dppf)Cl_2 \cdot CH_2Cl_2$ (61 mg, 0.075 mmol), K_2CO_3 (309 mg, 2.24 mmol), THF (7 mL) and water (0.7 mL). After purification by flash column chromatography (petroleum ether/EtOAc, 19:1-9:1), **15d** was obtained as a colourless oil (226 mg, 0.65 mmol, 87%).

^{1}H NMR (500 MHz, $CDCl_3$): δ 8.20 (s, 1H), 7.04 (s, 1H), 5.29 (qn, 1H, *J* = 1.6 Hz), 4.93-4.92 (m, 1H), 2.32 (s, 3H), 2.03 (m, 3H), 1.47 (s, 18H); **^{13}C NMR** (126 MHz, $CDCl_3$): δ 151.9, 150.9, 147.6, 146.4, 141.9, 138.6, 122.9, 117.1, 83.2, 28.1, 24.1, 19.6; **IR** ν_{max}: 2979, 2932, 1794, 1755, 1724, 1596, 1480, 1367, 1340, 1296, 1270, 1247, 1150, 1104, 1050, 901, 853, 806, 775, 748; **HRMS** (ESI): $[M+Na]^+$ calcd. for $C_{19}H_{28}N_2O_4Na$: 371.1947, found: 371.1933.

N,N-(bis-Boc)-4-methyl-5-(1-phenylvinyl)pyridin-2-amine, **15e**

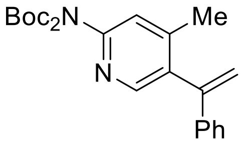

The compound was prepared in an analogous way to **15a**, using **14** (400 mg, 1.03 mmol), 1-phenylvinylboronic acid MIDA ester (401 mg, 1.55 mmol), $Pd(dppf)Cl_2{\cdot}CH_2Cl_2$ (84 mg, 0.103 mmol), K_2CO_3 (428 mg, 3.10 mmol), THF (10 mL) and water (1 mL). After purification by flash column chromatography (petroleum ether/EtOAc, 19:1-9:1), **15e** was obtained as a colourless oil (390 mg, 0.95 mmol, 92%).

^{1}H NMR (500 MHz, $CDCl_3$): δ 8.31 (s, 1H), 7.30-7.28 (m, 3H), 7.24-7.22 (m, 2H), 7.09 (s, 1H), 5.84 (d, 1H, *J* = 1.1 Hz), 5.28 (d, 1H, *J* = 1.1 Hz), 2.03 (s, 3H), 1.50 (s, 18H); **^{13}C NMR** (126 MHz, $CDCl_3$): δ 151.8, 151.8, 149.0, 148.1, 145.6, 139.8, 136.5, 128.7, 128.2, 126.5, 122.8, 116.8, 83.3, 28.1, 20.0; **IR** ν_{max}: 2979, 2932, 1793, 1756, 1725, 1597, 1480, 1368, 1340, 1297, 1252, 1150, 1111, 1070, 909, 851, 779, 735, 711; **HRMS** (ESI): $[M+H]^+$ calcd. for $C_{24}H_{31}N_2O_4$: 411.2284, found: 411.2272.

5-Allyl-*N,N*-(bis-Boc)-4-methylpyridin-2-amine, **15f**

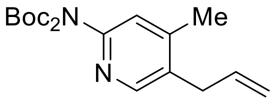

Allyltributylstannane (0.87 mL, 2.84 mmol) was added to a solution of **14** (1.00 g, 2.58 mmol), $Pd(PPh_3)_4$ (149 mg, 0.129 mmol) and KF (300 mg, 5.16 mmol) in toluene (25 mL) and the reaction mixture heated to reflux for 20 h. After cooling, 2 M aqueous KF (10 mL) was added and the reaction stirred vigorously for 15 min. The crude mixture was then filtered through Celite and the filtrate diluted with EtOAc. The layers were separated and the aqueous phase extracted with EtOAc (2x). The combined organic fractions were dried over $MgSO_4$ and evaporated. The residue was purified by flash column chromatography (petroleum ether/EtOAc, 19:1-9:1) to provide **15f** as a pale yellow oil (701 mg, 2.01 mmol, 78%).

^{1}H NMR (400 MHz, $CDCl_3$): δ 8.20 (s, 1H), 7.01 (s, 1H), 5.92 (ddt, 1H, *J* = 17.1, 10.2, 6.0 Hz), 5.09 (dd, 1H, *J* = 10.1, 1.5 Hz), 4.94 (dd, 1H, *J* = 17.1, 1.6 Hz), 3.37 (d, 2H, *J* = 5.9 Hz), 2.29 (s, 3H), 1.45 (s, 18H); **^{13}C NMR** (101 MHz, $CDCl_3$): δ 151.7, 150.8, 149.1, 148.1, 135.2, 132.8, 123.2, 116.6, 83.0, 34.4, 28.0, 19.0; **IR** ν_{max}: 2981, 1790, 1754, 1724, 1604, 1480, 1393, 1368, 1342, 1303, 1273, 1247, 1152, 1112, 1055, 912, 852, 730; **HRMS** (ESI): $[M+Na]^+$ calcd. for $C_{19}H_{28}N_2O_4Na$: 371.1941, found: 371.1933.

5-Allyl-2-chloro-4-methylpyridine, **15g**

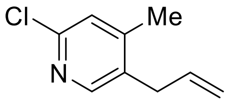

i-PrMgCl·LiCl (1.0 M in THF, 58.1 mL, 72.6 mmol) was added dropwise to a solution of 2-chloro-5-bromo-4-methylpyridine **18** (10.0 g, 48.4 mmol) in THF (20 mL) at -15 °C. After stirring for 2 h, allyl bromide (5.02 mL, 58.1 mmol) was added dropwise and the reaction stirred for 2 h. After warming to rt, the reaction was quenched with saturated aqueous NH_4Cl, the layers separated and the aqueous phase extracted with Et_2O (3x). The combined organic fractions were washed with brine, dried over $MgSO_4$ and evaporated. The residue was purified by flash column chromatography (hexane/Et_2O, 99:1-24:1) to provide **15g** as a colourless oil (5.87 g, 35.1 mmol, 73%).

^{1}H NMR (400 MHz, $CDCl_3$): δ 8.07 (s, 1H), 7.09 (s, 1H), 5.87 (ddt, 1H, *J* = 17.2, 10.0, 6.0 Hz), 5.08 (dq, 1H, *J* = 10.0, 1.6 Hz), 4.94 (dq, 1H, *J* = 17.2, 1.6 Hz), 3.31 (dt, 2H, *J* = 6.0, 1.6 Hz), 2.25 (s, 3H); **^{13}C NMR** (101 MHz, $CDCl_3$): δ 149.7, 149.5, 149.2, 134.8, 132.8, 125.1, 116.8, 34.1, 18.8; **IR** ν_{max}: 2980, 2911, 1638, 1587, 1552, 1471, 1436, 1351, 1090, 994, 889, 863, 738; **HRMS** (ESI): $[M+H]^+$ calcd. for $C_9H_{11}NCl$: 168.0575, found: 168.0570.

N,N-(bis-Boc)-4-(but-3-en-1-yl)-5-vinylpyridin-2-amine, **16a**

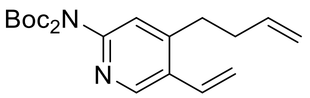

LDA (2.0 M in THF/heptane/ethylbenzene, 3.32 mL, 6.63 mmol) was added dropwise to a solution of **15a** (1.85 g, 5.53 mmol) in THF (55 mL) at -78 °C. After stirring for 25 min, allyl bromide (0.72 mL, 8.29 mmol) was added dropwise and the reaction stirred for 3 h. After warming to 0 °C the reaction was quenched with brine (10 mL) and diluted with EtOAc. The layers were separated and the aqueous phase extracted with EtOAc (3x). The combined organic fractions were dried over $MgSO_4$ and evaporated. The residue was purified by flash column chromatography (petroleum ether/EtOAc, 19:1-9:1) to provide **16a** as a colourless oil (1.24 g, 3.30 mmol, 60%).

^{1}H NMR (500 MHz, $CDCl_3$): δ 8.51 (s, 1H), 7.01 (s, 1H), 6.86 (dd, 1H, *J* = 17.4, 11.1 Hz), 5.81 (ddt, 1H, *J* = 16.9, 10.2, 6.4 Hz), 5.72 (dd, 1H, *J* = 17.5, 1.0 Hz), 5.42 (dd, 1H, *J* = 11.1, 1.1 Hz), 5.05 (dd, 1H, *J* = 17.1, 1.6 Hz), 5.01 (dd, 1H, *J* = 10.1, 1.7 Hz), 2.76 (t, 2H, *J* = 7.6 Hz), 2.36-2.31 (m, 2H), 1.45 (s, 18H); **^{13}C NMR** (126 MHz, $CDCl_3$): δ 151.5, 151.5, 149.8, 146.4, 137.0, 131.7, 131.3, 121.7, 118.2, 115.9, 83.1, 33.7, 32.1, 28.1; **IR** ν_{max}: 2979, 2933, 1791, 1756, 1726, 1595, 1479, 1368, 1342, 1305, 1273, 1250, 1151, 1097, 1058, 912, 852, 807, 776; **HRMS** (ESI): $[M+H]^+$ calcd. for $C_{21}H_{31}N_2O_4$: 375.2284, found: 375.2301.

N,N-(bis-Boc)-4-(3-methylbut-3-en-1-yl)-5-vinylpyridin-2-amine, **16b**

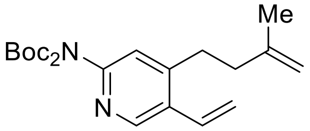

The compound was prepared in an analogous way to **16a**, using LDA (0.13 mL, 0.25 mmol), **15a** (70 mg, 0.21 mmol), 3-bromo-2-methylpropene (32 μL, 0.31 mmol) and THF (2.1 mL). After purification

by flash column chromatography (petroleum ether/EtOAc, 19:1-9:1), **16b** was obtained as a white solid (56 mg, 0.14 mmol, 69%).

^{1}H NMR (500 MHz, $CDCl_3$): δ 8.50 (s, 1H), 7.02 (s, 1H), 6.86 (dd, 1H, *J* = 17.5, 11.1 Hz), 5.73 (dd, 1H, *J* = 17.5, 1.1 Hz), 5.42 (dd, 1H, *J* = 11.1, 1.1 Hz), 4.76 (app s, 1H), 4.70 (d, 1H, *J* = 0.9 Hz), 2.82-2.78 (m, 2H), 2.26 (m, 2H), 1.77 (s, 3H), 1.45 (s, 18H); **^{13}C NMR** (126 MHz, $CDCl_3$): δ 151.5, 151.5, 150.2, 146.4, 144.4, 131.7, 131.2, 121.6, 118.2, 111.0, 83.2, 37.6, 31.1, 28.1, 22.7; **IR** ν_{max}: 2977, 2934, 1734, 1697, 1594, 1358, 1273, 1243, 1160, 1121, 1061, 914, 889, 964, 813, 768, 751; **HRMS** (ESI): $[M+Na]^+$ calcd. for $C_{22}H_{32}N_2O_4Na$: 411.2260, found: 411.2258.

N,N-(bis-Boc)-4-(3-trifluoromethylbut-3-en-1-yl)-5-vinylpyridin-2-amine, **16c**

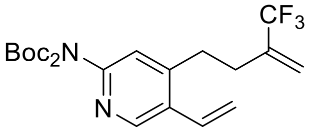

The compound was prepared in an analogous way to **16a**, using LDA (0.54 mL, 1.08 mmol), **15a** (300 mg, 0.897 mmol), 2-bromomethyl-3,3,3-trifluoropropene (0.17 mL, 1.35 mmol) and THF (9 mL). After purification by flash column chromatography (petroleum ether/EtOAc, 9:1), **16c** was obtained as a white solid (305 mg, 0.689 mmol, 77%).

^{1}H NMR (400 MHz, $CDCl_3$): δ 8.52 (s, 1H), 7.03 (s, 1H), 6.83 (dd, 1H, *J* = 17.5, 11.1 Hz), 5.76-5.71 (m, 2H), 5.46 (dd, 1H, *J* = 11.1, 1.0 Hz), 5.30 (app q, 1H, *J* = 1.3 Hz), 2.90-2.86 (m, 2H), 2.49-2.45 (m, 2H), 1.46 (s, 18H); **^{13}C NMR** (101 MHz, $CDCl_3$): δ 151.7, 151.5, 148.4, 146.7, 137.1 (q, *J* = 29.6 Hz), 131.7, 130.8, 123.7 (q, *J* = 273.7 Hz), 121.5, 119.2 (q, *J* = 5.7 Hz), 118.8, 83.3, 30.9, 29.6, 28.1; **^{19}F NMR** (376 MHz, $CDCl_3$): δ -68.3; **IR** ν_{max}: 2984, 2940, 1741, 1701, 1594, 1353, 1272, 1243, 1159, 1101, 1061, 941, 915, 813, 747; **HRMS** (ESI): $[M+Na]^+$ calcd. for $C_{22}H_{29}N_2O_4F_3Na$: 465.1977, found: 465.1962.

N,N-(bis-Boc)-4-(but-3-en-1-yl)-5-(prop-1-en-2-yl)pyridin-2-amine, **16d**

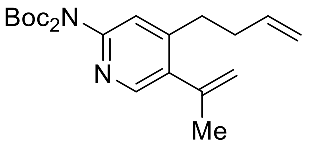

The compound was prepared in an analogous way to **16a**, using LDA (0.51 mL, 1.02 mmol), **15d** (295 mg, 0.85 mmol), allyl bromide (110 μL, 1.27 mmol) and THF (8 mL). After purification by flash column chromatography (petroleum ether/EtOAc, 19:1-9:1), **16d** was obtained as a colourless oil (198 mg, 0.51 mmol, 60%).

^{1}H NMR (500 MHz, $CDCl_3$): δ 8.20 (s, 1H), 7.05 (s, 1H), 5.80 (ddt, 1H, *J* = 17.0, 10.3, 6.6 Hz), 5.30 (app qn, 1H, *J* = 1.5 Hz), 5.03 (dq, 1H, *J* = 17.1, 1.6 Hz), 4.99 (dq, 1H, *J* = 10.2, 1.6 Hz), 4.93 (app q, 1H, *J* = 0.9 Hz), 2.75-2.72 (m, 2H), 2.37-2.32 (m, 2H), 2.04 (dd, 3H, *J* = 1.4, 1.0 Hz), 1.46 (s, 18H); **^{13}C NMR** (126 MHz, $CDCl_3$): δ 151.7, 151.1, 149.9, 147.9, 141.8, 138.4, 137.2, 121.8, 117.4, 115.7, 83.1, 34.3, 31.9, 28.1, 24.9; **IR** ν_{max}: 2979, 1795, 1759, 1727, 1595, 1479, 1369, 1342, 1306, 1274, 1251, 1154, 1116, 905, 854, 778; **HRMS** (ESI): $[M+H]^+$ calcd. for $C_{22}H_{33}N_2O_4$: 389.2440, found: 389.2437.

N,N-(bis-Boc)-4-(but-3-en-1-yl)-5-(1-phenylvinyl)pyridin-2-amine, **16e**

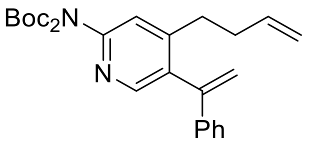

The compound was prepared in an analogous way to **16a**, using LDA (0.50 mL, 0.99 mmol), **15e** (326 mg, 0.79 mmol), allyl bromide (103 μL, 1.19 mmol) and THF (8 mL). After purification by flash column chromatography (petroleum ether/EtOAc, 19:1-9:1), **16e** was obtained as a colourless oil (245 mg, 0.54 mmol, 68%).

^{1}H NMR (400 MHz, $CDCl_3$): δ 8.31 (s, 1H), 7.30-7.27 (m, 3H), 7.24-7.22 (m, 2H), 7.10 (s, 1H), 5.84 (d, 1H, *J* = 1.0 Hz), 5.60 (ddt, 1H, *J* = 16.9, 10.3, 6.6 Hz), 5.29 (d, 1H, *J* = 1.0 Hz), 4.88-4.81 (m, 2H), 2.45-2.41 (m, 2H), 2.17-2.12 (m, 2H), 1.48 (s, 18H); **^{13}C NMR** (126 MHz, $CDCl_3$): δ 152.0, 151.6, 151.3, 149.4, 145.5, 140.1, 137.1, 136.1, 128.7, 128.3, 126.6, 121.9, 117.1, 115.6, 83.2, 33.6, 32.4, 28.1; **IR** ν_{max}: 2979, 1793, 1757, 1725, 1594, 1479, 1368, 1340, 1299, 1272, 1249, 1151, 1112, 911, 852, 777, 712, 695; **HRMS** (ESI): $[M+H]^+$ calcd. for $C_{27}H_{35}N_2O_4$: 451.2597, found: 451.2603.

5-Allyl-*N,N*-(bis-Boc)-4-(but-3-en-1-yl)pyridin-2-amine, **16f**

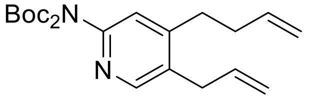

The compound was prepared in an analogous way to **16a**, using LDA (0.52 mL, 1.03 mmol), **15f** (300 mg, 0.86 mmol), allyl bromide (112 μL, 1.29 mmol) and THF (8 mL). After purification by flash column chromatography (petroleum ether/EtOAc, 19:1-4:1), **16f** was obtained as a white solid (161 mg, 0.41 mmol, 48%).

^{1}H NMR (500 MHz, $CDCl_3$): δ 8.20 (s, 1H), 7.02 (s, 1H), 5.95 (ddt, 1H, *J* = 17.0, 10.2, 6.0 Hz), 5.82 (ddt, 1H, *J* = 17.0, 10.3, 6.6 Hz), 5.09 (dq, 1H, *J* = 10.2, 1.5 Hz), 5.05 (dq, 1H, *J* = 17.1, 1.6 Hz), 5.01 (dq, 1H, *J* = 10.2, 1.4 Hz), 4.94 (dq, 1H, *J* = 17.1, 1.7 Hz), 3.40 (d, 2H, *J* = 6.0 Hz), 2.72-2.69 (m, 2H), 2.37-2.32 (m, 2H), 1.43 (s, 18H); **^{13}C NMR** (126 MHz, $CDCl_3$): δ 151.6, 151.3, 151.0, 149.7, 137.1, 136.0, 132.3, 122.0, 116.7, 115.9, 83.0, 34.0, 33.6, 31.4, 28.1; **IR** ν_{max}: 2981, 2933, 1762, 1717, 1601, 1480, 1393, 1367, 1340, 1304, 1237, 1149, 1111, 1050, 910, 848, 806, 771, 726; **HRMS** (ESI): $[M+H]^+$ calcd. for $C_{22}H_{33}N_2O_4$: 389.2440, found: 389.2450.

5-Allyl-4-(but-3-en-1-yl)-2-chloropyridine, **16g**

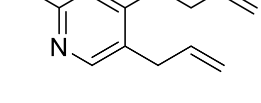

n-Butyllithium (1.5 M in hexanes, 9.95 mL, 14.93 mmol) was added dropwise to a solution of *i*-Pr_2NH (2.09 mL, 14.93 mmol) in THF (10 mL) at -78 °C. After stirring for 1 h, the LDA solution formed was added to a solution of **15g** (1.93 g, 11.49 mmol) in THF (20 mL) at -78 °C. After stirring for a further 1 h, allyl bromide (1.49 mL, 17.23 mmol) was added dropwise and the reaction stirred for 2 h. After warming to rt, the reaction was quenched with saturated aqueous NH_4Cl, the layers were separated

and the aqueous phase extracted with Et_2O (3x). The combined organic fractions were washed with brine, dried over $MgSO_4$ and evaporated. The residue was purified by flash column chromatography (hexane/Et_2O, 99:1-19:1) to provide **16g** as a yellow oil (1.88 g, 9.05 mmol, 79%).

^{1}H NMR (400 MHz, $CDCl_3$): δ 8.10 (s, 1H), 7.11 (s, 1H), 5.89 (ddt, 1H, *J* = 17.2, 10.0, 6.0 Hz), 5.80 (ddt, 1H, *J* = 16.8, 10.0, 6.4 Hz), 5.10 (dq, 1H, *J* = 10.0, 1.6 Hz), 5.04 (dq, 1H, *J* = 16.8, 1.6 Hz), 5.02 (dq, 1H, *J* = 10.0, 1.6 Hz), 4.95 (dq, 1H, *J* = 17.2, 1.6 Hz), 3.35 (dt, 2H, *J* = 6.0, 1.6 Hz), 2.68-2.64 (m, 2H), 2.35-2.29 (m, 2H); **^{13}C NMR** (101 MHz, $CDCl_3$): δ 152.4, 150.3, 149.6, 136.6, 135.4, 132.3, 123.9, 116.9, 116.0, 33.7, 33.3, 31.2; **IR** ν_{max}: 2980, 2915, 1639, 1585, 1548, 1466, 1439, 1355, 1151, 1086, 994, 910, 867; **HRMS** (ESI): $[M+H]^+$ calcd. for $C_{12}H_{15}NCl$: 208.0888, found: 208.0895.

N,N-(bis-Boc)-5,6-dihydroisoquinolin-3-amine, **17a**

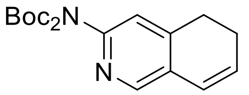

Grubbs catalyst, 2nd generation (179 mg, 0.211 mmol) was added to a solution of **16a** (1.578 g, 4.21 mmol) in CH_2Cl_2 (42 mL) and the mixture heated to reflux for 4 h. After cooling, 1-2 drops of DMSO were added then the solvent was evaporated. The residue was purified by flash column chromatography (petroleum ether/EtOAc, 19:1-6:1) to provide **17a** as a dark green oil (1.40 g, 4.04 mmol, 96%).

^{1}H NMR (400 MHz, $CDCl_3$): δ 8.09 (s, 1H), 6.97 (s, 1H), 6.49 (dt, 1H, *J* = 9.5, 1.7 Hz), 6.12 (dt, 1H, *J* = 9.6, 4.3 Hz), 2.81 (t, 2H, *J* = 8.3 Hz), 2.35 (tdd, 2H, *J* = 8.5, 4.4, 1.7 Hz), 1.46 (s, 18H); **^{13}C NMR** (126 MHz, $CDCl_3$): δ 151.8, 150.8, 146.4, 145.1, 130.7, 129.1, 123.9, 120.7, 83.2, 28.1, 26.9, 22.3; **IR** ν_{max}: 2979, 2934, 1793, 1754, 1722, 1598, 1485, 1368, 1338, 1295, 1241, 1149, 1111, 1045, 1032, 1012, 853, 807, 775, 711; **HRMS** (ESI): $[M+H]^+$ calcd. for $C_{19}H_{27}N_2O_4$: 347.1971, found: 347.1976.

N,N-(bis-Boc)-7-methyl-5,6-dihydroisoquinolin-3-amine, **17b**

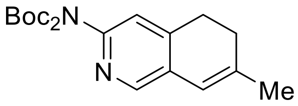

The compound was prepared in an analogous way to **17a**, using **16b** (201 mg, 0.517 mmol), Grubbs II (22 mg, 0.026 mmol) and CH_2Cl_2 (8.0 mL). After purification by flash column chromatography (petroleum ether/EtOAc, 9:1-17:3), **17b** was obtained as an off-white solid (175 mg, 0.49 mmol, 94%).

^{1}H NMR (500 MHz, $CDCl_3$): δ 8.03 (s, 1H), 6.92 (s, 1H), 6.23 (s, 1H), 2.82 (t, 2H, *J* = 8.4 Hz), 2.26 (t, 2H, *J* = 8.3 Hz), 1.92 (s, 3H), 1.45 (s, 18H); **^{13}C NMR** (126 MHz, $CDCl_3$): δ 151.8, 150.0, 145.0, 144.3, 140.6, 130.1, 120.5, 118.8, 83.0, 28.1, 27.9, 27.4, 23.7; **IR** ν_{max}: 2979, 2931, 1790, 1754, 1721, 1597, 1484, 1392, 1367, 1338, 1311, 1291, 1243, 1151, 1135, 1110, 1046, 1034, 851, 774, 729; **HRMS** (ESI): $[M+H]^+$ calcd. for $C_{20}H_{29}N_2O_4$: 361.2127, found: 361.2110.

N,N-(bis-Boc)-7-trifluoromethyl-5,6-dihydroisoquinolin-3-amine, **17c**

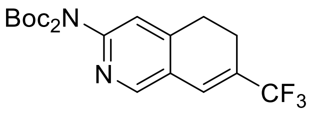

The compound was prepared in an analogous way to **17a**, using **16c** (295 mg, 0.667 mmol), Grubbs II (28 mg, 0.033 mmol) and CH_2Cl_2 (7 mL). After purification by flash column chromatography (petroleum ether/EtOAc, 19:1-9:1), **17c** was obtained as a colourless oil (148 mg, 0.357 mmol, 54%).

^{1}H NMR (500 MHz, $CDCl_3$): δ 8.23 (s, 1H), 7.13 (s, 1H), 6.99 (s, 1H), 2.95 (t, 2H, *J* = 8.3 Hz), 2.51 (t, 2H, *J* = 8.3 Hz), 1.48 (s, 18H); **^{13}C NMR** (126 MHz, $CDCl_3$): δ 152.8, 151.5, 146.9, 146.4, 129.4 (q, *J* = 31.7 Hz), 126.1, 125.1 (q, *J* = 6.1 Hz), 123.7 (q, *J* = 270.9 Hz), 120.0, 83.6, 28.1, 26.7, 20.1; **^{19}F NMR** (376 MHz, $CDCl_3$): δ -69.2; **IR** ν_{max}: 2982, 2933, 1795, 1764, 1729, 1603, 1487, 1395, 1369, 1330, 1311, 1294, 1258, 1238, 1158, 1111, 1042, 967, 858; **HRMS** (ESI): $[M+Na]^+$ calcd. for $C_{20}H_{25}N_2O_4F_3Na$: 437.1664, found: 437.1651.

N,N-(bis-Boc)-8-methyl-5,6-dihydroisoquinolin-3-amine, **17d**

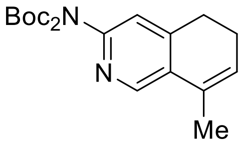

The compound was prepared in an analogous way to **17a**, using **16d** (192 mg, 0.494 mmol), Grubbs II (21 mg, 0.025 mmol) and CH_2Cl_2 (8.0 mL). After purification by flash column chromatography (petroleum ether/EtOAc, 19:1-9:1), **17d** was obtained as a colourless oil (167 mg, 0.46 mmol, 94%).

^{1}H NMR (500 MHz, $CDCl_3$): δ 8.27 (s, 1H), 7.00 (s, 1H), 5.90-5.88 (m, 1H), 2.77 (t, 2H, *J* = 8.1 Hz), 2.32-2.27 (m, 2H), 2.08 (q, 3H, *J* = 1.7 Hz), 1.47 (s, 18H); **^{13}C NMR** (126 MHz, $CDCl_3$): δ 151.8, 150.7, 147.4, 142.6, 130.6, 129.8, 127.1, 120.3, 83.2, 28.1, 27.7, 22.5, 18.8; **IR** ν_{max}: 2978, 2936, 1754, 1724, 1597, 1488, 1391, 1367, 1340, 1298, 1243, 1150, 1110, 1068, 1031, 854, 820, 775; **HRMS** (ESI): $[M+H]^+$ calcd. for $C_{20}H_{29}N_2O_4$: 361.2127, found: 361.2119.

N,N-(bis-Boc)-8-phenyl-5,6-dihydroisoquinolin-3-amine, **17e**

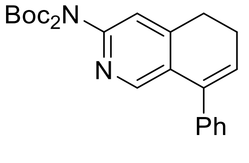

The compound was prepared in an analogous way to **17a**, using **16e** (215 mg, 0.477 mmol), Grubbs II (20 mg, 0.024 mmol) and CH_2Cl_2 (9.0 mL). After purification by flash column chromatography (toluene/EtOAc, 19:1), **17e** was obtained as a colourless wax (168 mg, 0.40 mmol, 83%).

^{1}H NMR (400 MHz, $CDCl_3$): δ 8.07 (s, 1H), 7.41-7.31 (m, 5H), 7.11 (s, 1H), 6.13 (t, 1H, *J* = 4.7 Hz), 2.87 (t, 2H, *J* = 8.0 Hz), 2.47-2.42 (m, 2H), 1.48 (s, 18H); **^{13}C NMR** (101 MHz, $CDCl_3$): δ 151.8, 150.7, 147.8, 144.9, 139.2, 137.2, 129.8, 129.0, 128.6, 128.6, 127.7, 120.1, 83.2, 28.1, 27.7, 22.8; **IR** ν_{max}: 2978, 2935,

1756, 1725, 1597, 1483, 1392, 1368, 1340, 1298, 1258, 1151, 1113, 1048, 853, 755, 702; **HRMS** (ESI): $[M+H]^+$ calcd. for $C_{25}H_{31}N_2O_4$: 423.2278, found: 423.2290.

N,N-(bis-Boc)-6,9-dihydro-5*H*-cyclohepta[*c*]pyridin-3-amine, **17f**

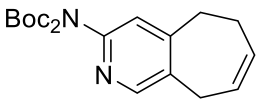

The compound was prepared in an analogous way to **17a**, using **16f** (125 mg, 0.322 mmol), Grubbs II (14 mg, 0.016 mmol) and CH_2Cl_2 (5.0 mL). After purification by flash column chromatography (toluene/EtOAc, 19:1-9:1), **17f** was obtained as a colourless oil (76 mg, 0.21 mmol, 65%).

^{1}H NMR (500 MHz, $CDCl_3$): δ 8.14 (s, 1H), 7.01 (s, 1H), 5.72-5.68 (m, 1H), 5.55-5.52 (m, 1H), 3.44-3.43 (m, 2H), 2.99 (t, 2H, *J* = 6.3 Hz), 2.36-2.32 (m, 2H), 1.45 (s, 18H); **^{13}C NMR** (126 MHz, $CDCl_3$): δ 152.9, 151.8, 151.1, 147.0, 136.4, 129.6, 124.8, 121.3, 83.1, 32.2, 29.9, 28.4, 28.1; **IR** ν_{max}: 2978, 2934, 1787, 1753, 1724, 1603, 1485, 1392, 1368, 1341, 1301, 1273, 1246, 1153, 1113, 1048, 854, 775; **HRMS** (ESI): $[M+Na]^+$ calcd. for $C_{20}H_{28}N_2O_4Na$: 383.1941, found: 383.1947.

3-Chloro-6,9-dihydro-5*H*-cyclohepta[*c*]pyridine, **17g**

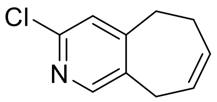

The compound was prepared in an analogous way to **17a**, using **16g** (100 mg, 0.48 mmol), Grubbs II (20 mg, 0.024 mmol) and CH_2Cl_2 (4.0 mL). After purification by flash column chromatography (petroleum ether/EtOAc, 19:1-9:1), **17g** was obtained as a pale pink solid (79 mg, 0.44 mmol, 91%).

^{1}H NMR (400 MHz, $CDCl_3$): δ 8.03 (s, 1H), 7.11 (s, 1H), 5.68 (dtt, 1H, *J* = 11.6, 5.2, 2.4 Hz), 5.52 (dtt, 1H, *J* = 11.6, 4.0, 2.0 Hz), 3.39 (dd, 2H, *J* = 5.2, 2.0 Hz), 2.95 (t, 2H, *J* = 6.4 Hz), 2.36-2.30 (m, 2H); **^{13}C NMR** (101 MHz, $CDCl_3$): δ 154.0, 149.5, 147.5, 136.4, 129.4, 124.5, 123.3, 31.9, 29.4, 28.1; **IR** ν_{max}: 2915, 2890, 1583, 1554, 1467, 1455, 1428, 1401, 1367, 1311, 1284, 1220, 1140, 1064, 961, 930, 880, 862, 800, 755, 660; **HRMS** (ESI): $[M+H]^+$ calcd. for $C_{10}H_{11}NCl$: 180.0575, found: 180.0569.

5,6-Dihydro-4*H*-pyrrolo[1,2-*b*]pyrazol-2-amine, **19a**

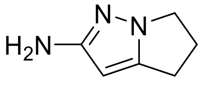

Palladium on activated carbon (5 wt% Pd, 14 mg, 0.006 mmol) was added to a solution of **10a** (10 mg, 0.066 mmol) in EtOH (1 mL). A H_2 atmosphere was applied and the reaction stirred at rt for 3 h. The mixture was filtered through a small pad of Celite and the filtrate evaporated. The residue was purified by flash column chromatography ($CHCl_3$/MeOH, 20:1) to provide **19a** as a yellow solid (5 mg, 0.041 mmol, 62%).

^{1}H NMR (400 MHz, $CDCl_3$): δ 5.33 (s, 1H), 3.95 (t, 2H, *J* = 7.1 Hz), 3.08 (br s, 2H), 2.79 (t, 2H, *J* = 7.3 Hz), 2.47 (app qn, 2H, *J* = 7.2 Hz); **^{13}C NMR** (126 MHz, $CDCl_3$): δ 158.3, 146.9, 86.1, 47.4, 25.2, 23.6; **IR** ν_{max}:

3437, 3284, 3176, 2946, 2893, 1629, 1545, 1479, 1467, 1440, 1415, 1323, 1298, 1092, 991, 738, 671; **HRMS** (ESI): $[M+H]^+$ calcd. for $C_6H_{10}N_3$: 124.0869, found: 124.0868.

4,5,6,7-Tetrahydropyrazolo[1,5-*a*]pyridin-2-amine, **19b**

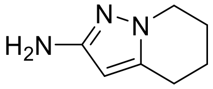

Palladium on activated carbon (10 wt% Pd, 16 mg, 0.015 mmol) was added to a solution of **10b** (51 mg, 0.309 mmol) in EtOH (1 mL) and THF (1 mL). A H_2 atmosphere was applied and the reaction stirred for 40 min. The mixture was filtered through a small pad of Celite and the filtrate evaporated. The residue was purified by flash column chromatography (petroleum ether/EtOAc, 1:2-0:1) to provide **19b** as a yellow oil (35 mg, 0.255 mmol, 83%).

^{1}H NMR (500 MHz, $CDCl_3$): δ 5.33 (s, 1H), 3.92 (t, 2H, *J* = 6.1 Hz), 2.96 (br s, 2H), 2.67 (t, 2H, *J* = 6.4 Hz), 2.00-1.96 (m, 2H), 1.81-1.77 (m, 2H); **^{13}C NMR** (126 MHz, $CDCl_3$): δ 153.7, 140.6, 90.0, 46.9, 23.5, 22.7, 20.5; **IR** ν_{max}: 3326, 3211, 2940, 2862, 1614, 1552, 1510, 1483, 1345, 1242, 1162, 944, 746, 668; **HRMS** (ESI): $[M+H]^+$ calcd. for $C_7H_{12}N_3$: 138.1026, found: 138.1024.

5,6,7,8-tetrahydro-4*H*-pyrazolo[1,5-*a*]azepin-2-amine, **19c**

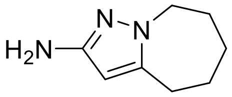

The compound was prepared in an analogous way to **19b**, using **10c** (30 mg, 0.167 mmol), Pd/C (10 wt% Pd, 8.8 mg, 0.008 mmol), EtOH (0.6 mL) and THF (0.6 mL). After purification by flash column chromatography (EtOAc), **19c** was obtained as a white solid (20 mg, 0.132 mmol, 79%).

^{1}H NMR (500 MHz, $CDCl_3$): δ 5.38 (s, 1H), 4.04-4.02 (m, 2H), 3.46 (br s, 2H), 2.63-2.61 (m, 2H), 1.82-1.77 (m, 2H), 1.74-1.70 (m, 2H), 1.67-1.62 (m, 2H); **^{13}C NMR** (126 MHz, $CDCl_3$): δ 151.9, 145.6, 92.9, 52.3, 30.9, 28.3, 27.0, 26.6; **IR** ν_{max}: 3431, 3297, 3183, 2930, 2849, 1621, 1556, 1483, 1450, 1437, 1410, 1361, 1355, 1269, 1079, 1007, 972, 864, 823, 756, 682, 667; **HRMS** (ESI): $[M+H]^+$ calcd. for $C_8H_{13}N_3$: 152.1182, found: 152.1183.

5,6,8,9-Tetrahydro-4*H*-pyrazolo[1,5-*d*][1,4]oxazocin-2-amine, **19d**

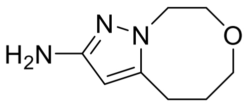

The compound was prepared in an analogous way to **19b**, using **10d** (5 mg, 0.027 mmol), Pd/C (5 wt% Pd, 3 mg, 0.001 mmol), EtOH (0.3 mL) and THF (0.3 mL). After purification by flash column chromatography (EtOAc/MeOH, 1:0-200:15), **19d** was obtained as a white solid (3 mg, 0.0179 mmol, 66%).

^{1}H NMR (500 MHz, $CDCl_3$): δ 5.44 (s, 1H), 4.10-4.08 (m, 2H), 3.78-3.76 (m, 2H), 3.47 (t, 2H, *J* = 5.6 Hz), 2.74 (t, 2H, *J* = 6.5 Hz), 1.86-1.82 (m, 2H); **^{13}C NMR** (126 MHz, $CDCl_3$): δ 153.4, 144.2, 92.6, 72.2, 69.9,

50.7, 32.4, 22.0; **IR** ν_{max}: 3422, 3271, 3194, 2920, 2904, 2857, 1619, 1557, 1489, 1453, 1420, 1264, 1196, 1097, 1050, 979, 907, 813, 748, 680, 667; **HRMS** (ESI): $[M+H]^+$ calcd. for $C_8H_{14}N_3O$: 168.1131, found: 168.1134.

4-Methyl-4,5,6,7-tetrahydropyrazolo[1,5-*a*]pyridin-2-amine, **19f**

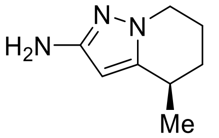

The compound was prepared in an analogous way to **19b**, using **10f** (29 mg, 0.16 mmol), Pd/C (5 wt% Pd, 17 mg, 0.008 mmol), EtOH (0.5 mL) and THF (0.5 mL). After purification by flash column chromatography (petroleum ether/EtOAc, 1:2-0:1), **19f** was obtained as a brown oil (20 mg, 0.132 mmol, 83%).

^{1}H NMR (500 MHz, $CDCl_3$): δ 5.39 (d, 1H, *J* = 0.8 Hz), 4.01-3.96 (m, 1H), 3.85-3.80 (m, 1H), 2.83 (br s, 2H), 2.83-2.76 (m, 1H), 2.09-2.03 (m, 1H), 1.97-1.88 (m, 2H), 1.41-1.33 (m, 1H), 1.24 (d, 3H, *J* = 7.0 Hz); **^{13}C NMR** (126 MHz, $CDCl_3$): δ 153.7, 146.4, 89.2, 46.9, 29.3, 29.0, 22.4, 20.3; **IR** ν_{max}: 3298, 3204, 2962, 2861, 1621, 1551, 1487, 1445, 1349, 1333, 1243, 1117, 956, 815, 748, 693, 667; **HRMS** (ESI): $[M+H]^+$ calcd. for $C_8H_{14}N_3$: 152.1182, found: 152.1179.

6-Nitro-1-tosyl-1a,2,3,7b-tetrahydro-1*H*-azirino[2,3-*c*]pyrazolo[1,5-*a*]pyridine, **20**

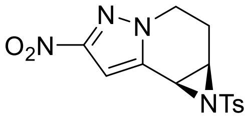

NBS (35 mg, 0.200 mmol) was added to a solution of **10b** (30 mg, 0.181 mmol), *p*-toluenesulfonamide (34 mg, 0.200 mmol), K_2CO_3 (52 mg, 0.381 mmol) and $Rh_2(cap)_4$ (3.6 mg, 0.0055 mmol) in CH_2Cl_2 (0.5 mL) and the reaction mixture stirred at rt for 19 h. The reaction was poured onto saturated aqueous NH_4Cl, the layers separated and the aqueous phase extracted with $CHCl_3$ (3x). The combined organic fractions were evaporated. The residue was purified by flash column chromatography (petroleum ether/EtOAc, 5:1-1:1) to provide **20** as a white solid (21 mg, 0.063 mmol, 35%).

^{1}H NMR (400 MHz, $CDCl_3$): δ 7.81 (d, 2H, *J* = 8.2 Hz), 7.37 (d, 2H, *J* = 8.1 Hz), 6.94 (s, 1H), 4.32 (dd, 1H, *J* = 13.5, 6.0 Hz), 4.03-3.95 (m, 2H), 3.68 (d, 1H, *J* = 6.9 Hz), 2.52 (br d, 1H, *J* = 14.4 Hz), 2.46 (s, 3H), 2.24-2.16 (m, 1H); **^{13}C NMR** (126 MHz, $CDCl_3$): δ 154.6, 145.5, 136.0, 134.2, 130.1, 127.8, 102.5, 44.4, 38.1, 34.0, 21.7, 21.5; **IR** ν_{max}: 3145, 2936, 1596, 1530, 1478, 1340, 1327, 1225, 1186, 1164, 1091, 987, 825, 816, 757, 706, 695, 674, 659; **HRMS** (ESI): $[M+H]^+$ calcd. for $C_{14}H_{15}N_4O_4S$: 335.0809, found: 335.0796.

trans-4,5-Dibromo-2-nitro-4,5,6,7-tetrahydropyrazolo[1,5-*a*]pyridine, **21**

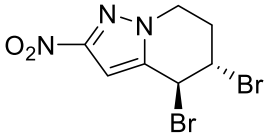

Br_2 (15 μL, 0.291 mmol) was added to a solution of **10b** (40 mg, 0.242 mmol) in $CHCl_3$ (0.8 mL) and stirred at rt for 18 h. The solvent was evaporated and the residue purified by flash column chromatography (petroleum ether/EtOAc, 5:1) to provide **21** as a white solid (76 mg, 0.235 mmol, 97%).

^{1}H NMR (400 MHz, $CDCl_3$): δ 6.95 (s, 1H), 5.59 (t, 1H, *J* = 1.9 Hz), 4.81-4.79 (m, 1H), 4.55-4.52 (m, 2H), 3.17-3.08 (m, 1H), 2.47-2.41 (m, 1H); **^{13}C NMR** (126 MHz, $CDCl_3$): δ 155.8, 139.3, 103.0, 45.3, 45.1, 39.1, 25.7; **IR** ν_{max}: 6127, 3006, 1526, 1476, 1408, 1343, 1315, 1279, 1227, 1112, 1005, 924, 930, 769, 759, 721; **HRMS** (ESI): $[M+H]^+$ calcd. for $C_7H_8Br_2N_3O_2$: 323.8983, found: 323.8986.

2-Nitro-6,7-dihydropyrazolo[1,5-*a*]pyridin-6-ol, **22**

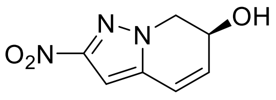

SeO_2 (120 mg, 1.09 mmol) was added to a solution of **10b** (30 mg, 0.182 mmol) in 1,4-dioxane (1 mL) and the reaction mixture heated to 80 °C for 20 h. After cooling,the mixture was filtered through Celite and the filtrate evaporated. The residue was purified by flash column chromatography (petroleum ether/EtOAc, 3:2-1:1) to provide **22** as a yellow solid (9 mg, 0.050 mmol, 27%).

^{1}H NMR (500 MHz, acetone-d_6): δ 6.87 (s, 1H), 6.67 (d, 1H, *J* = 10.0 Hz), 6.40 (dd, 1H, *J* = 9.9, 3.9 Hz), 4.77-4.74 (m, 2H), 4.36-4.35 (m, 2H); **^{13}C NMR** (126 MHz, acetone-d_6): δ 156.4, 139.6, 131.9, 117.7, 99.4, 63.3, 54.7; **IR** ν_{max}: 3433, 3115, 2923, 2545, 1529, 1469, 1407, 1365, 1322, 1293, 1223, 1080, 1054, 1003, 933, 891, 849, 828, 776, 757, 680; **HRMS** (ESI): $[M+H]^+$ calcd. for $C_7H_8N_3O_3$:182.0566, found: 182.0560.

cis-2-Nitro-4,5,6,7-tetrahydropyrazolo[1,5-*a*]pyridine-4,5-diol, **23**

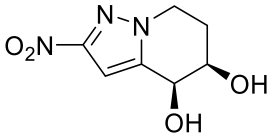

Osmium tetroxide (2.5 wt% in *t*-BuOH, 18 μL, 0.0018 mmol) was added to a solution of **10b** (30 mg, 0.182 mmol), NMO (43 mg, 0.364 mmol) and citric acid (70 mg, 0.364 mmol) in THF (0.5 mL) and water (0.5 mL) and the reaction mixture stirred at rt for 16 h. The reaction was diluted with saturated aqueous Na_2SO_3 (1.5 mL) and poured onto brine, the layers separated and the aqueous phase extracted with 2-butanone (3x). The combined organic fractions were dried over $MgSO_4$ and evaporated. The residue was purified by flash column chromatography (petroleum ether/EtOAc, 1:2-0:1) then triturated by EtOAc/Et_2O to provide **23** as a white solid (14 mg, 0.070 mmol, 39%).

^{1}H NMR (400 MHz, CD_3OD): δ 6.91 (s, 1H), 4.75 (d, 1H, *J* = 3.5 Hz), 4.33-4.26 (m, 1H), 4.20-4.14 (m, 2H), 2.40-2.32 (m, 1H), 2.20-2.12 (m, 1H); **^{13}C NMR** (101 MHz, CD_3OD): δ 156.8, 147.0, 101.9, 66.8, 66.8, 45.9, 27.5; **IR** ν_{max}: 3537, 3381, 3142, 2977, 1537, 1484, 1407, 1393, 1337, 1219, 1123, 1104, 1070, 1059, 1009, 976, 874, 826, 787, 758, 666; **HRMS** (ESI): $[M+H]^+$ calcd. for $C_7H_{10}N_3O_4$: 200.0671, found: 200.0674.

7,7-Difluoro-2-nitro-6,6a,7,7a-tetrahydro-5*H*-cyclopropa[*c*]pyrazolo[1,5-*a*]pyridine, **24**

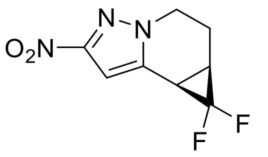

$TMSCF_3$ (67 μL, 0.455 mmol) was added to a solution of anhydrous NaI (59 mg, 0.364 mmol) and **10b** (30 mg, 0.182 mmol) in acetonitrile (540 μL) and the reaction mixture heated in a sealed tube to 110 °C for 17 h. After cooling, the mixture was poured onto water, the layers separated and the aqueous phase extracted with EtOAc (2x). The combined organic fractions were washed with brine, dried over $MgSO_4$ and evaporated. The residue was purified by flash column chromatography (petroleum ether/EtOAc, 4:1) to provide **24** as a white solid (6 mg, 0.028 mmol, 15%).

^{1}H NMR (500 MHz, $CDCl_3$): δ 6.90 (s, 1H), 4.40-4.34 (m, 1H), 4.05-3.99 (m, 1H), 2.94 (t, 1H, 10.5 Hz), 2.50-2.44 (m, 1H), 2.38-2.27 (m, 2H); **^{13}C NMR** (126 MHz, $CDCl_3$): δ 154.9, 134.3, 111.9 (t, *J* = 293.2 Hz), 101.9, 45.2 (d, *J* = 6.7 Hz), 19.8 (t, *J* = 11.0 Hz), 19.2 (dd, *J* = 15.6, 11.7 Hz), 17.1; **^{19}F NMR** (376 MHz, $CDCl_3$): δ -122.2, (d, *J* = 160.4 Hz), -145.5 (d, *J* = 160.2 Hz); **IR** ν_{max}: 3044, 1557, 1532, 1481, 1458, 1443, 1410, 1341, 1283, 1211, 1185, 1142, 1101, 1016, 1002, 966, 915, 856, 831, 810, 755, 726, 695; **HRMS** (ESI): $[M+H]^+$ calcd. for $C_8H_8FN_3O_2$: 216.0585, found: 216.0587.

trans-5-Bromo-4-hydroxy-2-nitro-4,5,6,7-tetrahydropyrazolo[1,5-*a*]pyridine, **25**

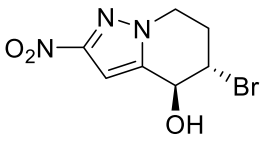

NBS (65 mg, 0.363 mmol) was added to a solution of **10b** (50 mg, 0.303 mmol) in THF (0.8 mL) and water (0.2 mL) and stirred at rt for 23 h. The reaction was diluted with water, the layers separated and the aqueous phase extracted with $CHCl_3$ (2x). The combined organic fractions were evaporated. The residue was purified by flash column chromatography (petroleum ether/EtOAc, 5:1-3:1) to provide **25** as a brown solid (27 mg, 0.103 mmol, 34%).

^{1}H NMR (500 MHz, CD_3OD): δ 6.96 (s, 1H), 4.97 (d, 1H, *J* = 4.6 Hz), 4.44-4.41 (m, 2H), 4.33 (app t, 1H, *J* = 5.5 Hz), 2.88-2.82 (m, 1H), 2.48-2.42 (m, 1H); **^{13}C NMR** (126 MHz, CD_3OD): δ 156.8, 144.6, 102.5, 68.6, 49.5, 47.5, 28.2; **IR** ν_{max}: 3448, 3297, 2970, 1541, 1489, 1405, 1330, 1281, 1223, 1191, 1067, 1013, 925, 826, 757; **HRMS** (ESI): $[M+H]^+$ calcd. for $C_7H_9BrN_3O_3$: 261.9827, found: 261.9837.

6-Nitro-1a,2,3,7b-tetrahydrooxireno[2,3-*c*]pyrazolo[1,5-*a*]pyridine, **26**

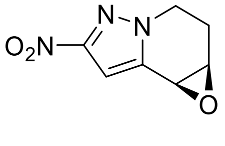

m-CPBA (136 mg, 0.606 mmol) was added to a suspension of **10b** (50 mg, 0.303 mmol) and $NaHCO_3$ (76 mg, 0.909 mmol) in CH_2Cl_2 (2 mL) at 0 °C and the reaction mixture stirred for 2 h. The reaction was incomplete so further $NaHCO_3$ (76 mg, 0.909 mmol) and *m*-CPBA (136 mg, 0.606 mmol) were added and the reaction stirred for a further 1.5 h. The reaction was quenched with 10% (w/v) aqueous

$Na_2S_2O_3$ then poured onto 1 M aqueous NaOH. The layers were separated and the aqueous phase extracted with $CHCl_3$ (3x). The combined organic fractions were washed with brine, dried over $MgSO_4$ and evaporated. The residue was purified by column chromatography (petroleum ether/EtOAc, 4:1-3:1) to provide **26** as a white solid (19 mg, 0.105 mmol, 35%).

^{1}H NMR (500 MHz, $CDCl_3$): δ 7.07 (s, 1H), 4.34 (dd, 1H, *J* = 13.4, 6.3 Hz), 4.06-3.98 (m, 2H), 3.85 (t, 1H, *J* = 3.4 Hz), 2.66 (dt, 1H, *J* = 15.1, 3.7 Hz), 2.34-2.25 (m, 1H); **^{13}C NMR** (126 MHz, $CDCl_3$): δ 154.6, 138.4, 103.2, 52.4, 44.7, 44.1, 23.1; **IR** ν_{max}: 3127, 1523, 1479, 1455, 1413, 1406, 1355, 1296, 1252, 1232, 1107, 1045, 1004, 930, 892, 854, 841, 830, 759, 739; **HRMS** (ESI): $[M+H]^+$ calcd for $C_7H_8N_3O_3$: 182.0566, found: 182.0562.

2-Nitro-4,5,6,7-tetrahydropyrazolo[1,5-*a*]pyridin-4-ol, **27**

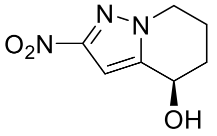

Borane THF complex (1.0 M in THF, 12.1 mL, 12.1 mmol) was added to a solution of **10b** (400 mg, 2.42 mmol) in THF (4 mL) at 0 °C and the reaction mixture stirred at rt for 20 min. The solution was then re-cooled to 0 °C and 3 M aqueous NaOH solution (4 mL) was carefully added, followed by H_2O_2 (30 wt% in H_2O, 4 mL). The reaction was then allowed to warm to rt and stirred for 2 h. A solution of 3 M aqueous HCl (2 mL) was added and the resulting mixture was poured onto brine, the layers separated and the aqueous phase extracted with $CHCl_3$ (2x). The combined organic fractions were dried over $MgSO_4$ and evaporated. The residue was purified by flash column chromatography (petroleum ether/EtOAc, 2:1-3:2) to provide **27** as a white solid (324 mg, 1.77 mmol, 73%).

^{1}H NMR (500 MHz, $CDCl_3$): δ 6.90 (d, 1H, *J* = 0.6 Hz), 4.96 (app q, 1H, *J* = 5.3 Hz), 4.28-4.23 (m, 1H), 4.20-4.15 (m, 1H), 2.40-2.33 (m, 1H), 2.21-2.15 (m, 1H), 2.10-2.02 (m, 2H), 1.99-1.93 (m, 1H); **^{13}C NMR** (126 MHz, $CDCl_3$): δ 155.5, 144.8, 100.6, 62.6, 48.9, 29.4, 19.0; **IR** ν_{max}: 3383, 3143, 2957, 1540, 1529, 1453, 1396, 1327, 1278, 1245, 1112, 1070, 996, 954, 879, 823, 758; **HRMS** (ESI): $[M+H]^+$ calcd. for $C_7H_{10}N_3O_3$: 184.0717, found: 184.0710.

2-Nitro-5,6,7,8-tetrahydro-4*H*-pyrazolo[1,5-*a*]azepin-4-ol, **28**

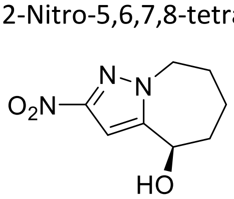

The compound was prepared in an analogous way to **27**, using **10c** (28 mg, 0.156 mmol), $BH_3 \cdot THF$ (0.78 mL, 0.78 mmol), THF (1 mL), NaOH (0.25 mL) and H_2O_2 (0.25 mL). After purification by flash column chromatography (petroleum ether/EtOAc, 2:1-3:2), **28** was obtained as a brown solid (24 mg, 0.122 mmol, 78%).

^{1}H NMR (500 MHz, $CDCl_3$): δ 6.82 (s, 1H), 4.97 (br d, 1H, *J* = 8.1 Hz), 4.52 (ddd, 1H, *J* = 14.2, 9.0, 1.6 Hz), 4.33-4.28 (m, 1H), 2.24-2.19 (m, 1H), 2.09-2.04 (m, 2H), 1.91-1.79 (m, 4H); **^{13}C NMR** (126 MHz, $CDCl_3$): δ 153.7, 148.6, 101.5, 66.0, 54.9, 34.4, 27.6, 24.1; **HRMS** (ESI): $[M+H]^+$ calcd. for $C_8H_{12}N_3O_3$: 198.0873, found: 198.0869.

2-Amino-4,5,6,7-tetrahydropyrazolo[1,5-*a*]pyridin-4-ol, **29**

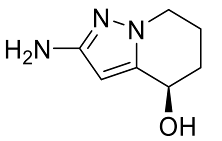

The compound was prepared in an analogous way to **19a**, using **27** (33 mg, 0.18 mmol), Pd/C (5 wt% Pd, 38 mg, 0.018 mmol) and EtOH (1 mL). After purification by flash column chromatography ($CHCl_3$/MeOH, 1:0-10:1), **29** was obtained as a brown solid (23 mg, 0.150 mmol, 84%).

^{1}H NMR (500 MHz, $CDCl_3$): δ 5.60 (s, 1H), 4.80 (t, 1H, *J* = 5.4 Hz), 3.97-3.92 (m, 1H), 3.89-3.84 (m, 1H), 2.27 (br s, 2H), 2.27-2.19 (m, 1H), 2.06-2.00 (m, 1H), 1.95-1.88 (m, 1H), 1.87-1.81 (m, 1H); **^{13}C NMR** (126 MHz, $CDCl_3$): δ 153.9, 143.2, 90.4, 62.9, 46.8, 29.9, 19.3; **IR** ν_{max}: 3350, 2930, 2855, 1621, 1557, 1485, 1444, 1365, 1342, 1064, 997, 891, 772; **HRMS** (ESI): $[M+H]^+$ calcd. for $C_7H_{12}N_3O$: 154.0975, found: 154.0973.

4-Bromo-2-nitro-4,5,6,7-tetrahydropyrazolo[1,5-*a*]pyridine, **30**

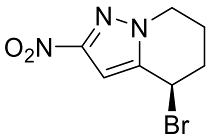

PBr_3 (27 μL, 0.284 mmol) was added to a solution of **27** (52 mg, 0.284 mmol) in CH_2Cl_2 (1 mL) at 0 °C and the resulting solution heated to 50 °C for 50 min. After cooling, saturated aqueous $NaHCO_3$ was added, the layers separated and the aqueous phase extracted with $CHCl_3$ (3x). The combined organic fractions were evaporated. The residue was purified by flash column chromatography (petroleum ether/EtOAc, 5:1) to provide **30** as a white solid (56 mg, 0.229 mmol, 80%).

^{1}H NMR (500 MHz, $CDCl_3$): δ 6.89 (s, 1H), 5.40 (t, 1H, *J* = 4.3 Hz), 4.43 (ddd, 1H, *J* = 13.5, 5.4, 4.0 Hz), 4.19 (ddd, 1H, *J* = 13.6, 10.5, 5.2 Hz), 2.60-2.51 (m, 1H), 2.44-2.30 (m, 2H), 2.21-2.14 (m, 1H); **^{13}C NMR** (126 MHz, $CDCl_3$): δ 155.4, 142.5, 102.0, 48.9, 36.8, 30.4, 19.6; **HRMS** (ESI): $[M+H]^+$ calcd. for $C_7H_9BrN_3O_2$: 245.9878, found: 245.9887.

4-Fluoro-2-nitro-4,5,6,7-tetrahydropyrazolo[1,5-*a*]pyridine, **31**

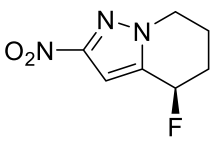

DAST (43 μL, 0.328 mmol) was added to a solution of **27** (30 mg, 0.164 mmol) in CH_2Cl_2 (0.6 mL) at -78 °C and the resulting solution stirred at rt for 30 min. Saturated aqueous $NaHCO_3$ was added, the layers separated and the aqueous phase extracted with $CHCl_3$ (2x). The combined organic fractions were evaporated. The residue was purified by flash column chromatography (petroleum ether/EtOAc, 4:1) to provide **31** as a white solid (25 mg, 0.135 mmol, 82%).

^{1}H NMR (400 MHz, $CDCl_3$): δ 7.00 (d, 1H, *J* = 2.6 Hz), 5.67 (dt, 1H, *J* = 51.1, 3.8 Hz), 4.41 (dt, 1H, *J* = 13.7, 4.6 Hz), 4.16-4.08 (m, 1H), 2.46-2.32 (m, 2H), 2.17-1.99 (m, 2H); **^{13}C NMR** (101 MHz, $CDCl_3$): δ 155.5,

139.4 (d, J = 23.8 Hz), 102.5 (d, J = 2.8 Hz), 80.2 (d, J = 172.3 Hz), 49.0, 26.8 (d, J = 22.0 Hz), 18.0 (d, J = 2.3 Hz); **^{19}F NMR** (376 MHz, $CDCl_3$): δ -164.0; **IR** ν_{max}: 3153, 2969, 2921, 1530, 1482, 1461, 1409, 1344, 1326, 1281, 1254, 1209, 1109, 1064, 1006, 985, 946, 927, 877, 832, 818, 757, 701, 665; **HRMS** (ESI): $[M+H]^+$ calcd. for $C_7H_9FN_3O_2$: 186.0679, found: 168.0677.

4-Azido-2-nitro-4,5,6,7-tetrahydropyrazolo[1,5-*a*]pyridine, **32**

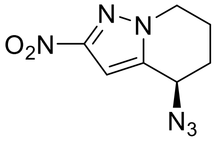

DPPA (117 μL, 0.546 mmol) was added to a suspension of **27** (50 mg, 0.273 mmol) and DBU (77 μL, 0.546 mmol) in toluene (1 mL) at 0 °C and the resulting solution stirred at rt for 30 min. Saturated aqueous NH_4Cl was added, the layers separated and the aqueous phase extracted with $CHCl_3$ (3x). The combined organic fractions were evaporated. The residue was purified by flash column chromatography (petroleum ether/EtOAc, 4:1-3:2) to provide **32** as a yellow solid (24 mg, 0.115 mmol, 42%).

^{1}H NMR (500 MHz, $CDCl_3$): δ 6.92 (d, 1H, J = 0.5 Hz), 4.75 (t, 1H, J = 5.1 Hz), 4.34-4.29 (m, 1H), 4.20-4.15 (m, 1H), 2.38-2.30 (m, 1H), 2.18-2.01 (m, 3H); **^{13}C NMR** (126 MHz, $CDCl_3$): δ 155.5, 139.8, 101.0, 52.9, 48.9, 26.3, 19.1; **IR** ν_{max}: 3143, 2977, 2115, 2091, 1537, 1524, 1487, 1459, 1404, 1331, 1279, 1266, 1255, 1235, 1204, 1114, 1006, 949, 915, 878, 832, 817, 756, 708, 668; **HRMS** (ESI): $[M+H]^+$ calcd. for $C_7H_9N_6O_2$: 209.0787, found: 209.0790.

4,5,6,7-Tetrahydropyrazolo[1,5-*a*]pyridine-2,4-diamine, **33**

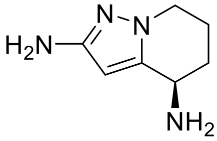

The compound was prepared in an analogous way to **19b**, using **32** (22 mg, 0.106 mmol), Pd/C (5 wt % Pd, 22 mg, 0.011 mmol), EtOH (1 mL) and THF (0.5 mL). After purification by flash column chromatography ($CHCl_3$/MeOH, 1:0-6:1), **33** was obtained as a brown oil (11 mg, 0.072 mmol, 68%).

^{1}H NMR (500 MHz, CD_3OD): δ 5.64 (s, 1H), 3.93-3.86 (m, 2H), 3.82-3.76 (m,1H), 2.18-2.07 (m, 2H), 1.98-1.90 (m, 1H), 1.59-1.52 (m, 1H); **^{13}C NMR** (126 MHz, CD_3OD): δ 155.9, 146.7, 91.2, 47.6, 46.6, 31.2, 21.9; **HRMS** (ESI): $[M+H]^+$ calcd. for $C_7H_{13}N_4$: 153.1135, found: 153.1130.

2-Amino-5,6,7,8-tetrahydro-4*H*-pyrazolo[1,5-*a*]azepin-4-ol, **34**

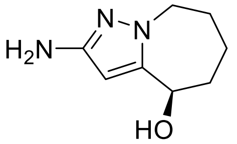

The compound was prepared in an analogous way to **19a**, using **28** (22 mg, 0.112 mmol), Pd/C (5 wt% Pd, 23 mg, 0.011 mmol) and EtOH (1 mL). After purification by flash column chromatography ($CHCl_3$/MeOH, 1:0-10:1), **34** was obtained as a colourless oil (17 mg, 0.102 mmol, 91%).

^{1}H NMR (500 MHz, $CDCl_3$): δ 5.54 (s, 1H), 4.80 (dd, 1H, *J* = 8.1, 2.0 Hz), 4.17 (ddd, 1H, *J* = 14.2, 9.0, 1.8 Hz), 4.00-3.96 (m, 1H), 2.63 (br s, 3H), 2.13-2.06 (m, 1H), 2.00-1.93 (m, 1H), 1.84-1.67 (m, 4H); **^{13}C NMR** (126 MHz, $CDCl_3$): δ 151.8, 147.2, 92.6, 66.5, 52.4, 34.6, 28.2, 24.5; **IR** ν_{max}: 3350, 3243, 3139, 2925, 1559, 1505, 1484, 1366, 1296, 1120, 1056, 964, 925, 881, 773; **HRMS** (ESI): $[M+H]^+$ calcd. for $C_8H_{14}N_3O$: 168.1131, found: 168.1129.

trans-4-Fluoro-2-nitro-4,5,6,7-tetrahydropyrazolo[1,5-*a*]pyridin-5-ol, **35**

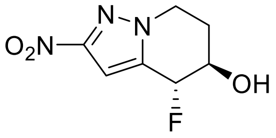

HF-Pyridine (75 μL, 2.88 mmol) was added to a solution of **26** (17.3 mg, 0.096 mmol) in CH_2Cl_2 (0.5 mL) at 0 °C and the reaction mixture stirred for 20 min. Saturated aqueous $NaHCO_3$ was then added, the layers separated and the aqueous phase extracted with $CHCl_3$ (3x). The combined organic fractions were evaporated. The residue was purified by flash column chromatography (petroleum ether/EtOAc, 2:1-3:2) to provide **35** as a white solid (6 mg, 0.030 mmol, 31%).

^{1}H NMR (500 MHz, acetone-d$_6$): δ 7.15 (d, 1H, *J* = 2.4 Hz), 5.55 (dd, 1H, *J* = 50.5, 4.5 Hz), 4.97 (d, 1H, *J* = 4.1 Hz), 4.43-4.34 (m, 3H), 2.47-2.40 (m, 1H), 2.34-2.28 (m, 1H); **^{13}C NMR** (126 MHz, acetone-d$_6$): δ 156.4, 139.9 (d, *J* = 23.4 Hz), 104.0 (d, *J* = 1.9 Hz), 84.6 (d, *J* = 174.0 Hz), 65.5 (d, *J* = 24.7 Hz), 45.9, 26.5; **^{19}F NMR** (376 MHz, acetone-d$_6$): δ -170.3; **IR** ν_{max}: 3392, 3165, 2927, 2513, 1551, 1535, 1485, 1410, 1325, 1296, 1283, 1269, 1111, 1097, 1003, 979, 938, 832, 813, 756; **HRMS** (ESI): $[M+H]^+$ calcd. for $C_7H_9FN_3O_3$: 202.0628, found: 202.0636.

2-Nitro-4,5,6,7-tetrahydropyrazolo[1,5-*a*]pyridin-5-ol, **36**

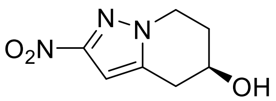

Zinc iodide (49 mg, 0.157 mmol) and sodium cyanoborohydride (50 mg, 0.794 mmol) were added to a solution of **26** (18.9 mg, 0.104 mmol) in 1,2-dichloroethane (1 mL) and the reaction mixture heated to reflux for 2 h. The reaction was incomplete so, after cooling, further zinc iodide (49 mg, 0.157 mmol) and sodium cyanoborohydride (50 mg, 0.794 mmol) were added and the reaction heated to reflux for a further 3 h. After cooling, the reaction was poured onto water, the layers separated and the aqueous phase extracted with $CHCl_3$ (3x). The combined organic fractions were washed with brine, dried over $MgSO_4$ and evaporated. The residue was purified by flash column chromatography (petroleum ether/EtOAc, 3:1-1:1) to provide **36** as a white solid (5 mg, 0.027 mmol, 26%).

^{1}H NMR (500 MHz, CD_3OD): δ 6.71 (s, 1H), 4.35-4.28 (m, 2H), 4.22 (dt, 1H, *J* = 13.2, 5.7 Hz), 3.07 (dd, 1H, *J* = 16.9, 4.4 Hz), 2.87 (dd, 1H, *J* = 16.9, 5.2 Hz), 2.26-2.15 (m, 2H); **^{13}C NMR** (126 MHz, CD_3OD): δ 156.7, 142.7, 101.3, 63.1, 46.2, 32.0, 30.8; **IR** ν_{max}: 3474, 3140, 2954, 2572, 1537, 1527, 1481, 1401,

1338, 1317, 1276, 1214, 1156, 1102, 1070, 1008, 972, 838, 828, 811, 760; **HRMS** (ESI): $[M+H]^+$ calcd. for $C_7H_{10}N_3O_3$: 184.0722, found: 184.0726.

N,N-(bis-Boc)-5,6,7,8-tetrahydroisoquinolin-3-amine, **53a**

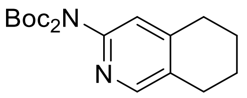

Palladium on activated carbon (10 wt% Pd, 17 mg, 0.015 mmol) was added to a solution of **17a** (53 mg, 0.153 mmol) in EtOAc (1.5 mL). A H_2 atmosphere was applied and the reaction stirred for 4 h. The mixture was filtered through a small pad of Celite and the filtrate evaporated. The residue was purified by flash column chromatography (petroleum ether/EtOAc, 9:1-4:1) to provide **53a** as a white solid (48 mg, 0.138 mmol, 90%).

^{1}H NMR (500 MHz, $CDCl_3$): δ 8.18 (s, 1H), 6.90 (s, 1H), 2.77-2.73 (m, 4H), 1.82-1.79 (m, 4H), 1.46 (s, 18H); **^{13}C NMR** (126 MHz, $CDCl_3$): δ 152.0, 149.6, 149.4, 148.5, 132.3, 122.1, 83.0, 28.9, 28.1, 26.1, 22.6, 22.3; **IR** ν_{max}: 2977, 2932, 1737, 1702, 1604, 1357, 1274, 1246, 1162, 1120, 1047, 1032, 988, 984, 858, 814, 769, 749; **HRMS** (ESI): $[M+H]^+$ calcd. for $C_{19}H_{29}N_2O_4$: 349.2122, found: 349.2117.

N,N-(bis-Boc)-7-methyl-5,6,7,8-tetrahydroisoquinolin-3-amine, **53b**

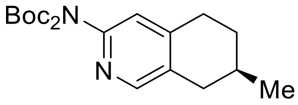

The compound was prepared in an analogous way to **53a**, using **17b** (167 mg, 0.463 mmol), Pd/C (10 wt% Pd, 49 mg, 0.046 mmol) and EtOAc (4.5 mL). After purification by flash column chromatography (petroleum ether/EtOAc, 19:1-17:3), **53b** was obtained as a white solid (141 mg, 0.39 mmol, 84%).

^{1}H NMR (500 MHz, $CDCl_3$): δ 8.17 (s, 1H), 6.91 (s, 1H), 2.85-2.79 (m, 3H), 2.33 (dd, 1H, *J* = 16.4, 10.6 Hz), 1.91-1.81 (m, 2H), 1.46 (s, 18H), 1.42-1.35 (m, 1H), 1.08 (d, 3H, *J* = 6.6 Hz); **^{13}C NMR** (126 MHz, $CDCl_3$): δ 152.1, 149.7, 149.3, 148.1, 132.1, 121.8, 83.0, 34.6, 30.5, 28.9, 28.7, 28.1, 21.8; **IR** ν_{max}: 2978, 2943, 2867, 1744, 1708, 1602, 1478, 1393, 1366, 1342, 1269, 1243, 1154, 1112, 1048, 853, 814, 742; **HRMS** (ESI): $[M+H]^+$ calcd. for $C_{20}H_{31}N_2O_4$: 363.2278, found: 363.2268.

N,N-(bis-Boc)-7-trifluoromethyl-5,6,7,8-tetrahydroisoquinolin-3-amine, **53c**

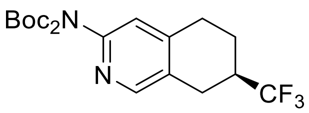

The compound was prepared in an analogous way to **53a**, using **17c** (140 mg, 0.338 mmol), Pd/C (10 wt% Pd, 36 mg, 0.034 mmol) and EtOAc (3.5 mL). After purification by flash column chromatography (petroleum ether/EtOAc, 19:1-9:1), **53c** was obtained as a white solid (122 mg, 0.293 mmol, 87%).

^{1}H NMR (500 MHz, $CDCl_3$): δ 8.26 (s, 1H), 6.99 (s, 1H), 3.07-3.03 (m, 1H), 2.98-2.93 (m, 1H), 2.87-2.77 (m, 2H), 2.52-2.44 (m, 1H), 2.23-2.18 (m, 1H), 1.76-1.68 (m, 1H), 1.47 (s, 18H); **^{13}C NMR** (126 MHz, $CDCl_3$): δ 151.9, 150.4, 149.5, 146.7, 128.8, 127.5 (q, *J* = 278.4 Hz), 121.6, 83.3, 38.9 (q, *J* = 27.6 Hz),

28.1, 27.7, 25.2 (q, J = 2.8 Hz), 21.4 (q, J = 2.4 Hz); **^{19}F NMR** (376 MHz, $CDCl_3$): δ -74.6; **IR** ν_{max}: 2980, 2936, 1793, 1754, 1726, 1605, 1480, 1393, 1369, 1345, 1308, 1267, 1250, 1165, 1115, 1047, 860; **HRMS** (ESI): $[M+H]^+$ calcd. for $C_{20}H_{28}N_2O_4F_3$: 417.1996, found: 417.1994.

N,N-(bis-Boc)-8-methyl-5,6,7,8-tetrahydroisoquinolin-3-amine, **53d**

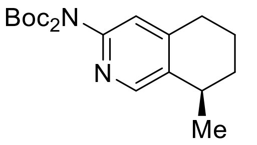

The compound was prepared in an analogous way to **53a**, using **17d** (155 mg, 0.43 mmol), Pd/C (10 wt% Pd, 46 mg, 0.043 mmol) and EtOAc (4.3 mL). After purification by flash column chromatography (petroleum ether/EtOAc, 19:1-17:3), **53d** was obtained as a colourless oil (122 mg, 0.337 mmol, 78%).

^{1}H NMR (500 MHz, $CDCl_3$): δ 8.30 (s, 1H), 6.90 (s, 1H), 2.97-2.91 (m, 1H), 2.80-2.69 (m, 2H), 1.95-1.82 (m, 2H), 1.76-1.69 (m, 1H), 1.59-1.52 (m, 1H), 1.47 (s, 18H), 1.31 (d, 3H, J = 7.1 Hz); **^{13}C NMR** (126 MHz, $CDCl_3$): δ 152.1, 149.4, 148.9, 148.2, 137.1, 121.7, 83.1, 31.0, 30.1, 29.4, 28.1, 22.5, 19.6; **IR** ν_{max}: 2977, 2934, 1753, 1725, 1601, 1479, 1593, 1368, 1344, 1318, 1247, 1152, 1113, 1048, 858, 806, 774; **HRMS** (ESI): $[M+H]^+$ calcd. for $C_{20}H_{31}N_2O_4$: 363.2278, found: 363.2273.

N,N-(bis-Boc)-8-phenyl-5,6,7,8-tetrahydroisoquinolin-3-amine, **53e**

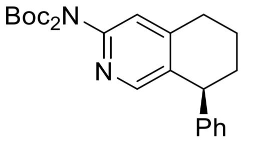

The compound was prepared in an analogous way to **53a**, using **17e** (141 mg, 0.334 mmol), Pd/C (10 wt% Pd, 35 mg, 0.033 mmol) and EtOAc (3 mL). After purification by flash column chromatography (petroleum ether/EtOAc, 19:1-9:1), **53e** was obtained as a colourless oil (99 mg, 0.23 mmol, 70%).

^{1}H NMR (400 MHz, $CDCl_3$): δ 7.96 (s, 1H), 7.29-7.25 (m, 2H), 7.22-7.19 (m, 1H), 7.06-7.04 (m, 2H), 7.01 (s, 1H), 4.14 (t, 1H, J = 6.4 Hz), 2.91 (dt, 1H, J = 17.7, 6.7 Hz), 2.83 (dt, 1H, J = 17.6, 6.2 Hz), 2.21-2.13 (m, 1H), 1.94-1.82 (m, 2H), 1.80-1.70 (m, 1H), 1.47 (s, 18H); **^{13}C NMR** (101 MHz, $CDCl_3$): δ 151.9, 150.8, 149.8, 148.9, 146.0, 134.4, 128.7, 128.6, 126.5, 121.4, 83.1, 42.7, 32.7, 29.3, 28.1, 19.9; **IR** ν_{max}: 2979, 2932, 1791, 1755, 1724, 1601, 1478, 1393, 1368, 1343, 1298, 1273, 1248, 1152, 1114, 858, 755, 702; **HRMS** (ESI): $[M+H]^+$ calcd. for $C_{25}H_{33}N_2O_4$: 425.2440, found: 425.2426.

N,N-(bis-Boc)-6,7,8,9-tetrahydro-5*H*-cyclohepta[*c*]pyridin-3-amine, **53f**

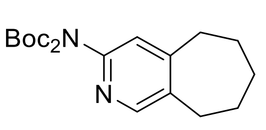

The compound was prepared in an analogous way to **53a**, using **17f** (22mg, 0.061 mmol), Pd/C (5 wt% Pd, 13 mg, 0.006 mmol) and EtOAc (1 mL). After purification by flash column chromatography (petroleum ether/EtOAc, 9:1-17:3), **53f** was obtained as a colourless oil (20 mg, 0.055 mmol, 90%).

^{1}H NMR (400 MHz, $CDCl_3$): δ 8.19 (s, 1H), 6.97 (s, 1H), 2.80-2.78 (m, 4H), 1.86 (app qn, 2H, *J* = 5.7 Hz), 1.66-1.61 (m, 4H), 1.45 (s, 18H); **^{13}C NMR** (101 MHz, $CDCl_3$): δ 155.4, 151.6, 150.1, 147.4, 138.3, 122.4, 83.3, 36.4, 32.9, 32.6, 28.1, 27.9, 27.5; **IR** ν_{max}: 2979, 2925, 2852, 1789, 1754, 1725, 1601, 1485, 1393, 1368, 1342, 1305, 1248, 1154, 1113, 1053, 852; **HRMS** (ESI): $[M+H]^+$ calcd. for $C_{20}H_{31}N_2O_4$: 363.2278, found: 363.2280.

5,6,7,8-tetrahydroisoquinolin-3-amine hydrochloride, **37a**

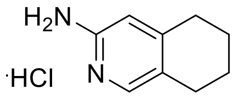

HCl (2 M in Et_2O, 5 mL, 10.0 mmol) was added to a solution of **53a** (95 mg, 0.273 mmol) in Et_2O (1 mL) and MeOH (0.5 mL) and the resulting mixture heated to reflux for 16 h. After cooling, the solvent was evaporated and the resulting solid dried *in vacuo* to provide **37a** as a yellow solid (50 mg, 0.271 mmol, 99%).

^{1}H NMR (400 MHz, DMSO-d_6): δ 13.68 (br s, 1H), 7.72 (s, 1H), 7.66 (br s, 2H), 6.69 (s, 1H), 2.75 (t, 2H, *J* = 5.8 Hz), 2.58 (t, 2H, *J* = 5.8 Hz), 1.70-1.67 (m, 4H); **^{13}C NMR** (101 MHz, DMSO-d_6): δ 156.5, 152.0, 133.4, 122.3, 111.0, 28.8, 24.4, 21.6, 21.2; **IR** ν_{max}: 3425, 3274, 3106, 2942, 1668, 1614, 1473, 1427, 1322, 1204, 1151, 893, 836, 812; **HRMS** (ESI): $[M+H]^+$ calcd. for $C_9H_{13}N_2$: 149.1073, found: 149.1077.

7-Methyl-5,6,7,8-tetrahydroisoquinolin-3-amine hydrochloride, **37b**

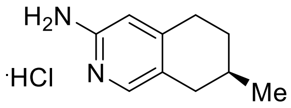

HCl (2 M in Et_2O, 6.6 mL, 13.2 mmol) was added to a solution of **53b** (120 mg, 0.331 mmol) in Et_2O (1.5 mL) and the resulting mixture stirred at rt for 16 h. The solid formed was collected under suction, washed on the filter with Et_2O and dried *in vacuo* to provide **37b** as a pale yellow solid (60 mg, 0.30 mmol, 91%).

^{1}H NMR (500 MHz, DMSO-d_6): δ 13.62 (br s, 1H), 7.71 (s, 1H), 7.62 (br s, 2H), 6.69 (s, 1H), 2.88-2.83 (m, 1H), 2.79-2.70 (m, 2H), 2.14 (dd, 1H, *J* = 16.0, 10.8 Hz), 1.82-1.71 (m, 2H), 1.29 (dtd, 1H, *J* = 12.9, 10.9, 5.7 Hz), 1.00 (d, 3H, *J* = 6.6 Hz); **^{13}C NMR** (126 MHz, DMSO-d_6): δ 156.1, 152.0, 133.5, 122.2, 110.8, 32.8, 29.4, 28.6, 27.9, 21.3; **IR** ν_{max}: 3354, 3293, 3244, 3151, 2926, 2865, 2653, 1663, 1624, 1479, 1209, 1185, 1056, 914, 850; **HRMS** (ESI): $[M+H]^+$ calcd. for $C_{10}H_{15}N_2$: 163.1230, found: 163.1225.

7-Trifluoromethyl-5,6,7,8-tetrahydroisoquinolin-3-amine hydrochloride, **37c**

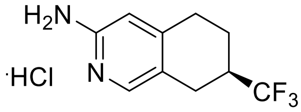

The compound was prepared in an analogous way to **37a**, using HCl (5.5 mL, 11.1 mmol), **53c** (116 mg, 0.279 mmol), Et_2O (1.5 mL) and MeOH (1 mL). **37c** was obtained as a white solid (59 mg, 0.234 mmol, 84%).

^{1}H NMR (400 MHz, DMSO-d$_6$): δ 13.90 (br s, 1H), 7.85 (s, 1H), 7.78 (br s, 2H), 6.75 (s, 1H), 2.97-2.90 (m, 2H), 2.88-2.71 (m, 2H), 2.58 (dd, 1H, *J* = 15.6, 11.3 Hz), 2.06-2.02 (m, 1H), 1.66-1.56 (m, 1H); **^{13}C NMR** (101 MHz, DMSO-d$_6$): δ 155.0, 152.3, 134.1, 127.9 (q, *J* = 278.7 Hz), 119.4, 110.7, 36.7 (q, *J* = 26.7 Hz), 27.4, 23.4 (q, *J* = 2.9 Hz), 20.4 (q, *J* = 2.1 Hz); **^{19}F NMR** (376 MHz, DMSO-d$_6$): δ -72.9; **IR** ν_{max}: 3228, 3111, 2923, 2794, 1669, 1615, 1475, 1430, 1370, 1273, 1257, 1226, 1201, 1168, 1146, 1110, 1054, 1010, 925, 903, 848, 808, 743, 689; **HRMS** (ESI): $[M+H]^+$ calcd. for $C_{10}H_{12}N_2F_3$: 217.0947, found: 217.0943.

8-Methyl-5,6,7,8-tetrahydroisoquinolin-3-amine hydrochloride, **37d**

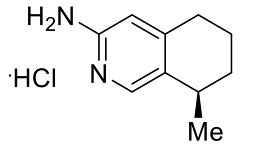

The compound was prepared in an analogous way to **37b**, using HCl (4.4 mL, 8.83 mmol), **53d** (80 mg, 0.221 mmol) and Et_2O (1.6 mL). **37d** was obtained as a pale yellow solid (28 mg, 0.141 mmol, 64%).

^{1}H NMR (500 MHz, DMSO-d$_6$): δ 13.65 (br s, 1H), 7.80 (s, 1H), 7.61 (br s, 2H), 6.67 (s, 1H), 2.80-2.73 (m, 3H), 1.87-1.73 (m, 2H), 1.68-1.60 (m, 1H), 1.41-1.34 (m, 1H), 1.21 (d, 3H, *J* = 6.9 Hz); **^{13}C NMR** (126 MHz, DMSO-d$_6$): δ 156.3, 151.9, 133.5, 127.5, 110.7, 30.2, 29.3, 28.9, 21.6, 19.1; **IR** ν_{max}: 3367, 3148, 2932, 2661, 1665, 1623, 1479, 1329, 1244, 1044, 1027, 944, 884, 868, 657; **HRMS** (ESI): $[M+H]^+$ calcd. for $C_{10}H_{15}N_2$: 163.1230, found: 163.1224.

8-Phenyl-5,6,7,8-tetrahydroisoquinolin-3-amine hydrochloride, **37e**

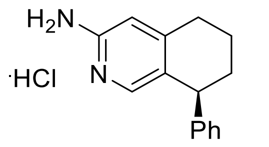

HCl (4 M in dioxane, 1.3 mL, 5.2 mmol) was added to a solution of **53e** in dioxane (1.3 mL) and the resulting mixture stirred at rt for 16 h. The solid formed was collected under suction, washed on the filter with Et_2O and dried *in vacuo* to provide **37e** as a white solid (24 mg, 0.092 mmol, 72%).

^{1}H NMR (400 MHz, DMSO-d$_6$): δ 13.53 (br s, 1H), 7.75 (br s, 2H), 7.34 (app t, 2H, *J* = 7.4 Hz), 7.27-7.23 (m, 1H), 7.22 (s, 1H), 7.16 (d, 2H, *J* = 7.3 Hz), 6.77 (s, 1H), 4.04 (dd, 1H, *J* = 7.8, 5.9 Hz), 2.95-2.81 (m, 2H), 2.06-1.99 (m, 1H), 1.84-1.63 (m, 3H); **^{13}C NMR** (101 MHz, DMSO-d$_6$): δ 156.5, 151.9, 145.1, 135.0, 128.6, 128.3, 126.6, 125.5, 110.9, 41.1, 31.4, 29.1, 19.4; **IR** ν_{max}: 3262, 3072, 2934, 1664, 1640, 1602, 1470, 1422, 1314, 1281, 1224, 1213, 1146, 825, 767, 753, 746, 714, 701, 666; **HRMS** (ESI): $[M+H]^+$ calcd. for $C_{15}H_{17}N_2$: 225.1386, found: 225.1381.

6,7,8,9-Tetrahydro-5*H*-cyclohepta[*c*]pyridin-3-amine hydrochloride, **37f**

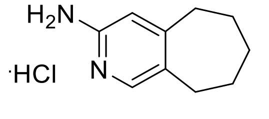

The compound was prepared in an analogous way to **37a**, using HCl (1 mL, 2.0 mmol), **53f** (18 mg, 0.050 mmol), Et_2O (0.5 mL) and MeOH (0.25 mL). **37f** was obtained as a white solid (9 mg, 0.046 mmol, 92%).

^{1}H NMR (400 MHz, DMSO-d_6): δ 13.49 (br s, 1H), 7.82 (br s, 2H), 7.70 (s, 1H), 6.75 (s, 1H), 2.77-2.74 (m, 2H), 2.65-2.63 (m, 2H), 1.74 (app qn, 2H, *J* = 5.6 Hz), 1.59-1.53 (m, 4H); **^{13}C NMR** (101 MHz, DMSO-d_6): δ 161.7, 152.7, 132.3, 127.9, 111.4, 35.7, 31.2, 30.7, 28.2, 27.2; **IR** ν_{max}: 3214, 3073, 2917, 2851, 1665, 1615, 1519, 1472, 1441, 1210, 1073, 945, 859, 831, 675; **HRMS** (ESI): $[M+H]^+$ calcd. for $C_{10}H_{15}N_2$: 163.1230, found: 163.1234.

N,N-(bis-Boc)-1-tosyl-1a,2,3,7b-tetrahydro-1H-azirino[2,3-h]isoquinolin-5-amine, **38**

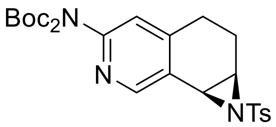

p-Toluenesulfonamide (27 mg, 0.159 mmol), K_2CO_3 (42 mg, 0.303 mmol) and $Rh_2(OAc)_4$ (0.1 mg, 0.14 μmol) were added to a solution of **17a** (50 mg, 0.144 mmol) in CH_2Cl_2. NBS (28 mg, 0.159 mmol) was then added and the reaction stirred at rt for 18 h. The mixture was diluted with water (10 mL) and CH_2Cl_2 (10 mL), the layers separated and the aqueous phase extracted with CH_2Cl_2 (2x). The combined organic fractions were dried over $MgSO_4$ and evaporated. The residue was purified by flash column chromatography (petroleum ether/EtOAc, 9:1-7:3) to provide **38** as an off-white solid (43 mg, 0.083 mmol, 58%).

^{1}H NMR (500 MHz, $CDCl_3$): δ 8.31 (s, 1H), 7.80 (d, 2H, *J* = 8.3 Hz), 7.31 (d, 2H, *J* = 8.2 Hz), 7.01 (s, 1H), 3.80 (d, 1H, *J* = 7.0 Hz), 3.65 (m, 1H), 2.76 (m, 1H), 2.57 (dd, 1H, *J* = 16.2, 5.2 Hz), 2.42 (s, 3H), 2.34 (m, 1H), 1.69 (m, 1H), 1.47 (s, 18H); **^{13}C NMR** (126 MHz, $CDCl_3$): δ 152.3, 151.7, 148.4, 147.9, 144.8, 135.3, 130.0, 127.8, 125.5, 120.8, 83.6, 41.1, 39.1, 28.1, 24.6, 21.8, 19.2; **IR** ν_{max}: 2979, 1754, 1722, 1609, 1368, 1324, 1297, 1242, 1155, 1113, 1091, 991, 952, 909, 874, 853, 809, 723, 675; **HRMS** (ESI): $[M+H]^+$ calcd. for $C_{26}H_{34}N_3O_6S$: 516.2163, found: 516.2165.

trans-7,8-dibromo-*N,N*-(bis-Boc)-5,6,7,8-tetrahydroisoquinolin-3-amine, **39**

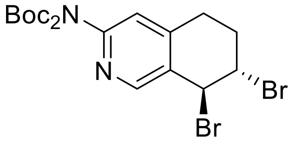

Br_2 (9 μL, 0.173 mmol) was added to a solution of **17a** (50 mg, 0.144 mmol) in $CHCl_3$ (0.5 mL) and stirred at rt for 3.5 h. The solvent was evaporated and the residue purified by flash column chromatography (petroleum ether/EtOAc, 9:1) to provide **39** as a white solid (61 mg, 0.120 mmol, 84%).

^{1}H NMR (400 MHz, $CDCl_3$): δ 8.39 (s, 1H), 7.12 (s, 1H), 5.61 (app s, 1H), 4.91-4.89 (m, 1H), 3.25 (ddd, 1H, *J* = 18.3, 11.9, 6.1 Hz), 2.96 (dd, 1H, *J* = 18.2, 6.0 Hz), 2.84-2.76 (m, 1H), 2.24-2.18 (m, 1H), 1.48 (s, 18H); **^{13}C NMR** (101 MHz, $CDCl_3$): δ 151.7, 151.5, 151.4, 145.7, 128.3, 120.3, 83.6, 50.1, 46.9, 28.0, 24.4, 24.3; **IR** ν_{max}: 2981, 1765, 1729, 1600, 1482, 1415, 1371, 1337, 1293, 1261, 1230, 1141, 1108, 1031, 845, 805, 766, 674; **HRMS** (ESI): $[M+H]^+$ calcd. for $C_{19}H_{27}Br_2N_2O_4$: 505.0338, found: 505.0357.

cis-3-(*N,N*-bis-Boc-amino)-5,6,7,8-tetrahydroisoquinoline-7,8-diol, **40**

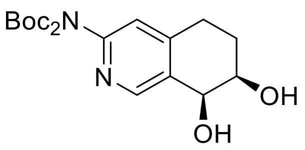

Osmium tetroxide (2.5 wt% in *t*-BuOH, 14 µL, 1.4 µmol) was added to a solution of **17a** (48 mg, 0.139 mmol), NMO (32 mg, 0.277 mmol) and citric acid (53 mg, 0.277 mmol) in THF (2.2 mL) and water (2.2 mL) and stirred at rt for 16 h. The reaction was diluted with saturated aqueous Na_2SO_3 (10 mL) and EtOAc (10 mL) and stirred vigorously for 5 min. The layers were separated and the organic phase washed with water (3x) and brine, then dried over $MgSO_4$ and evaporated. The residue was purified by flash column chromatography (petroleum ether/EtOAc, 3:1-1:9) to provide **40** as a colourless wax (32 mg, 0.084 mmol, 61%).

^{1}H NMR (500 MHz, $CDCl_3$): δ 8.49 (s, 1H), 7.02 (s, 1H), 4.74 (br s, 1H), 4.04-4.03 (m, 1H), 2.99 (dt, 1H, *J* = 18.0, 6.0 Hz), 2.81-2.72 (m, 2H), 2.45 (br d, 1H, *J* = 5.1 Hz), 2.11-2.03 (m, 1H), 1.93-1.87 (m, 1H), 1.48 (s, 18H); **^{13}C NMR** (126 MHz, $CDCl_3$): δ 151.9, 151.4, 150.5, 148.1, 131.7, 120.8, 83.5, 68.8, 67.6, 28.1, 26.1, 25.7; **IR** ν_{max}: 3362, 2980, 2934, 1726, 1605, 1479, 1457, 1368, 1272, 1245, 1151, 1106, 1066, 1047, 962, 850, 729; **HRMS** (ESI): $[M+H]^+$ calcd. for $C_{19}H_{29}N_2O_6$: 381.2020, found: 381.2013.

3-(*N,N*-bis-Boc-amino)-5,6,7,8-tetrahydroisoquinolin-8-ol, **41**

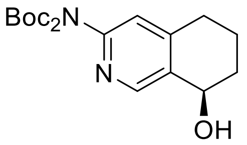

Borane dimethyl sulfide complex (0.41 mL, 4.33 mmol) was added dropwise to a solution of **17a** (150 mg, 0.433 mmol) in THF (4.5 mL) at 0 °C and the solution stirred for 30 min before being allowed to warm to rt and stirred for a further 1 h. After re-cooling to 0 °C, $NaHCO_3$ (18 mg, 0.217 mmol) was added in one portion, followed by careful dropwise addition of H_2O_2 (30 wt% in H_2O, 1.5 mL, 13.2 mmol). The mixture was allowed to warm to rt and stirred for 18 h. The reaction was diluted with brine and EtOAc, the layers separated and the aqueous phase extracted with EtOAc (2x). The combined organic fractions were dried over $MgSO_4$ and evaporated. The residue was purified by flash column chromatography (CH_2Cl_2/MeOH, 49:1) to provide **41** as a colourless wax (95 mg, 0.261 mmol, 60%).

^{1}H NMR (500 MHz, $CDCl_3$): δ 8.53 (s, 1H), 6.99 (s, 1H), 4.85 (app dd, 1H, *J* = 9.7, 5.8 Hz), 2.83 (dt, 1H, *J* = 17.6, 5.5 Hz), 2.72 (dt, 1H, *J* = 17.8, 6.6 Hz), 2.02-1.90 (m, 3H), 1.85 (d, 1H, *J* = 6.4 Hz), 1.82-1.76 (m, 1H), 1.47 (s, 18H); **^{13}C NMR** (126 MHz, $CDCl_3$): δ 151.9, 151.2, 149.6, 148.7, 133.8, 121.4, 83.3, 65.9, 32.1, 28.8, 28.1, 18.2; **IR** ν_{max}: 3362, 2979, 2937, 1785, 1750, 1725, 1603, 1478, 1457, 1393, 1367, 1343, 1247, 1152, 1113, 1047, 1005, 967, 853, 776, 735; **HRMS** (ESI): $[M+H]^+$ calcd. for $C_{19}H_{29}N_2O_5$: 365.2076, found: 365.2075.

7-bromo-3-(*N,N*-bis-Boc-amino)-6,7-dihydroisoquinolin-8(5H)-one, **42**

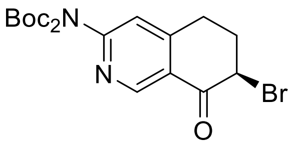

A solution of **17a** (60 mg, 0.173 mmol) in DMSO (0.6 mL) was added to a solution of IBX (45 wt%, 216 mg, 0.346 mmol) in DMSO (0.9 mL). NBS (34 mg, 0.190 mmol) was added and the reaction stirred at rt for 22 h. The reaction was poured onto saturated aqueous $NaHCO_3$ (10 mL) and diluted with CH_2Cl_2 (10 mL). The layers were separated and the aqueous phase extracted with CH_2Cl_2 (3x). The combined organic fractions were washed with brine, dried over $MgSO_4$ and evaporated. The residue was purified by flash column chromatography (CH_2Cl_2) to provide **42** as a white solid (40 mg, 0.091 mmol, 52%).

^{1}H NMR (400 MHz, $CDCl_3$): δ 9.02 (s, 1H), 7.46 (s, 1H), 4.70 (t, 1H, *J* = 4.1 Hz), 3.31 (ddd, 1H, *J* = 17.5, 10.0, 5.1 Hz), 2.92 (dt, 1H, *J* = 17.5, 4.1 Hz), 2.56-2.42 (m, 2H), 1.52 (s, 18H); **^{13}C NMR** (101 MHz, $CDCl_3$): δ 189.1, 155.4, 153.1, 151.0, 150.4, 123.1, 116.6, 84.2, 49.5, 31.0, 28.0, 25.9; **IR** ν_{max}: 2984, 2922, 2853, 1767, 1730, 1685, 1593, 1370, 1335, 1285, 1259, 1233, 1143, 1108, 1047, 1035, 1023, 855, 843, 803, 778, 753; **HRMS** (ESI): $[M+H]^+$ calcd. for $C_{19}H_{26}BrN_2O_5$: 441.1020, found: 441.1030.

trans-7-Bromo-3-(*N,N*-bis-Boc-amino)-5,6,7,8-tetrahydroisoquinolin-8-ol, **43**

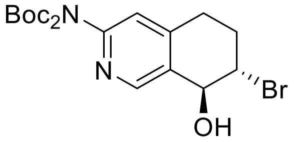

NBS (116 mg, 0.654 mmol) was added to a solution of **17a** (206 mg, 0.595 mmol) in THF (2.4 mL) and water (0.6 mL) and stirred at rt for 4.5 h. The reaction was diluted with water (10 mL) and Et_2O (10 mL), the layers were separated and the aqueous phase extracted with Et_2O (3x). The combined organic fractions were dried over $MgSO_4$ and evaporated. The residue was purified by flash column chromatography (petroleum ether/EtOAc, 3:1-1:1) to provide **43** as an off-white solid (199 mg, 0.449 mmol, 75%).

^{1}H NMR (500 MHz, $CDCl_3$): δ 8.57 (s, 1H), 7.04 (s, 1H), 4.94 (dd, 1H, *J* = 6.6, 4.7 Hz), 4.34 (ddd, 1H, *J* = 9.5, 6.7, 3.0 Hz), 2.99 (dt, 1H, *J* = 18.2, 3.0 Hz), 2.91 (dt, 1H, *J* = 18.0, 6.9 Hz), 2.76 (d, 1H, *J* = 4.6 Hz), 2.56-2.50 (m, 1H), 2.30-2.22 (m, 1H), 1.48 (s, 18H); **^{13}C NMR** (126 MHz, $CDCl_3$): δ 151.7, 151.6, 149.5, 146.6, 130.4, 120.5, 83.5, 72.0, 54.2, 28.6, 28.1, 27.5; **IR** ν_{max}: 3156, 2977, 1794, 1613, 1369, 1279, 1255, 1153, 1098, 1036, 998, 850, 820, 778, 683; **HRMS** (ESI): $[M+H]^+$ calcd. for $C_{19}H_{28}BrN_2O_5$: 443.1182, found: 443.1189.

tert-Butyl (8-Amino-7-hydroxy-5,6,7,8-tetrahydroisoquinolin-3-yl) carbamate, **44**

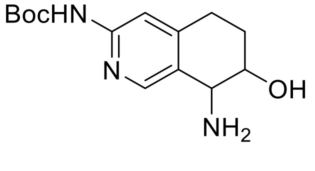

A suspension of **43** (75 mg, 0.169 mmol) in NH_3 (35% in H_2O) was stirred at rt in a stoppered flask for 72 h. The solvent was evaporated under a stream of nitrogen and the residue purified by flash column chromatography (CH_2Cl_2/MeOH, 9:1-4:1) to provide **44** as a pale brown solid (32 mg, 0.115 mmol, 68%). The compound exists as an inseparable 2:1 mixture of stereoisomers.

^{1}H NMR (500 MHz, DMSO-d$_6$): δ 9.60 (s, 0.5H), 9.56 (s, 1H), 8.30 (s, 1H), 8.26 (s, 0.5H), 7.52 (s, 0.5H), 7.49 (s, 1H), 5.04 (br s, 1H), 3.97 (d, 0.5H, *J* = 4.0 Hz), 3.88 (dt, 0.5H, *J* = 9.2, 3.5 Hz), 3.64 (d, 1H, *J* = 6.6 Hz), 3.58-3.55 (m, 1H), 2.87-2.76 (m, 1.5H), 2.73-2.63 (m, 1.5H), 2.00-1.94 (m, 1H), 1.92-1.84 (m, 0.5H), 1.77-1.71 (m, 0.5H), 1.69-1.61 (m, 1H), 1.45 (s, 13.5H); **^{13}C NMR** (126 MHz, DMSO-d$_6$): δ 152.8, 152.8, 151.0, 150.6, 149.0, 148.2, 147.4, 146.9, 128.8, 127.4, 110.7, 110.6, 79.5, 79.4, 71.0, 66.9, 53.9, 50.2, 28.1, 28.1, 27.2, 26.2, 26.2, 25.5; **HRMS** (ESI): $[M+H]^+$ calcd. for $C_{14}H_{22}N_3O_3$: 280.1656, found: 280.1659.

N,N-(bis-Boc)-7,8-epoxy-5,6,7,8-tetrahydroisoquinolin-3-amine, **45**

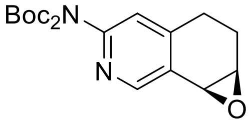

A solution of **43** (50 mg, 0.113 mmol) in MeOH (0.6 mL) was added dropwise to a solution of NaOMe (7 mg, 0.124 mmol) in MeOH (1 mL) at 0 °C and stirred for 30 min before being allowed to warm to rt and stirred for a further 30 min. Two further portions of NaOMe (7 mg, 0.124 mmol) were then added at 2 h intervals and the reaction stirred for 16 h. The solvent was evaporated and the residue purified by flash column chromatography (petroleum ether/EtOAc, 3:1) to provide **45** as a colourless oil (32 mg, 0.088 mmol, 78%).

^{1}H NMR (400 MHz, $CDCl_3$): δ 8.43 (s, 1H), 7.01 (s, 1H), 3.90 (d, 1H, *J* = 4.1 Hz), 3.78-3.76 (m, 1H), 2.77 (ddd, 1H, *J* = 15.5, 13.7, 6.8 Hz), 2.56 (dd, 1H, *J* = 15.9, 5.4 Hz), 2.47-2.41 (m, 1H), 1.76 (td, 1H, *J* = 13.9, 5.6 Hz), 1.45 (s, 18H); **^{13}C NMR** (101 MHz, $CDCl_3$): δ 152.5, 151.5, 148.6, 148.0, 127.9, 121.0, 83.3, 54.8, 49.8, 28.0, 24.1, 20.9; **IR** ν_{max}: 2980, 2935, 1790, 1755, 1722, 1607, 1489, 1368, 1337, 1317, 1296, 1245, 1150, 1112, 1046, 1032, 981, 849, 806, 776, 730; **HRMS** (ESI): $[M+Na]^+$ calcd. for $C_{19}H_{26}N_2O_5Na$: 385.1739, found: 385.1754.

3-Chloro-6,7,8,9-tetrahydro-5*H*-cyclohepta[*c*]pyridine, **46**

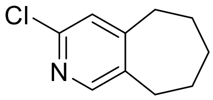

PtO_2 (7 mg, 0.03 mmol) was added to a solution of **17g** (50 mg, 0.28 mmol) in MeOH (2 mL). A H_2 atmosphere was applied and the reaction stirred at rt for 1 h. The mixture was filtered through a small pad of Celite and the filtrate evaporated. The residue was purified by flash column chromatography (hexane/Et_2O, 19:1) to provide **46** as a colourless oil (35 mg, 0.20 mmol, 69%).

^{1}H NMR (500 MHz, $CDCl_3$): δ 8.05 (s, 1H), 7.04 (s, 1H), 2.75-2.72 (m, 4H), 1.85 (qn, 2H, *J* = 5.5 Hz), 1.66-1.60 (m, 4H, m); **^{13}C NMR** (126 MHz, $CDCl_3$): δ 155.5, 149.2, 148.7, 137.7, 123.7, 36.0, 32.5, 32.4, 27.8, 27.2; **IR** ν_{max}: 2922, 2852, 1585, 1554, 1470, 1439, 1371, 1358, 1316, 1213, 1155, 1086, 961, 929, 915, 872, 835, 655; **HRMS** (ESI): $[M+H]^+$ calcd. for $C_{10}H_{13}NCl$: 182.0731, found: 182.0726.

trans-7,8-Dibromo-3-chloro-6,7,8,9-tetrahydro-5*H*-cyclohepta[*c*]pyridine, **47**

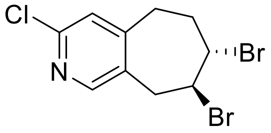

Br_2 (35 µL, 0.67 mmol) was added to a solution of **17g** (100 mg, 0.56 mmol) in CH_2Cl_2 (0.5 ml) and stirred for 16 h. The reaction was diluted with CH_2Cl_2 (2 mL) and H_2O (2 mL), the layers were separated and the aqueous phase extracted with CH_2Cl_2 (3x). The combined organic fractions were washed with brine, dried over $MgSO_4$ and evaporated. The residue was purified by flash column chromatography (hexane/Et_2O, 19:1-9:1) to provide **47** as a white solid (123 mg, 0.36 mmol, 65%).

^{1}H NMR (500 MHz, $CDCl_3$): δ 8.07 (s, 1H), 7.12 (s, 1H), 4.83 (app s, 1H), 4.76 (app s, 1H), 3.98 (d, 1H, *J* = 15.6 Hz), 3.35 (t, 1H, *J* = 14.8 Hz), 2.96 (dd, 1H, *J* = 15.6, 6.0 Hz), 2.57 (dd, 1H, *J* = 14.8, 6.8 Hz), 2.42 (t, 1H, *J* = 14.8 Hz), 2.24-2.18 (m, 1H); **^{13}C NMR** (126 MHz, $CDCl_3$): δ 153.6, 150.8, 150.5, 132.1, 123.9, 57.6, 51.0, 33.8, 29.8, 29.1; **IR** ν_{max}: 2971, 2901, 1590, 1558, 1471, 1429, 1379, 1319, 1211, 1087, 1066, 1057, 957, 893, 867; **HRMS** (ESI): $[M+H]^+$ calcd. for $C_{10}H_{11}NBr_2Cl$: 337.8941, found: 337.8942.

3-Chloro-6,7,8,9-tetrahydro-5*H*-cyclohepta[*c*]pyridin-7-ol, **48**, and 3-Chloro-6,7,8,9-tetrahydro-5*H*-cyclohepta[*c*]pyridin-8-ol, **49**

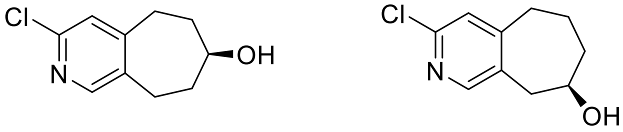

Borane dimethyl sulfide complex (556 µL, 5.56 mmol) was added dropwise to a solution of **17g** (100 mg, 0.56 mmol) in THF (4.5 mL) at 0 °C and stirred at for 30 min, then allowed to warm to rt and stirred for a further 1 h. The solution was re-cooled to 0 °C and 3 M aqueous NaOH solution (1.85 mL, 5.56 mmol) was added dropwise, followed by H_2O_2 (30 wt% in H_2O, 1.90 mL, 16.68 mmol). The mixture was stirred vigorously for 4 h before diluting with Et_2O (5 mL) and brine (5 mL). The layers were separated and the aqueous phase extracted with Et_2O (3x). The combined organic fractions were washed with brine, dried over $MgSO_4$ and evaporated. The residue was purified by flash column chromatography (hexane/Et_2O, 3:1-0:1) to provide the two products:

48: white solid (48 mg, 0.24 mmol, 44%). **^{1}H NMR** (400 MHz, DMSO-d_6): δ 8.11 (s, 1H), 7.29 (s, 1H), 4.72 (d, 1H, *J* = 4.0 Hz), 3.81-3.76 (m, 1H), 2.91-2.85 (m, 2H), 2.59-2.52 (m, 2H), 1.87-1.82 (m, 2H), 1.38-1.36 (m, 2H); **^{13}C NMR** (101 MHz, DMSO-d_6): δ 155.6, 148.7, 148.3, 137.9, 123.5, 70.7, 35.7, 35.0, 28.6, 25.4; **IR** ν_{max}: 3350, 2919, 2853, 1590, 1555, 1472, 1439, 1369, 1319, 1164, 1093, 1048, 1021, 928, 915, 882, 846, 736, 661; **HRMS** (ESI): $[M+H]^+$ calcd. for $C_{10}H_{13}ONCl$: 198.0686, found: 198.0695.

49: white solid (25 mg, 0.13 mmol, 23%). **^{1}H NMR** (400 MHz, DMSO-d_6): δ 8.09 (s, 1H), 7.26 (s, 1H), 4.73 (d, 1H, *J* = 4.0 Hz), 3.55-3.50 (m, 1H), 2.90-2.81 (m, 2H), 2.73-2.69 (m, 2H), 2.00-1.95 (m, 1H), 1.81 -1.66 (m, 2H), 1.41-1.32 (m, 1H); **^{13}C NMR** (101 MHz, $CDCl_3$): δ 156.1, 149.9, 148.4, 133.6, 123.4, 67.4, 40.2, 40.2, 34.1, 23.2; **IR** ν_{max}: 3256, 2932, 2858, 1589, 1558, 1471, 1449, 1371, 1221, 1092, 1051, 1036, 924, 901, 868, 836, 816, 695; **HRMS** (ESI): $[M+H]^+$ calcd. for $C_{10}H_{13}ONCl$: 198.0680, found: 198.0672.

5-Chloro-1a,7,8,8a-tetrahydro-2*H*-oxireno[2',3':5,6]cyclohepta[1,2-*c*]pyridine, **50**

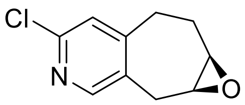

NBS (165 mg, 0.92 mmol) was added portionwise to a solution of **17g** (150 mg, 0.84 mmol) in THF (1.5 mL) and water (1.5 mL) and stirred at rt for 12 h. The reaction was diluted with water, the layers were separated and the aqueous phase extracted with EtOAc (3x). The combined organic fractions were washed with brine, dried over $MgSO_4$ and evaporated. The residue was dissolved in THF (2 mL) and added dropwise at 0 °C to a solution of NaH (60 wt% in mineral oil, 46 mg, 1.90 mmol) in THF (8 mL). The mixture was allowed to warm to rt and stirred for 5 h. The reaction was then quenched with water, the layers were separated and the aqueous phase extracted with Et_2O (3x). The combined organic fractions were washed with brine, dried over $MgSO_4$ and evaporated to provide **50** as a pale yellow oil (76 mg, 0.39 mmol, 47%).

^{1}H NMR (400 MHz, DMSO-d_6): δ 8.14 (s, 1H), 7.33 (s, 1H), 3.41 (dd, 1H, *J* = 15.6, 4.4 Hz), 3.25 (app q, 1H, *J* = 4.4 Hz), 3.11 (dd, 1H, *J* = 15.6, 4.4 Hz), 3.01 (t, 1H, *J* = 4.4 Hz), 2.69 (ddd, 1H, *J* = 13.2, 8.2, 4.0 Hz), 2.54 (ddd, 1H, *J* = 13.2, 10.0, 3.6 Hz), 2.19 (ddd, 1H, *J* = 14.8, 8.2, 3.6 Hz), 2.02-1.94 (m, 1H); **^{13}C NMR** (101 MHz, DMSO-d_6): δ 154.1, 148.9, 148.6, 132.0, 123.2, 53.5, 52.3, 29.4, 27.4, 25.5; **IR** ν_{max}: 2971, 1590, 1557, 1470, 1432, 1372, 1099, 1079, 1055, 1022, 913, 866, 756, 734; **HRMS** (ESI): $[M+H]^+$ calcd. for $C_{10}H_{11}ONCl$: 196.0529, found: 196.0535.

cis-3-chloro-6,7,8,9-tetrahydro-5*H*-cyclohepta[*c*]pyridine-7,8-diol, **51**

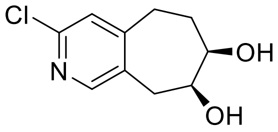

The compound was prepared in an analogous way to **40**, using osmium tetroxide (144 μL, 0.014 mmol), **17g** (50 mg, 0.278 mmol), NMO (49 mg, 0.418 mmol), citric acid (108 mg, 0.562 mmol), THF (1.0 mL) and water (0.5 mL). After purification by flash column chromatography (hexane/EtOAc, 1:1-1:9), **51** was obtained as a dark green solid (10 mg, 0.047 mmol, 17%).

^{1}H NMR (400 MHz, DMSO-d_6): δ 8.04 (s, 1H), 7.22 (s, 1H), 4.64 (d, 1H, *J* = 4.4 Hz), 4.55 (d, 1H, *J* = 3.2 Hz), 3.72 (br s, 1H), 3.62 (br s, 1H), 3.05 (t, 1H, *J* = 14.0 Hz), 2.85 (t, 1H, *J* = 13.6 Hz), 2.57 (d, 1H, *J* = 14.0 Hz), 2.48-2.42 (m, 1H), 1.70-1.53 (m, 2H); **^{13}C NMR** (101 MHz, DMSO-d_6): δ 155.5, 149.9, 148.3, 133.5, 123.2, 73.4, 70.0, 33.4, 30.2, 28.1; **IR** ν_{max}: 3431, 3256, 2923, 1586, 1553, 1471, 1459, 1433, 1353, 1231, 1099, 1082, 1045, 1025, 1003, 925, 913, 863, 813, 673; **HRMS** (ESI): $[M+H]^+$ calcd. for $C_{10}H_{13}O_2NCl$: 214.0629, found: 214.0622.

3. Computational evaluation of physicochemical properties

Computational analysis was performed using the Molecular Operating Environment (MOE) software package, version 2012.10, from the Chemical Computing Group.

Merck molecular force field 94X (MMFF94x), an all-atom force field parameterised for small organic molecules with the Generalised Born solvation model, was used to minimise the energy potential of the library members. A LowModeMD search was employed for the conformation generation. Detailed settings for the conformational search are listed below. The option to "enforce chair conformations" was de-selected.

**Conformational Search Settings**	
Method	LowModeMD
Rejection Limit	100
RMS Gradient	0.005
Iteration Limit	10000
MM Iteration Limit	500
RMSD Limit	0.15
Energy Window	3
Conformation Limit	10000

Only final compounds in their fully deprotected forms were analysed. The relevant structures are shown in Figure 1.

The compounds were analysed for the following properties: SlogP, molecular weight (MW), topological polar surface area (PSA), number of hydrogen-bond acceptors (HBA), number of hydrogen-bond donors (HBD), number of heavy (ie. non-hydrogen) atoms (HAC), number of rotatable bonds (RBC), number of chiral centres and fraction aromatic (the number of aromatic atoms expressed as a fraction of the total number of heavy atoms). NB. All properties of hydrochloride salts were calculated excluding the chloride counteranion.

The distribution of these data and the mean values are displayed in a series of histograms in Figure 2.

By means of comparison with existing libraries, the mean values are shown alongside those of two popular commercially available fragment libraries, Chembridge (consisting of 7,547 fragments) and Maybridge (consisting of 29,819 fragments), in Table 1.

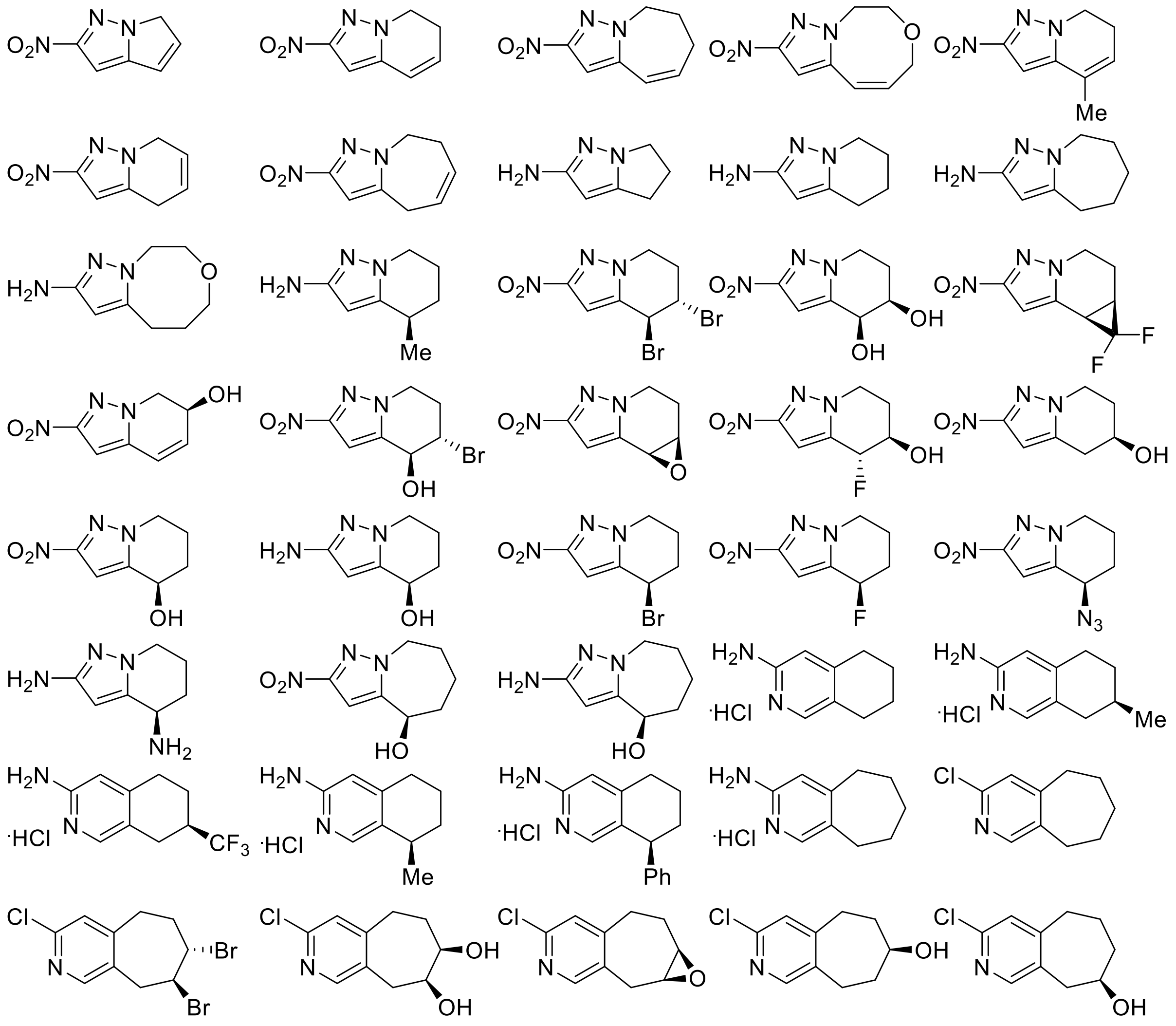

Figure 1: The structures of the analysed final compounds.

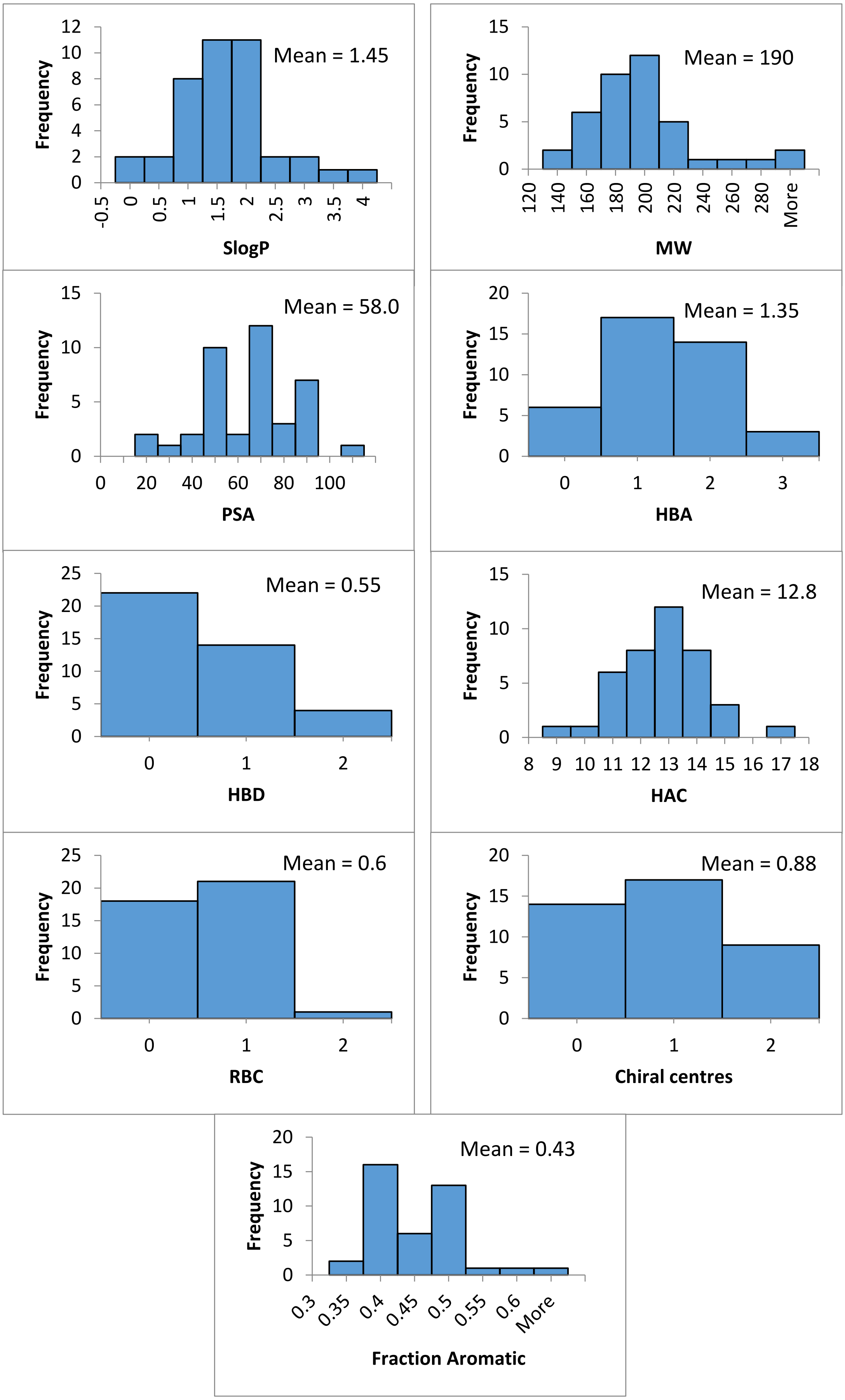

Figure 2: Histograms showing the distribution of physicochemical properties amongst the compounds in Figure 1. Mean values are also included.

Property	This work	Chembridge	Maybridge
SlogP	1.45	1.31	2.55
MW	190	222	265
PSA	58.0	53.9	57.5
HBA	1.35	1.81	2.12
HBD	0.55	1.04	0.81
HAC	12.8	15.5	18.0
RBC	0.6	3.2	2.8
Chiral centres	0.88	0.27	0.18
Fraction Aromatic	0.43	0.42	0.52

Table 1: Mean physicochemical properties of the library presented in this work and the commercially available fragment libraries available from Chembridge and Maybridge.

4. References

[1] L. Leonard, B. Lygo, G. Proctor, *Advanced Practical Organic Chemistry, 2nd ed.,* Blackie Academic and Professional, Glasgow, **1995**.

[2] A. F. Burchat, J. M. Chong, N. Nielsen, *J. Organomet. Chem.* **1997**, *542*, 281-283.

5. NMR Spectra

3-Nitro-1-((2-(trimethylsilyl)ethoxy)methyl)-1*H*-pyrazole, **5**

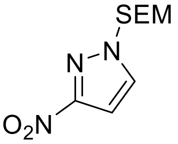

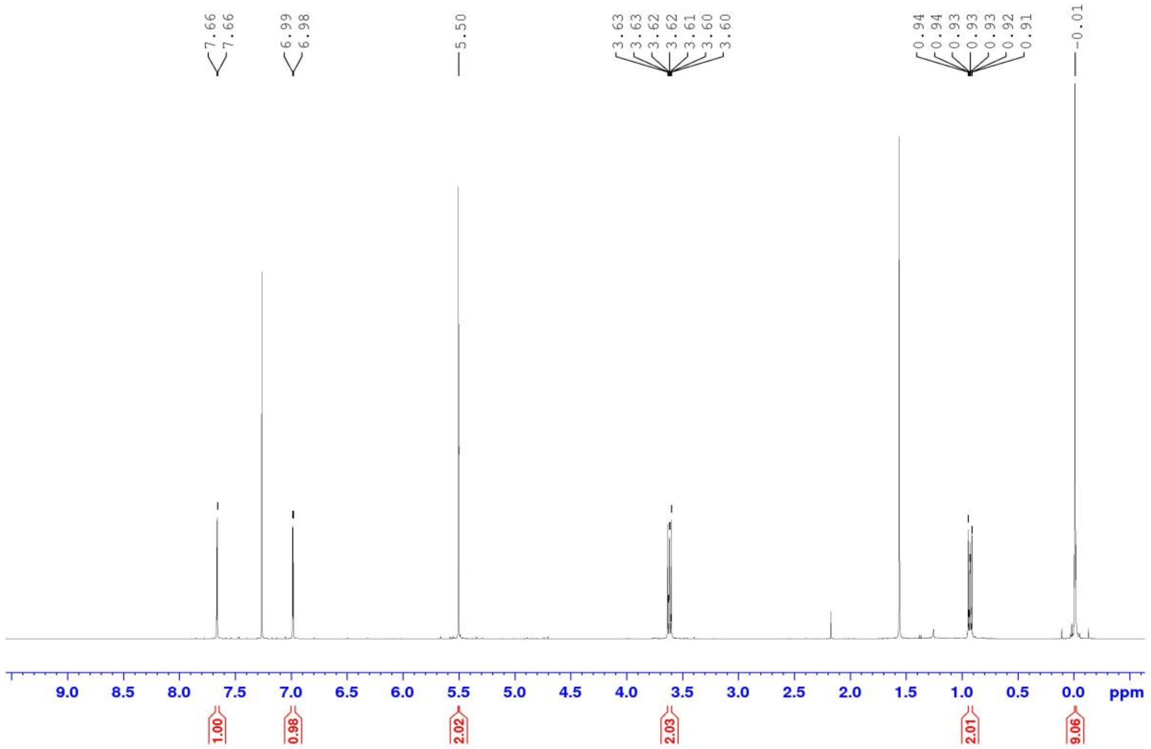

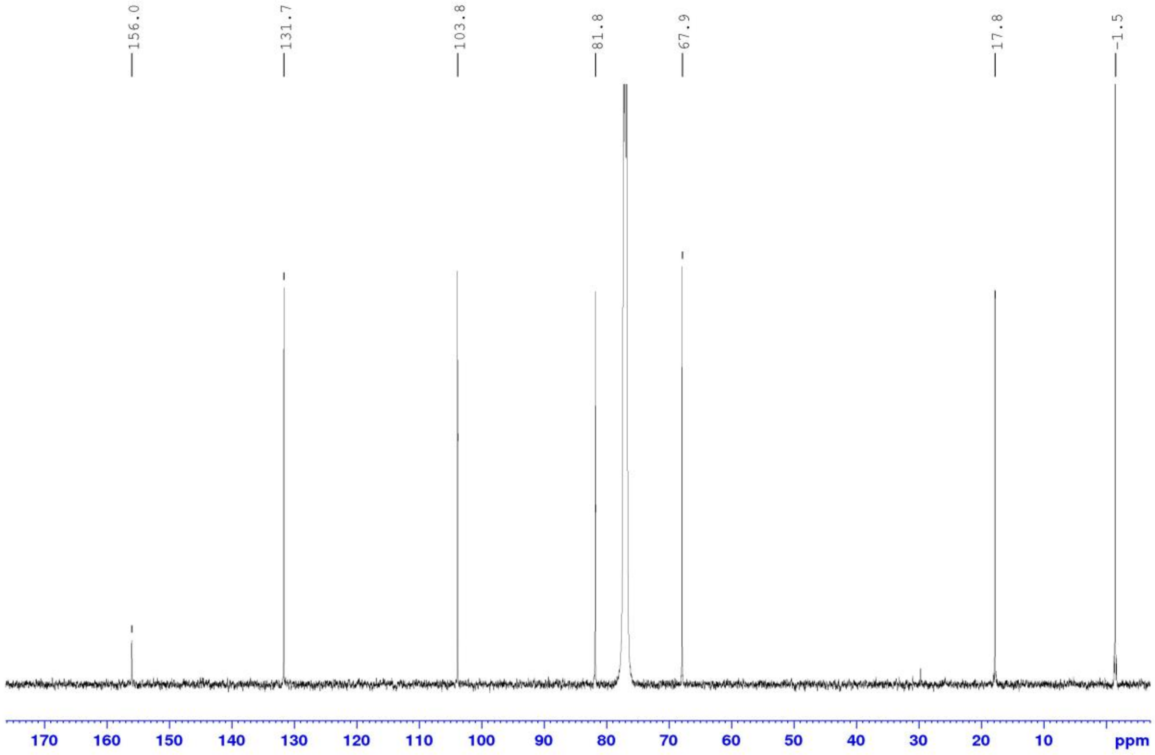

5-Iodo-3-nitro-1-((2-(trimethylsilyl)ethoxy)methyl)-1*H*-pyrazole, **6**

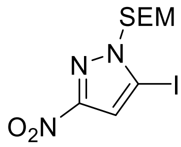

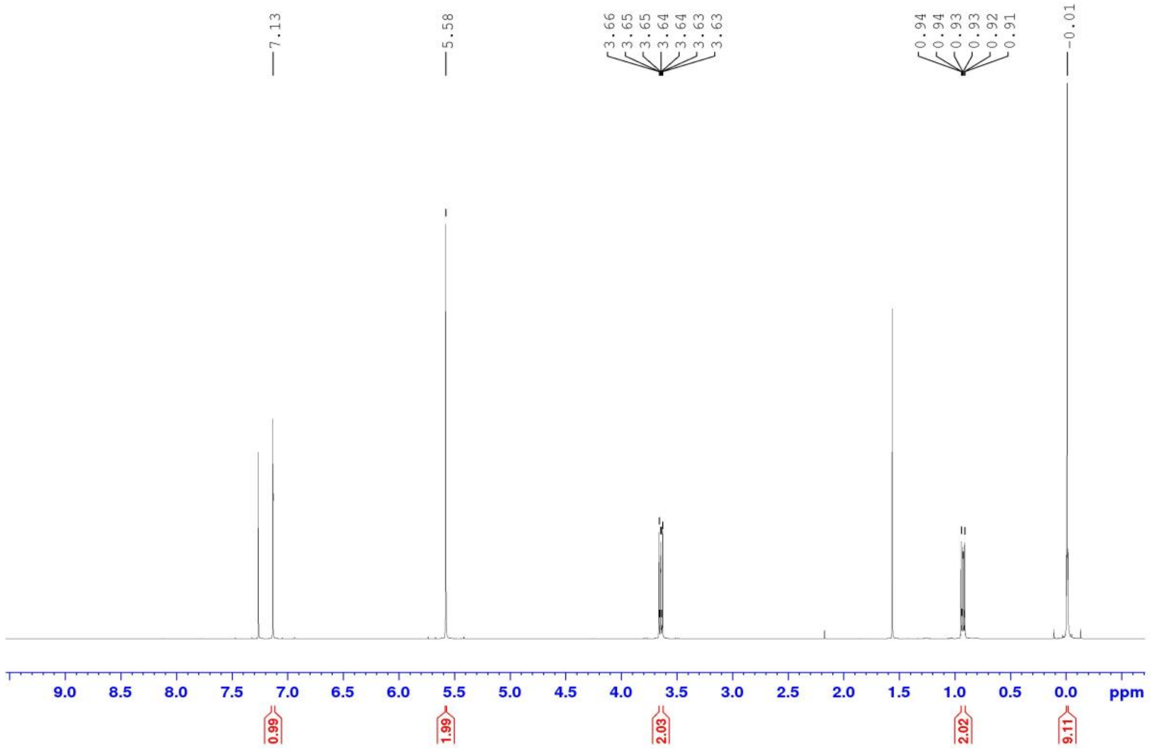

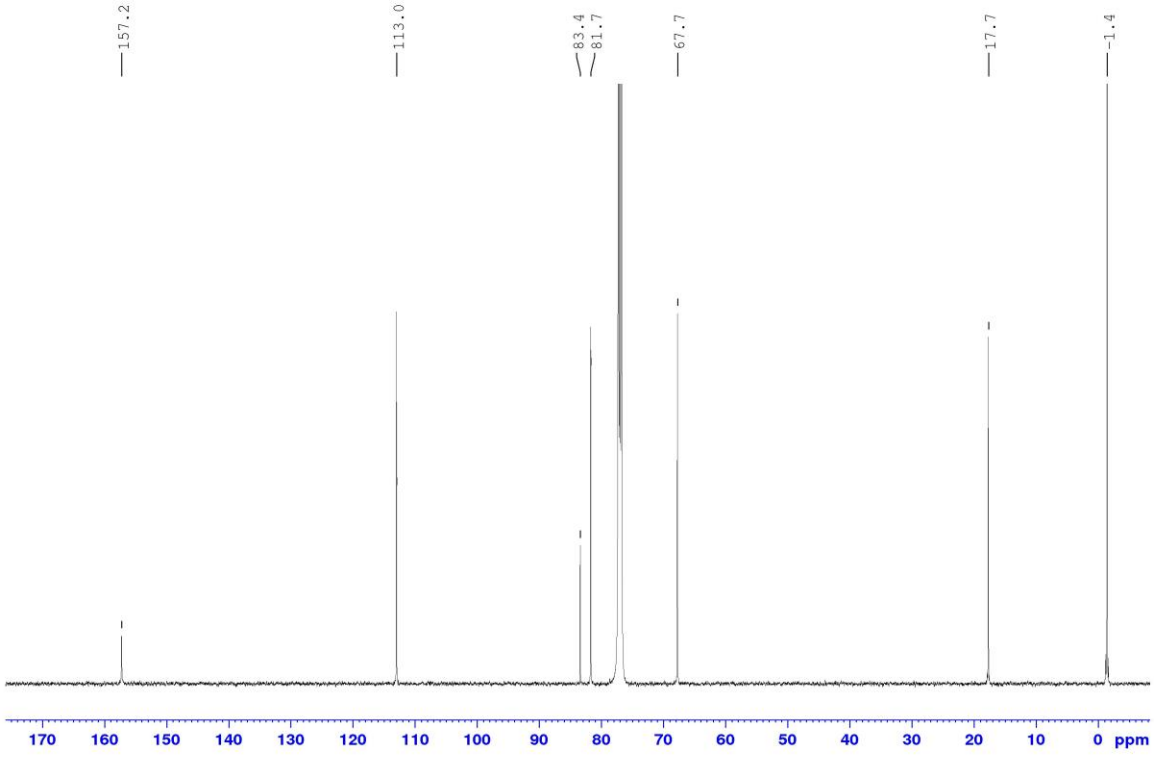

3-Nitro-1-((2-(trimethylsilyl)ethoxy)methyl)-5-vinyl-1*H*-pyrazole, **7a**

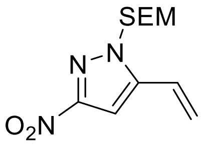

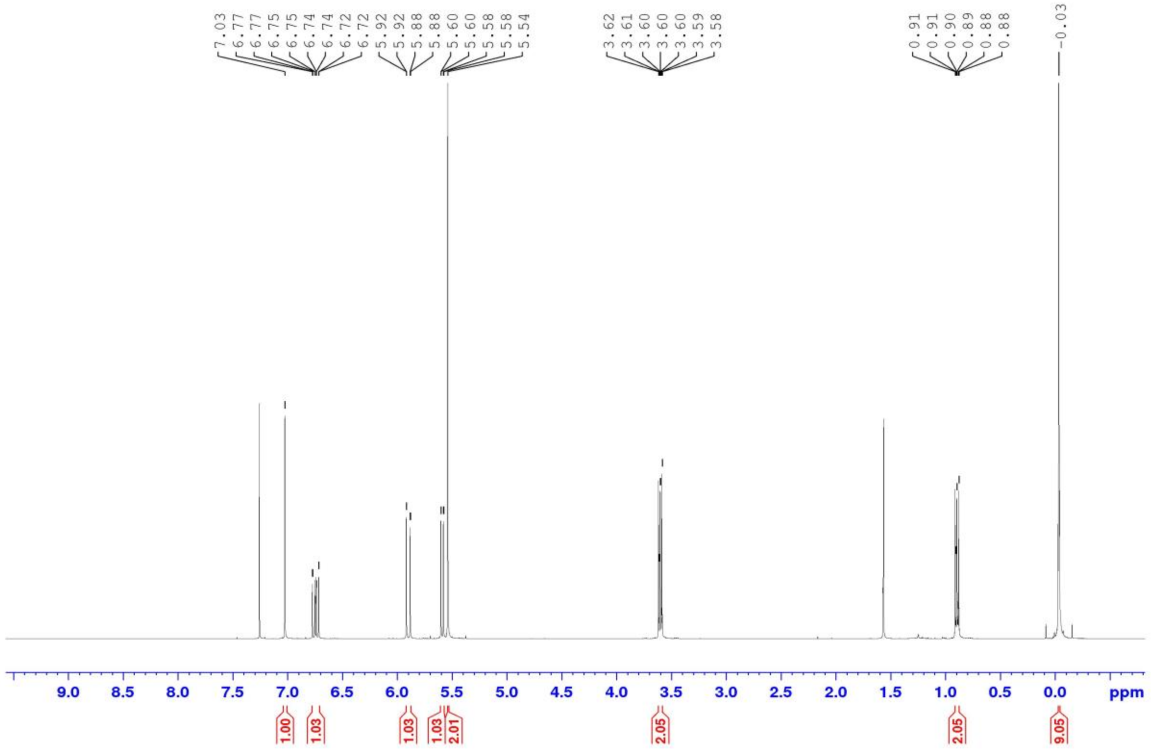

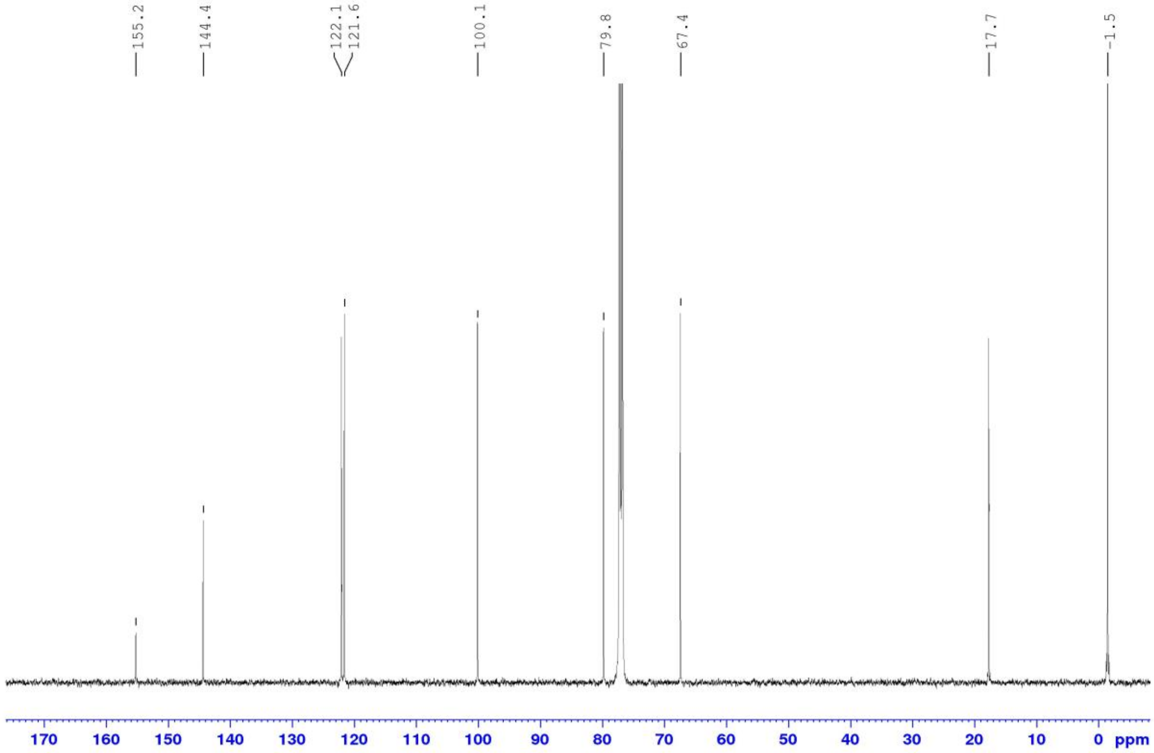

3-Nitro-5-(prop-1-en-2-yl)-1-((2-(trimethylsilyl)ethoxy)methyl)-1*H*-pyrazole, **7f**

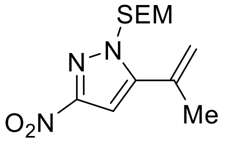

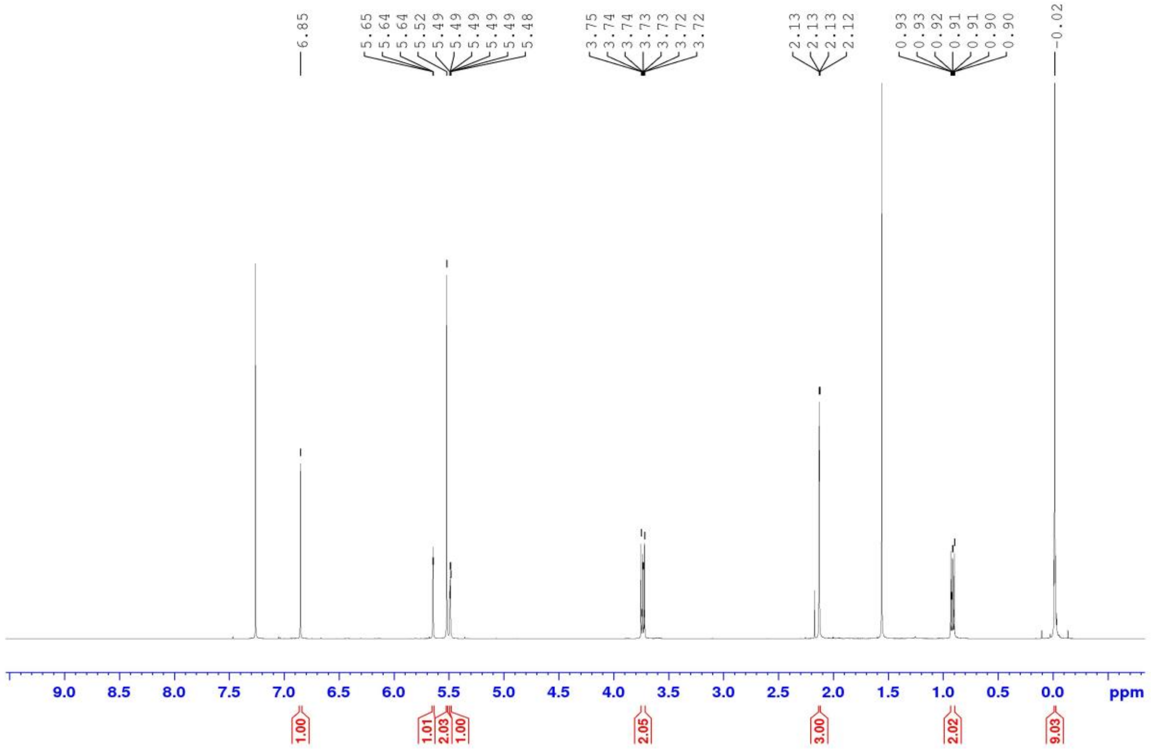

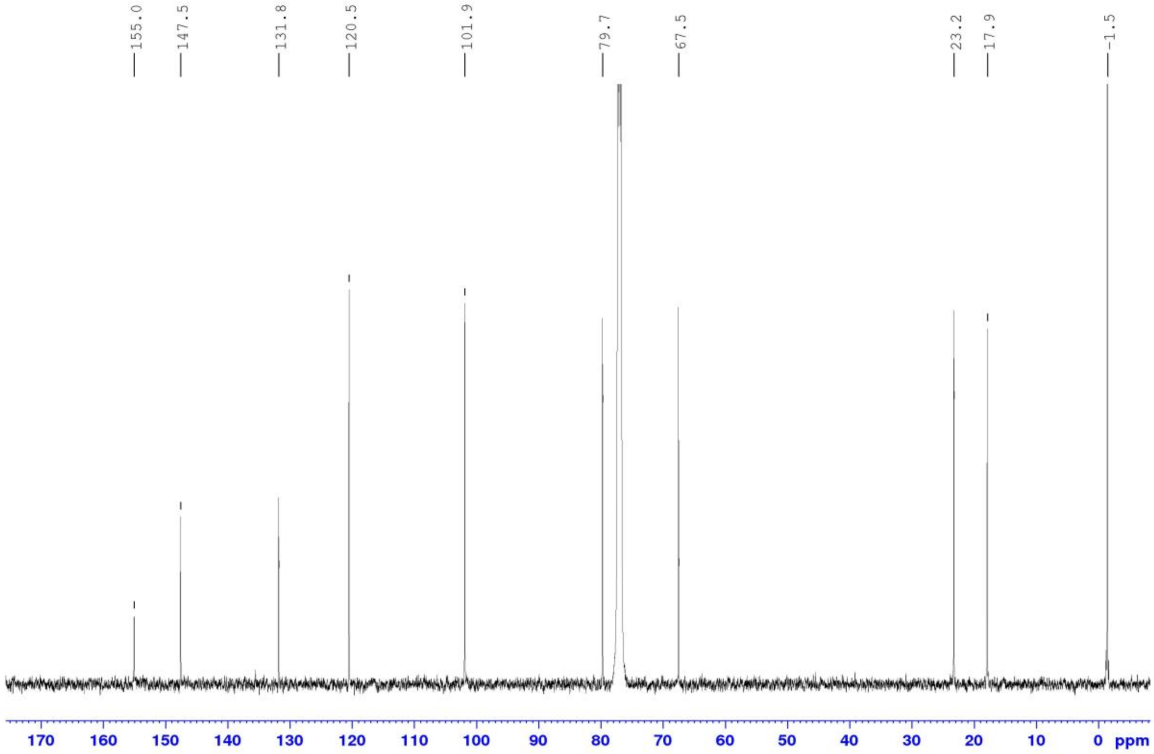

5-Allyl-3-nitro-1-((2-(trimethylsilyl)ethoxy)methyl)-1*H*-pyrazole, **7g**

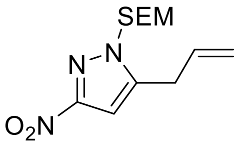

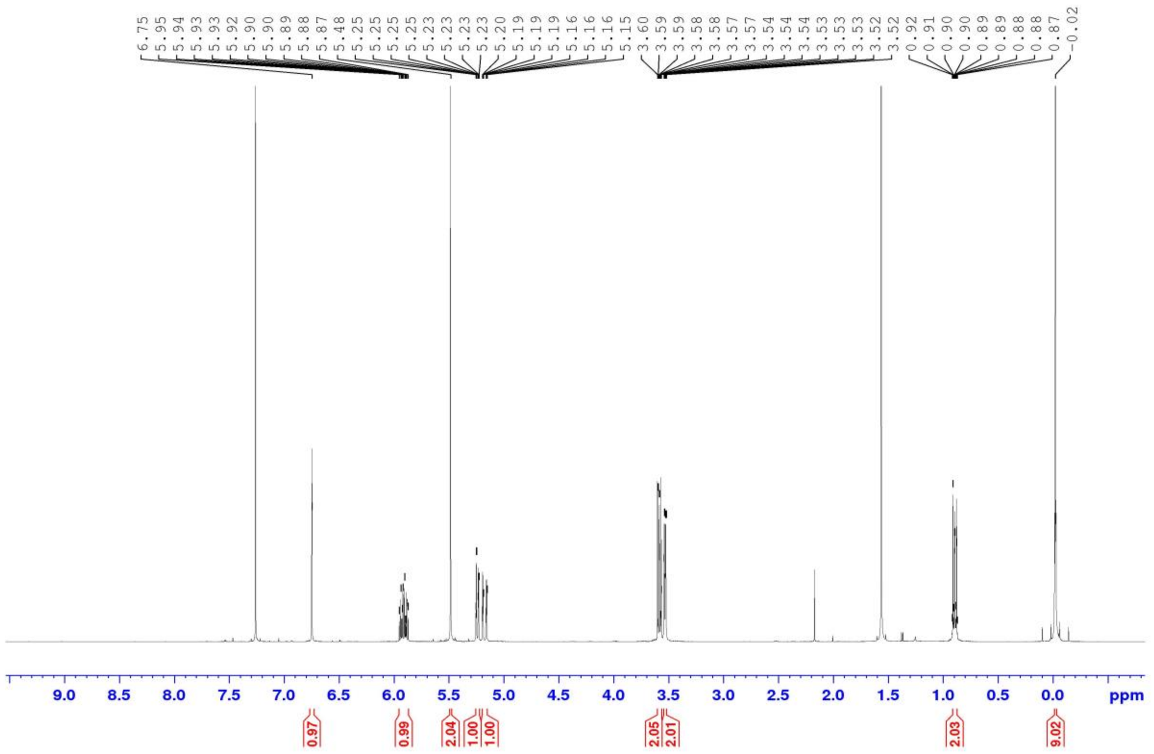

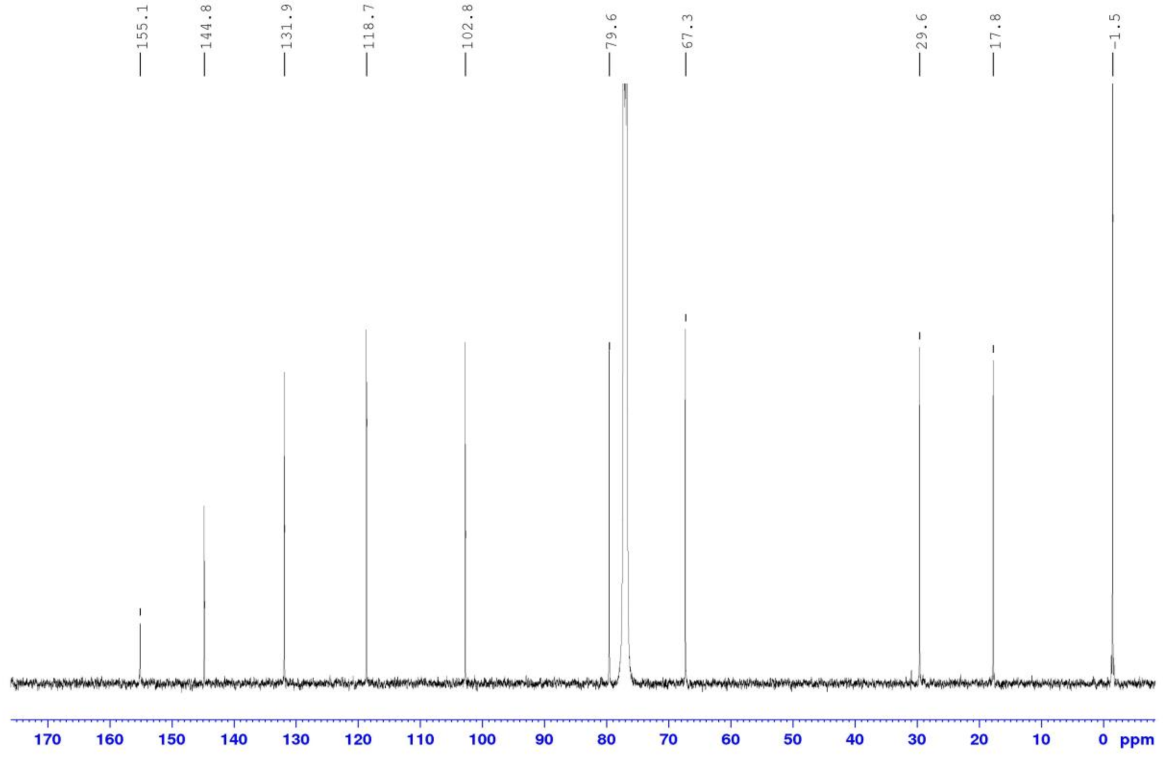

3-nitro-5-vinyl-1*H*-pyrazole, **8a**

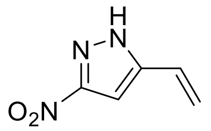

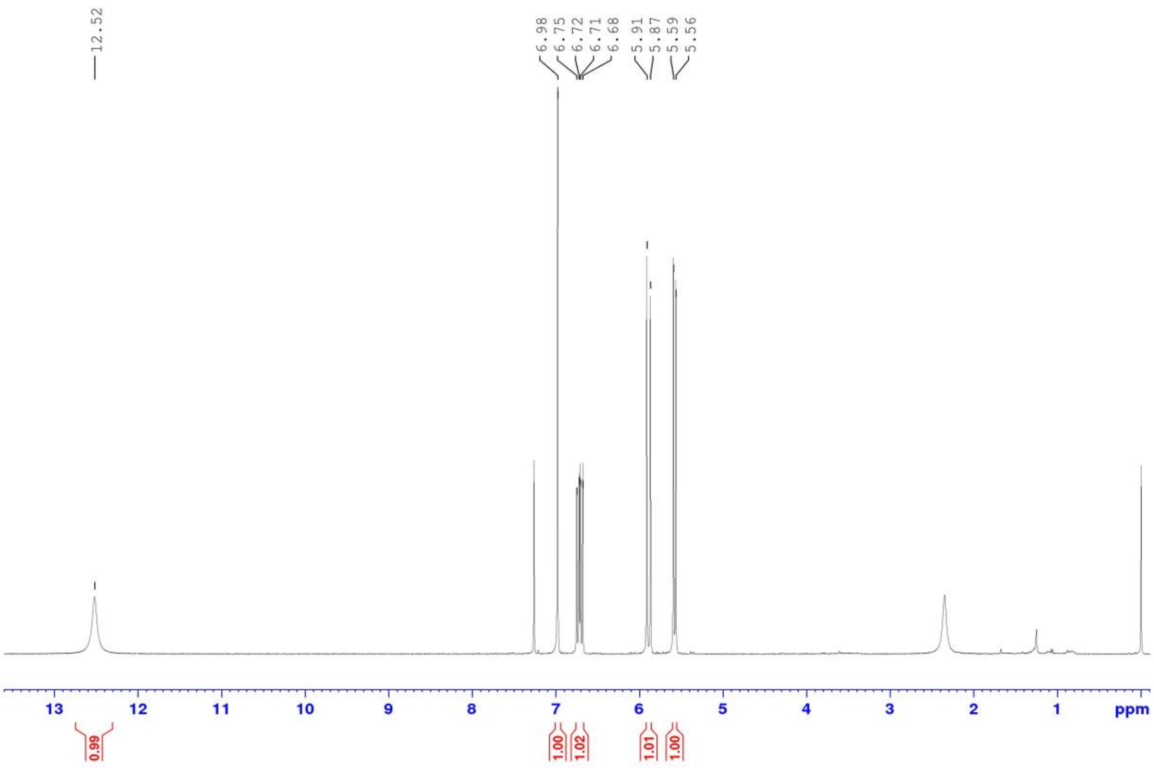

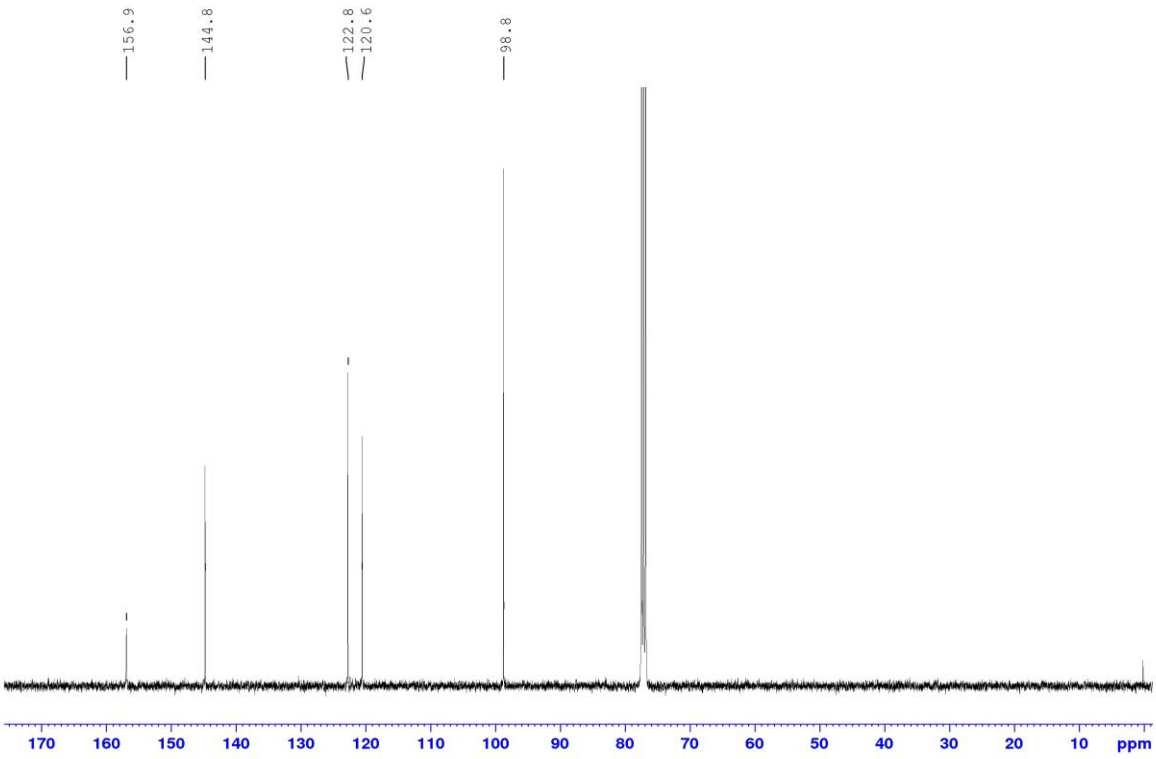

3-Nitro-5-(prop-1-en-2-yl)-1*H*-pyrazole, **8f**

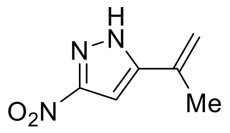

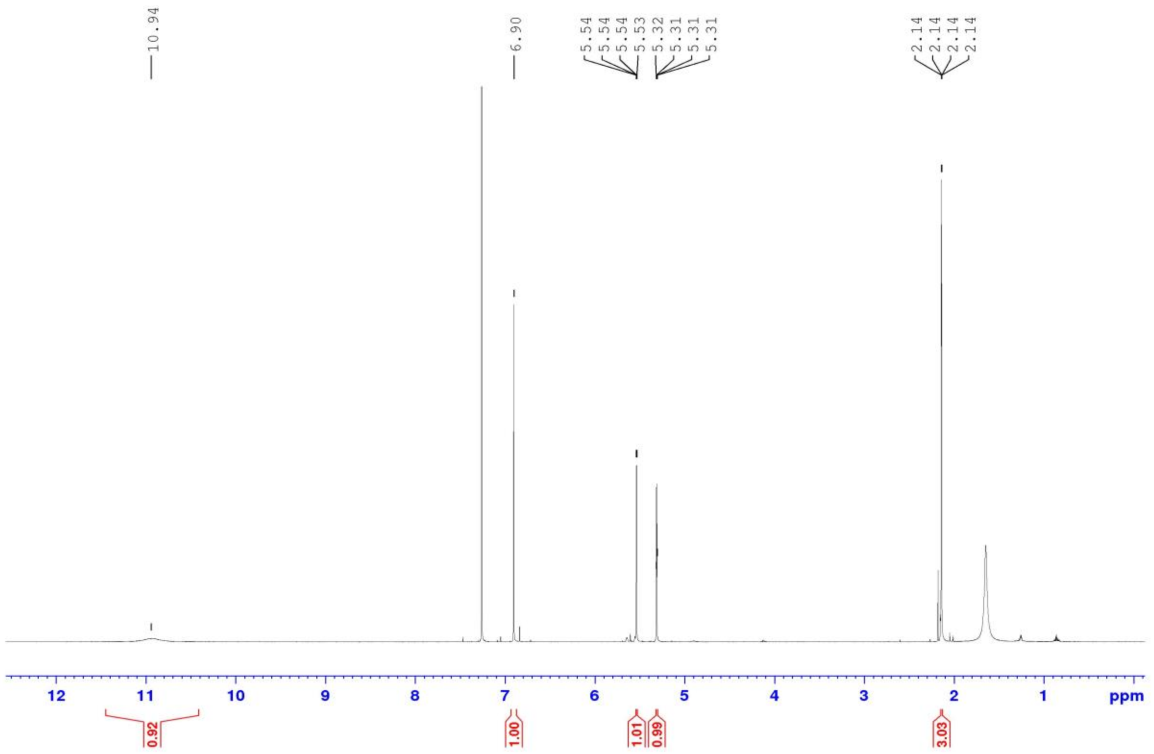

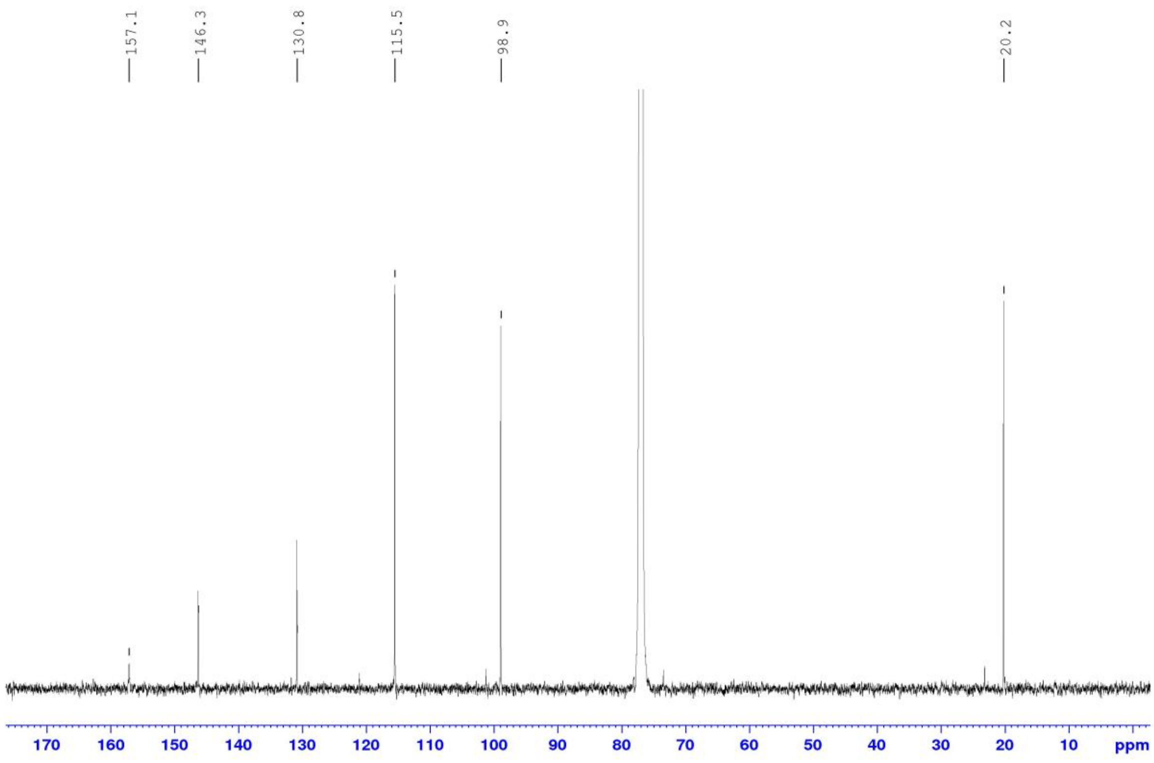

5-Allyl-3-nitro-1*H*-pyrazole, **8g**

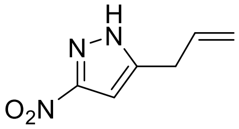

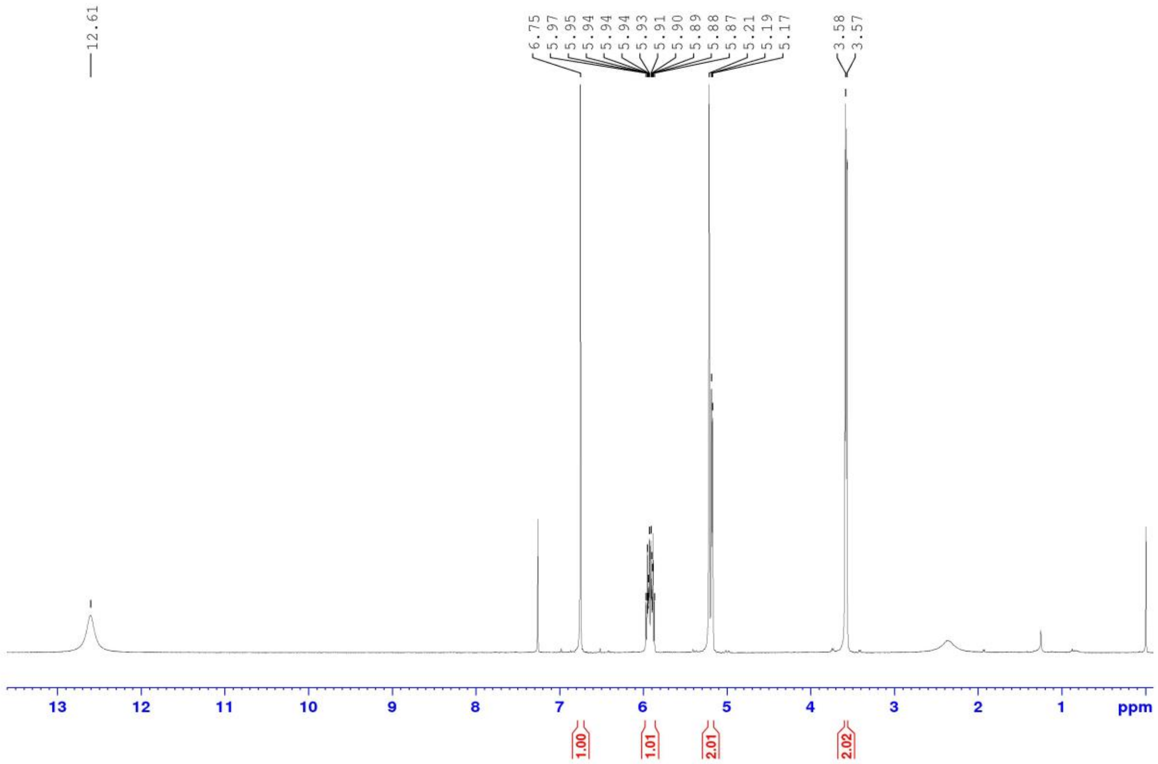

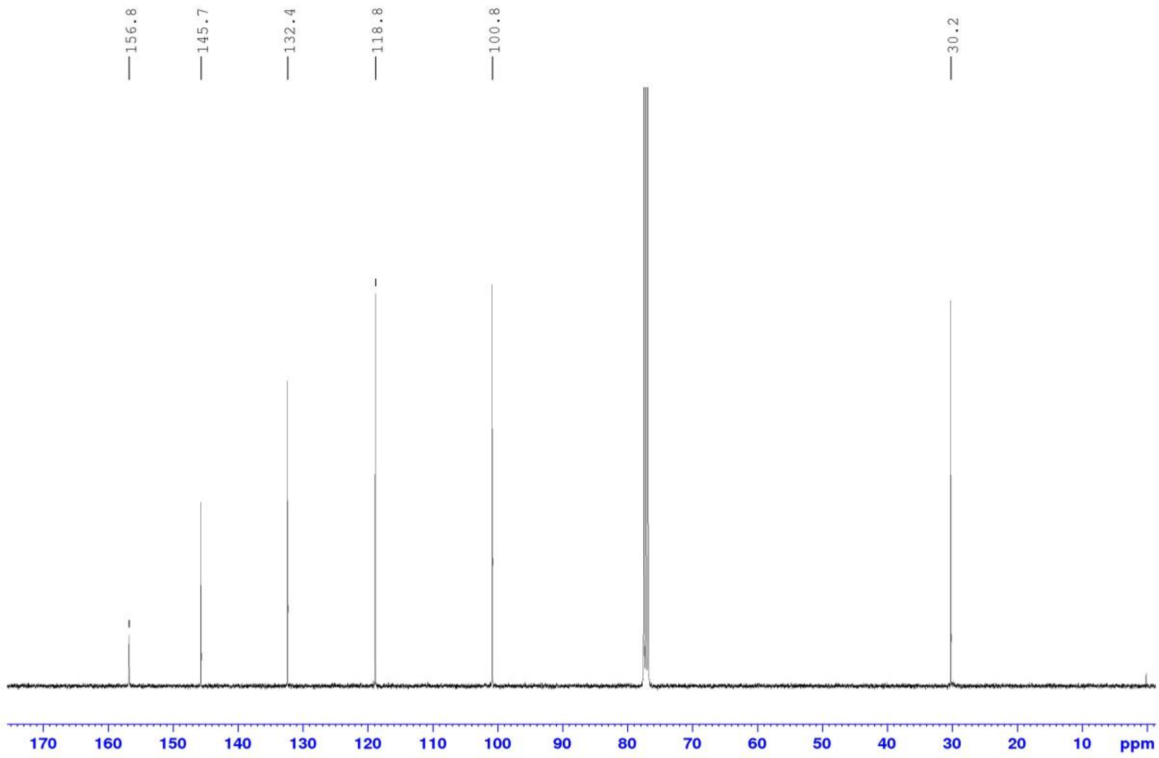

1-Allyl-3-nitro-5-vinyl-1*H*-pyrazole, **9a**

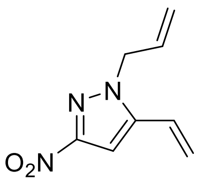

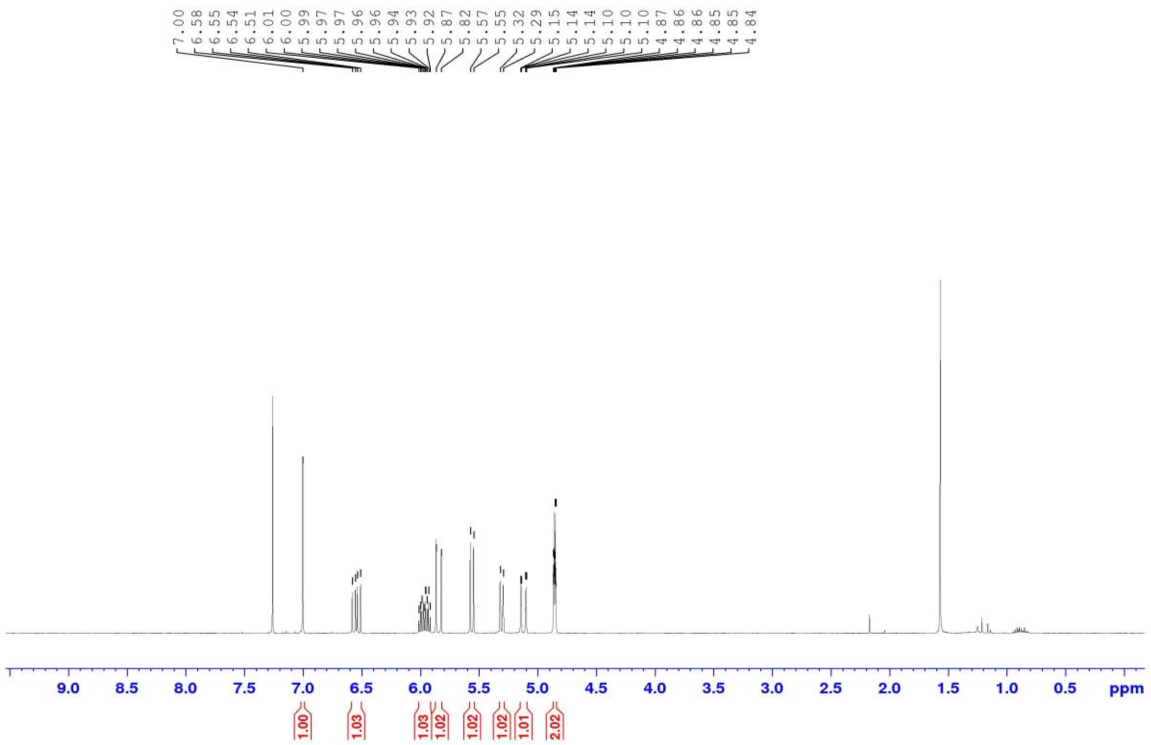

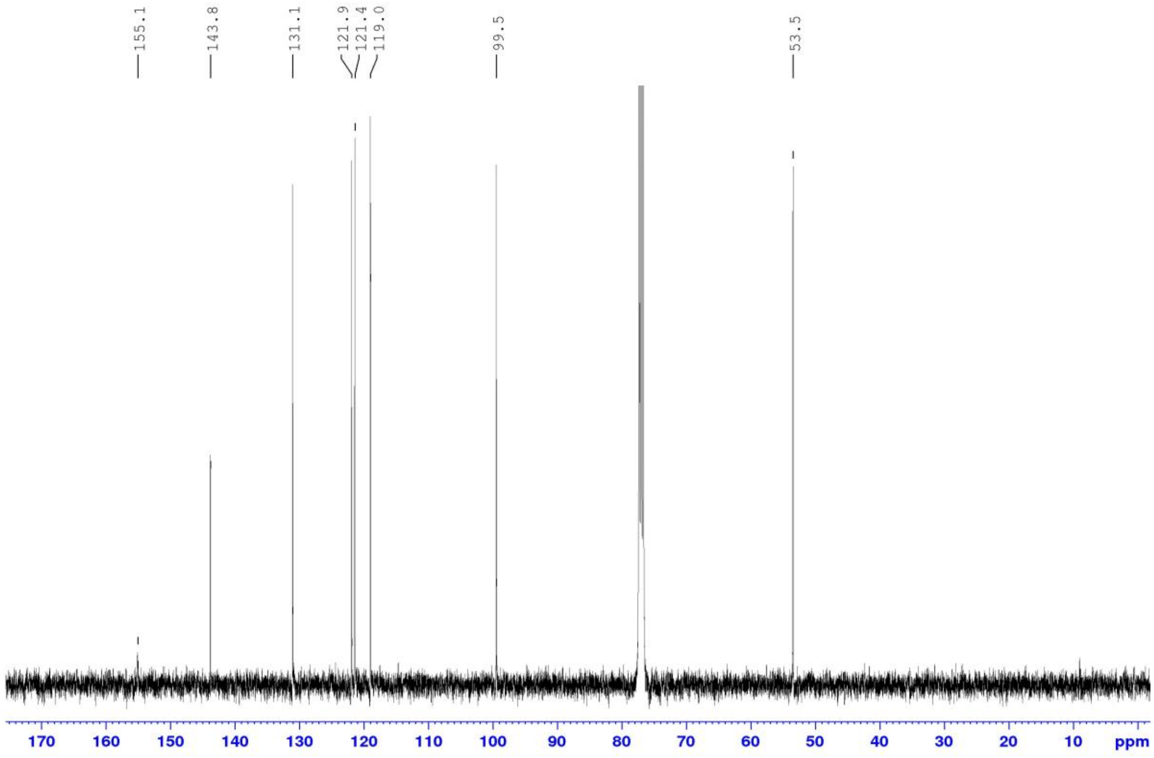

1-(But-3-en-1-yl)-3-nitro-5-vinyl-1*H*-pyrazole, **9b**

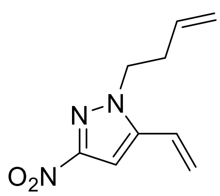

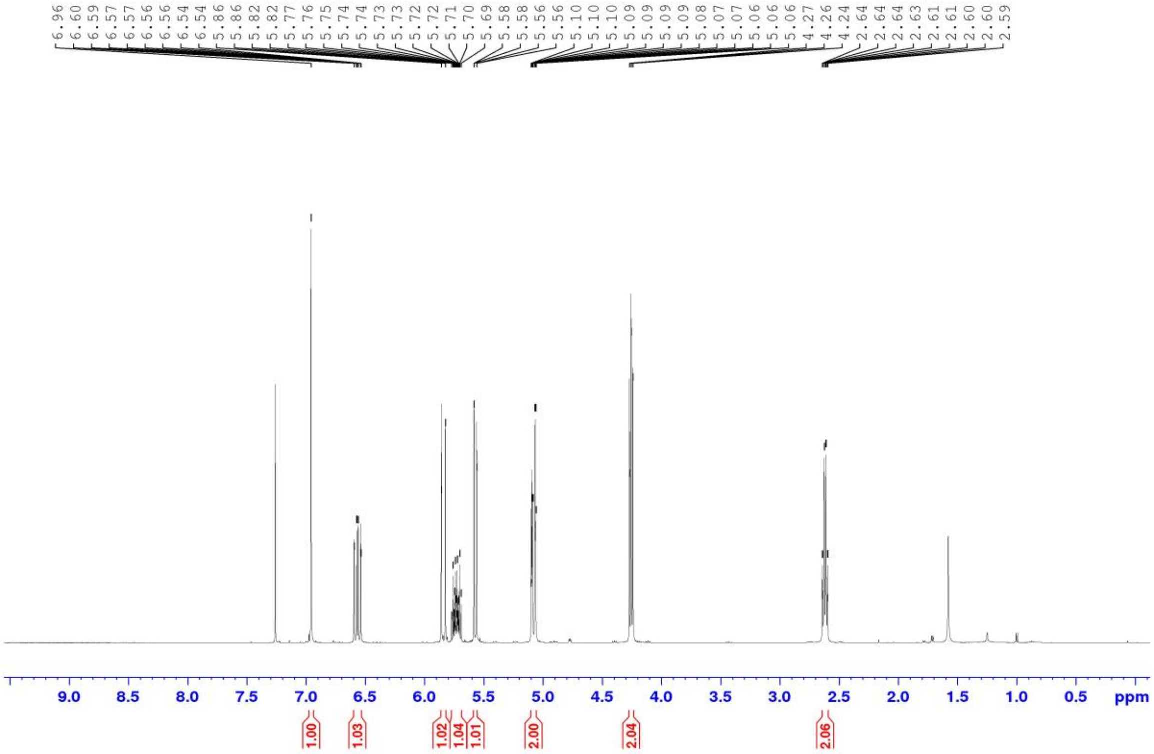

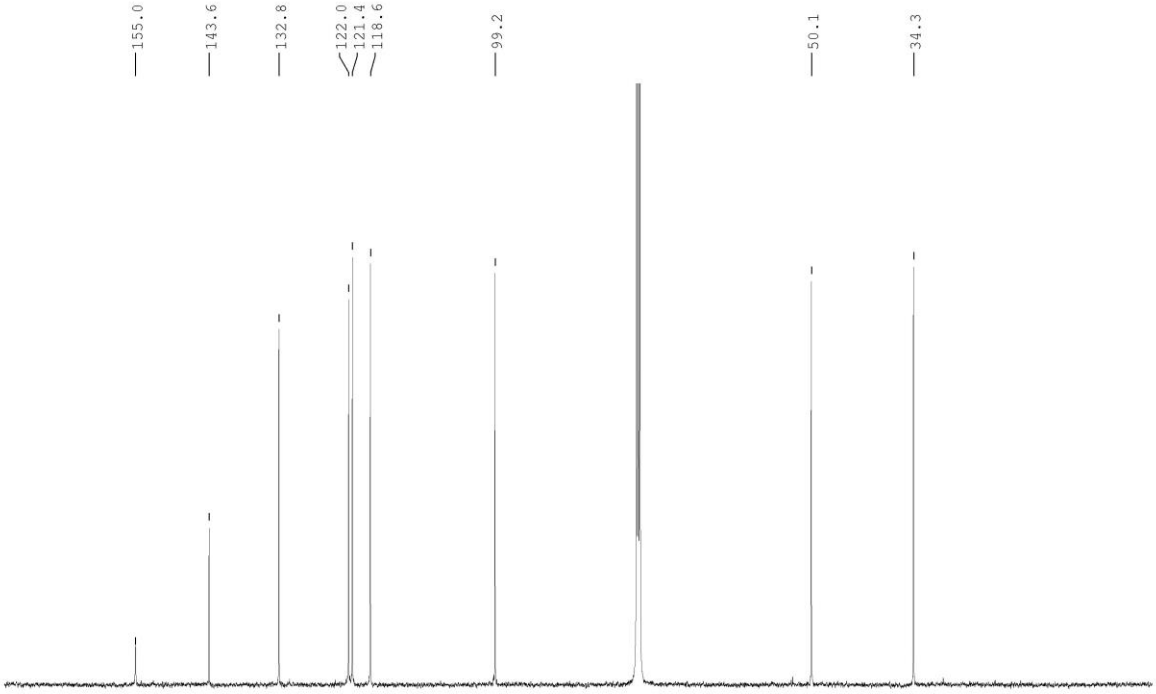

3-Nitro-1-(pent-4-en-1-yl)-5-vinyl-1*H*-pyrazole, **9c**

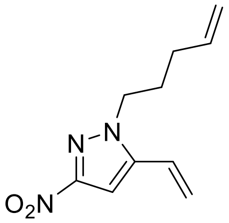

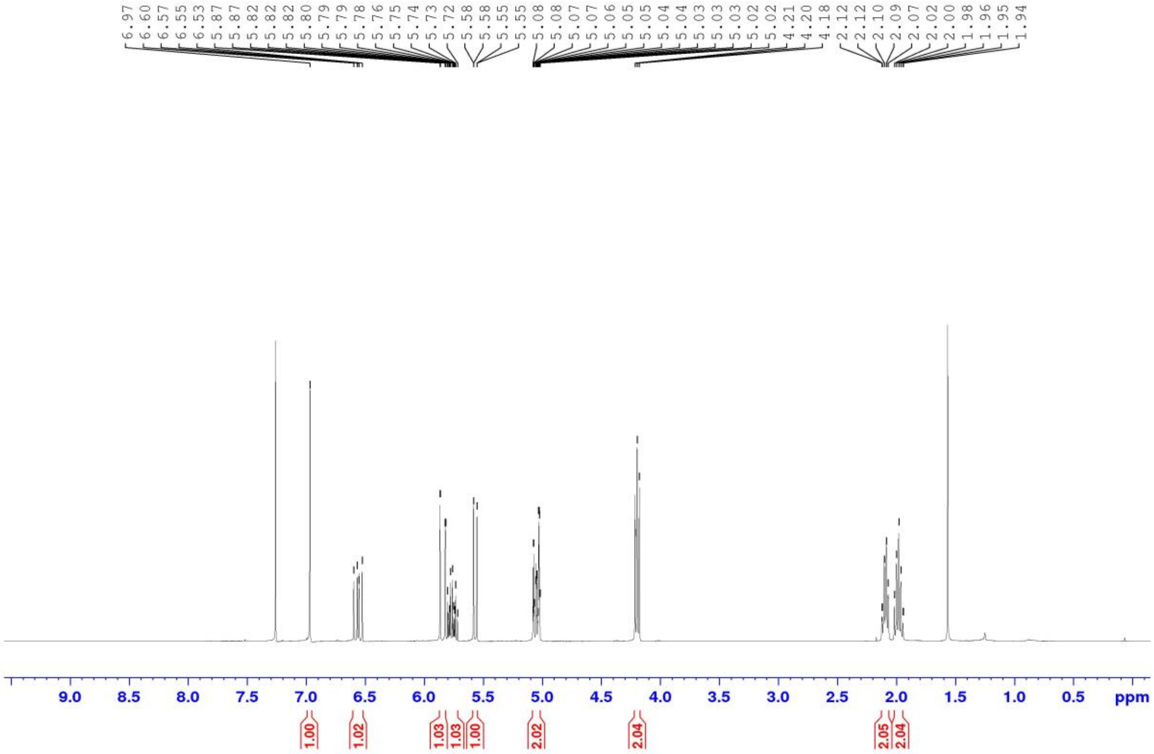

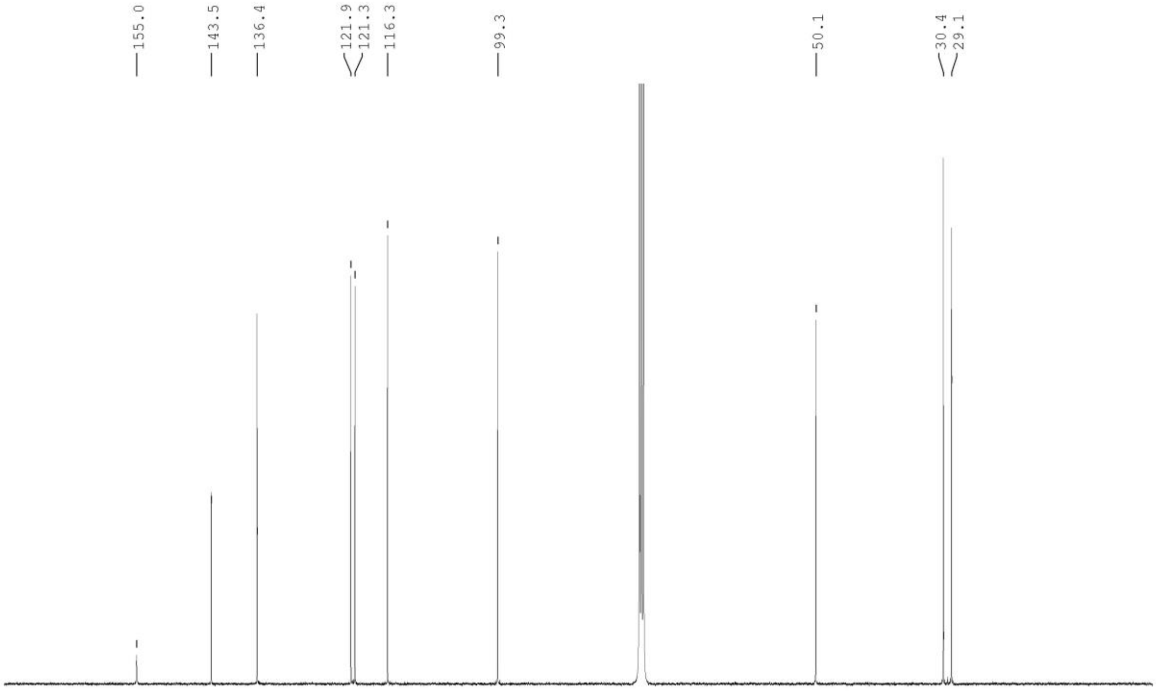

1-(2-(Allyloxy)ethyl)-3-nitro-5-vinyl-1*H*-pyrazole, **9d**

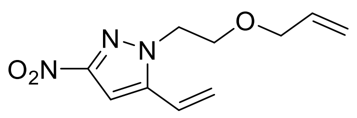

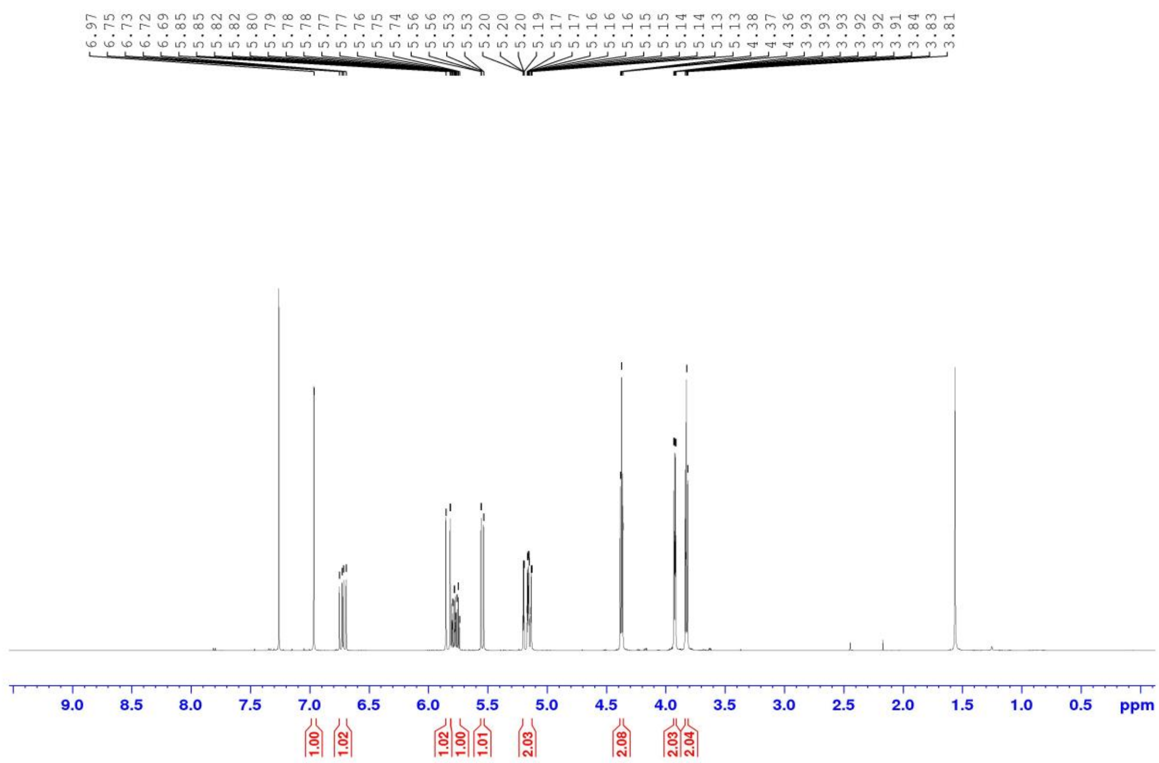

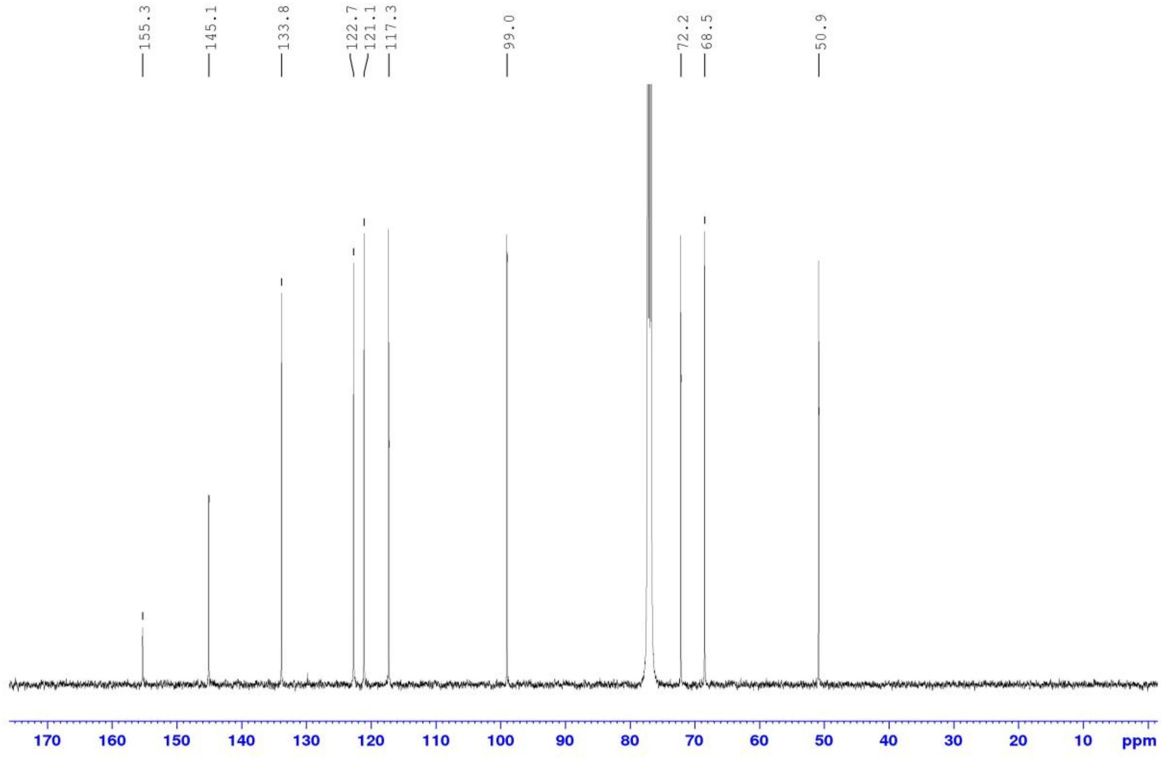

tert-Butyl (2-(3-nitro-5-vinyl-1*H*-pyrazol-1-yl)ethyl)carbamate, **52**

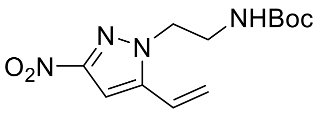

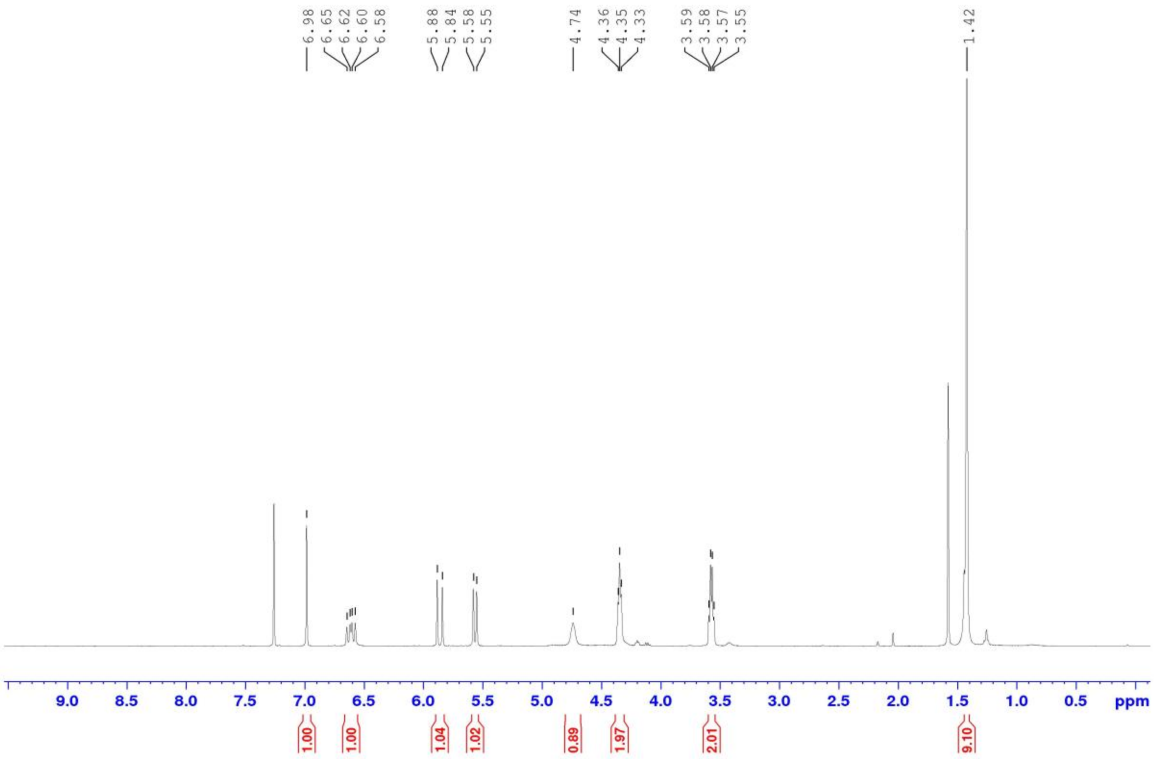

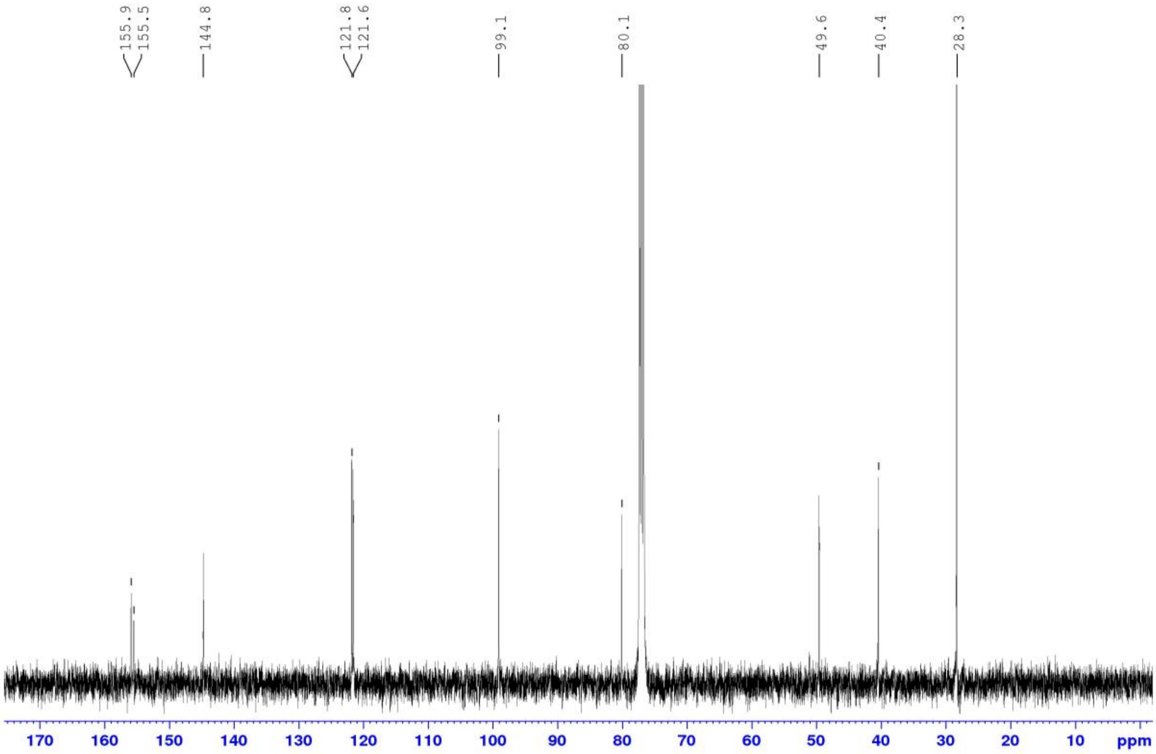

tert-Butyl allyl(2-(3-nitro-5-vinyl-1*H*-pyrazol-1-yl)ethyl)carbamate, **9e**

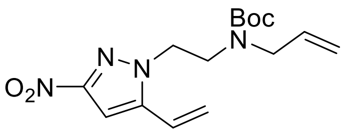

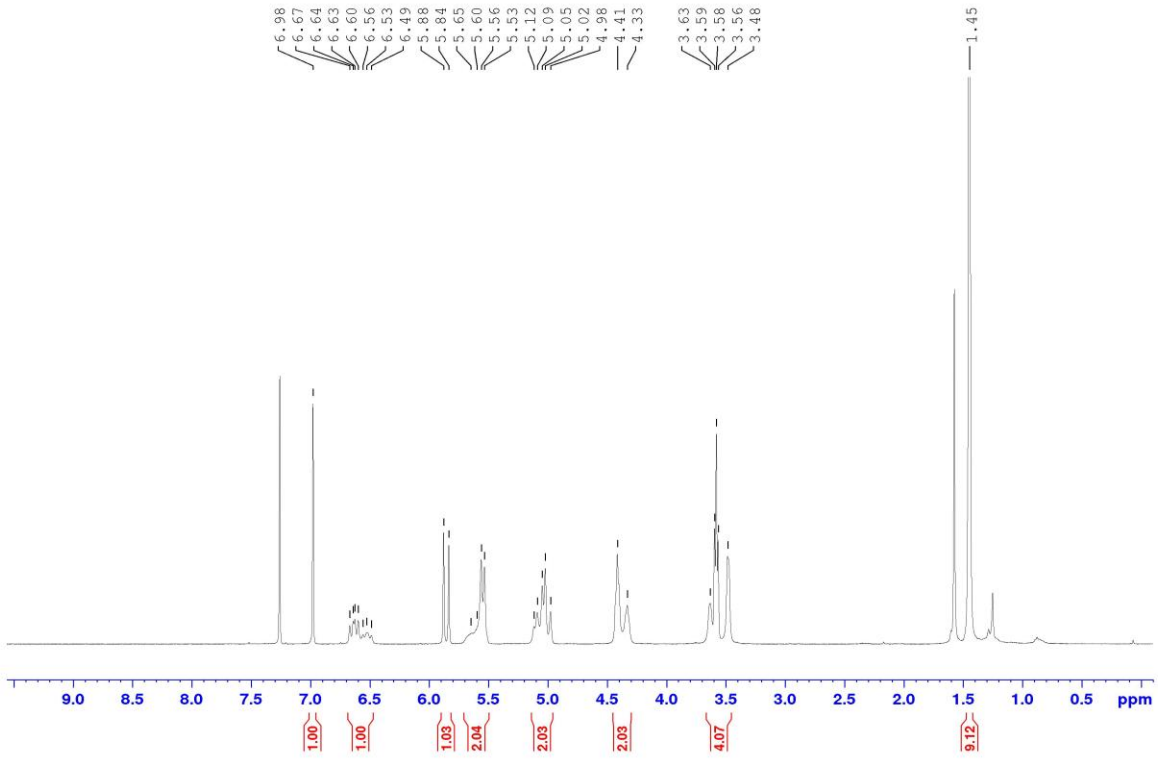

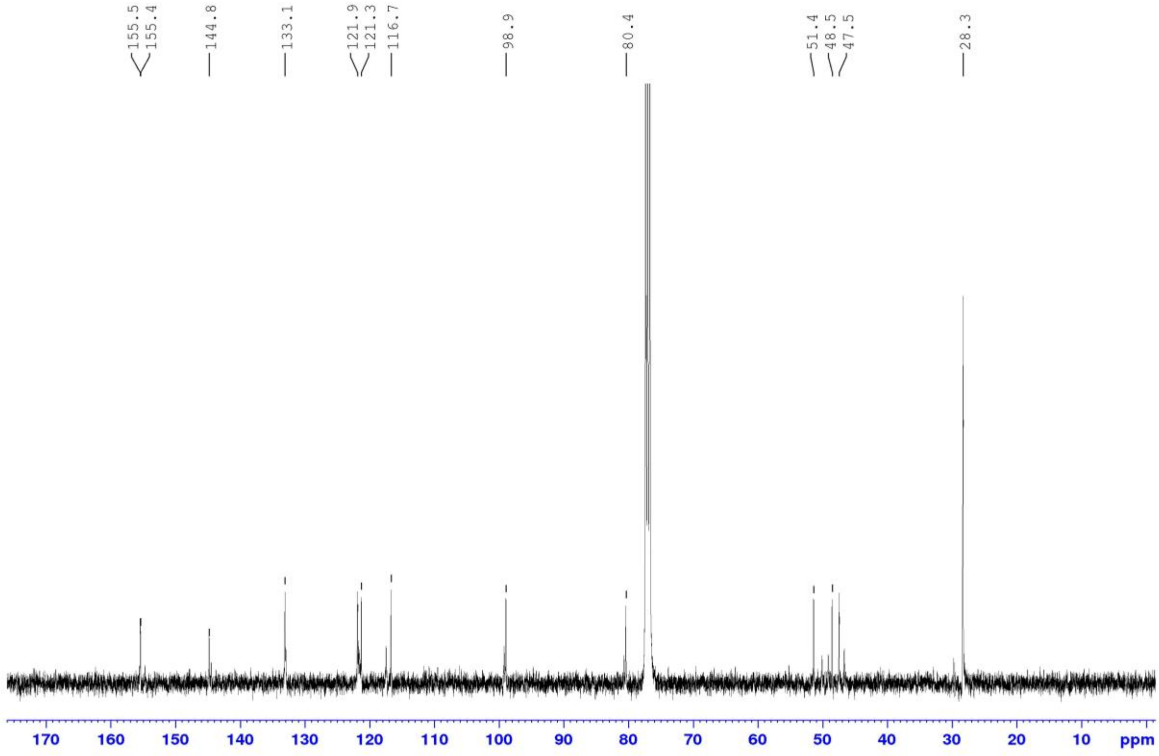

1-(But-3-en-1-yl)-3-nitro-5-(prop-1-en-2-yl)-1*H*-pyrazole, **9f**

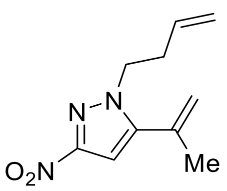

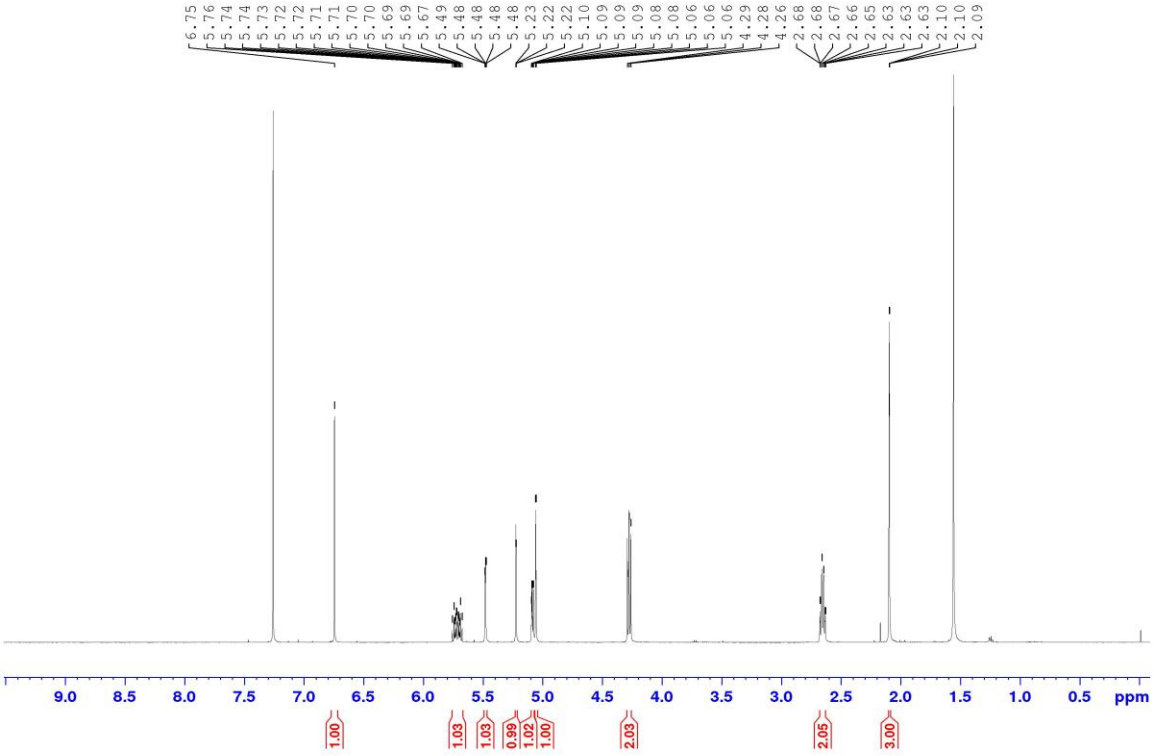

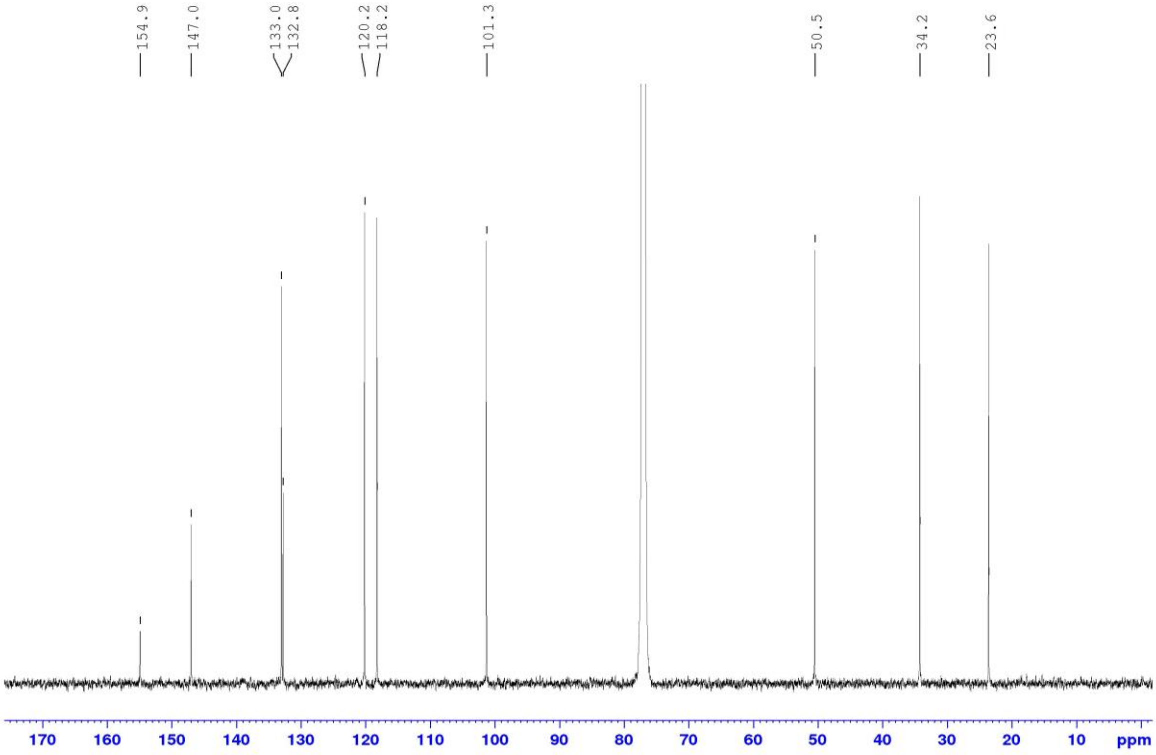

1,5-Diallyl-3-nitro-1*H*-pyrazole, **9g**

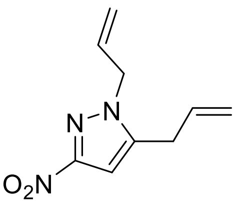

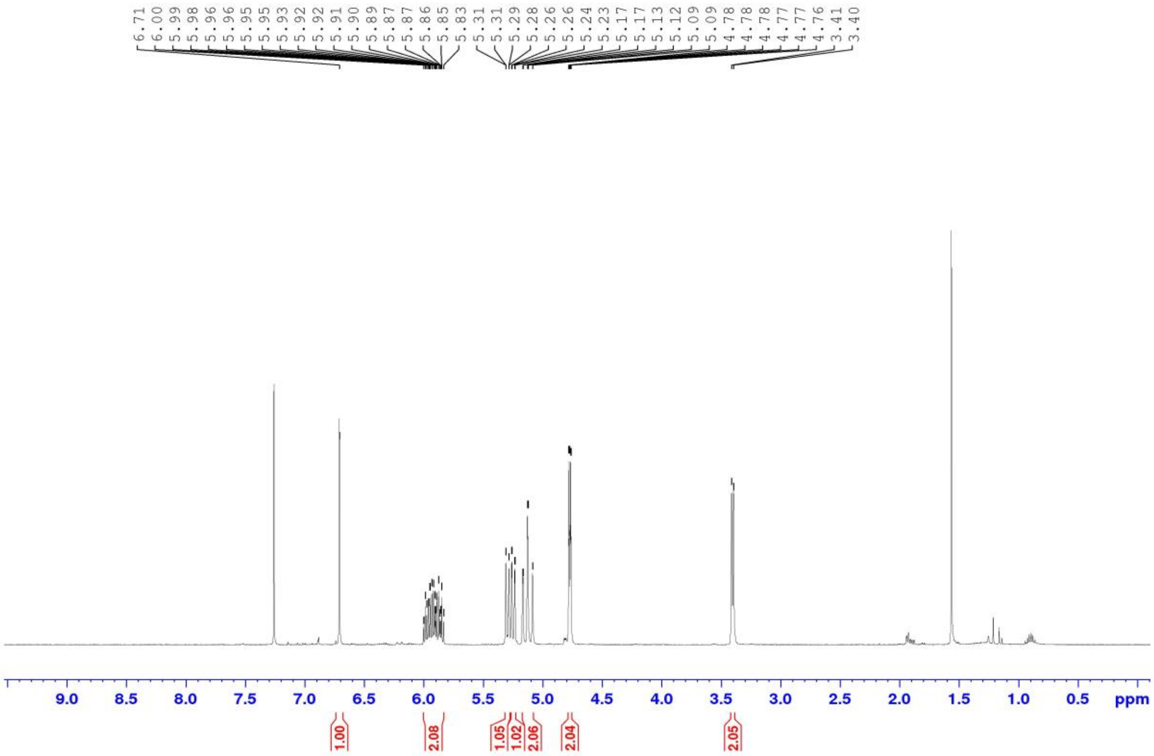

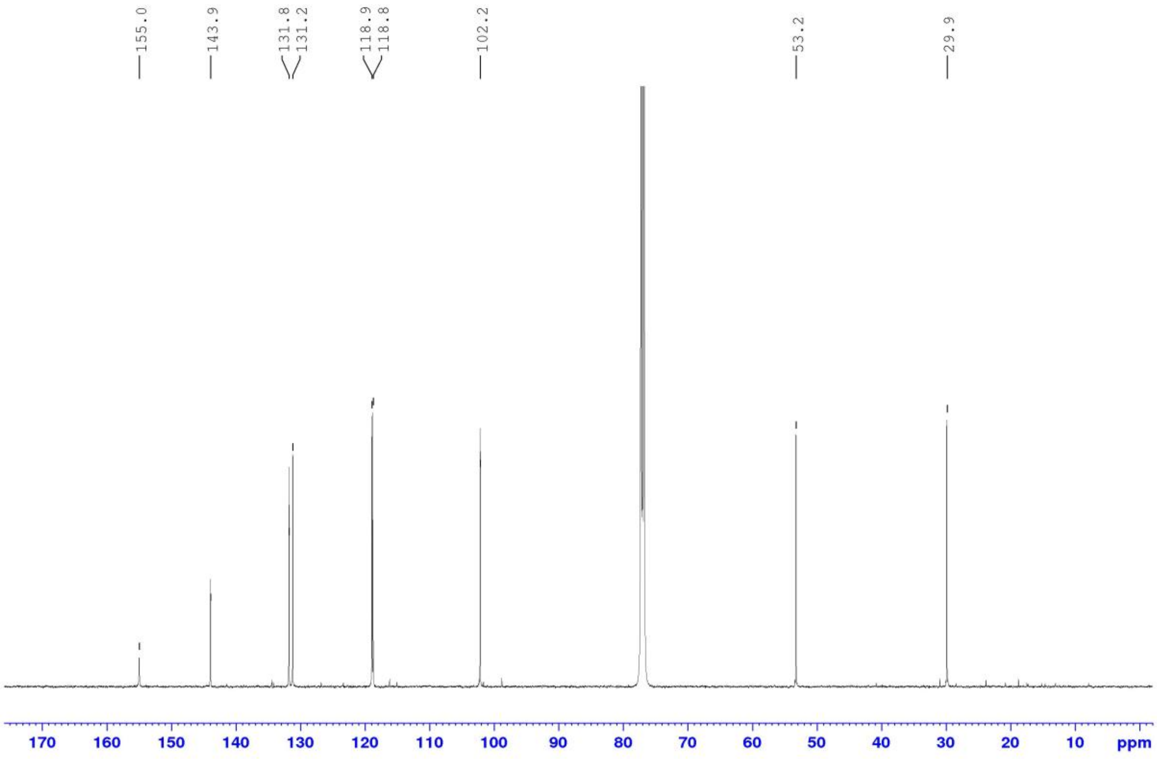

5-Allyl-1-(but-3-en-1-yl)-3-nitro-1*H*-pyrazole, **9h**

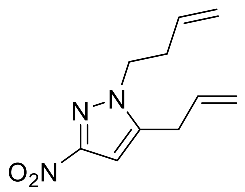

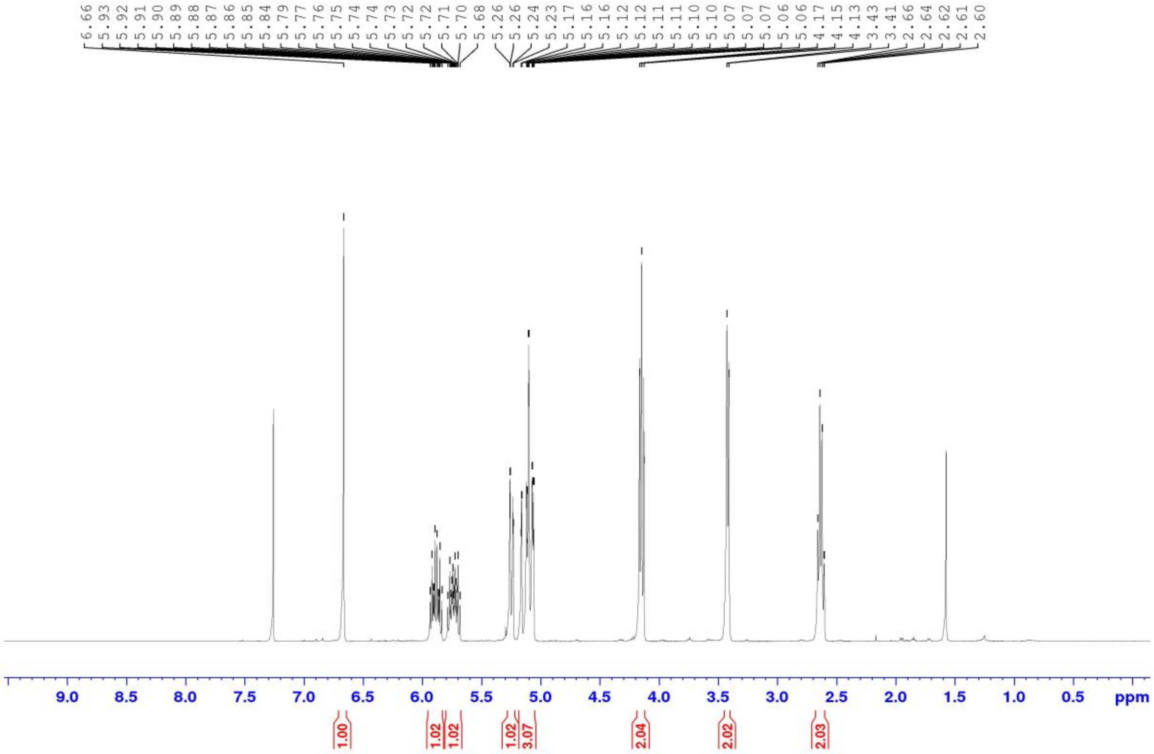

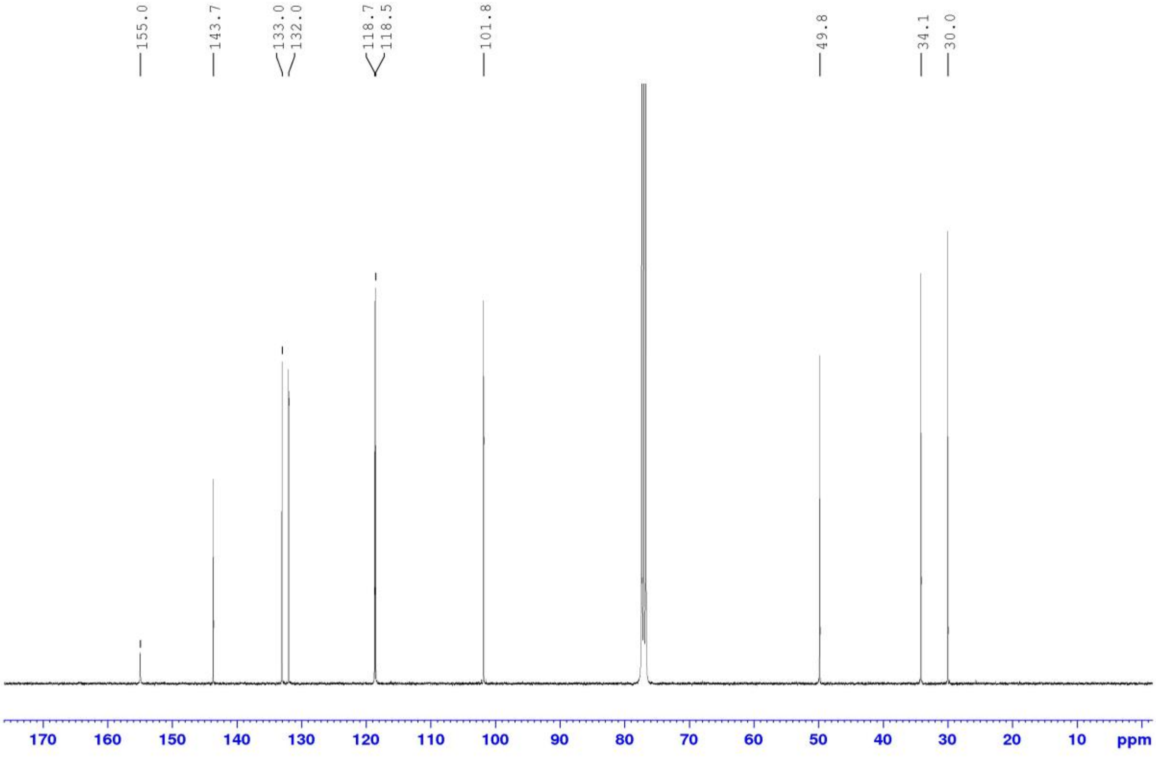

2-Nitro-6*H*-pyrrolo[1,2-*b*]pyrazole, **10a**

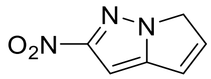

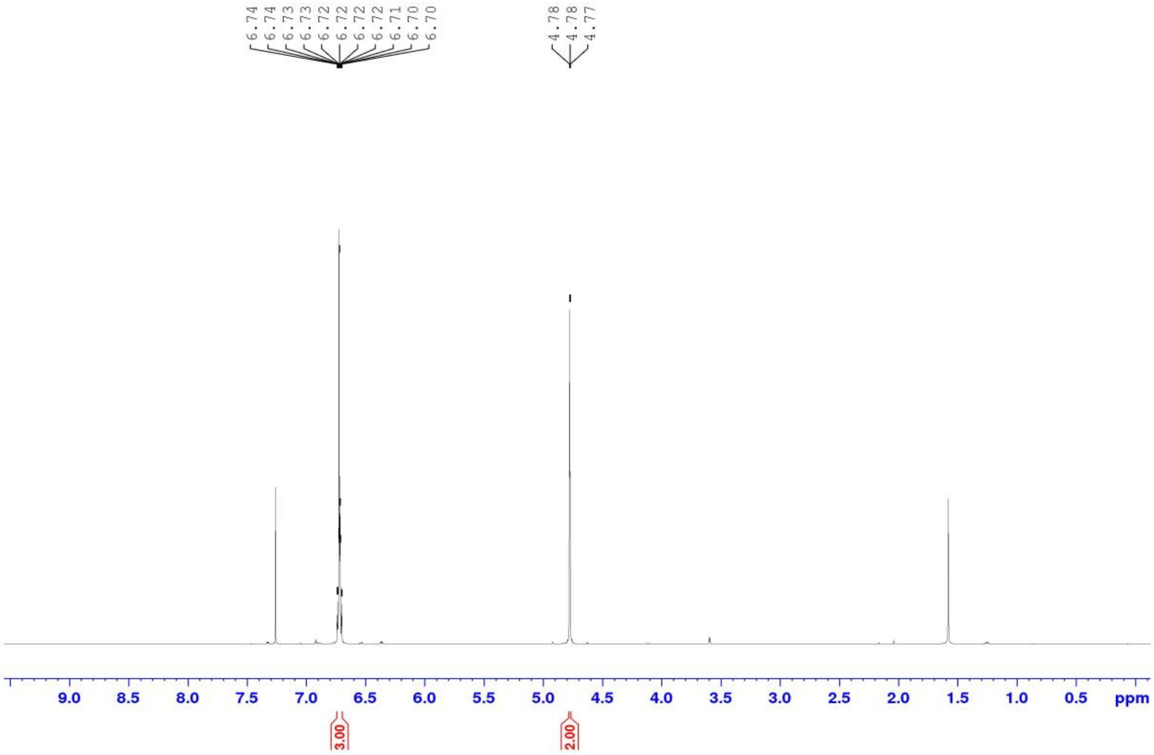

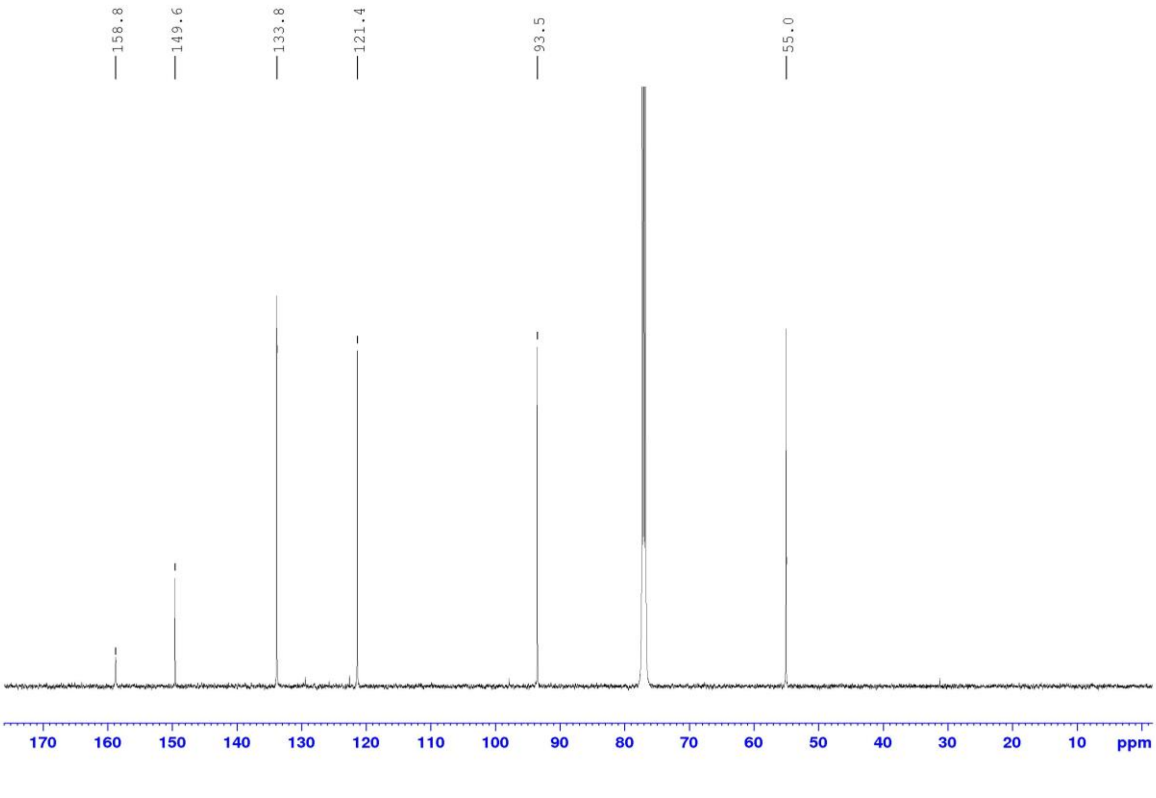

2-Nitro-6,7-dihydropyrazolo[1,5-*a*]pyridine, **10b**

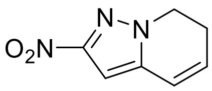

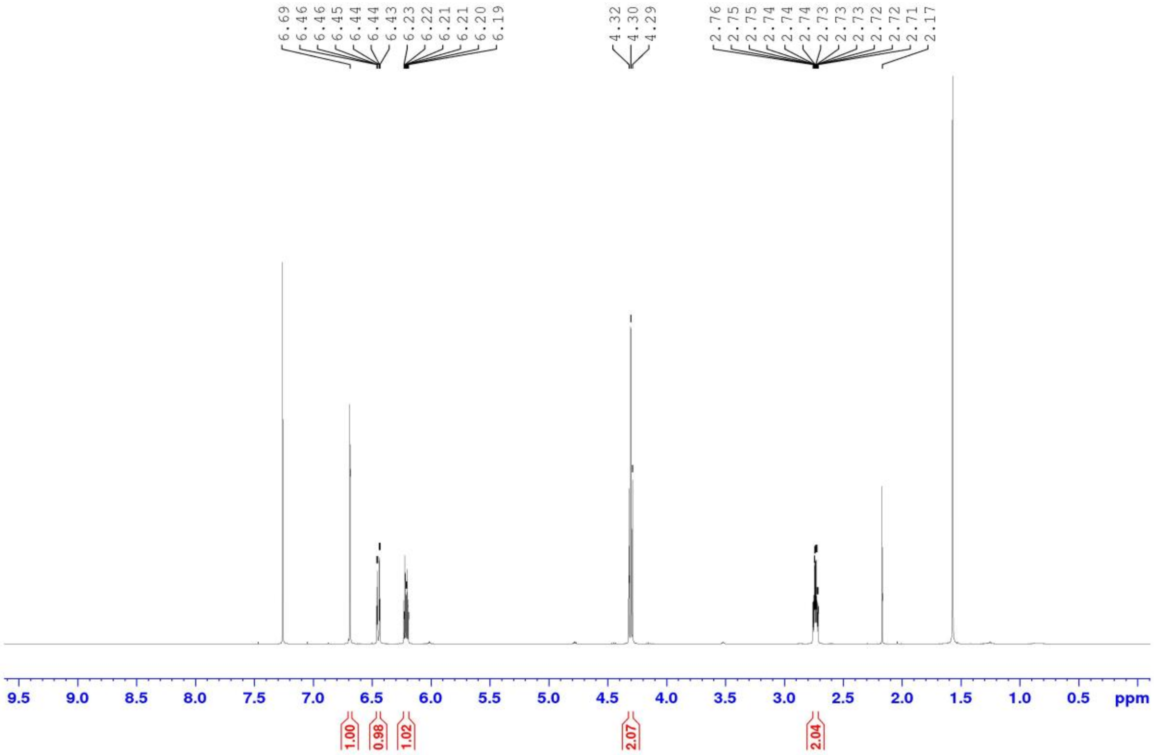

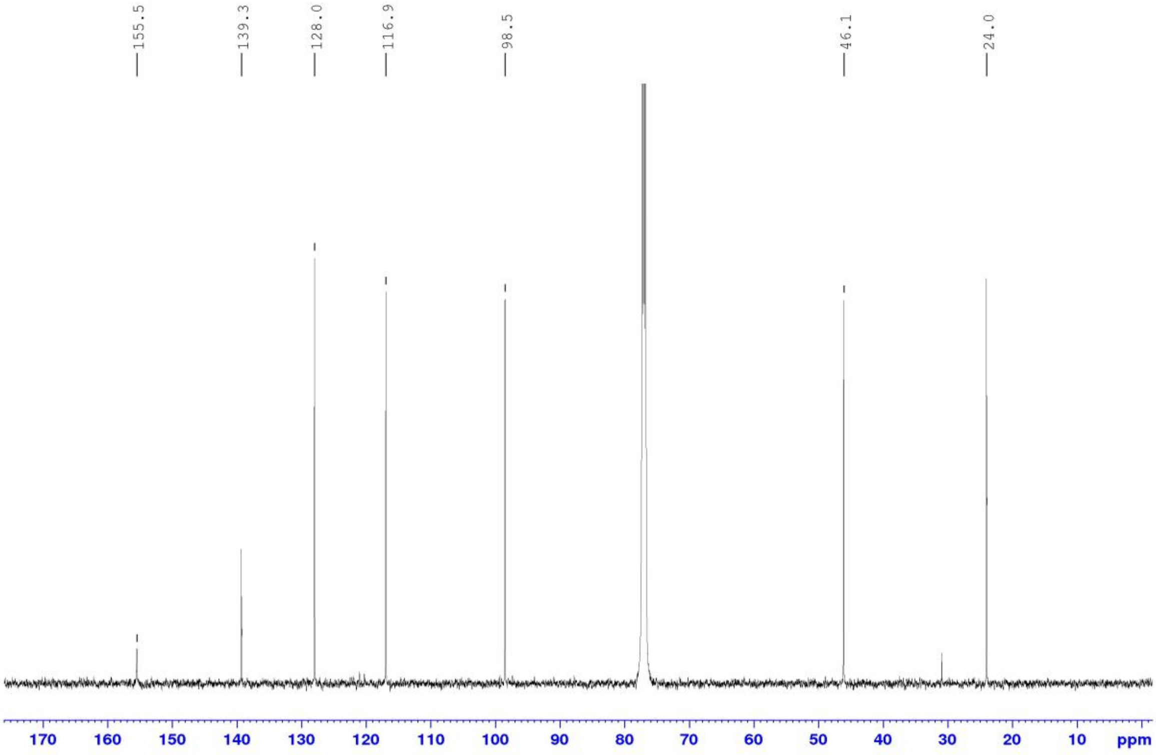

2-Nitro-7,8-dihydro-6*H*-pyrazolo[1,5-*a*]azepine, **10c**

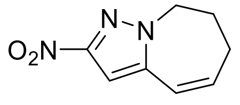

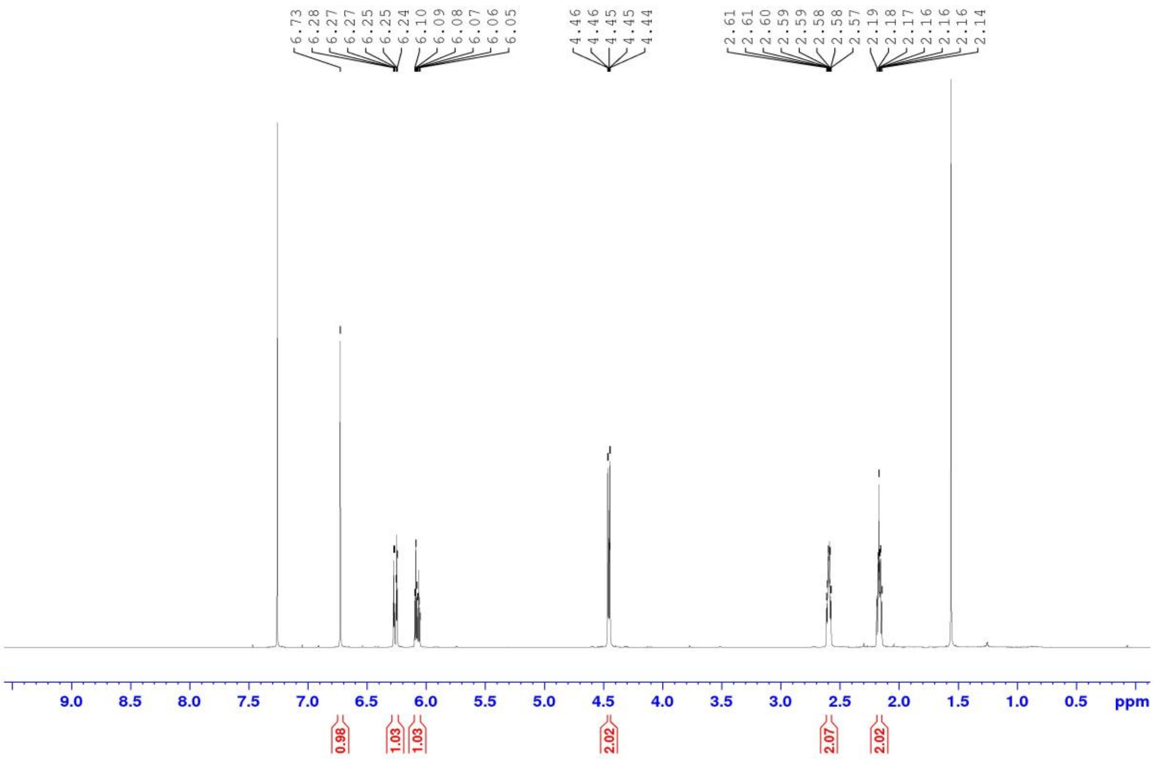

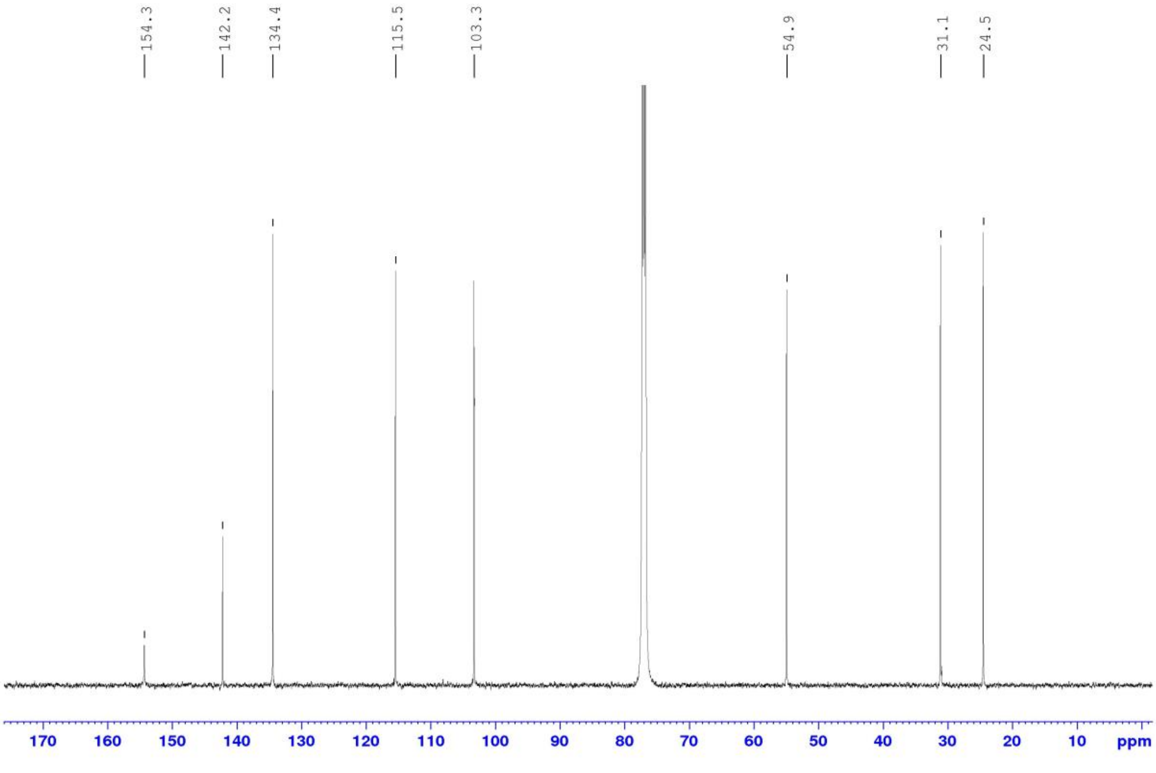

(*Z*)-2-Nitro-8,9-dihydro-6*H*-pyrazolo[1,5-d][1,4]oxazocine, **10d**

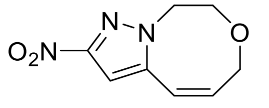

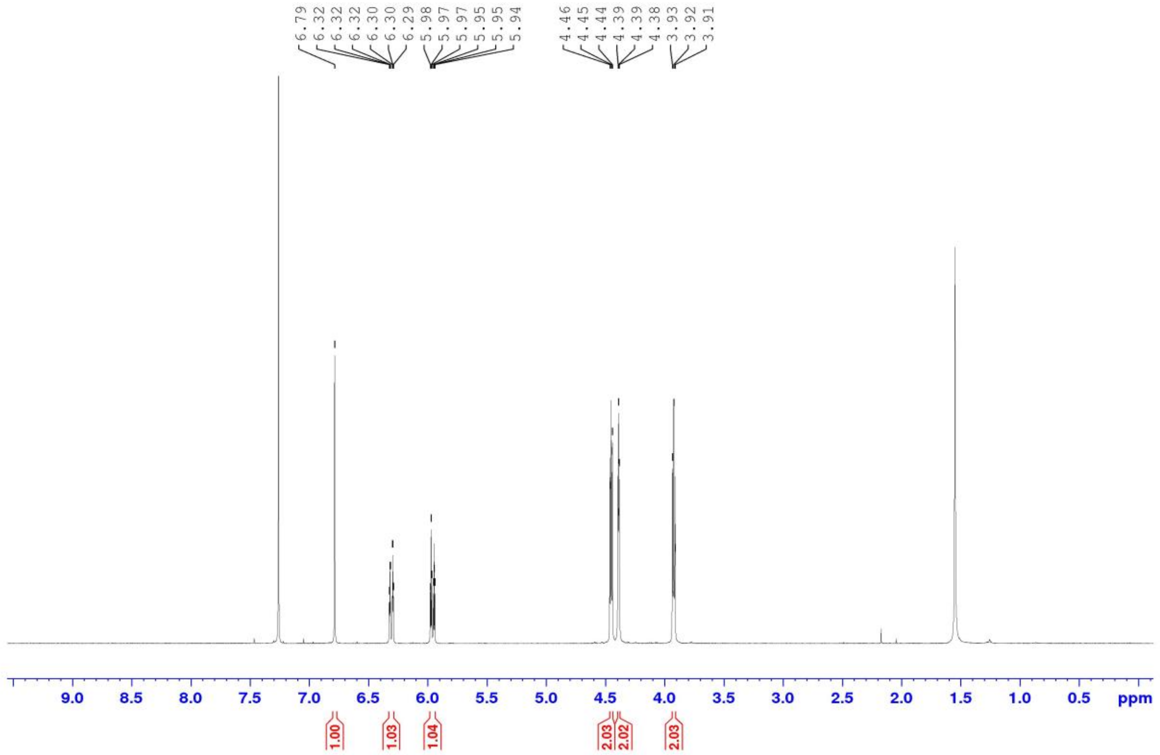

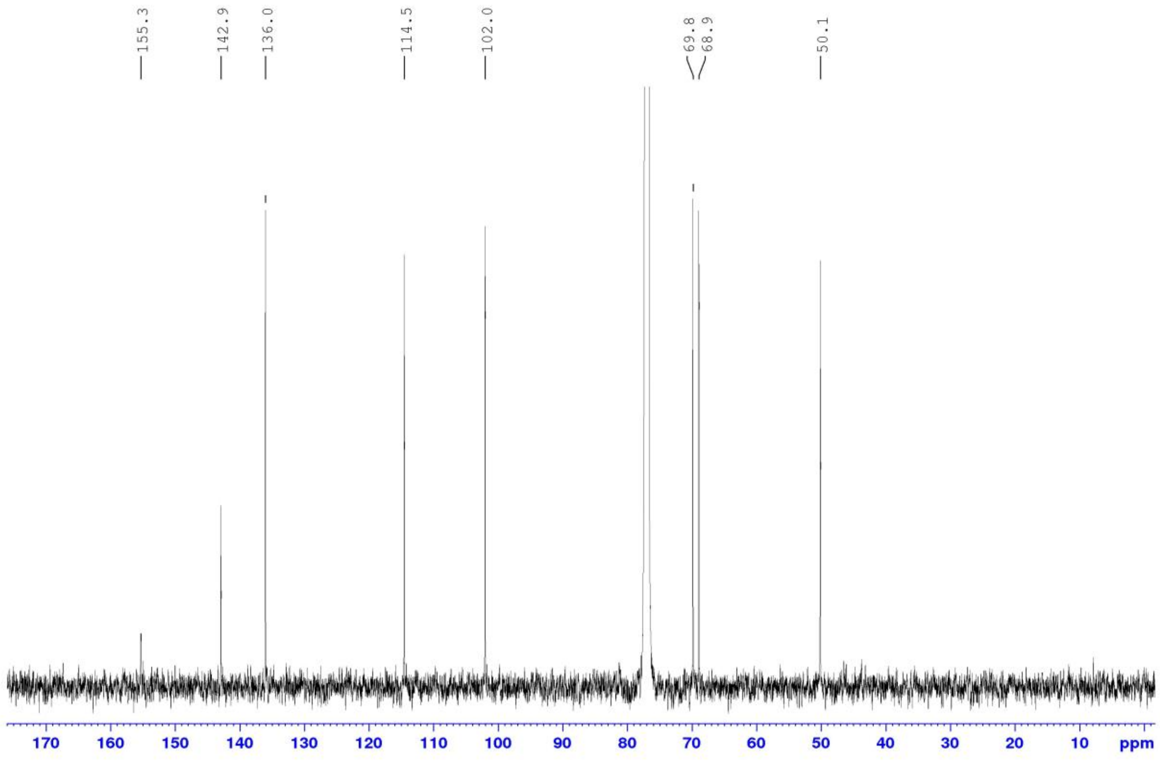

tert-Butyl (*Z*)-2-nitro-8,9-dihydropyrazolo[1,5-*d*][1,4]diazocine-7(6*H*)-carboxylate, **10e**

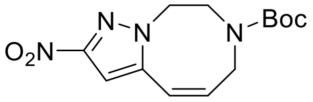

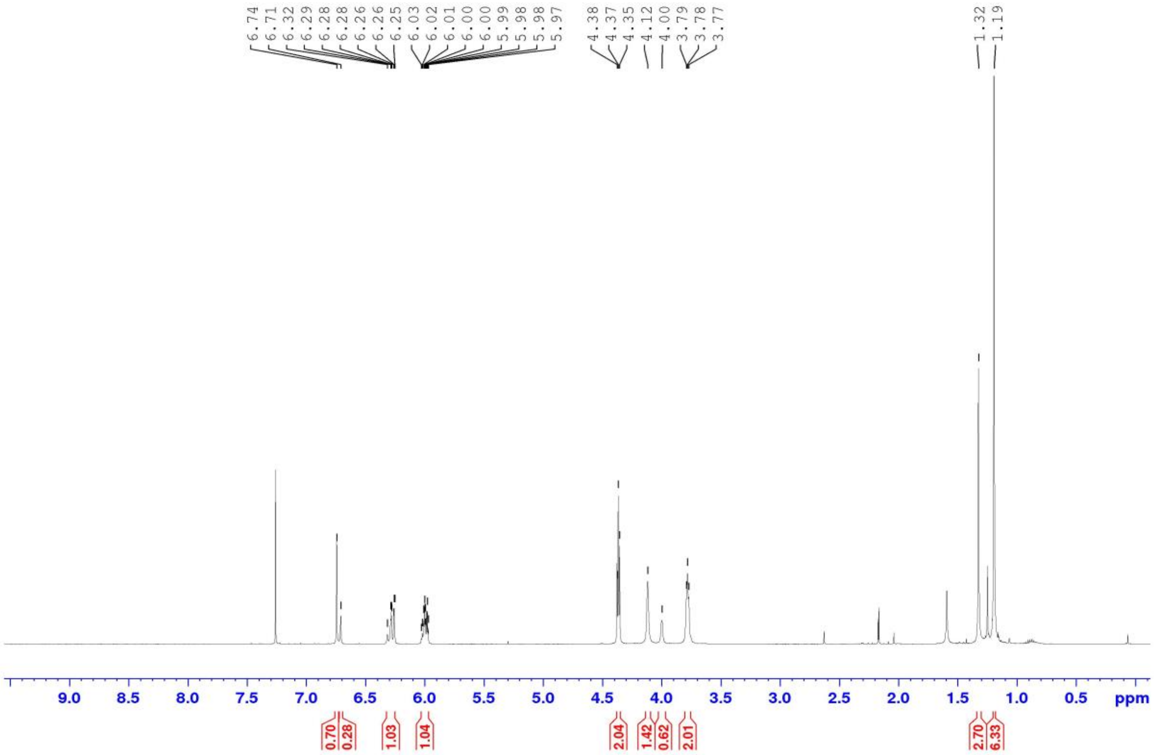

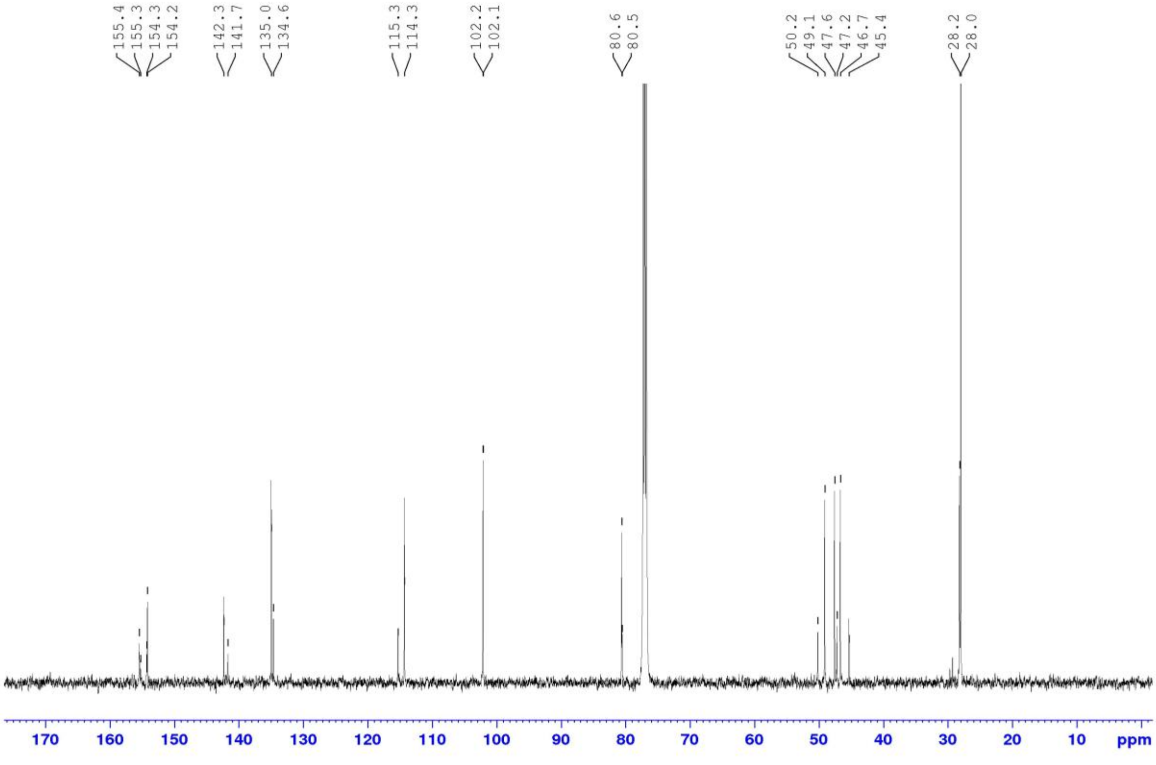

4-Methyl-2-nitro-6,7-dihydropyrazolo[1,5-*a*]pyridine, **10f**

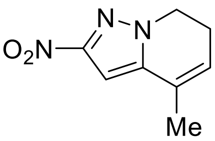

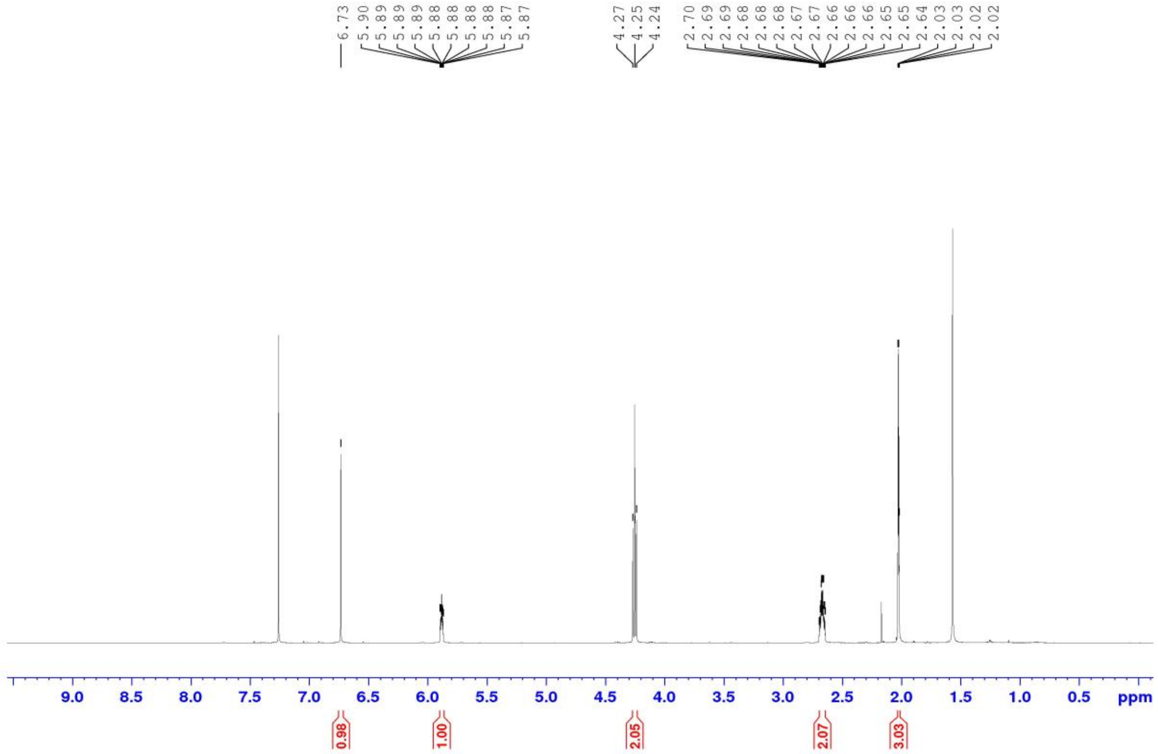

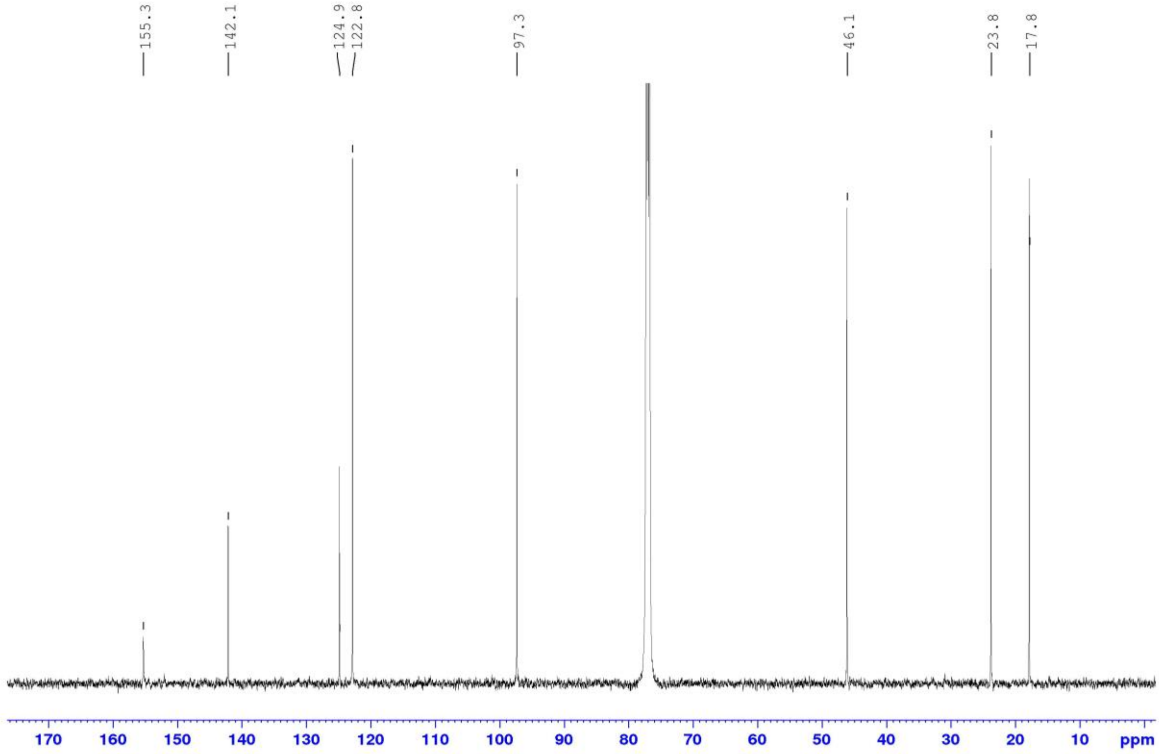

2-Nitro-4,7-dihydropyrazolo[1,5-*a*]pyridine, **10g**

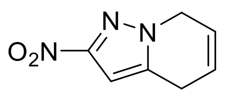

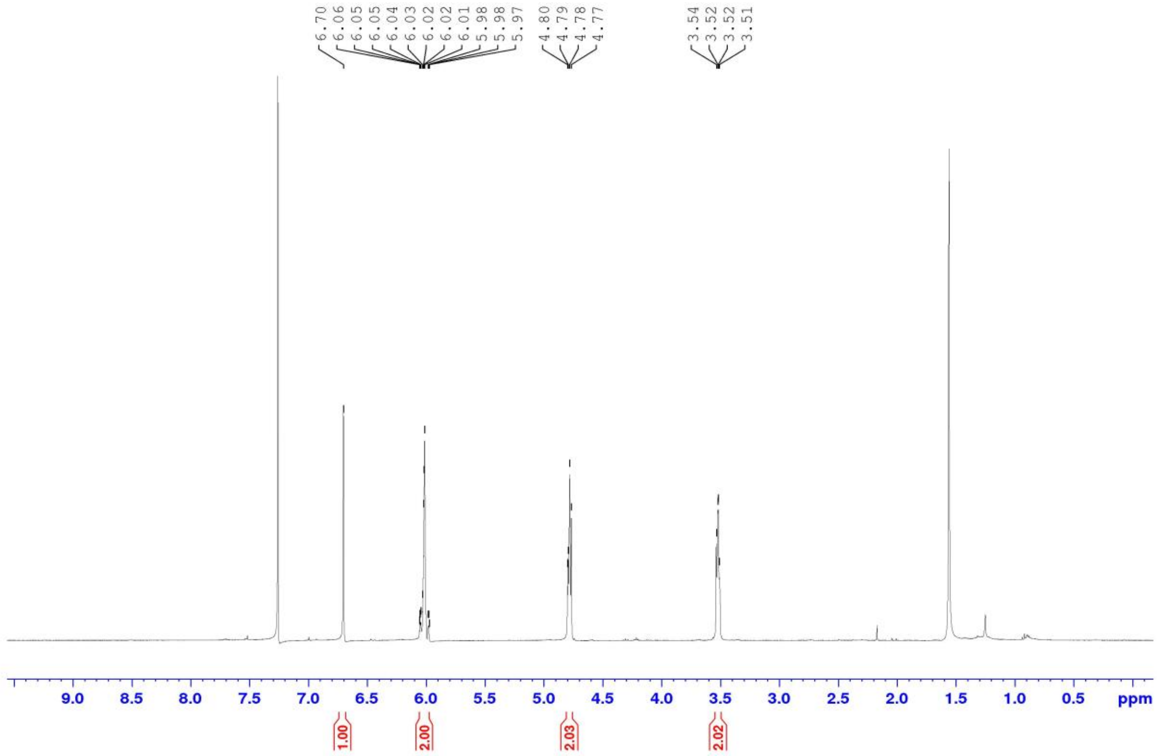

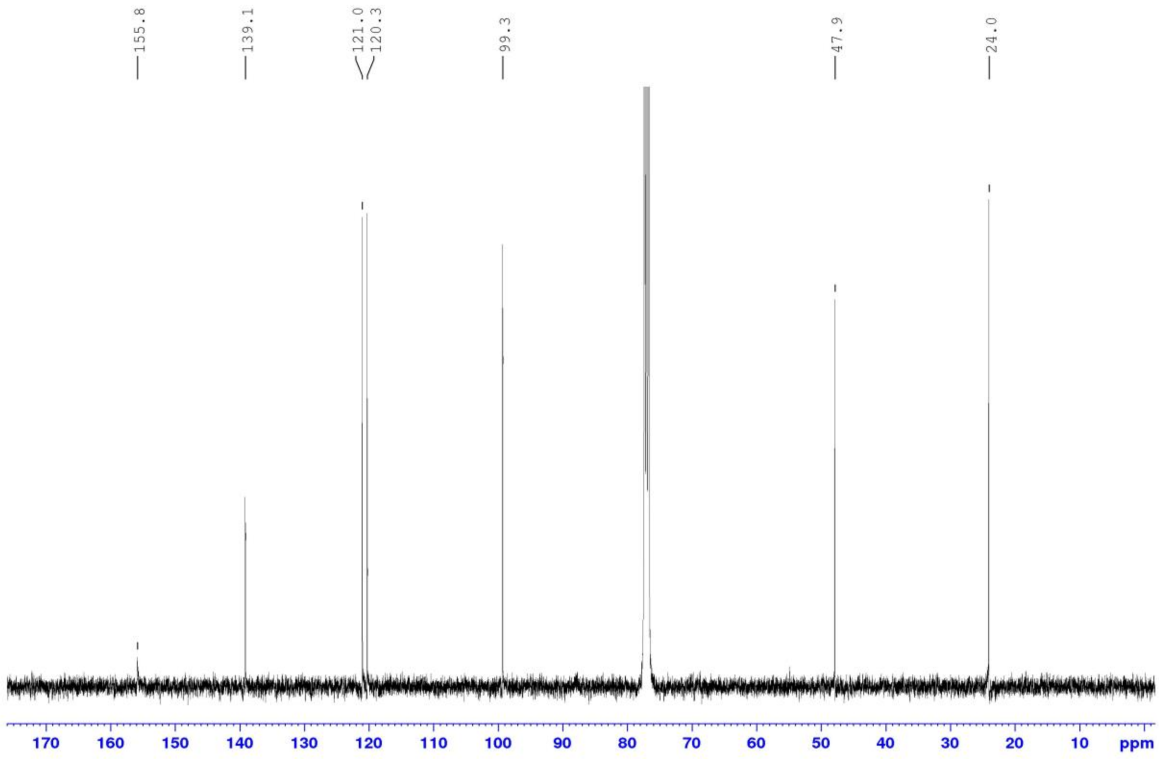

2-Nitro-7,8-dihydro-4*H*-pyrazolo[1,5-*a*]azepine, **10h**

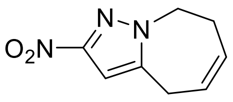

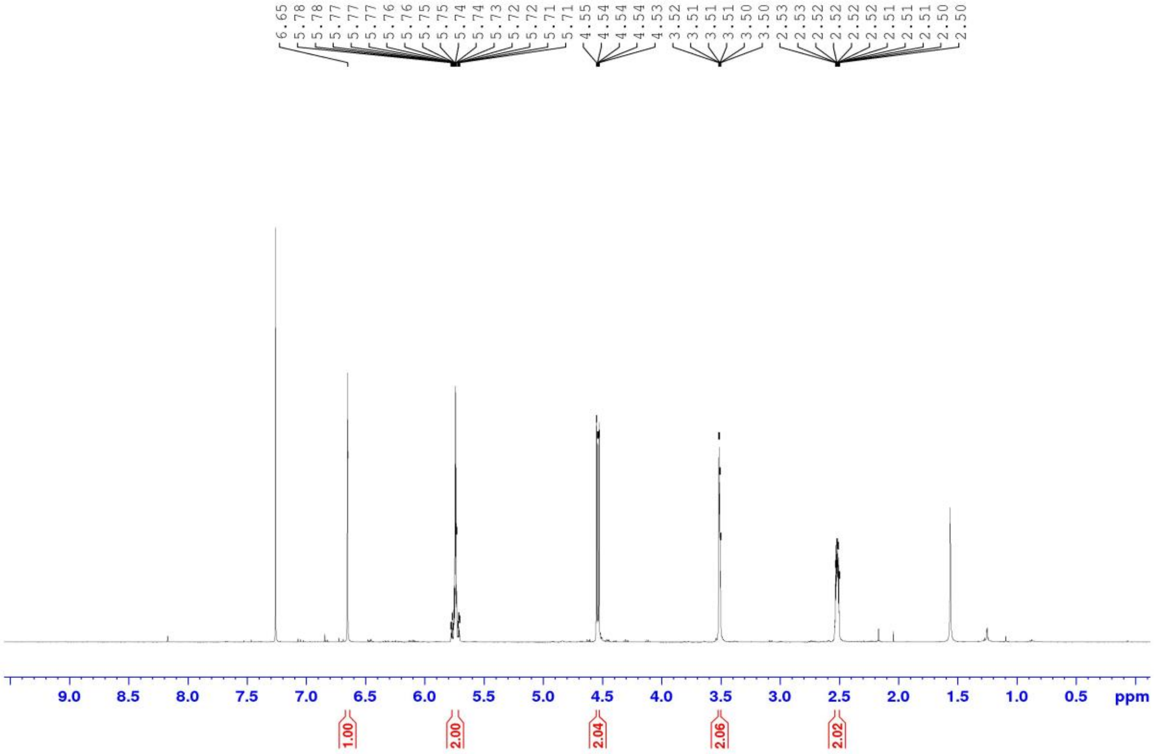

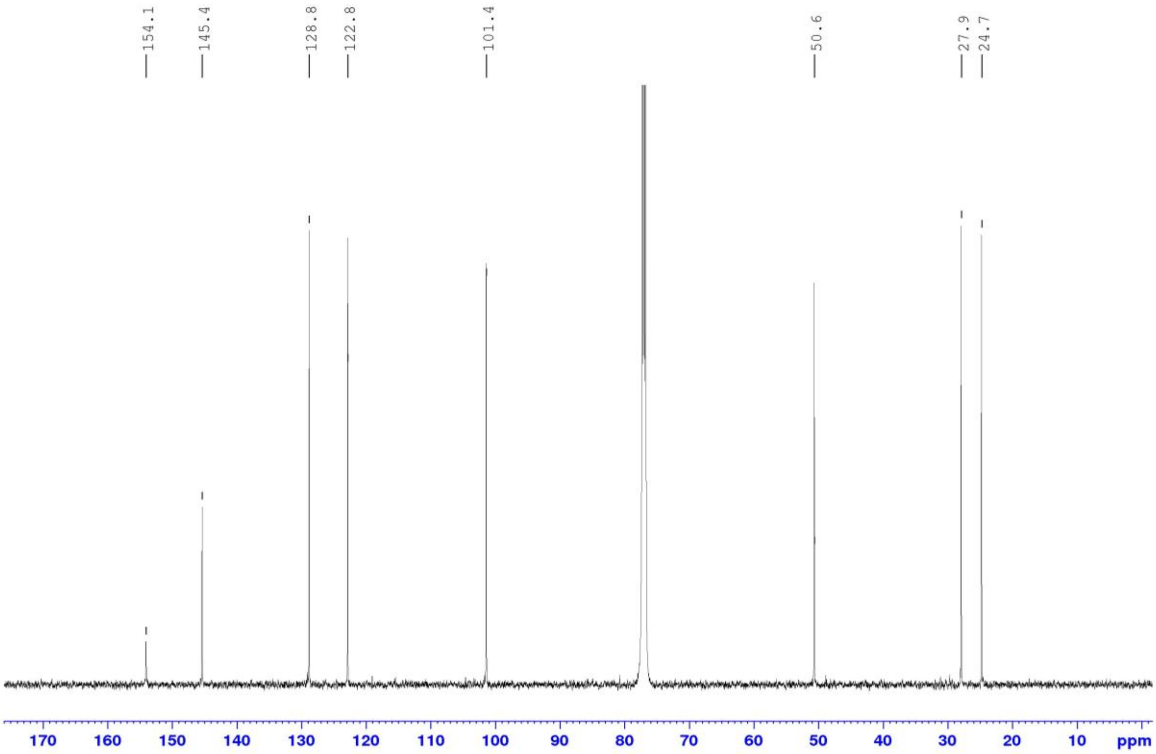

N,N-(bis-Boc)-5-bromo-4-methylpyridin-2-amine, **14**

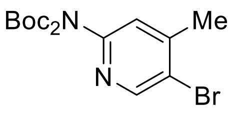

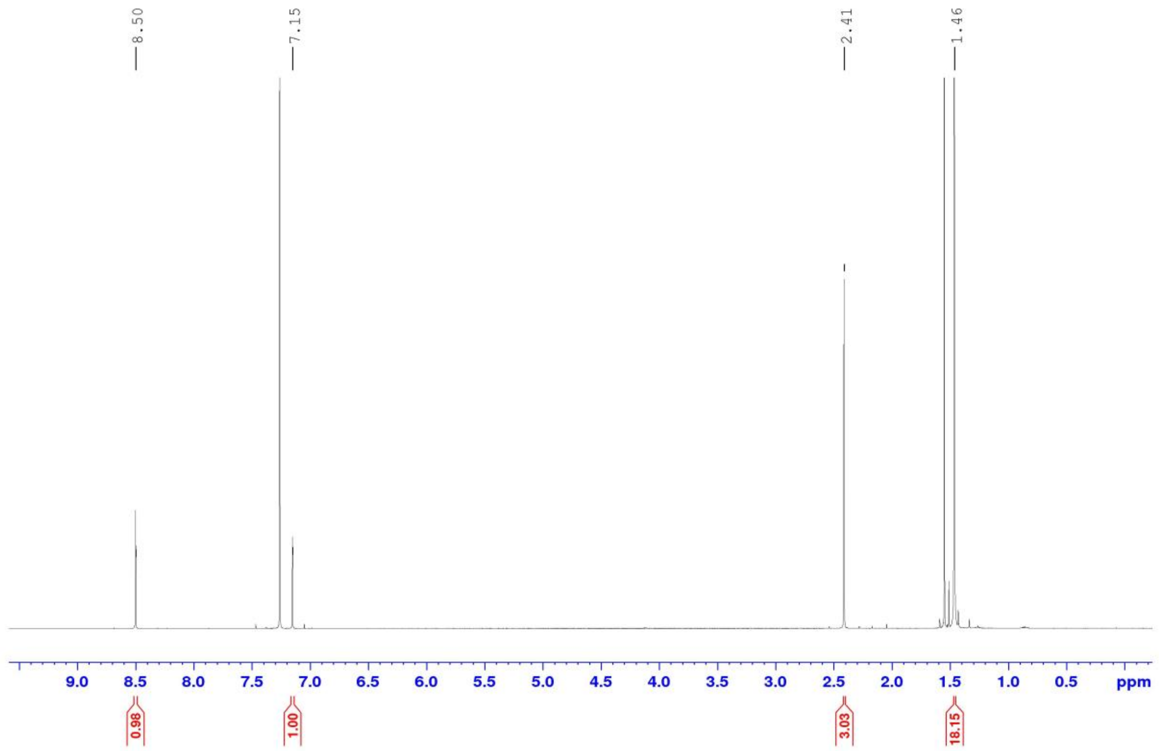

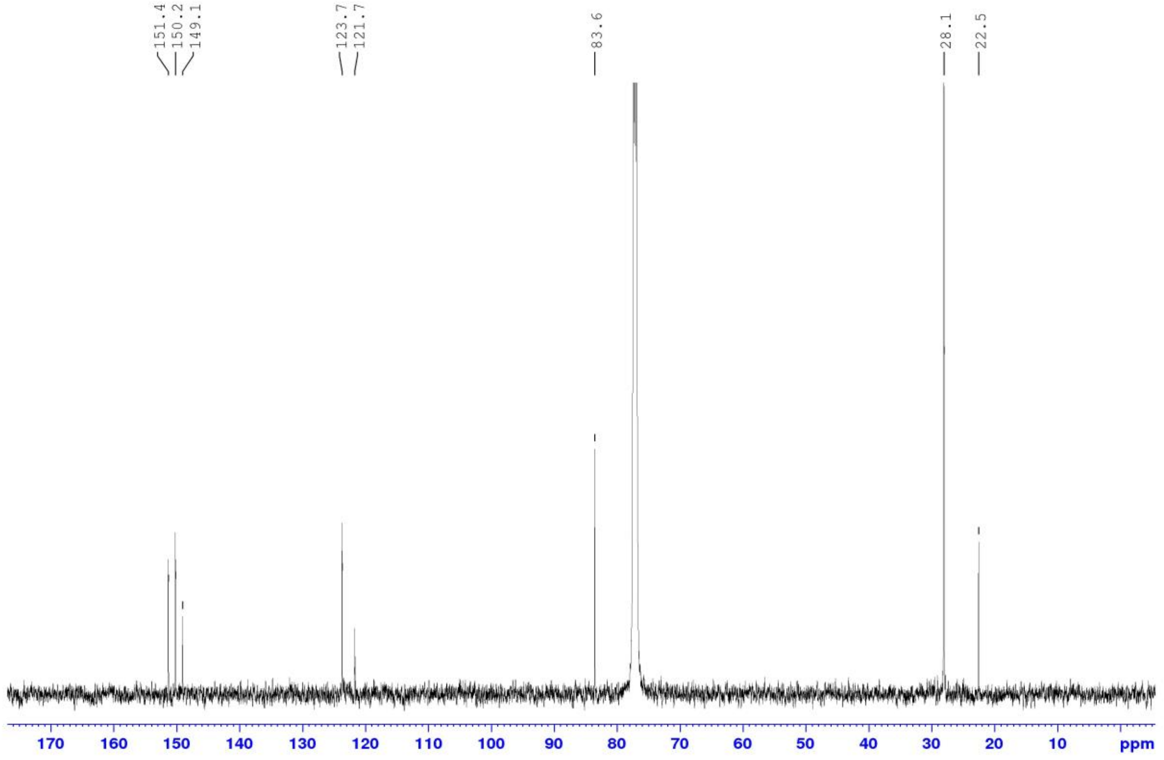

N,N-(bis-Boc)-4-methyl-5-vinylpyridin-2-amine, **15a**

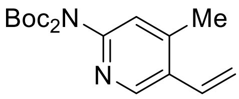

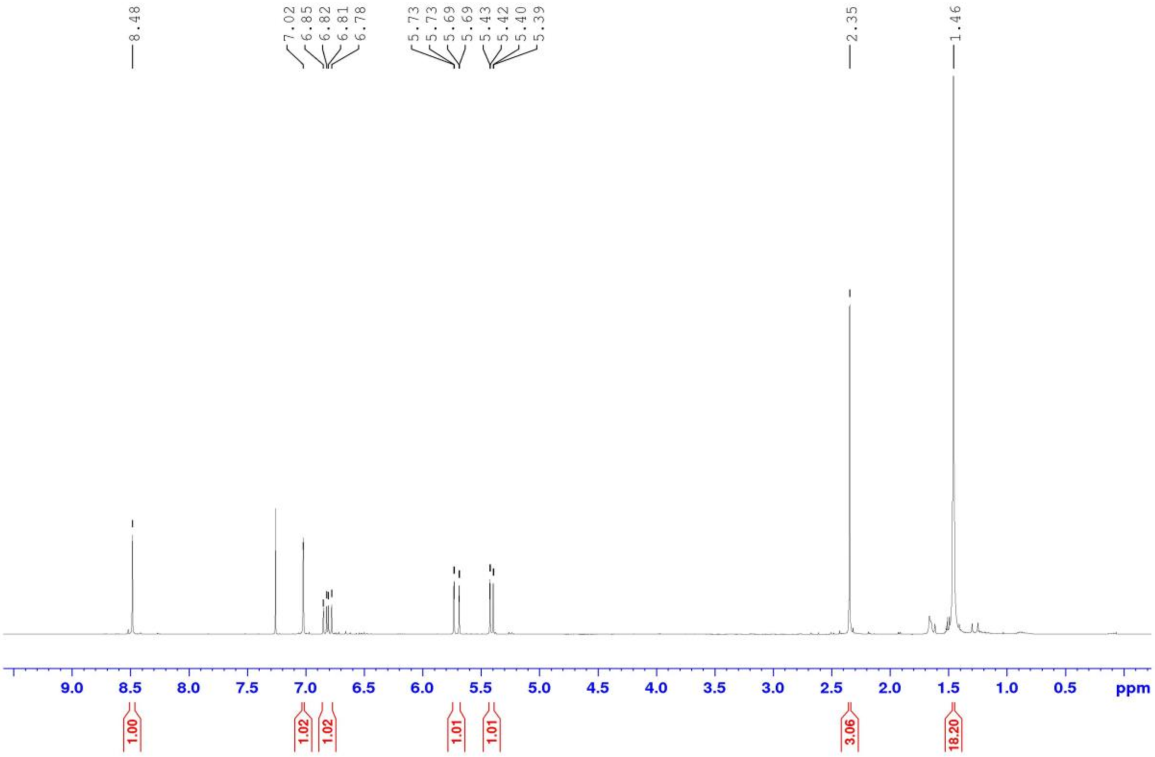

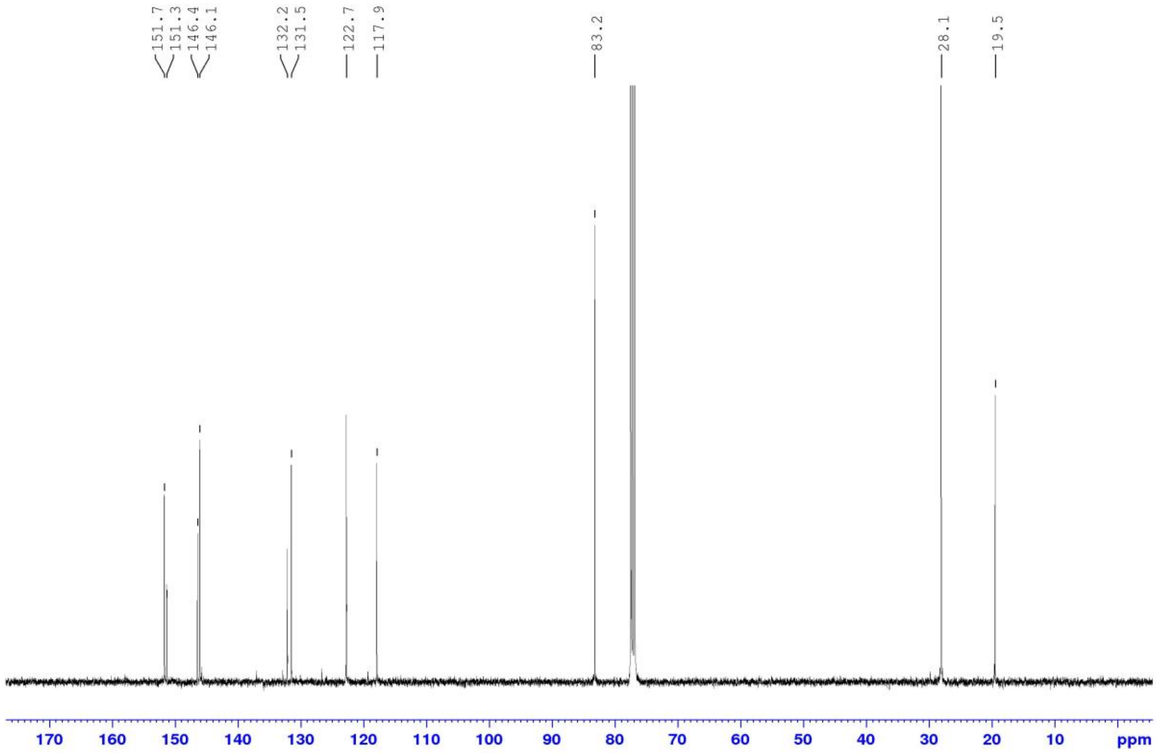

N,N-(bis-Boc)-4-methyl-5-(prop-1-en-2-yl)pyridin-2-amine, **15d**

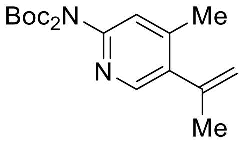

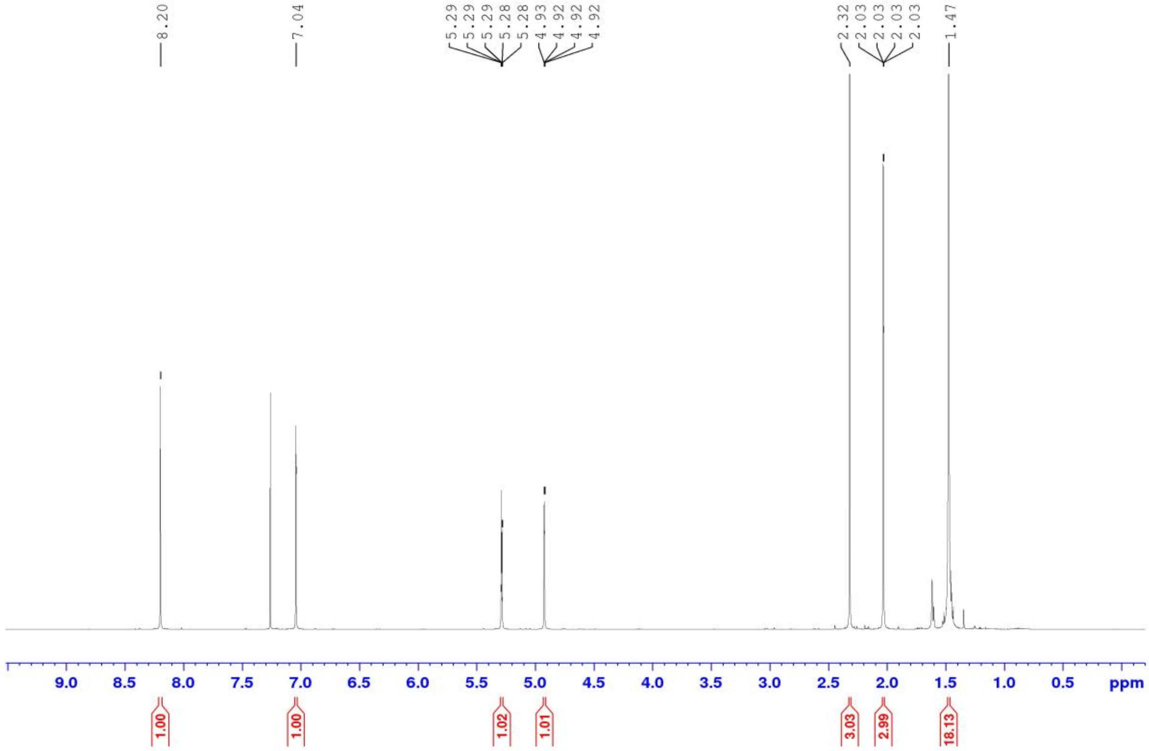

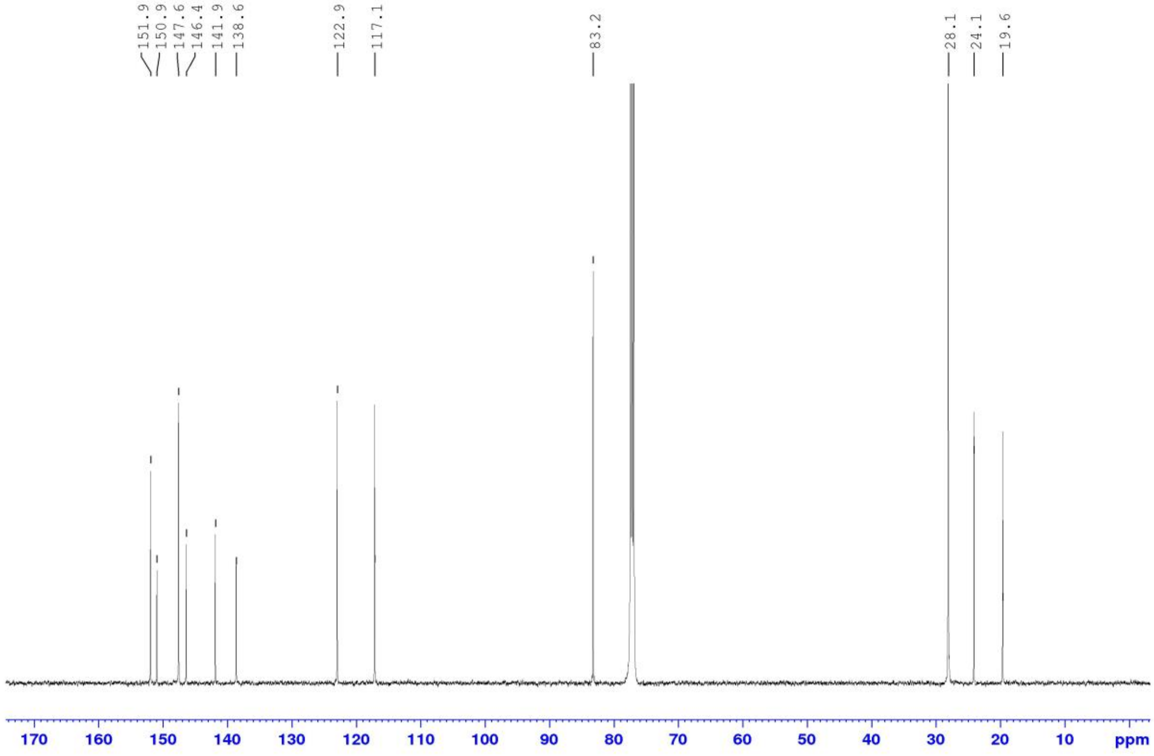

N,N-(bis-Boc)-4-methyl-5-(1-phenylvinyl)pyridin-2-amine, **15e**

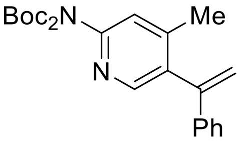

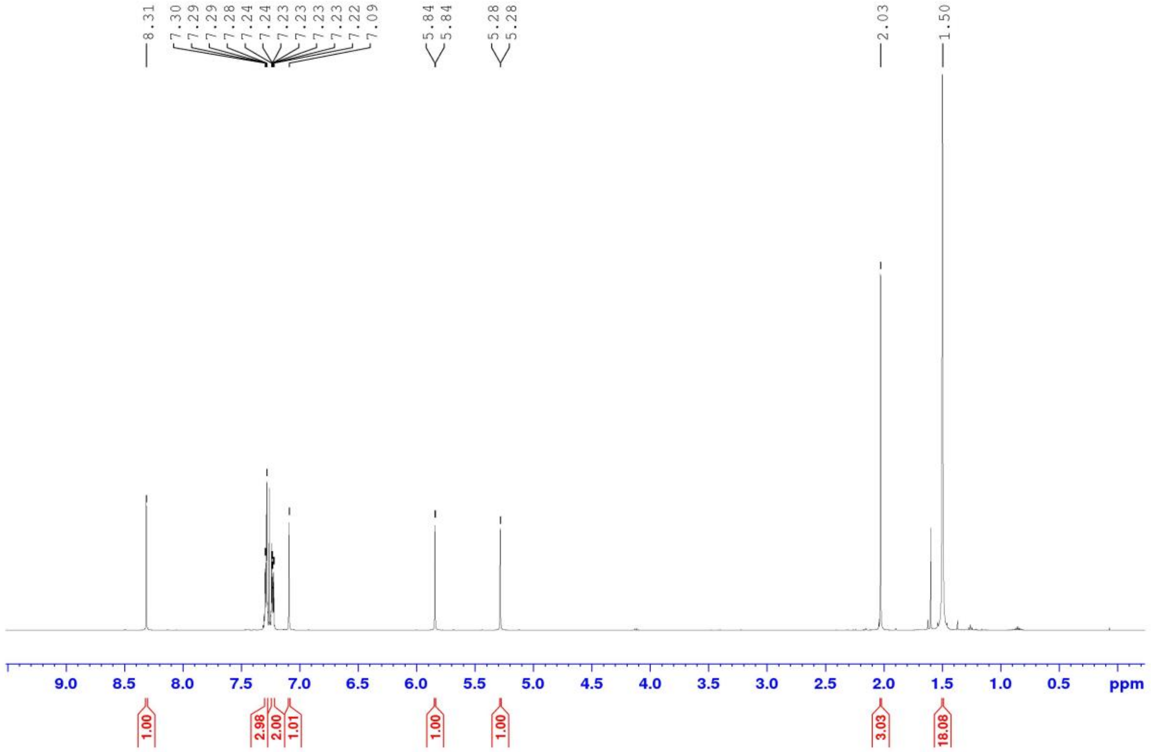

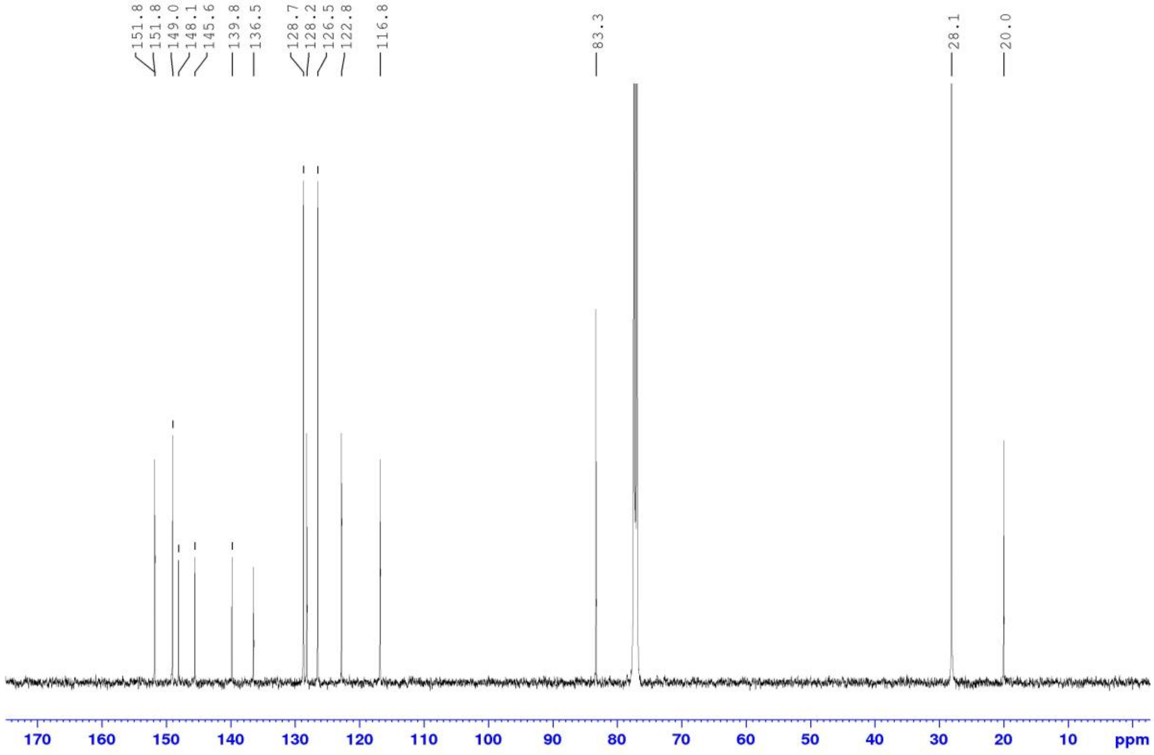

5-Allyl-*N,N*-(bis-Boc)-4-methylpyridin-2-amine, **15f**

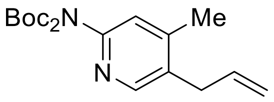

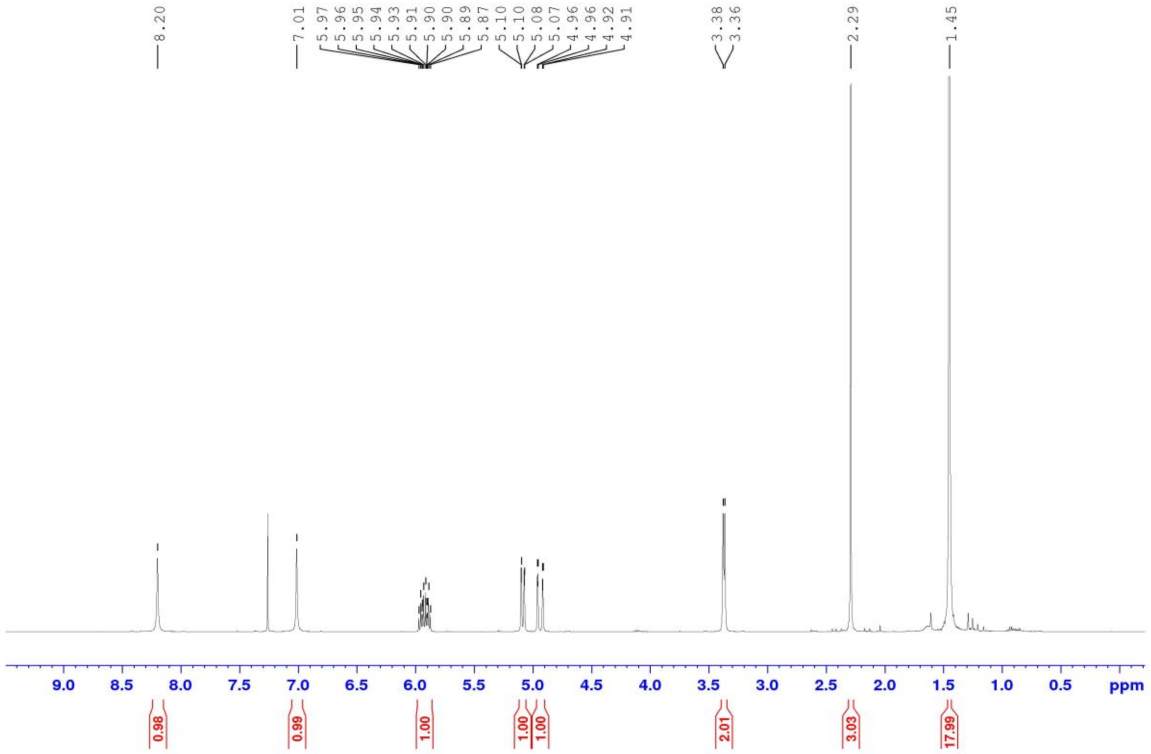

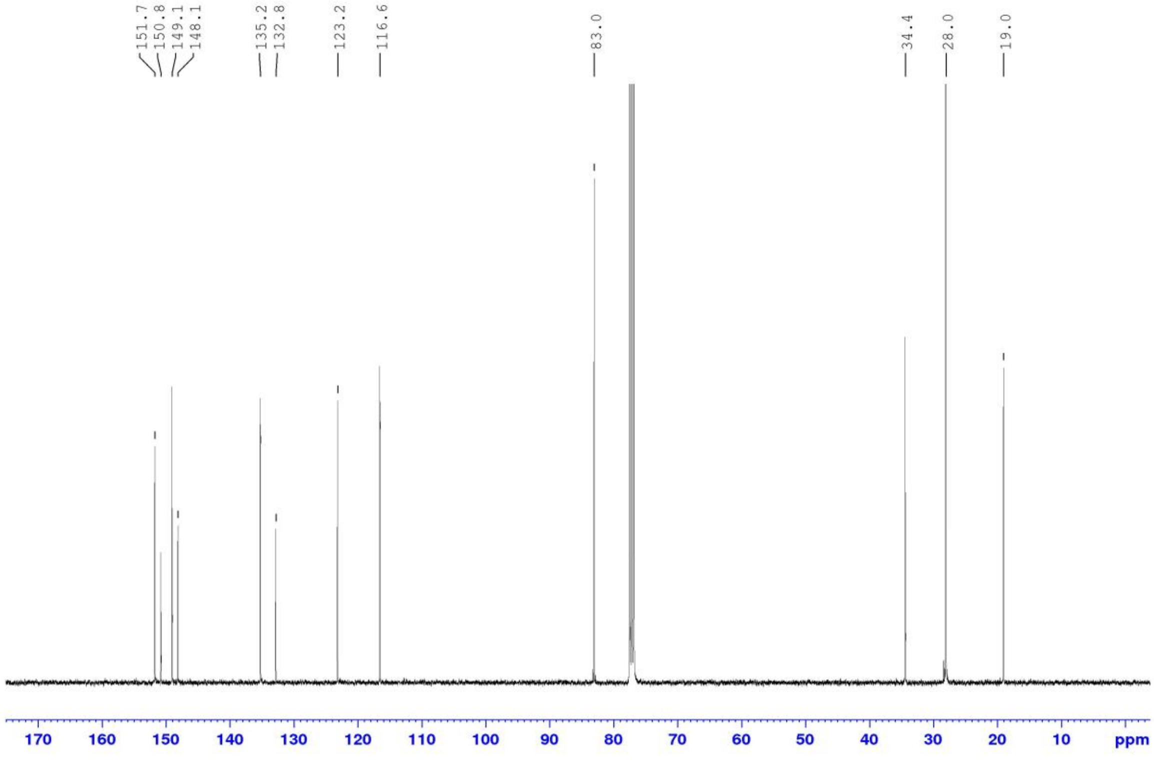

5-Allyl-2-chloro-4-methylpyridine, **15g**

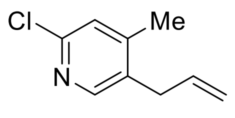

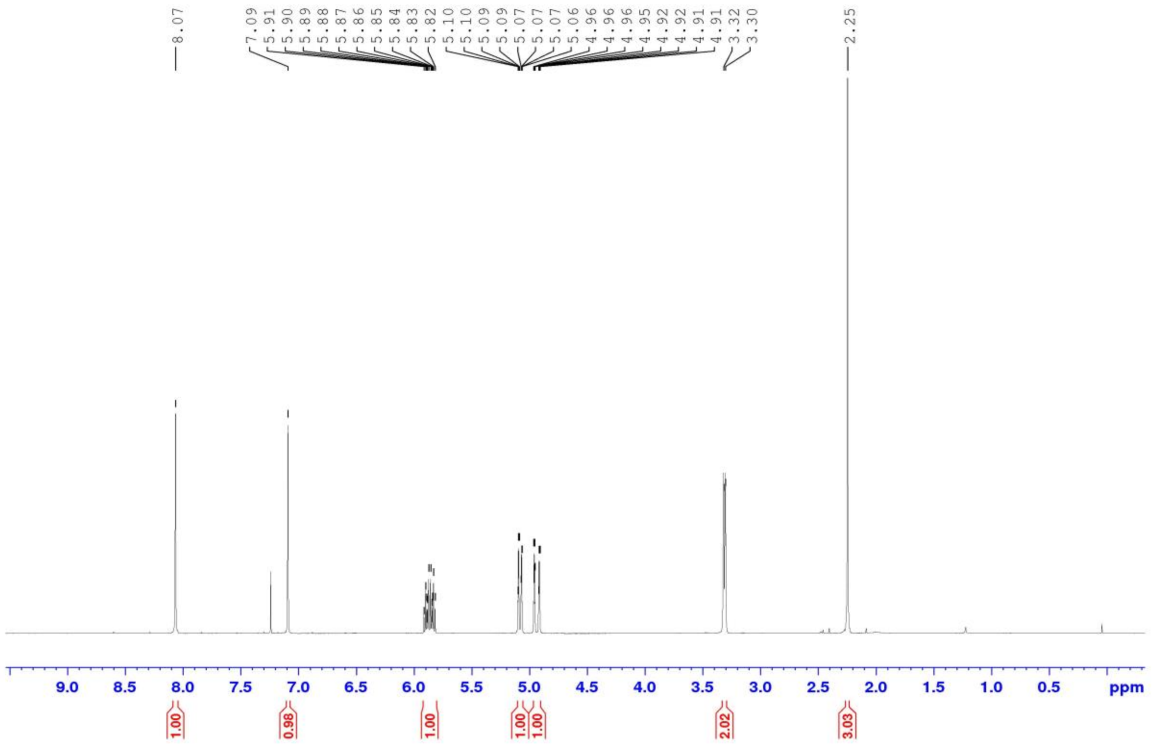

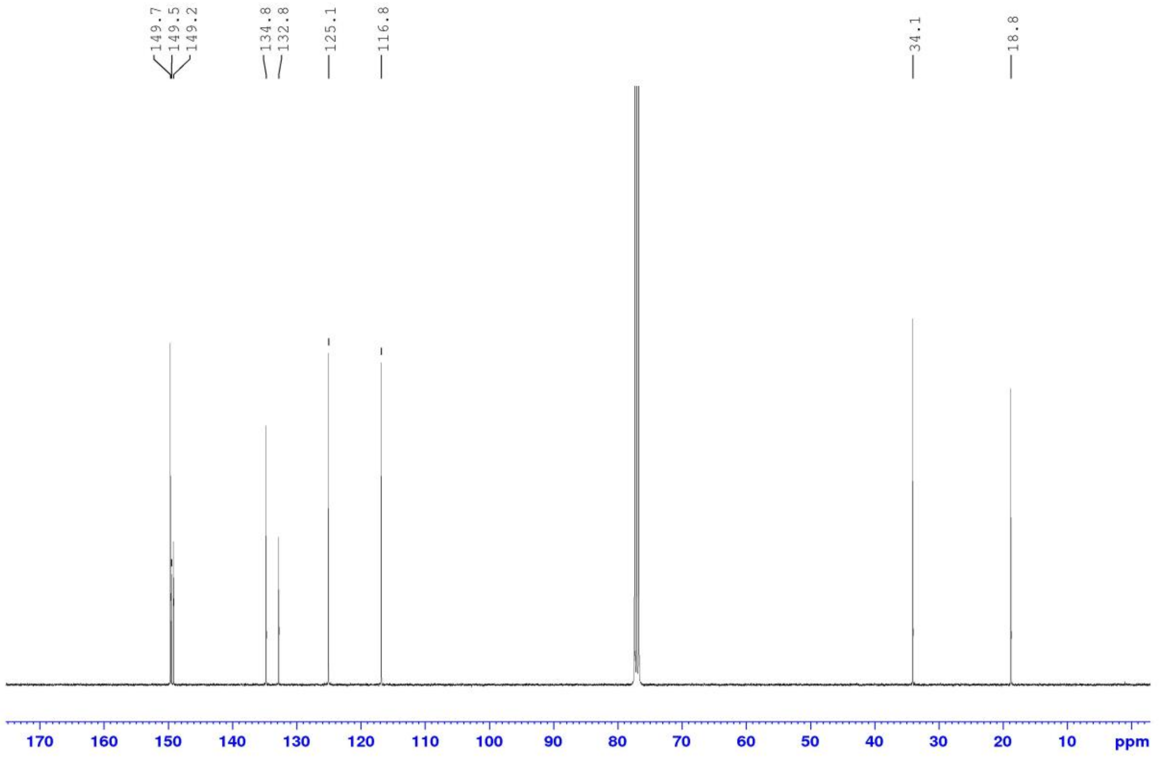

N,N-(bis-Boc)-4-(but-3-en-1-yl)-5-vinylpyridin-2-amine, **16a**

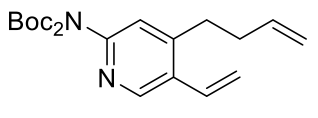

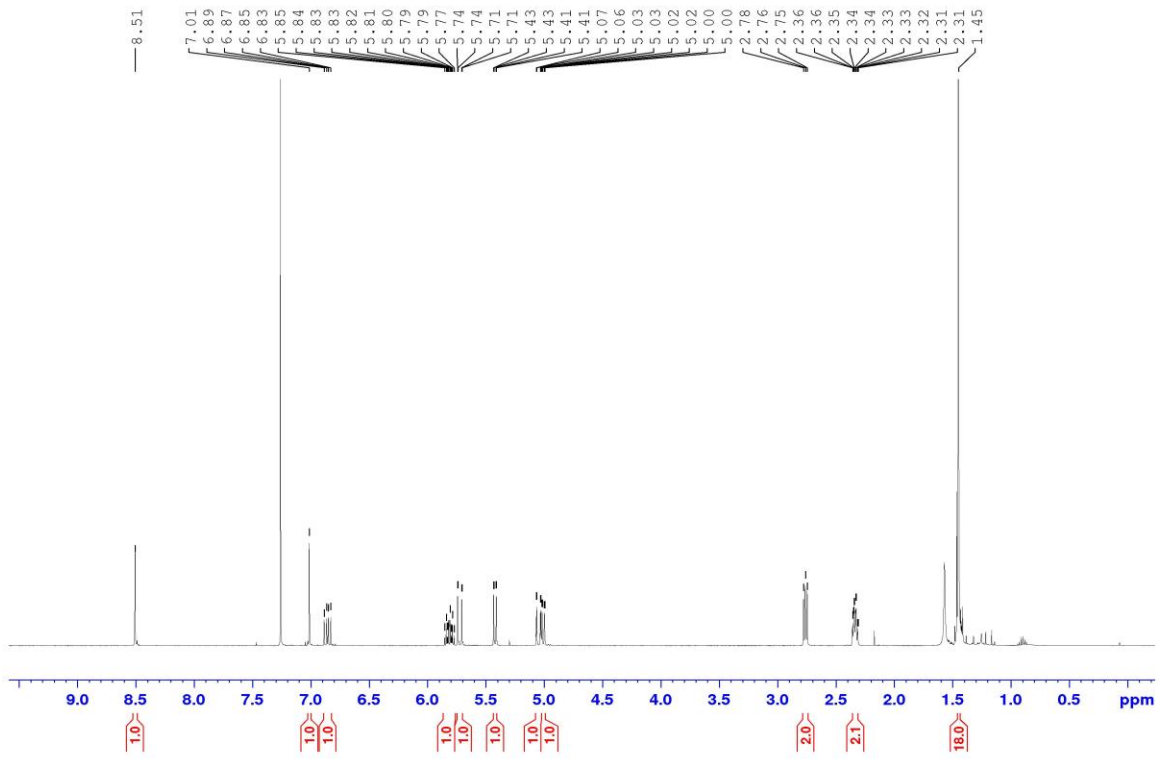

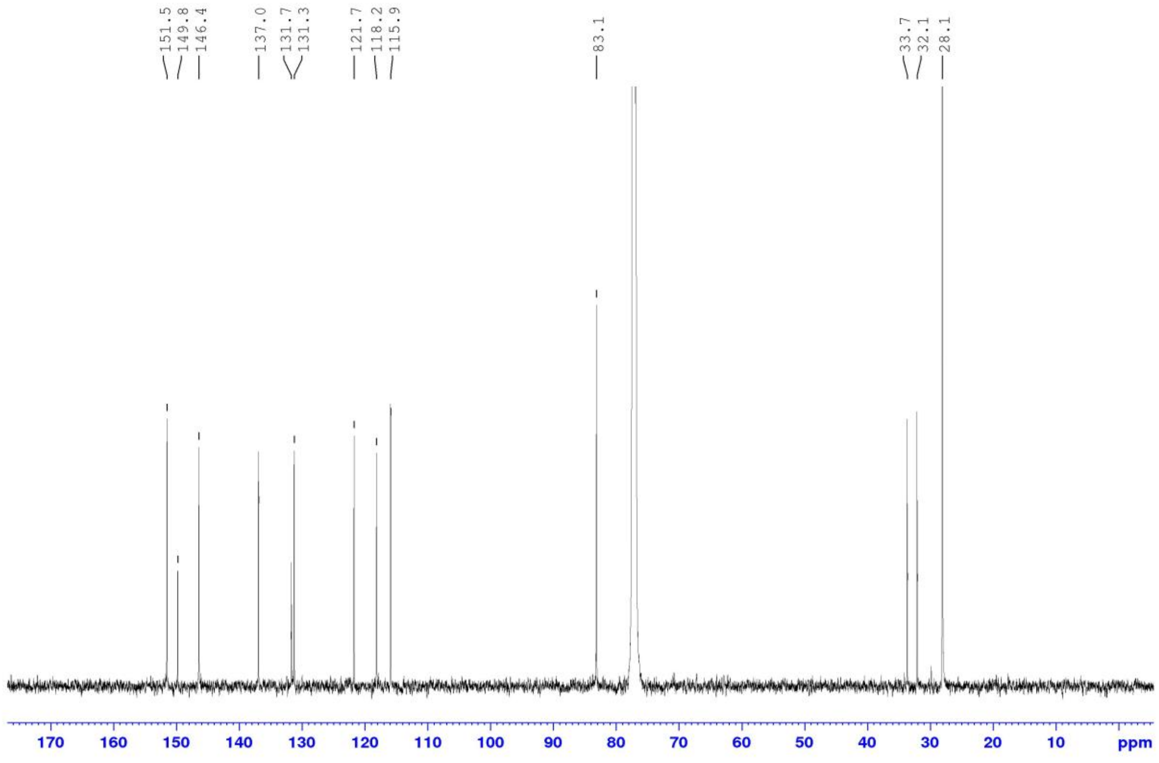

N,N-(bis-Boc)-4-(3-methylbut-3-en-1-yl)-5-vinylpyridin-2-amine, **16b**

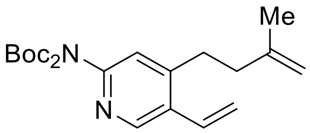

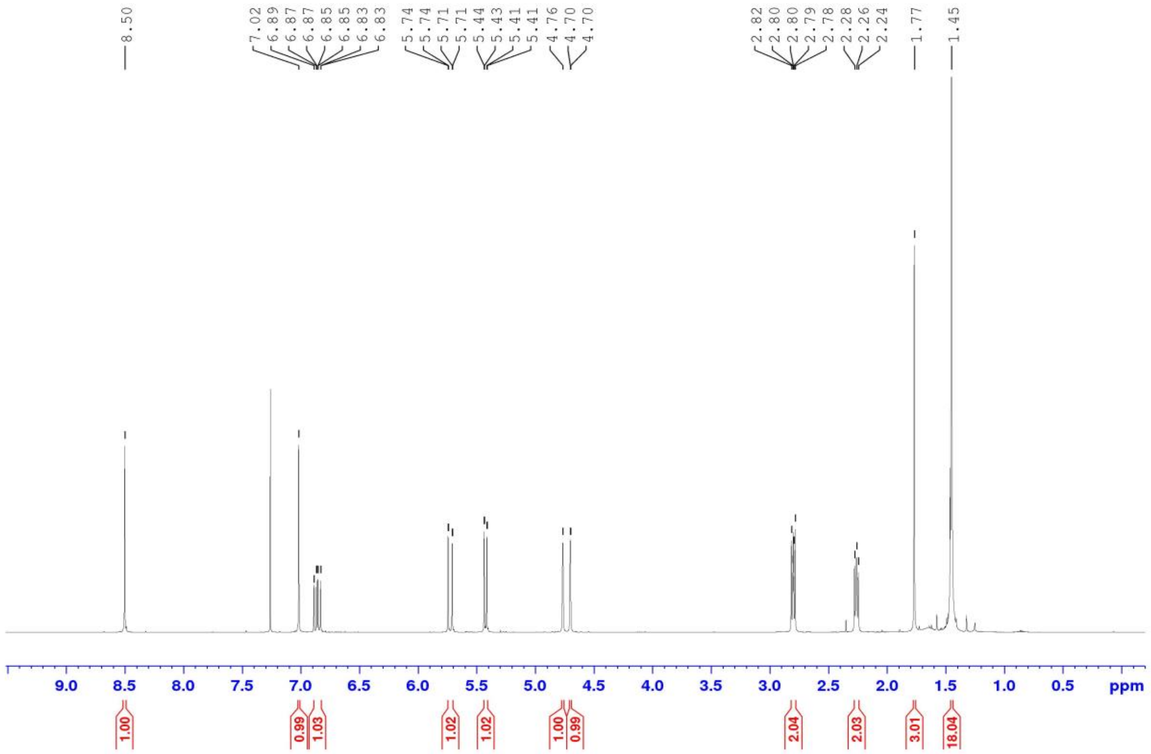

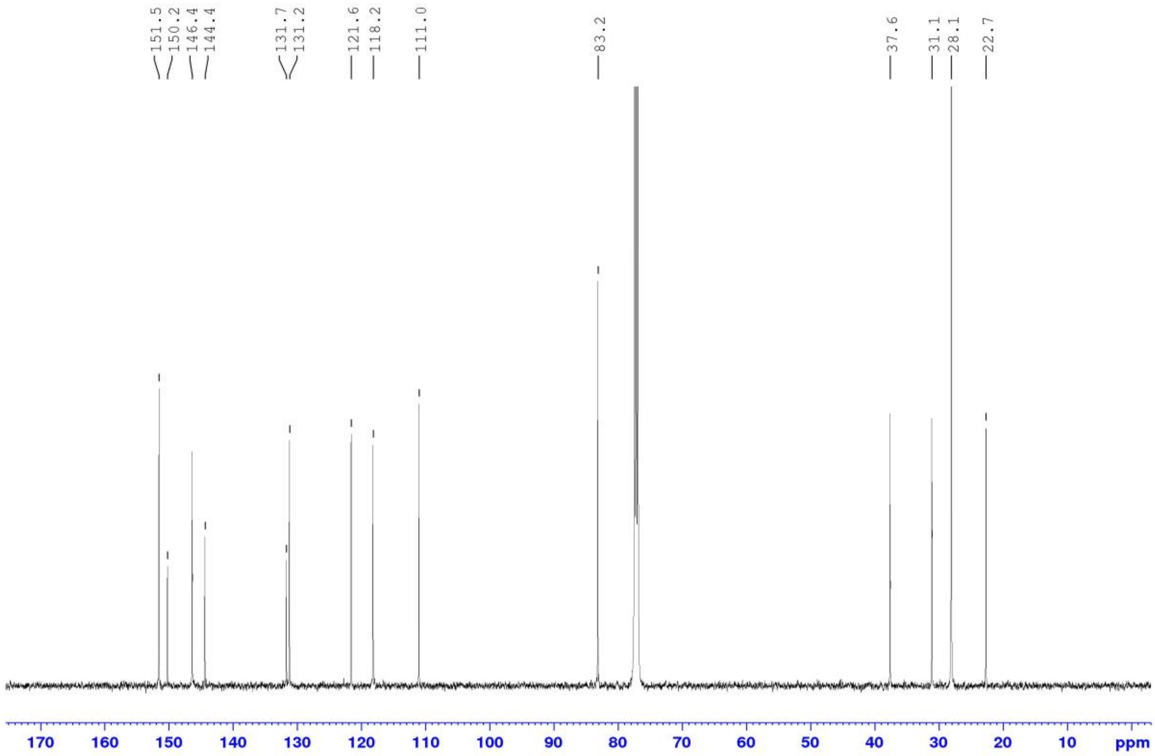

N,N-(bis-Boc)-4-(3-trifluoromethylbut-3-en-1-yl)-5-vinylpyridin-2-amine, **16c**

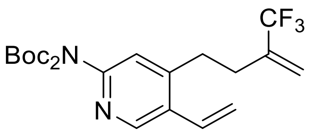

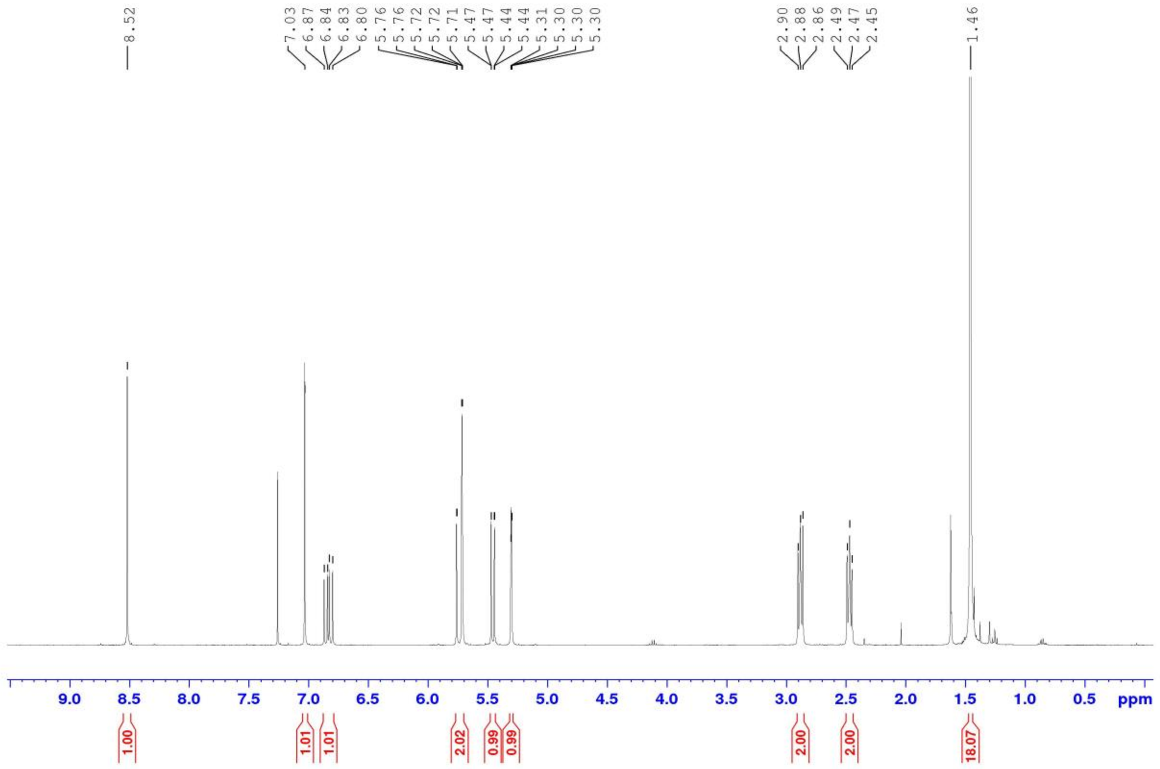

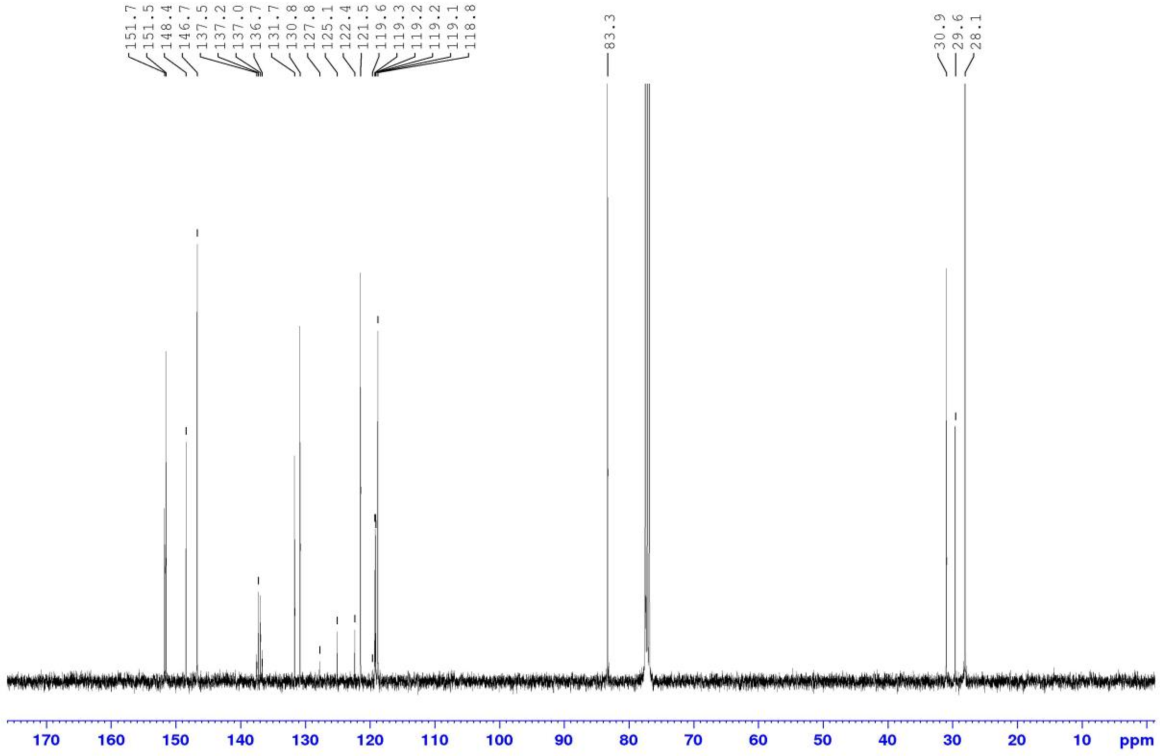

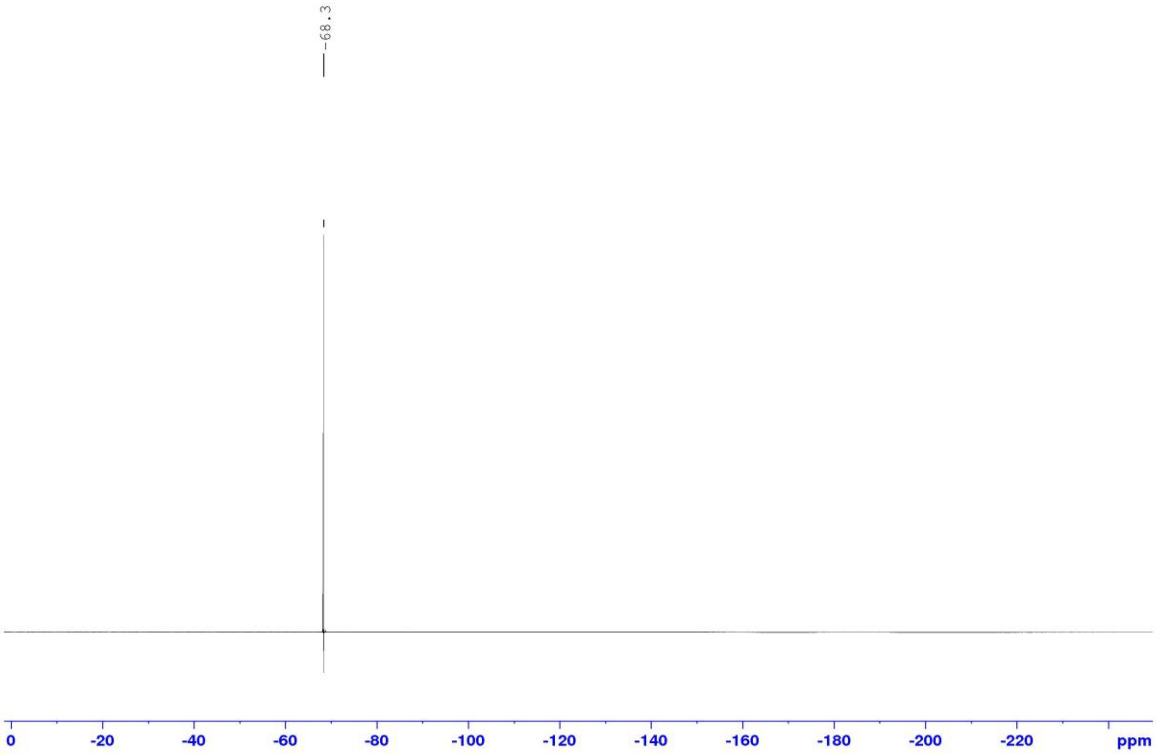
-68.3
0
-20
-40
-60
-80
-100
-120
-140
-160
-180
-200
-220
ppm

N,N-(bis-Boc)-4-(but-3-en-1-yl)-5-(prop-1-en-2-yl)pyridin-2-amine, **16d**

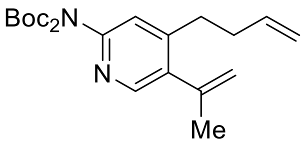

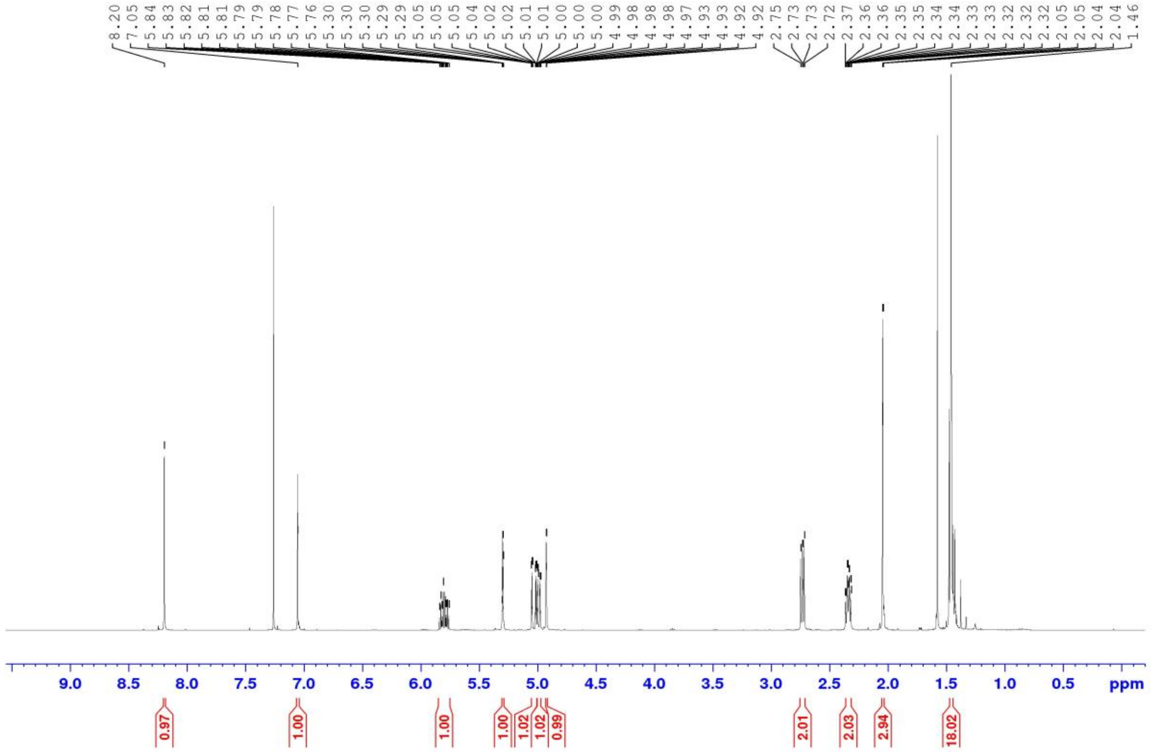

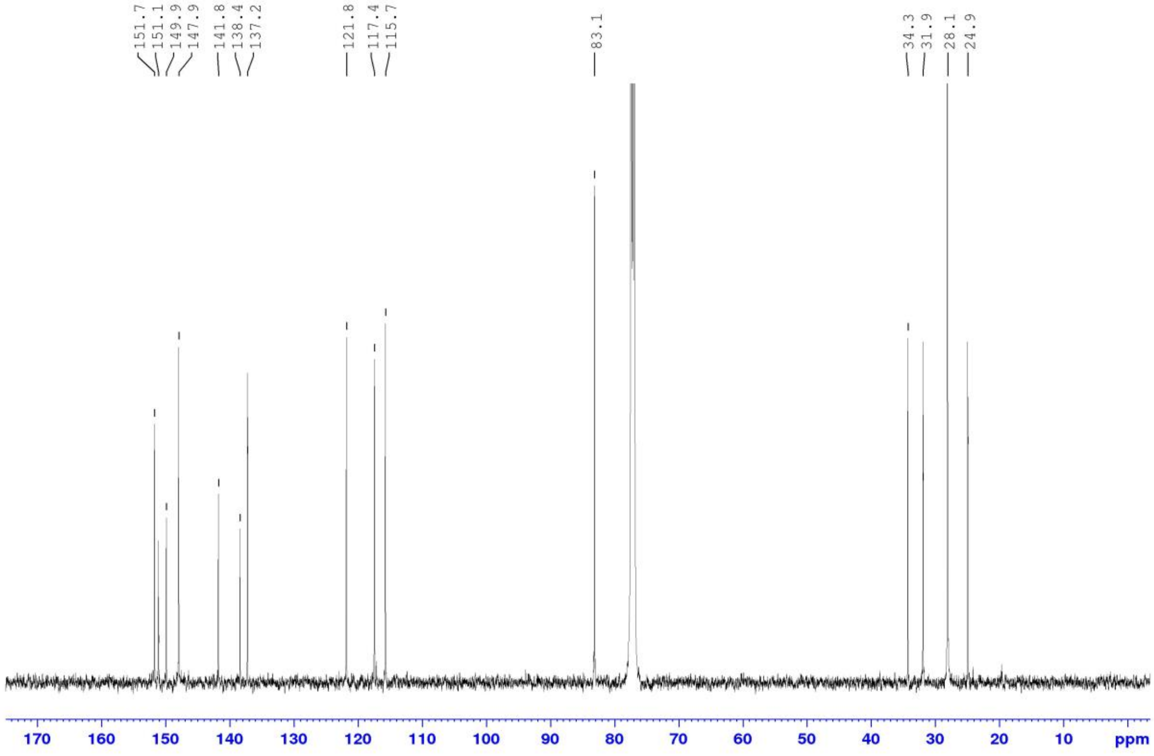

N,N-(bis-Boc)-4-(but-3-en-1-yl)-5-(1-phenylvinyl)pyridin-2-amine, **16e**

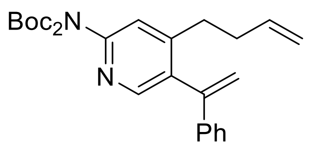

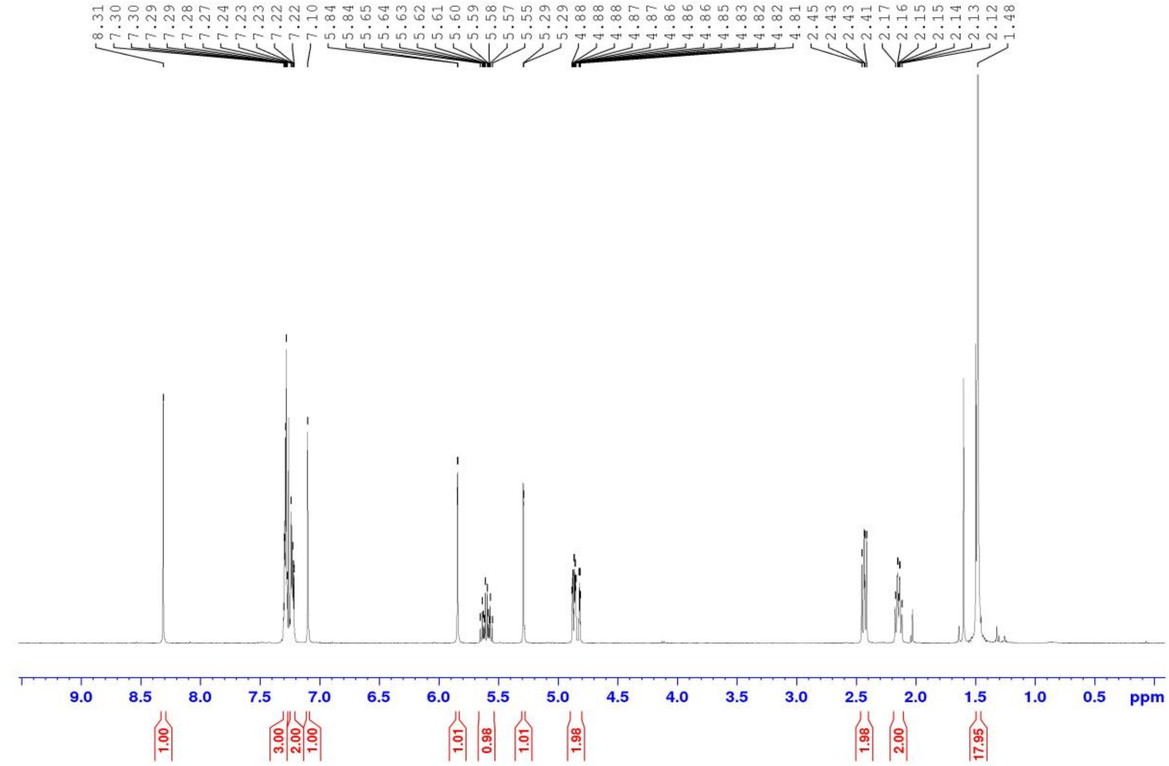

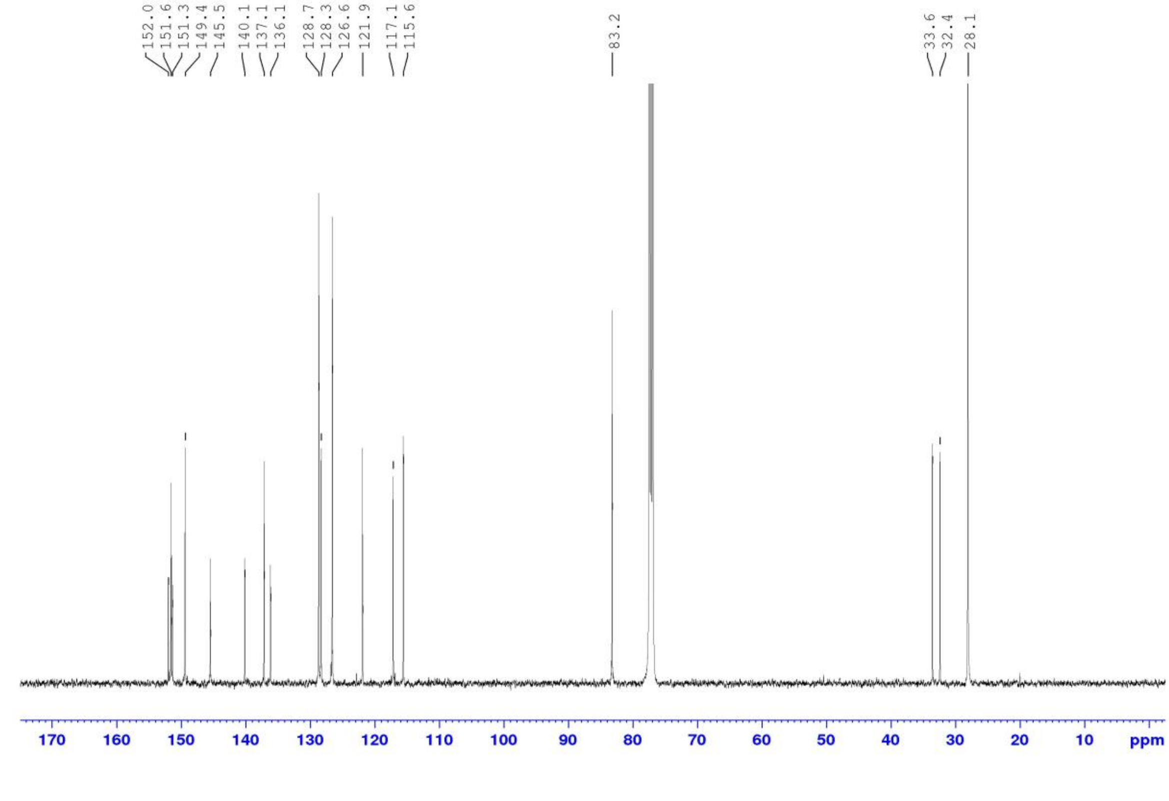

5-Allyl-*N,N*-(bis-Boc)-4-(but-3-en-1-yl)pyridin-2-amine, **16f**

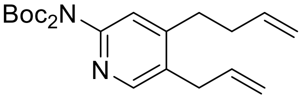

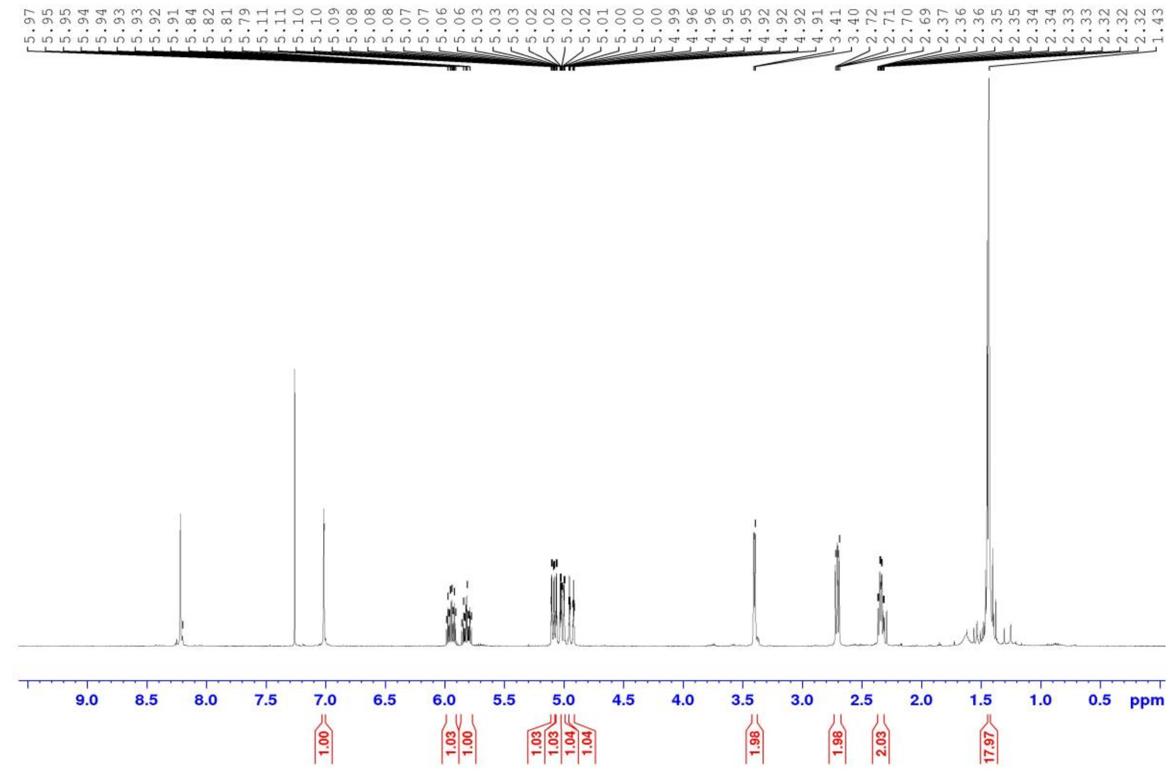

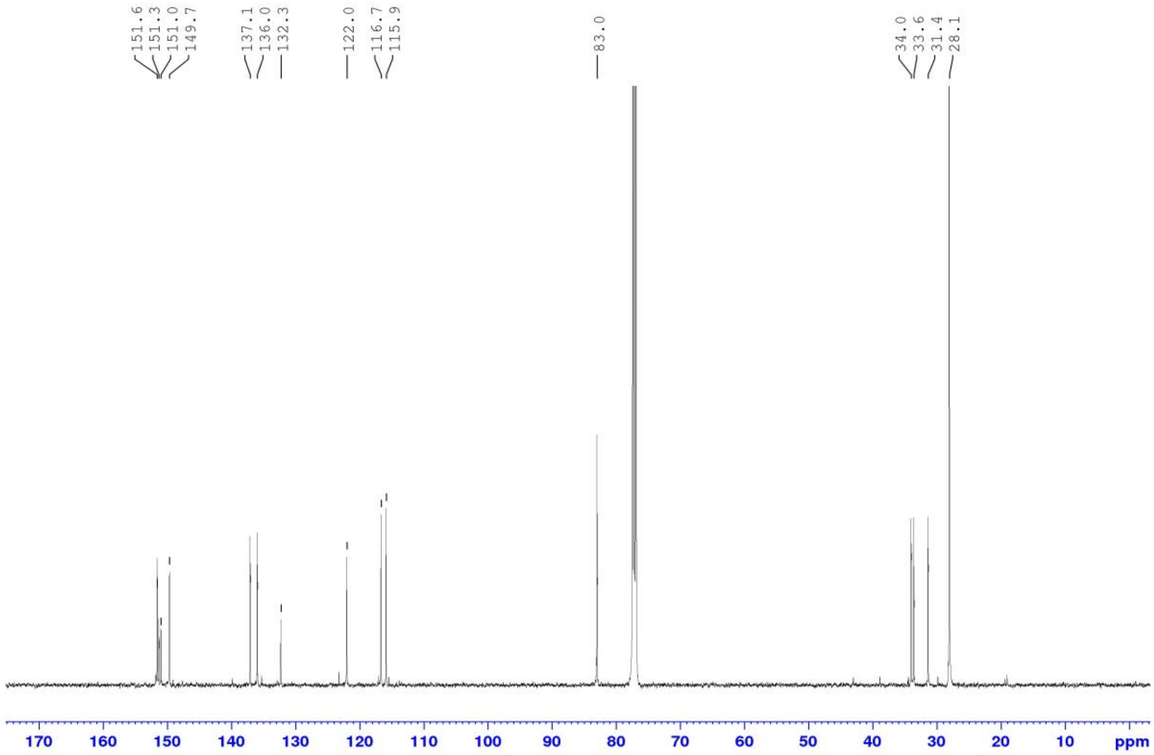

5-Allyl-4-(but-3-en-1-yl)-2-chloropyridine, **16g**

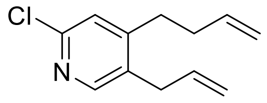

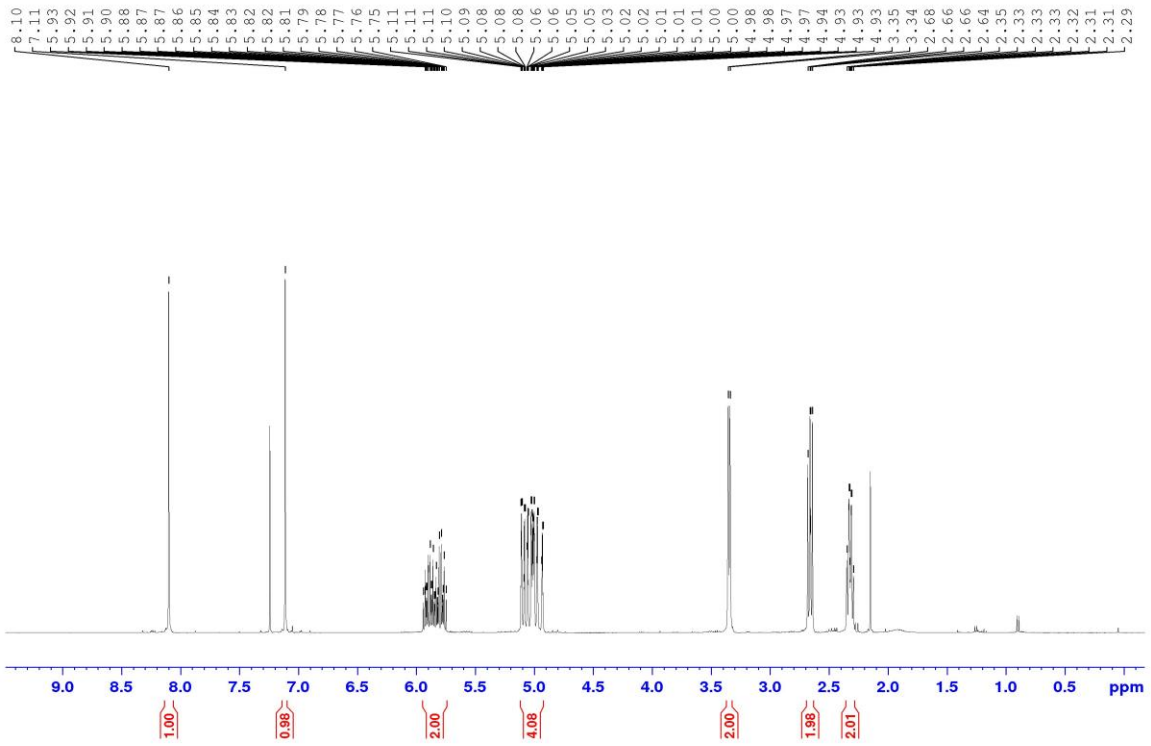

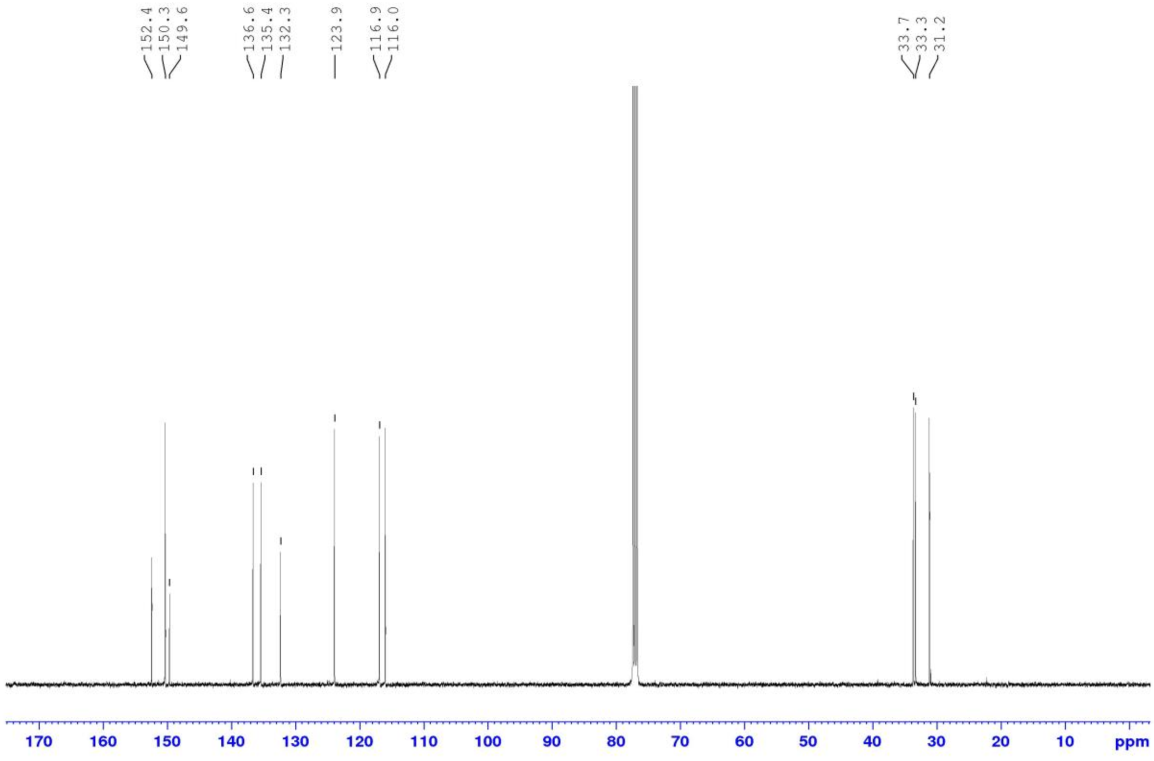

N,N-(bis-Boc)-5,6-dihydroisoquinolin-3-amine, **17a**

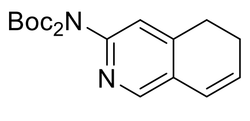

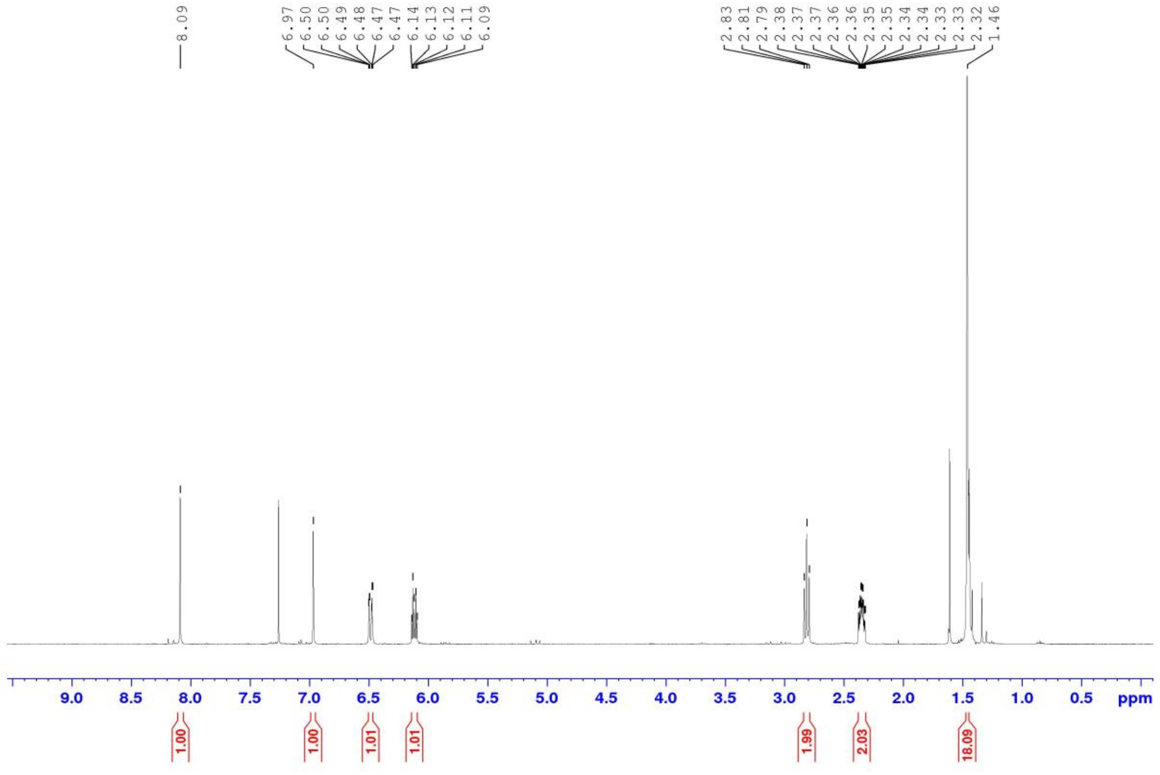

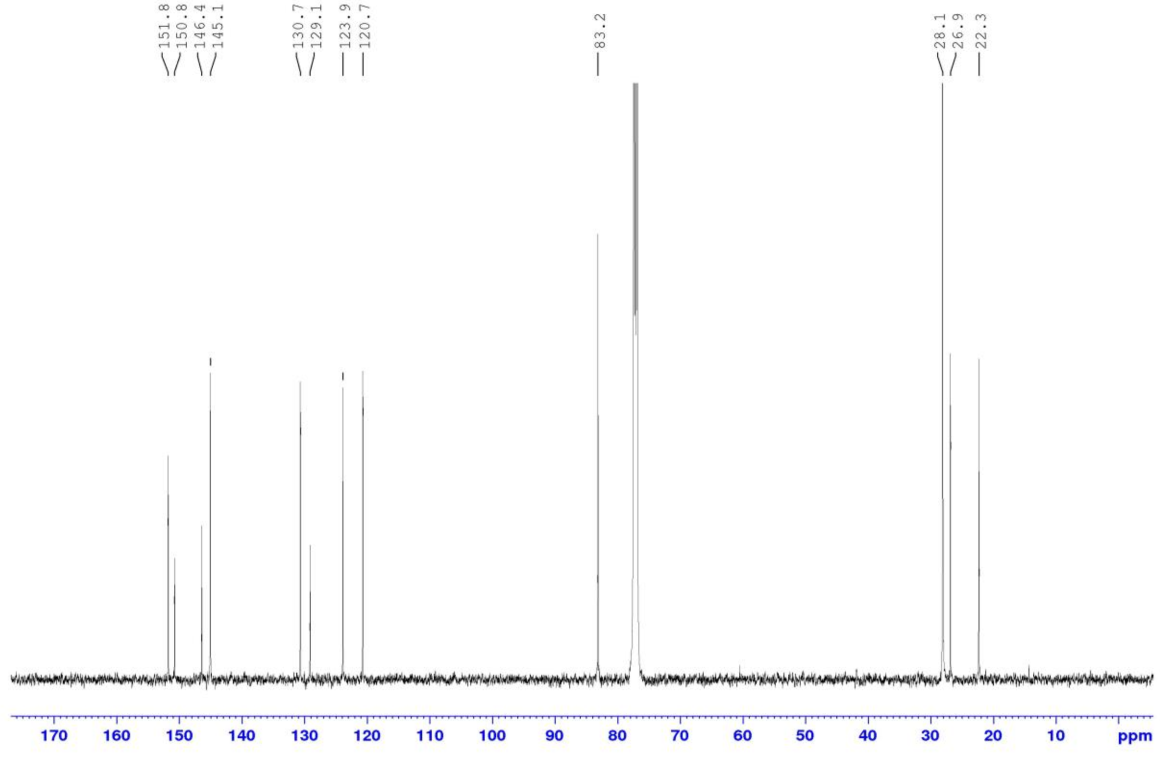

N,N-(bis-Boc)-7-methyl-5,6-dihydroisoquinolin-3-amine, **17b**

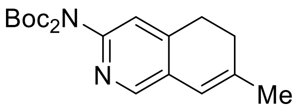

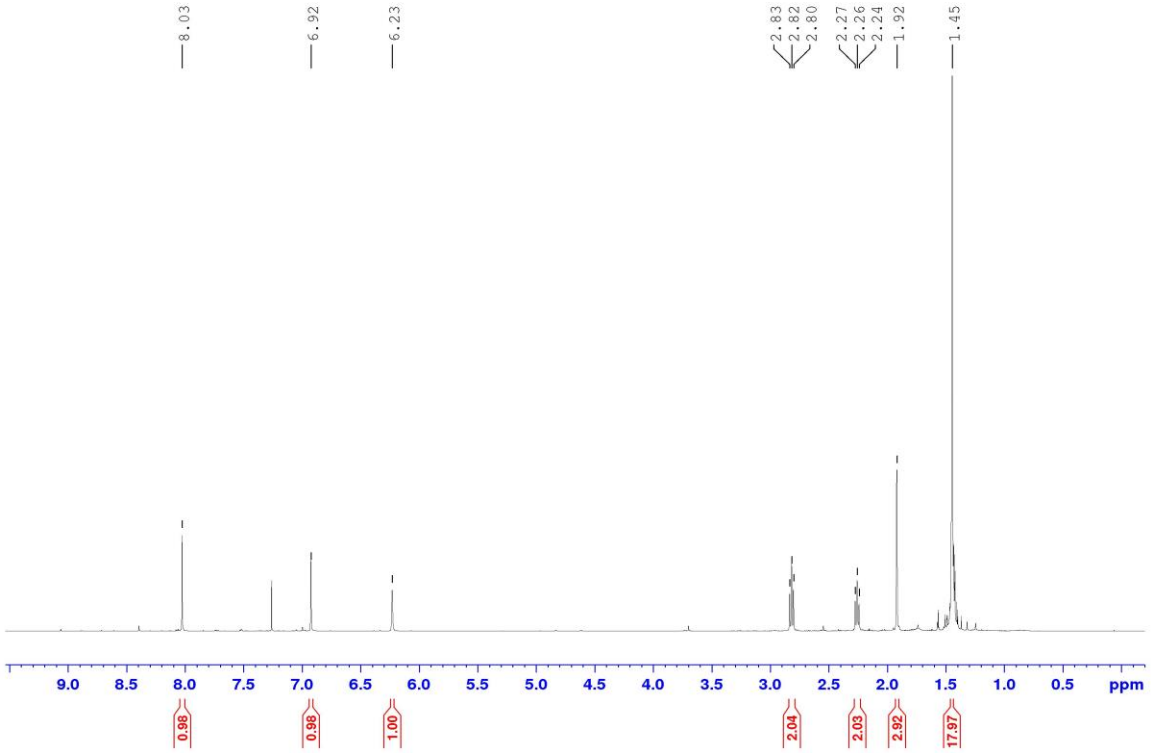

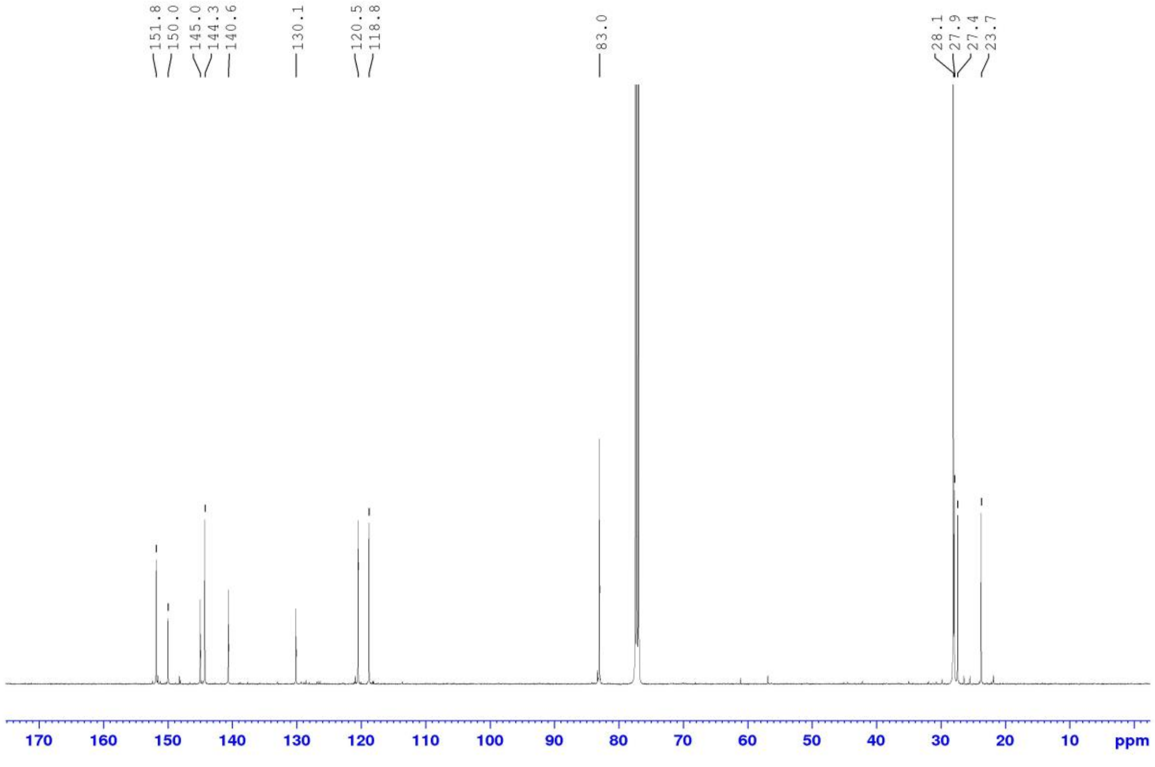

N,N-(bis-Boc)-7-trifluoromethyl-5,6-dihydroisoquinolin-3-amine, **17c**

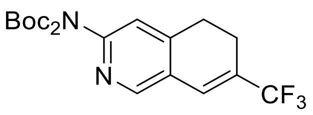

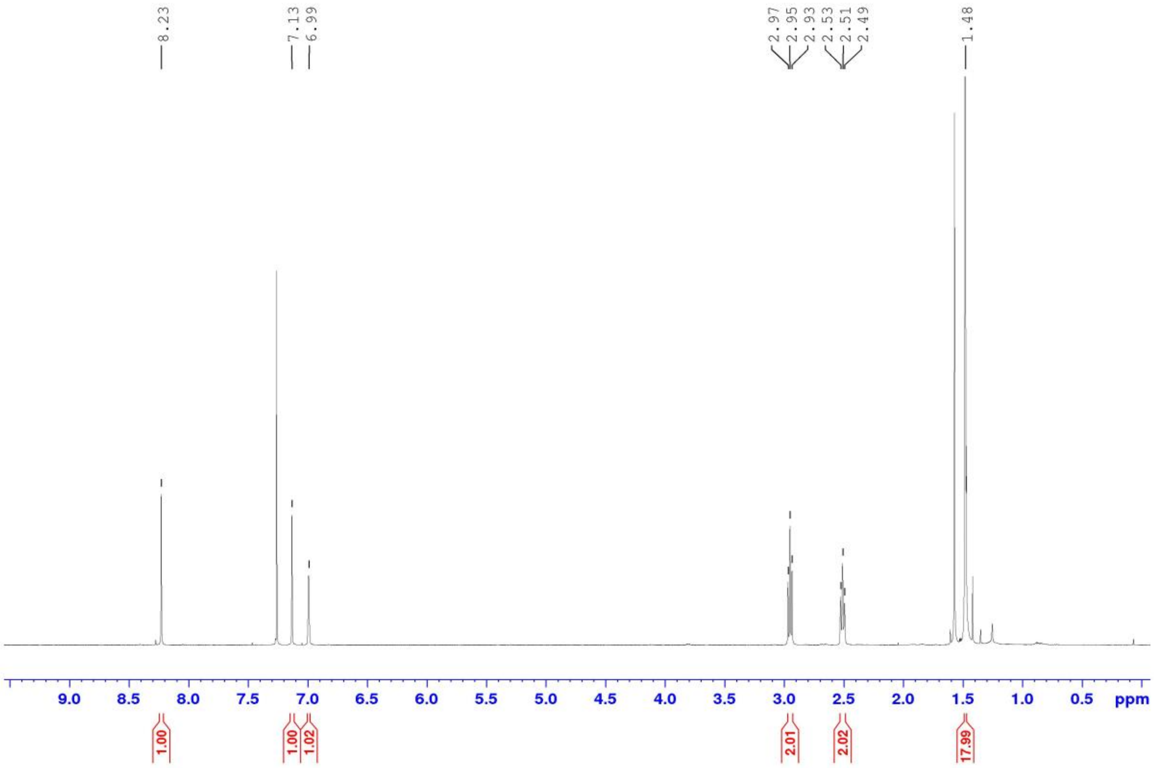

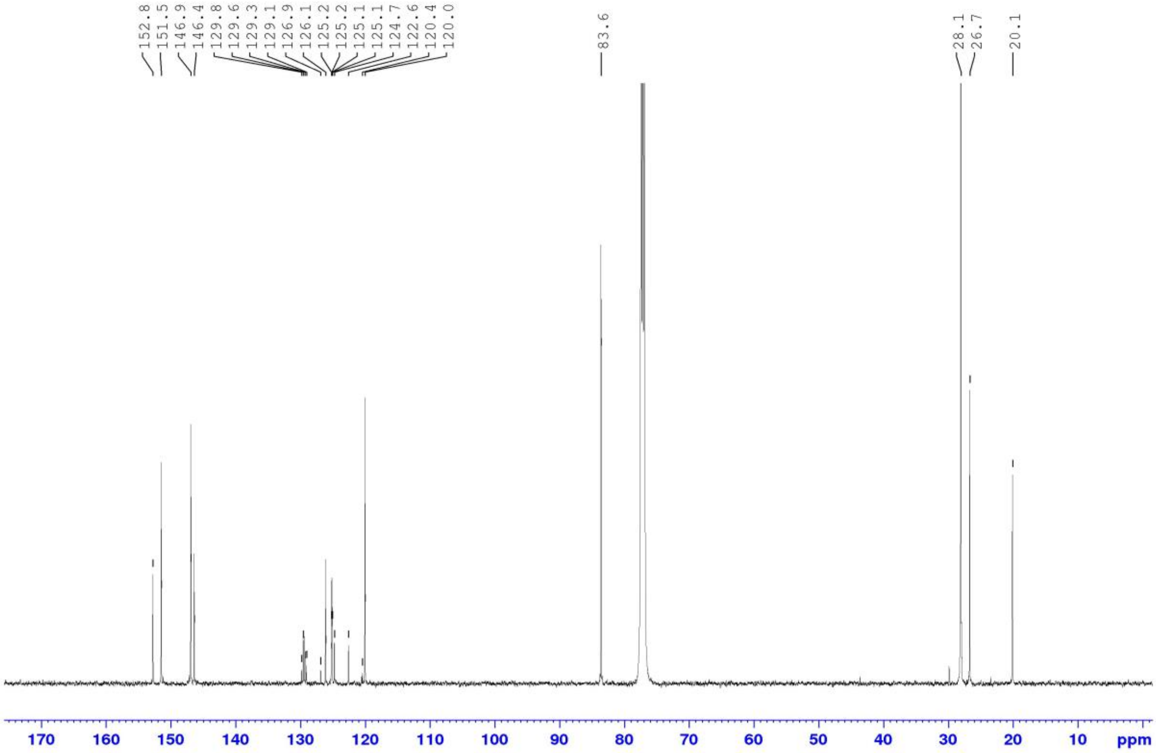

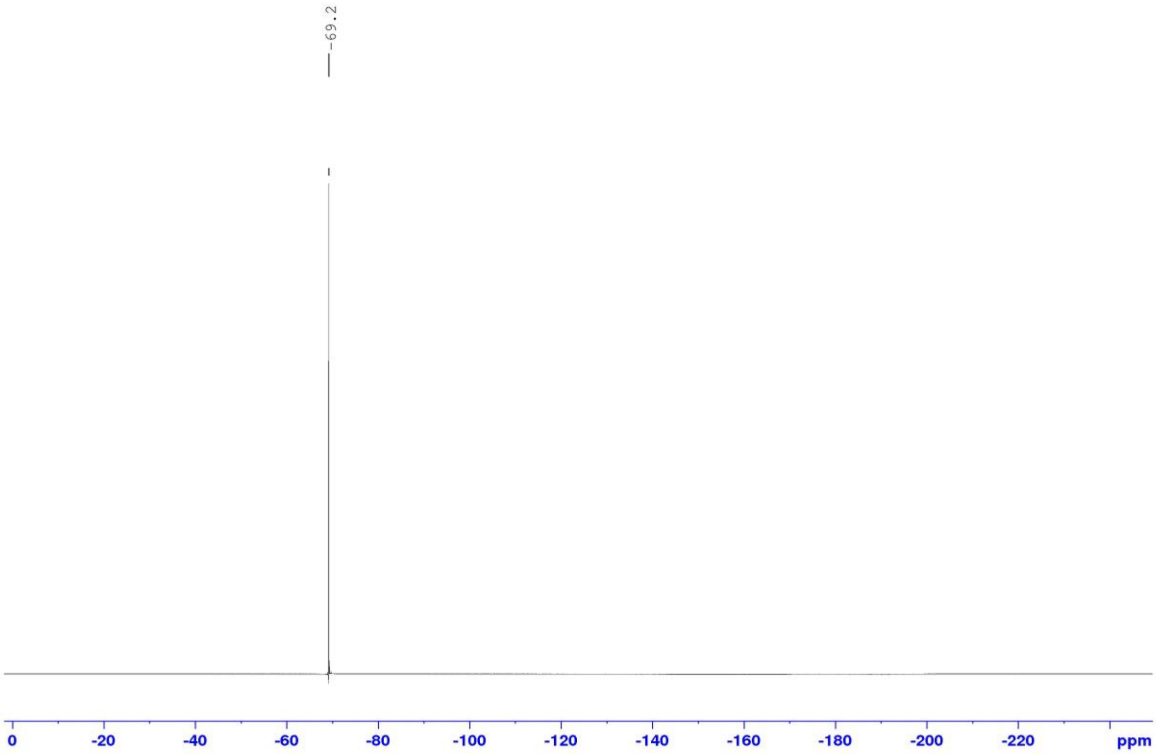

-69.2
0
-20
-40
-60
-80
-100
-120
-140
-160
-180
-200
-220
ppm

N,N-(bis-Boc)-8-methyl-5,6-dihydroisoquinolin-3-amine, **17d**

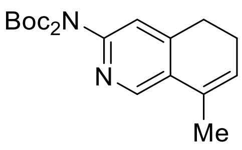

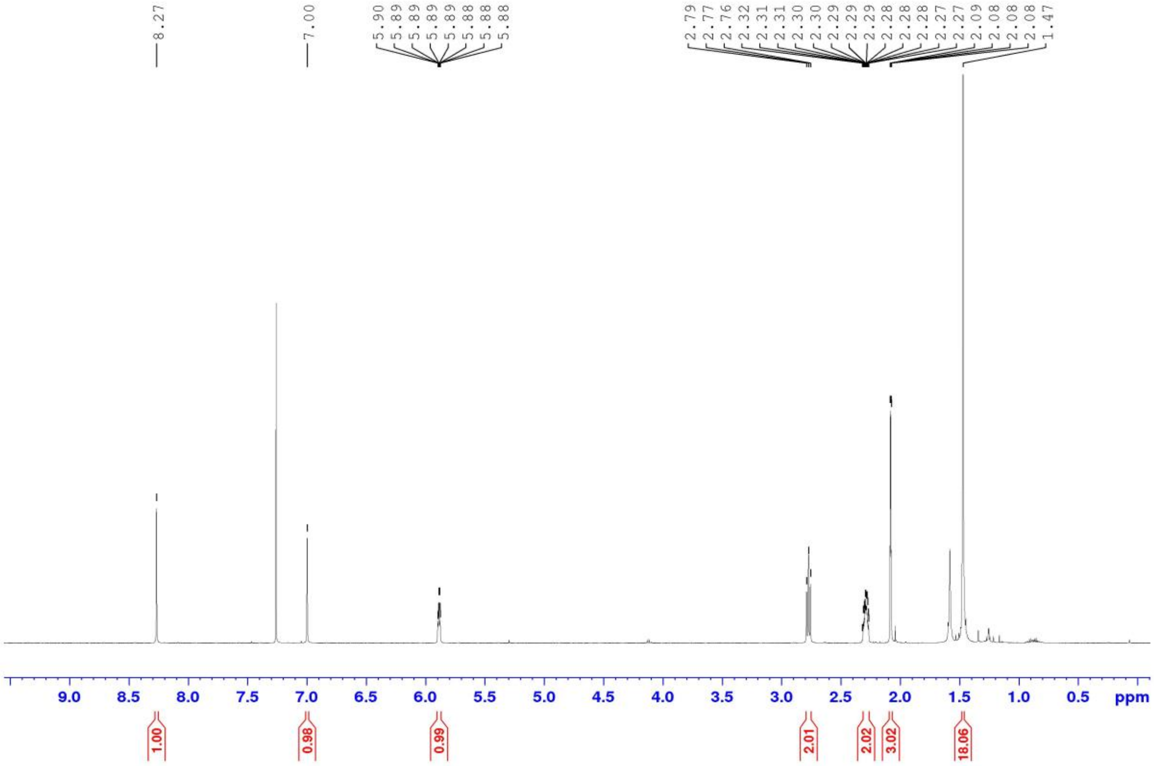

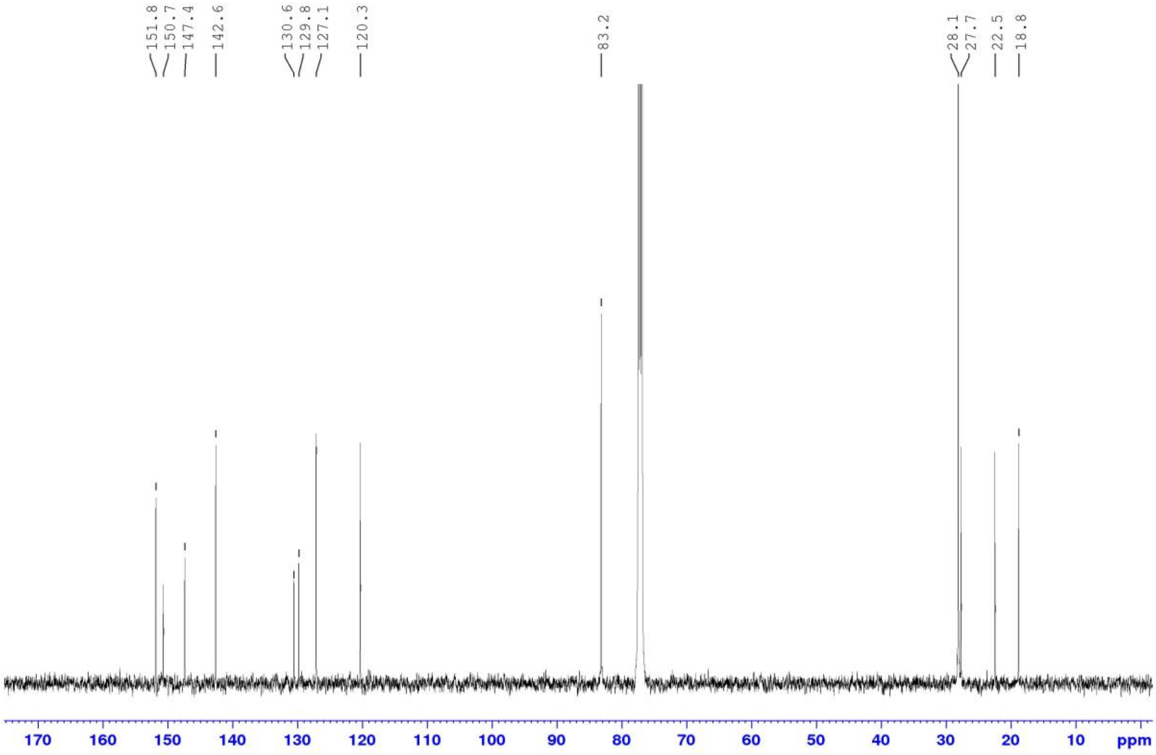

N,N-(bis-Boc)-8-phenyl-5,6-dihydroisoquinolin-3-amine, **17e**

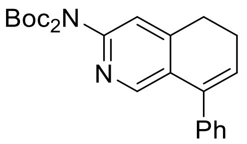

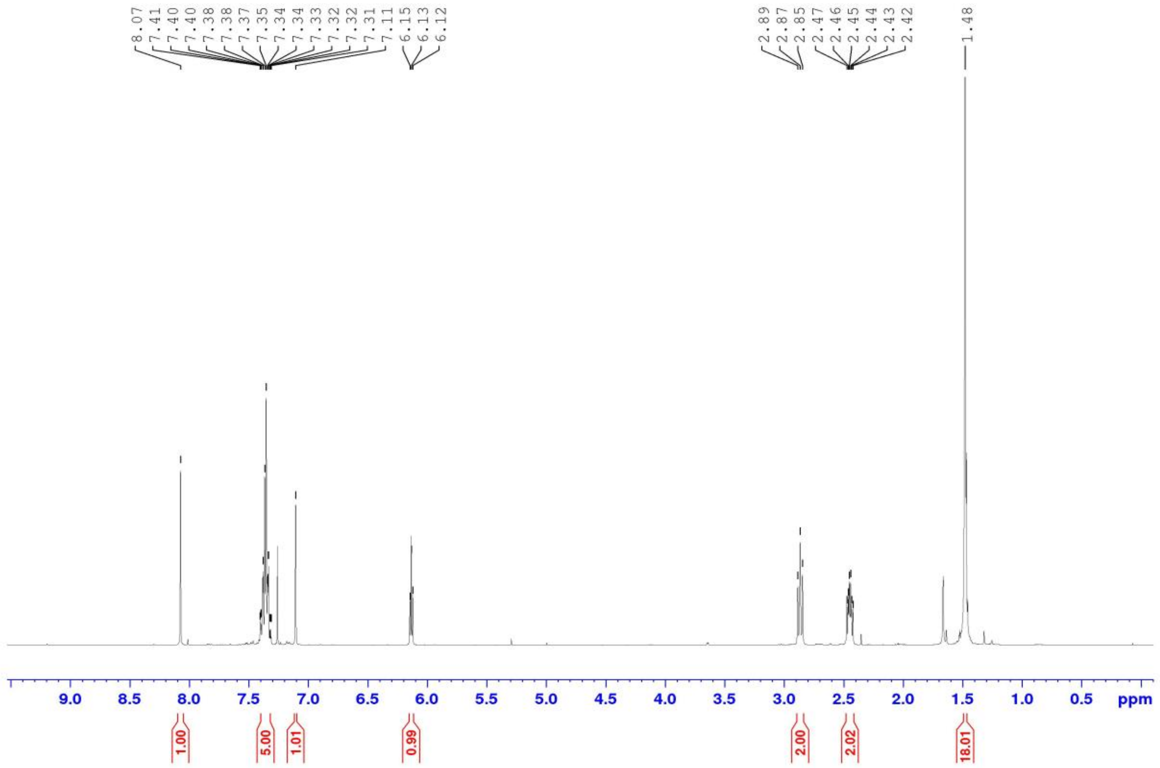

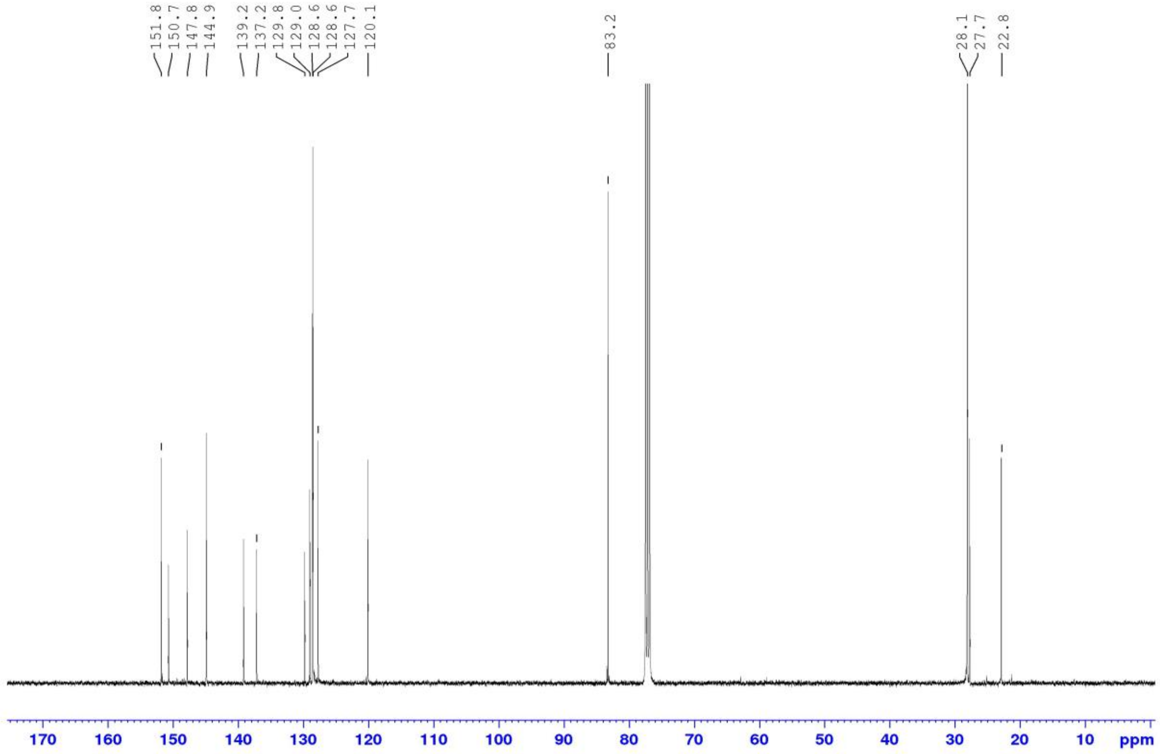

N,N-(bis-Boc)-6,9-dihydro-5*H*-cyclohepta[*c*]pyridin-3-amine, **17f**

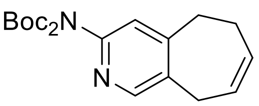

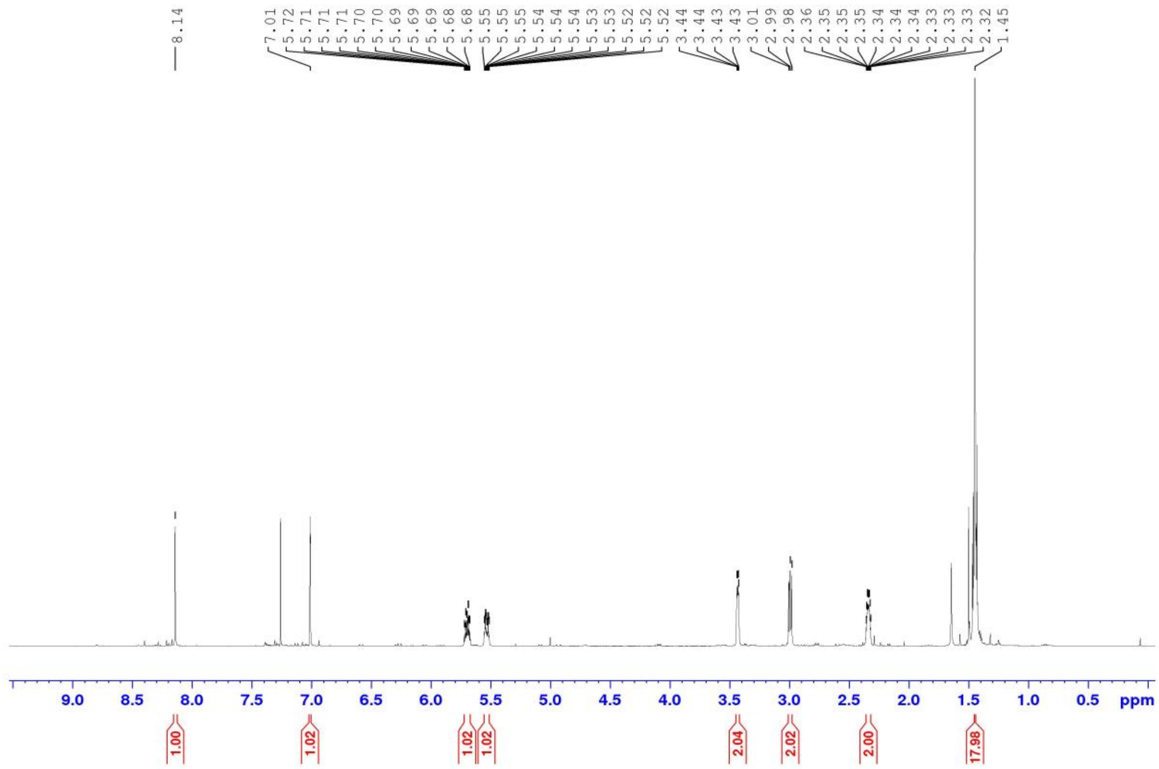

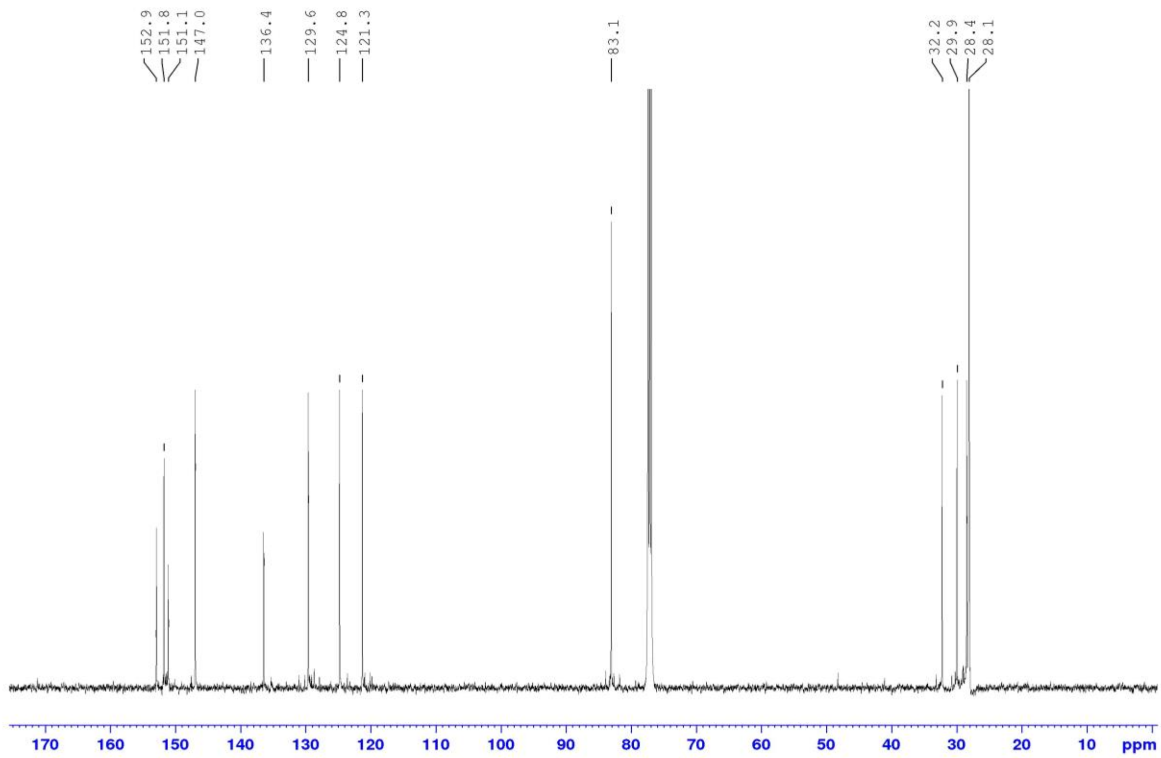

3-Chloro-6,9-dihydro-5*H*-cyclohepta[*c*]pyridine, **17g**

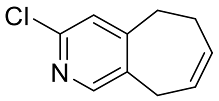

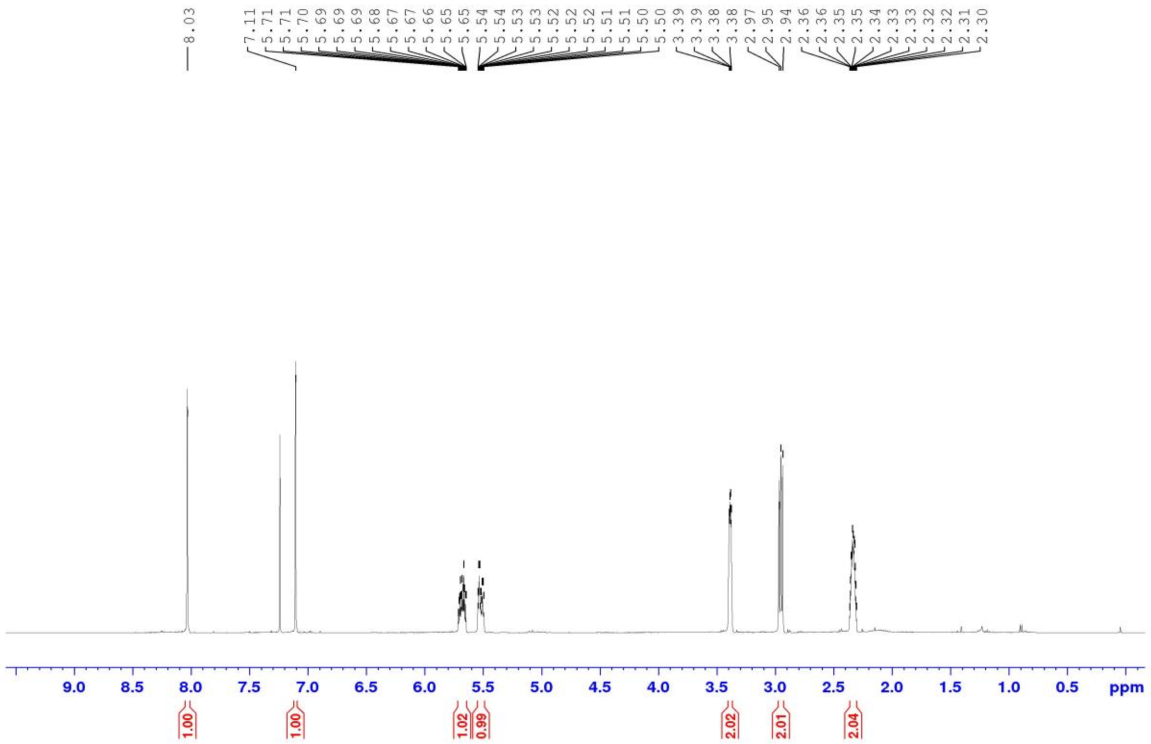

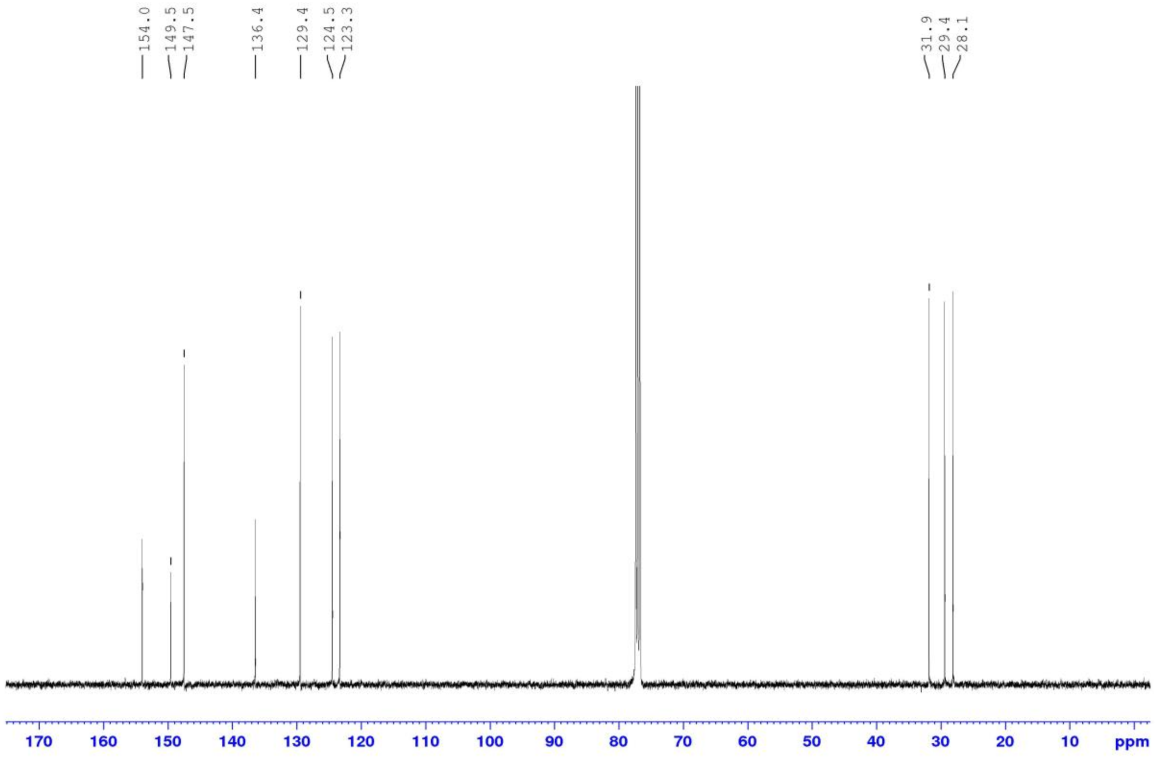

5,6-Dihydro-4*H*-pyrrolo[1,2-*b*]pyrazol-2-amine, **19a**

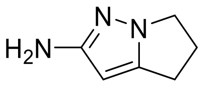

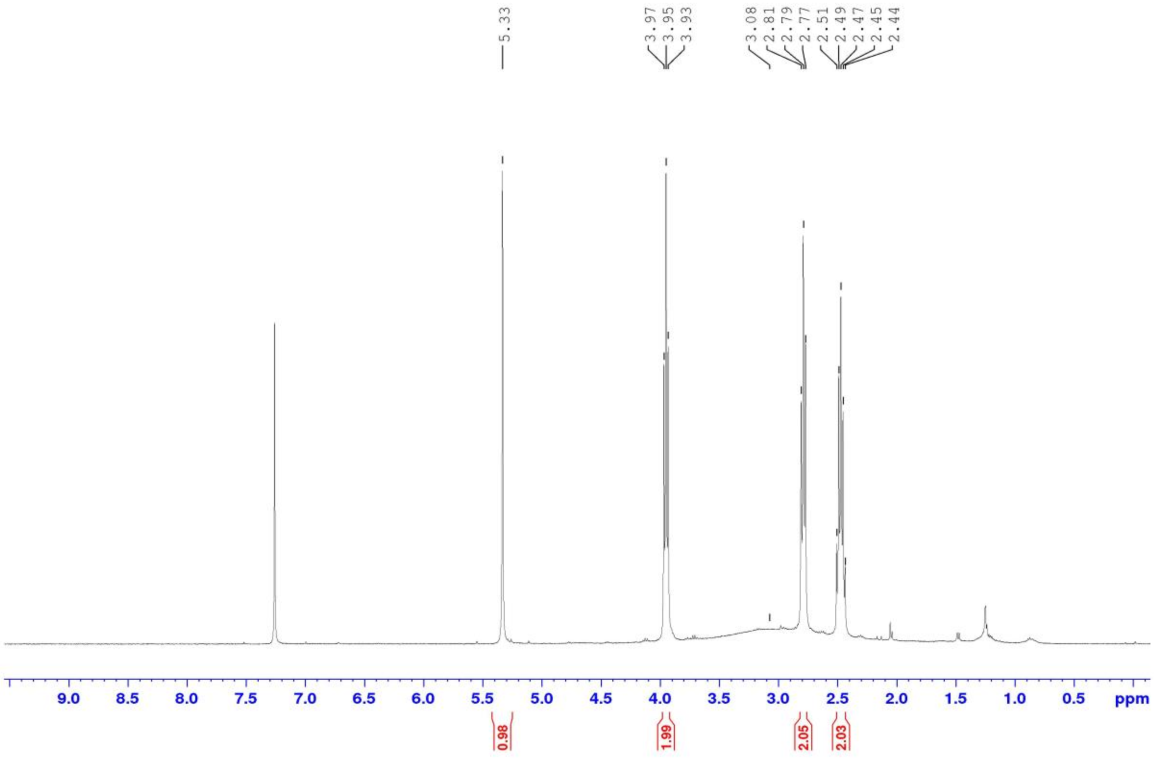

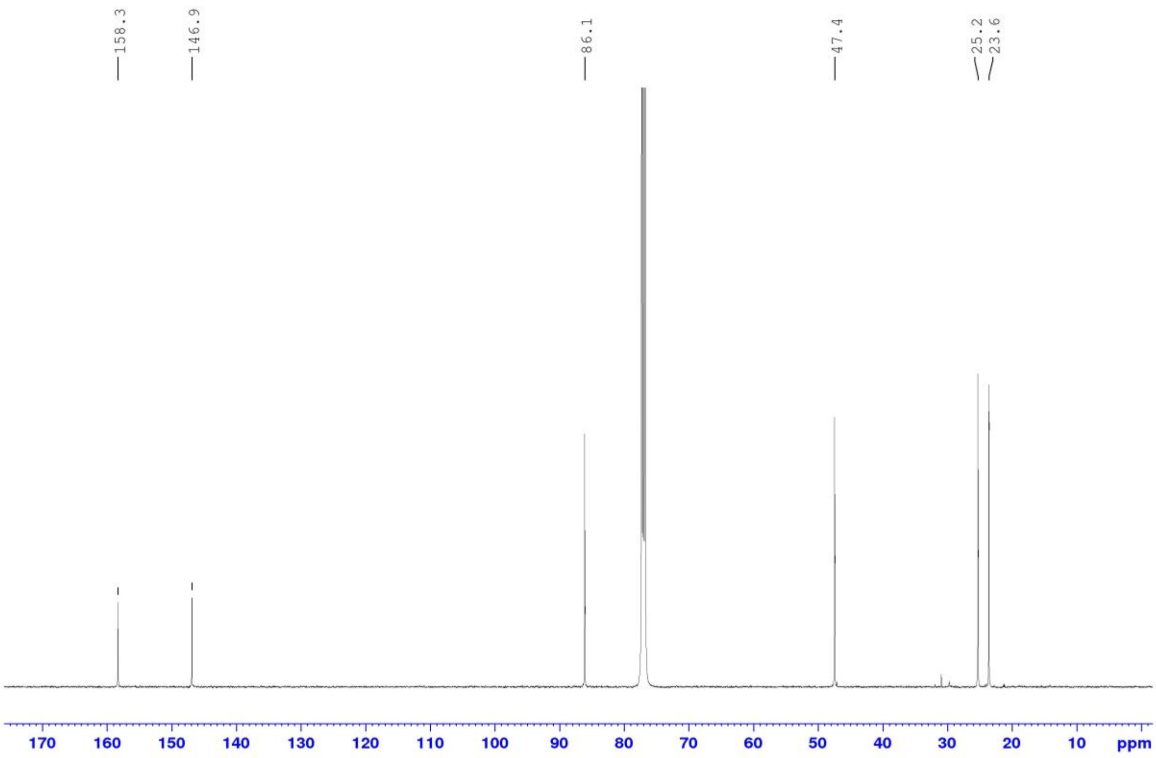

4,5,6,7-Tetrahydropyrazolo[1,5-*a*]pyridin-2-amine, **19b**

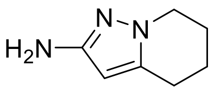

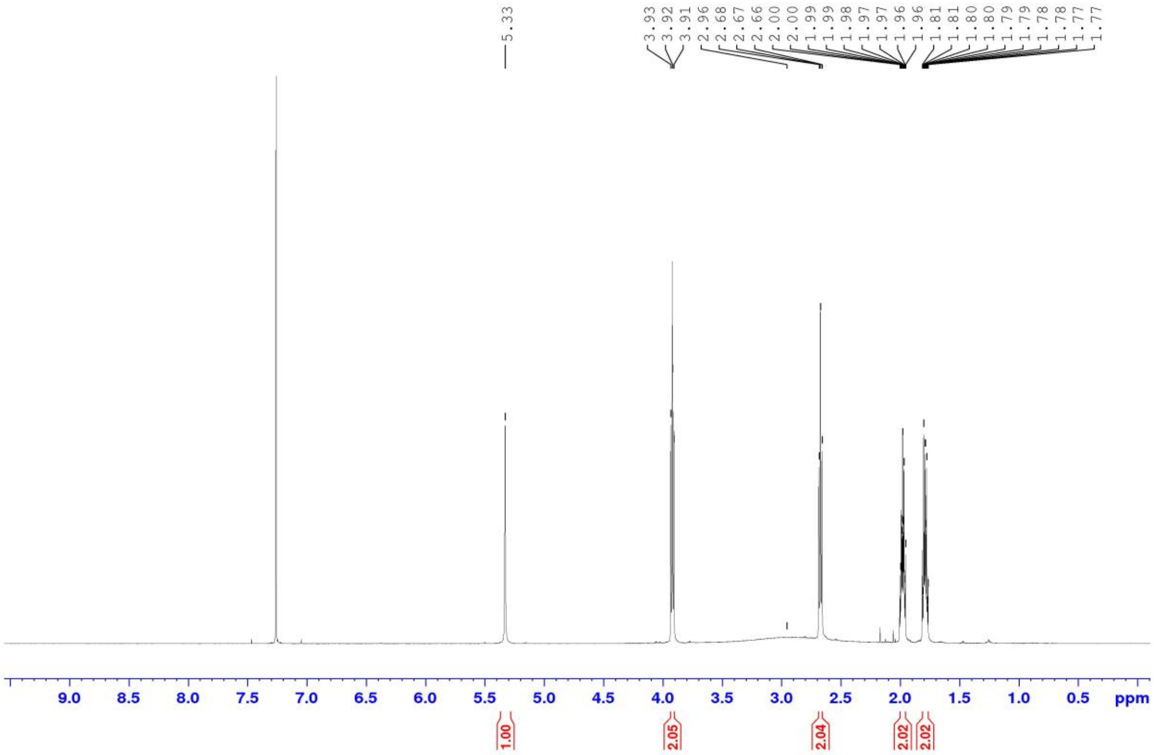

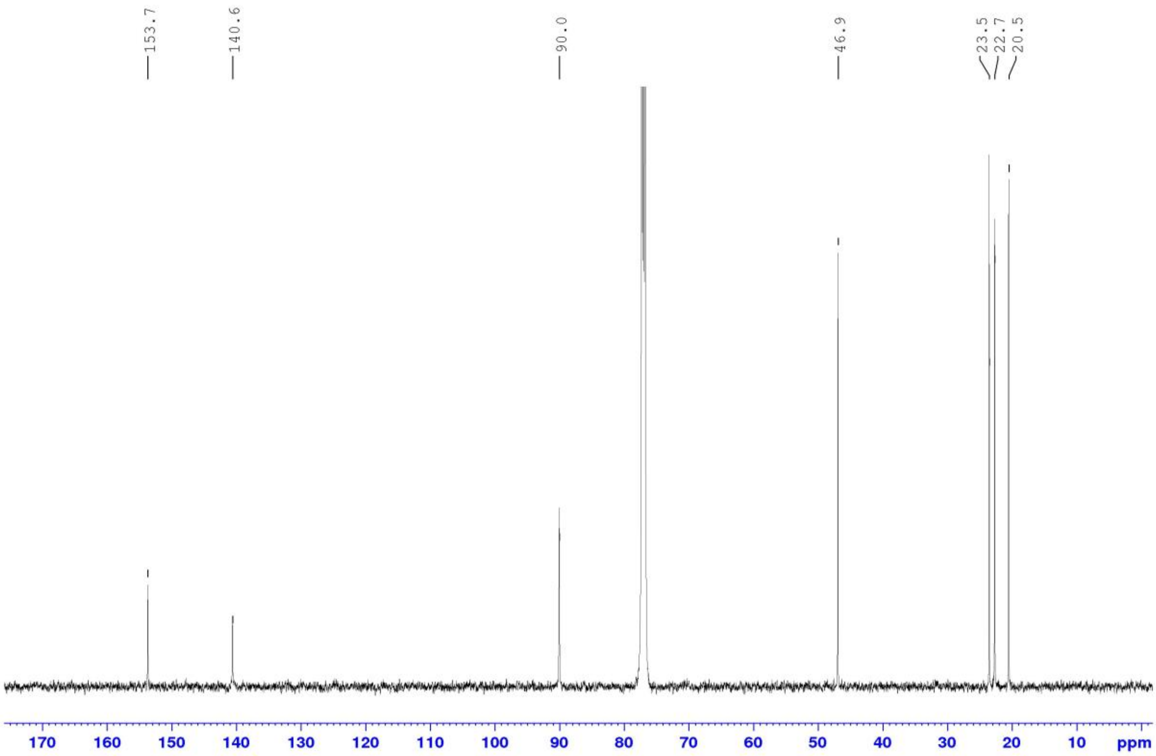

5,6,7,8-tetrahydro-4*H*-pyrazolo[1,5-*a*]azepin-2-amine, **19c**

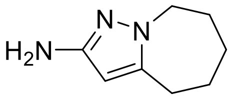

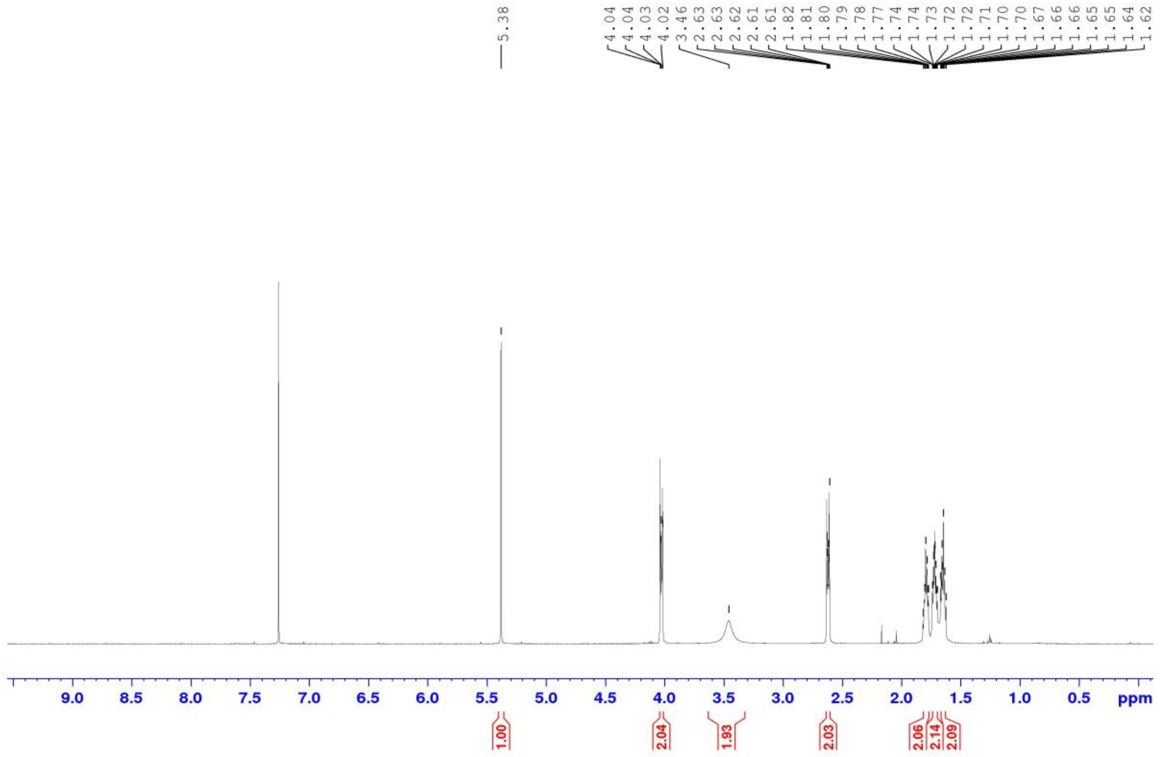

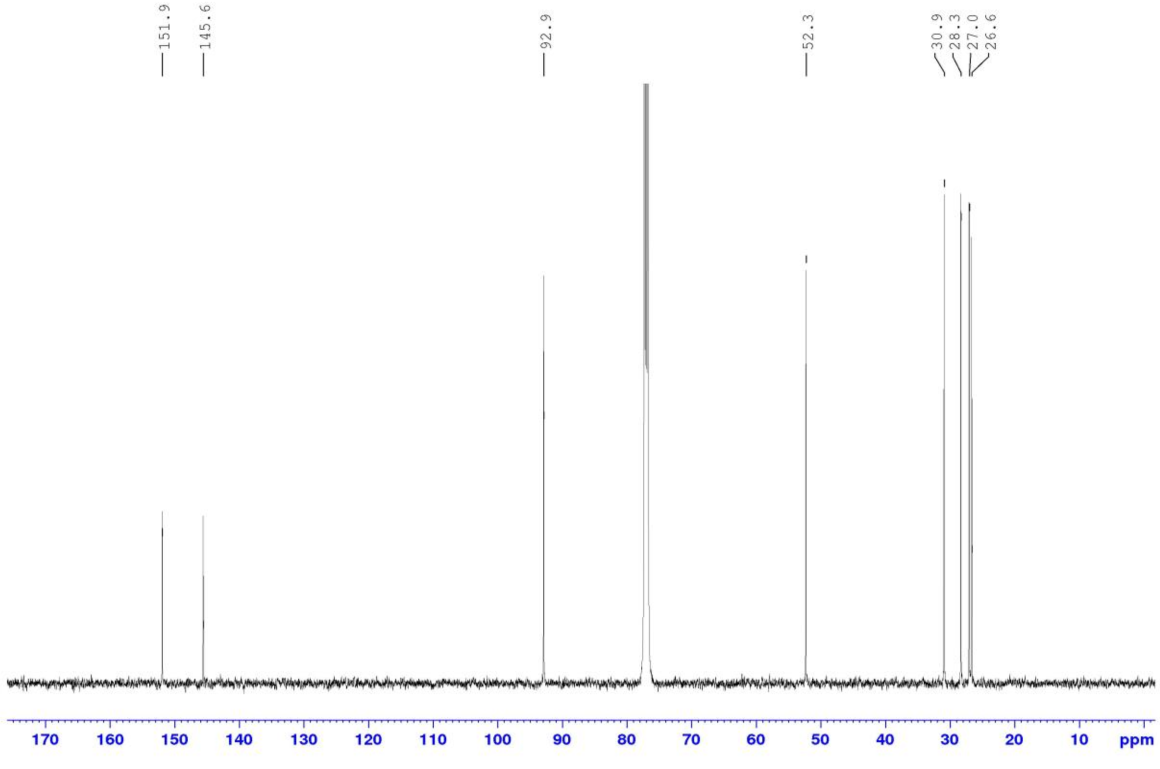

5,6,8,9-Tetrahydro-4*H*-pyrazolo[1,5-*d*][1,4]oxazocin-2-amine, **19d**

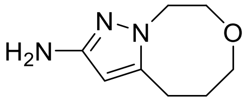

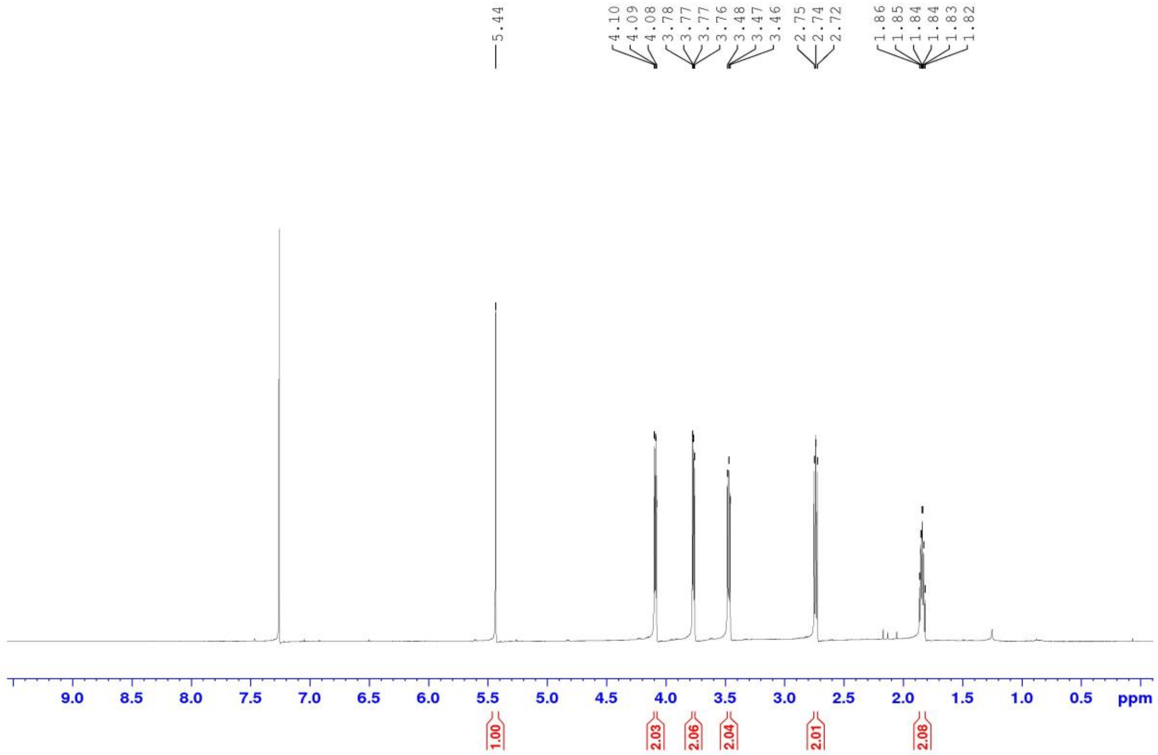

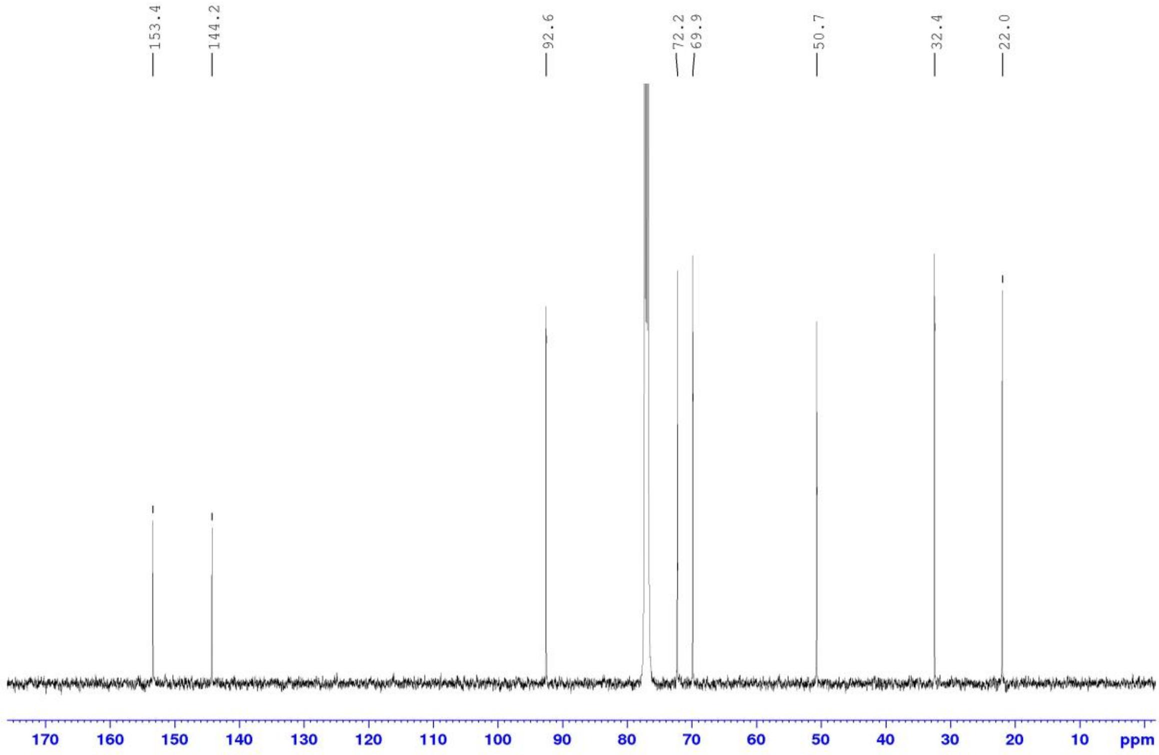

4-Methyl-4,5,6,7-tetrahydropyrazolo[1,5-*a*]pyridin-2-amine, **19f**

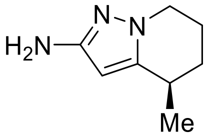

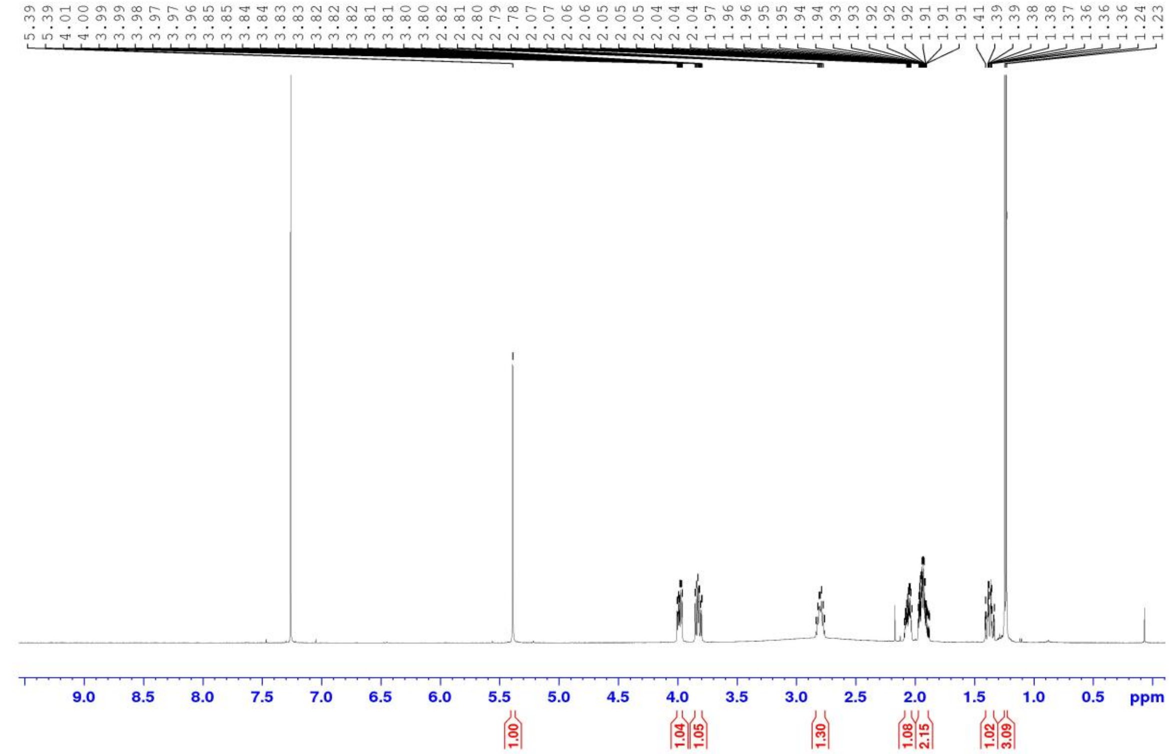

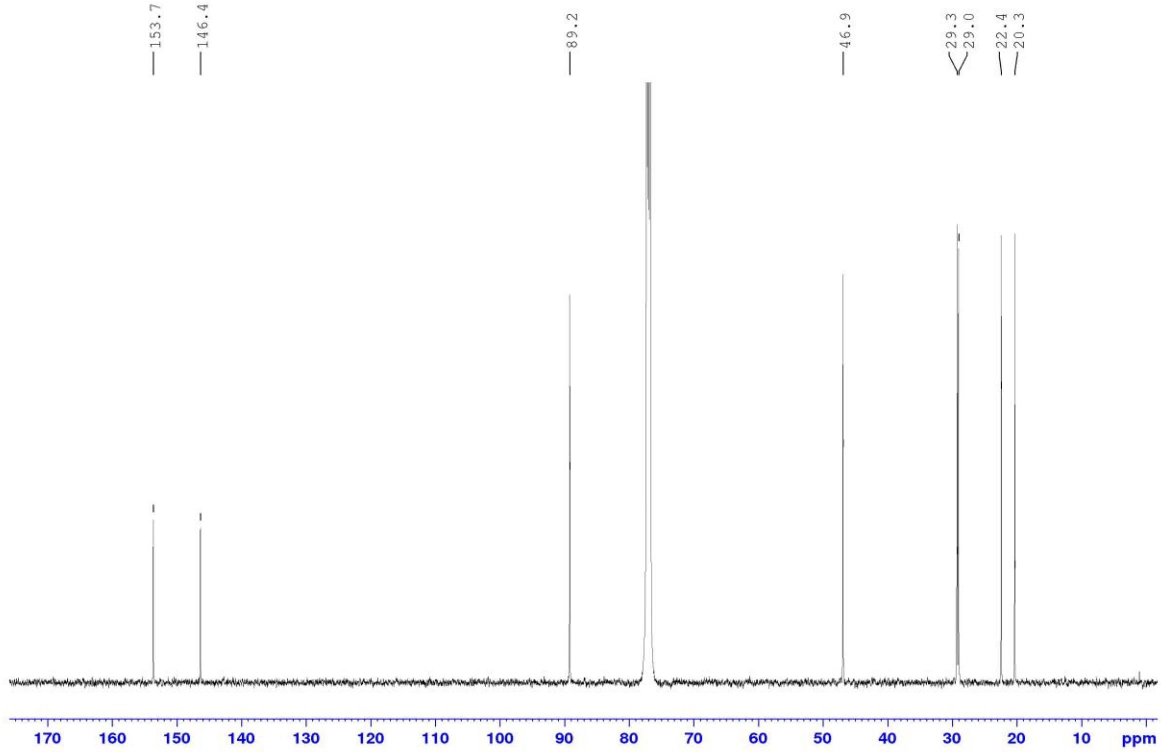

6-Nitro-1-tosyl-1a,2,3,7b-tetrahydro-1*H*-azirino[2,3-*c*]pyrazolo[1,5-*a*]pyridine, **20**

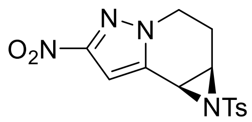

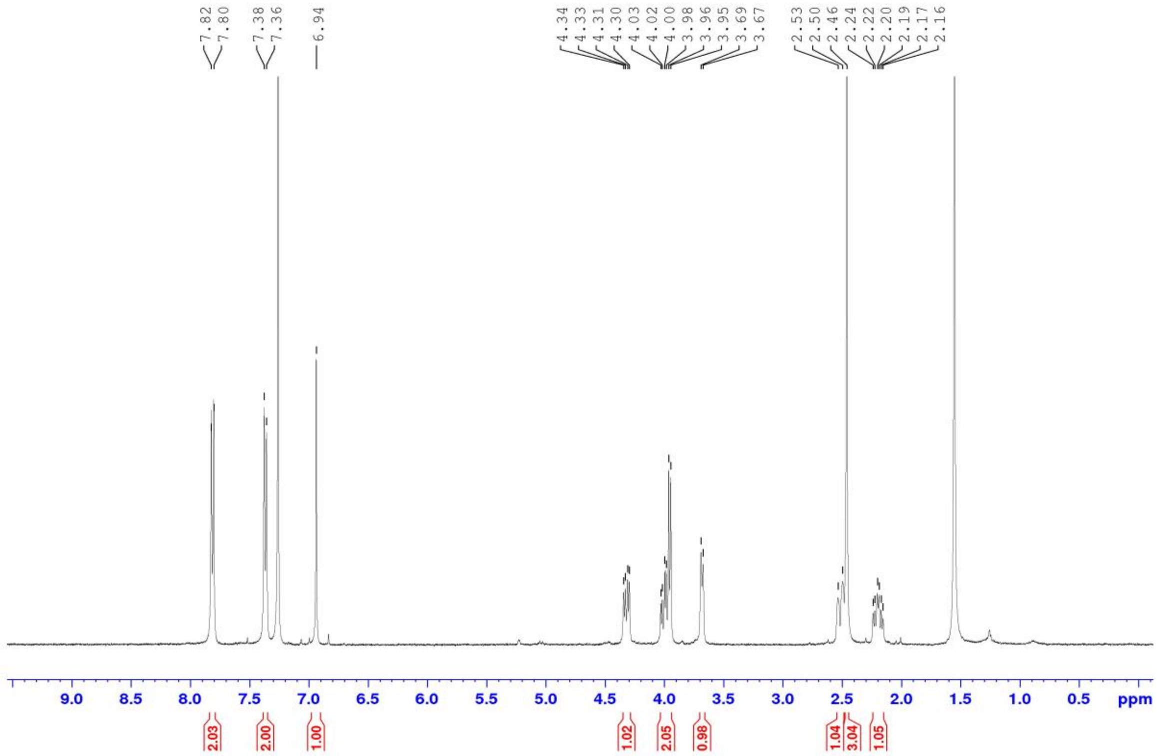

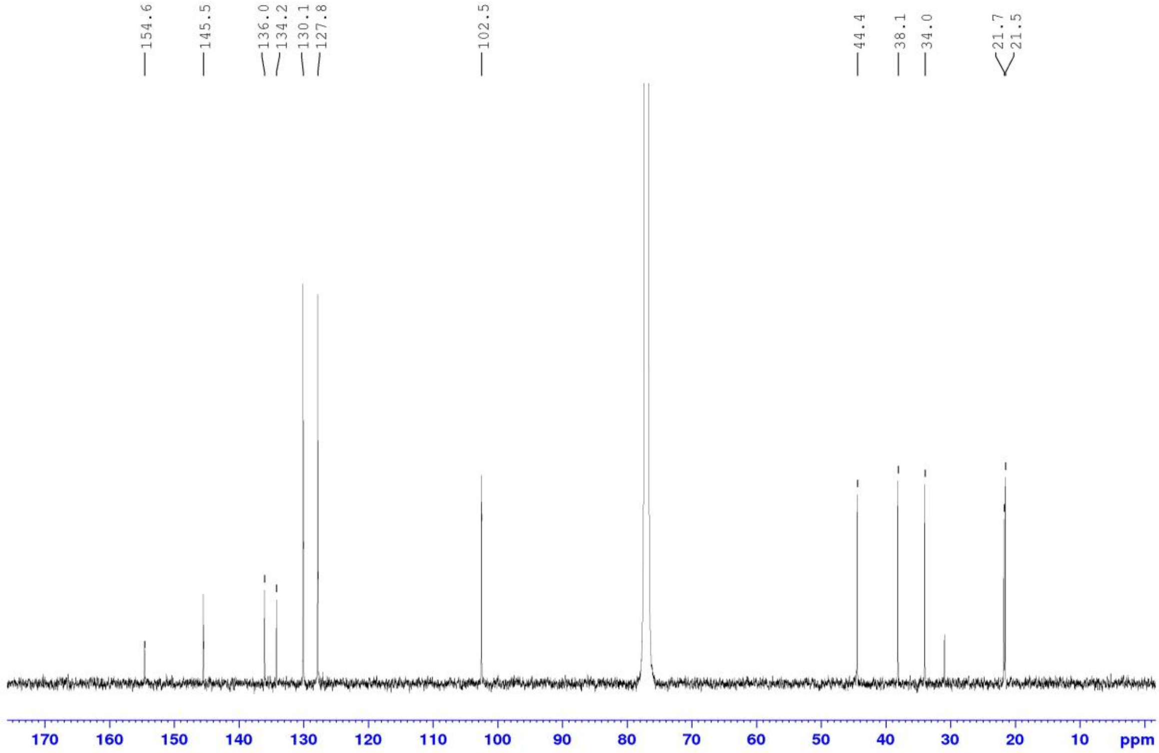

trans-4,5-Dibromo-2-nitro-4,5,6,7-tetrahydropyrazolo[1,5-*a*]pyridine, **21**

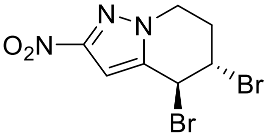

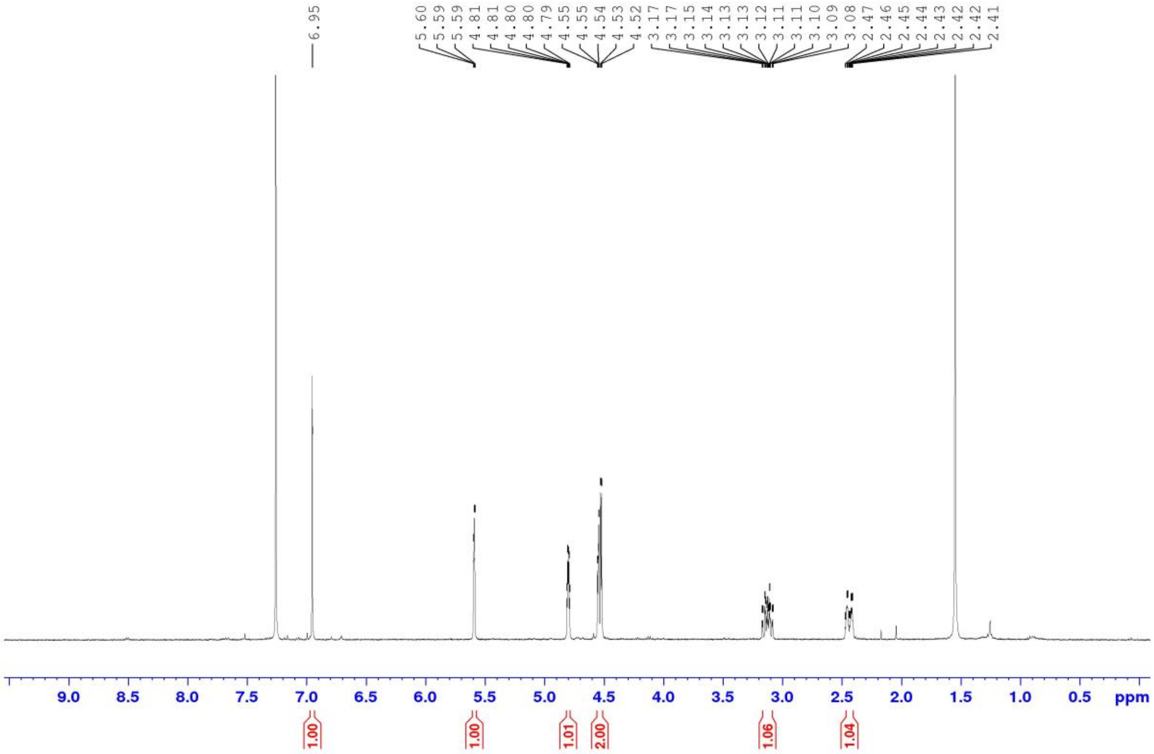

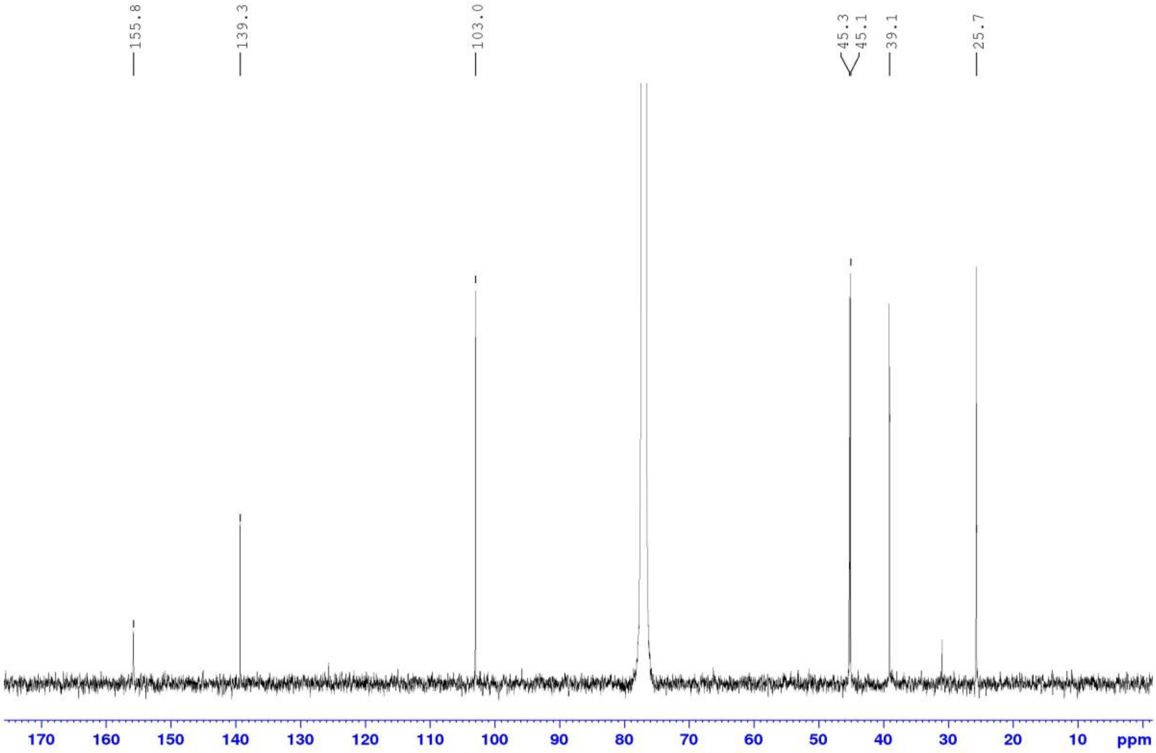

2-Nitro-6,7-dihydropyrazolo[1,5-*a*]pyridin-6-ol, **22**

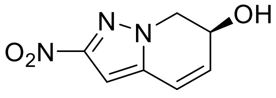

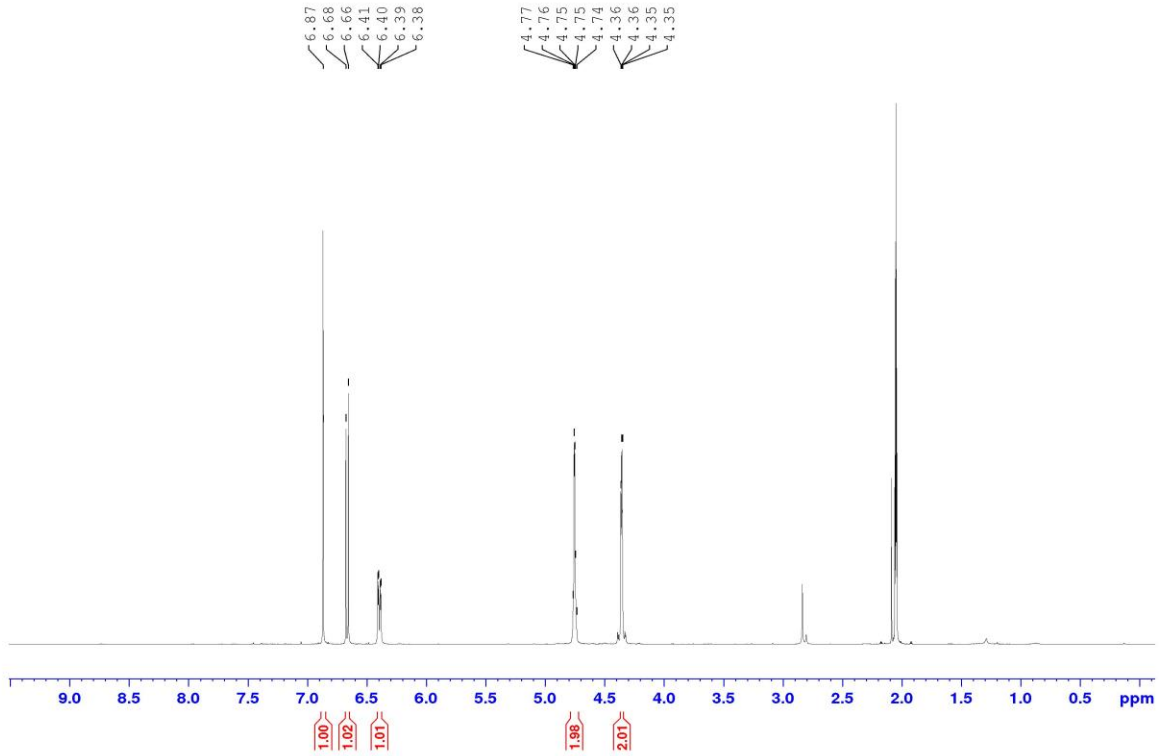

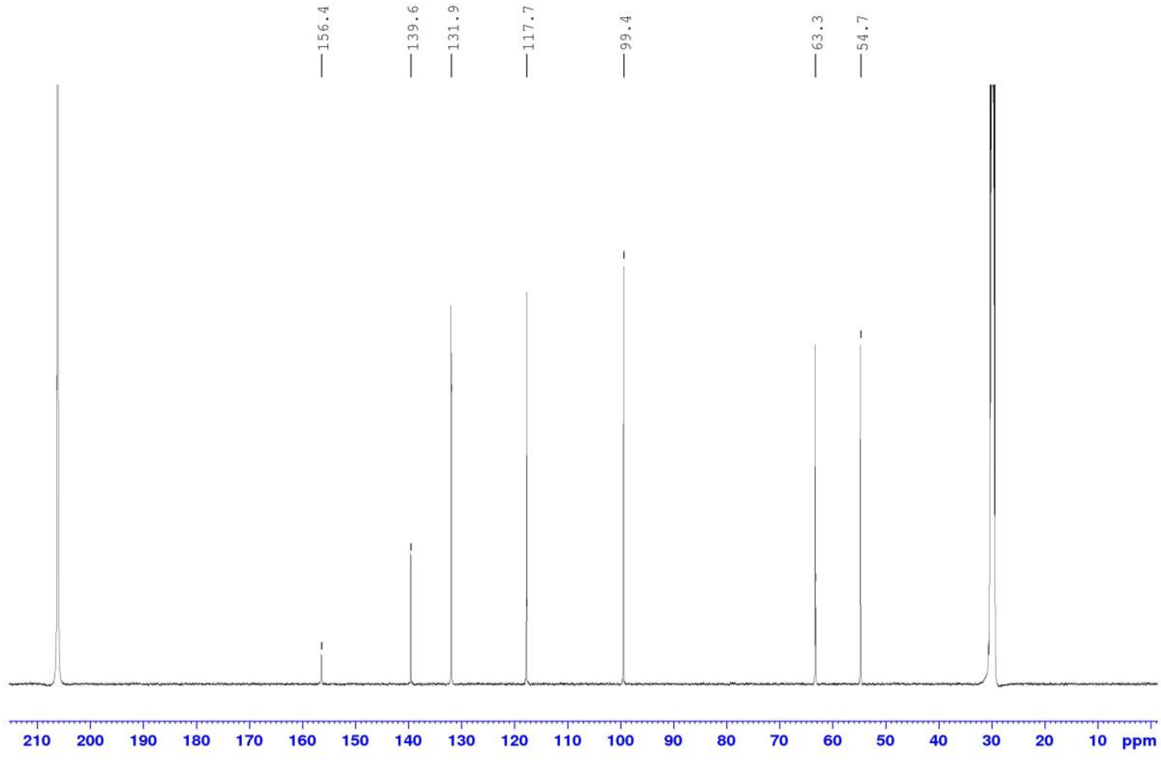

cis-2-Nitro-4,5,6,7-tetrahydropyrazolo[1,5-*a*]pyridine-4,5-diol, **23**

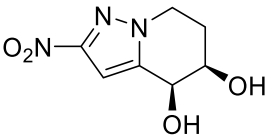

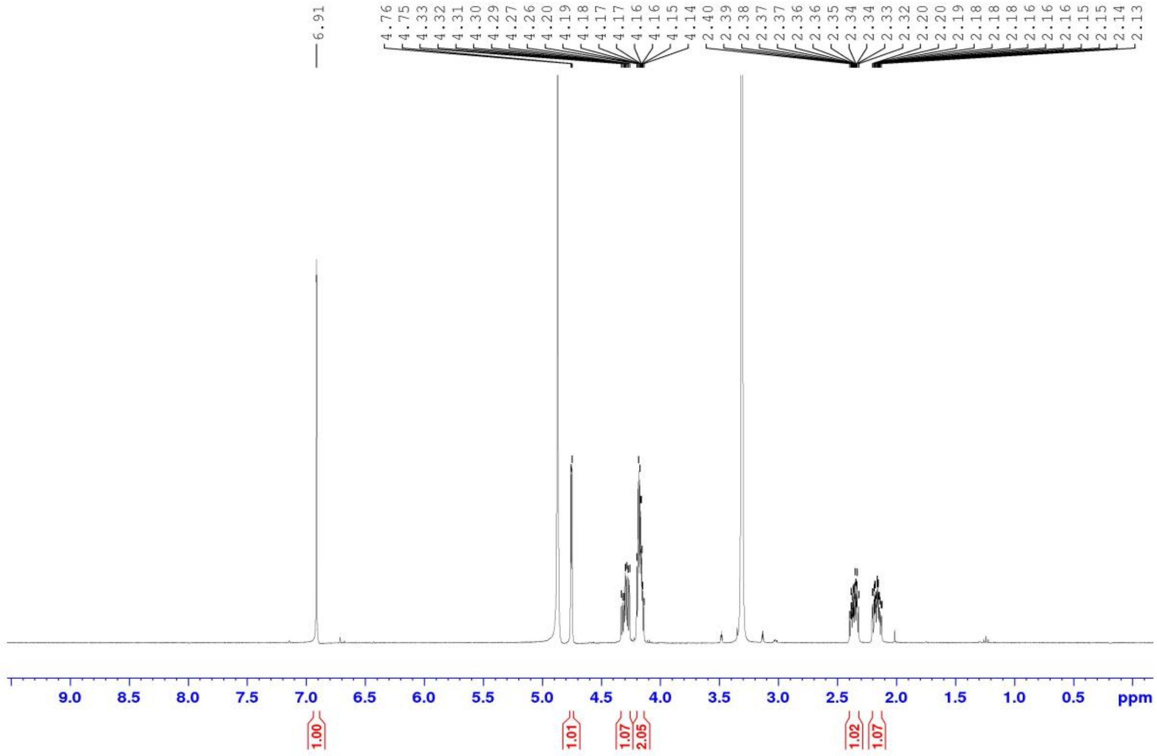

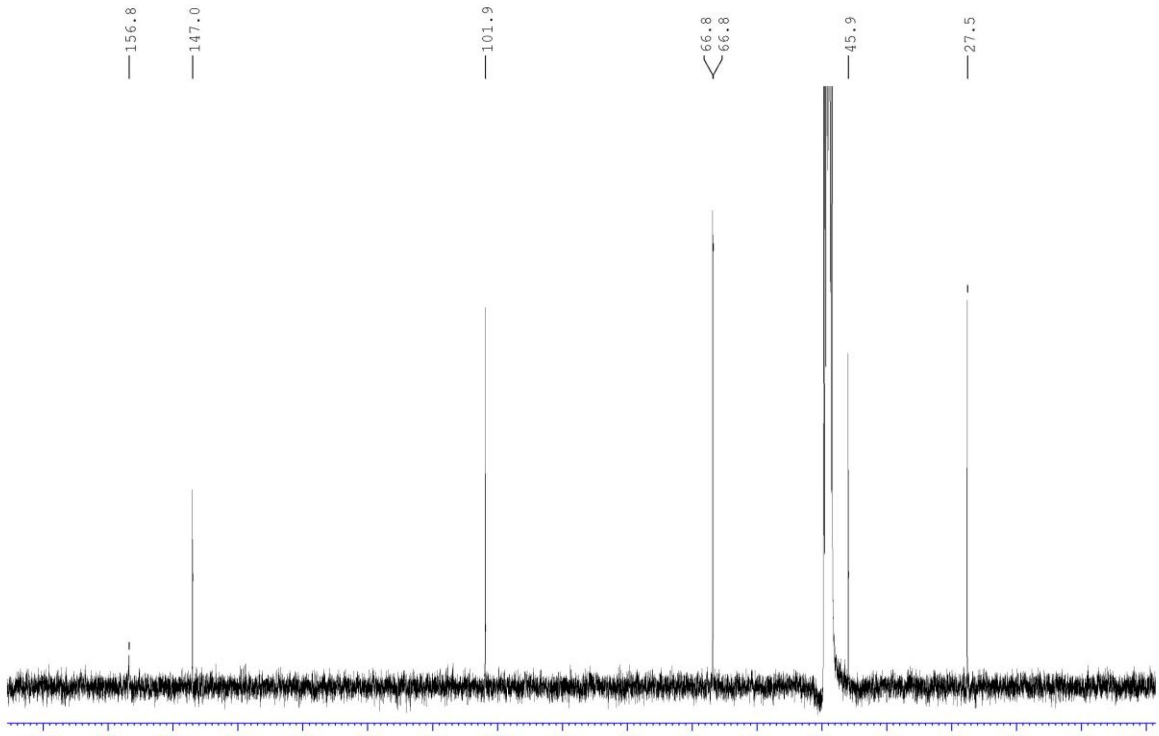

7,7-Difluoro-2-nitro-6,6a,7,7a-tetrahydro-5*H*-cyclopropa[*c*]pyrazolo[1,5-*a*]pyridine, **24**

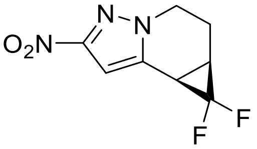

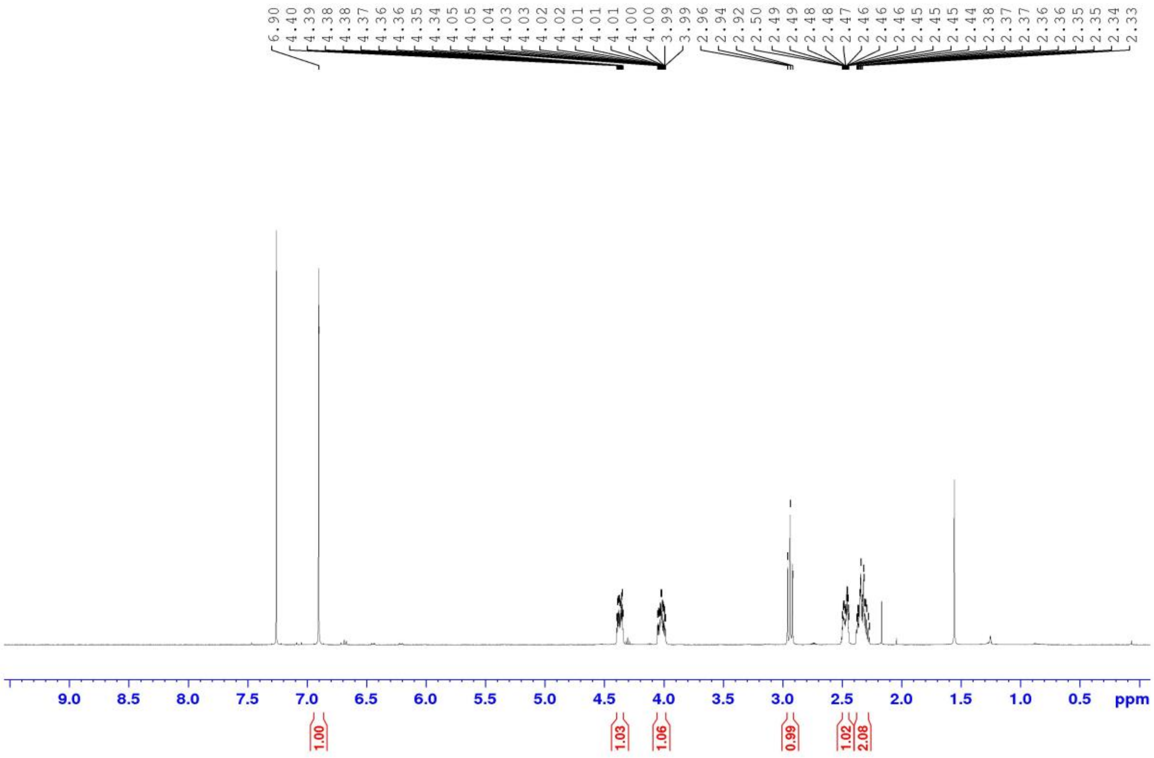

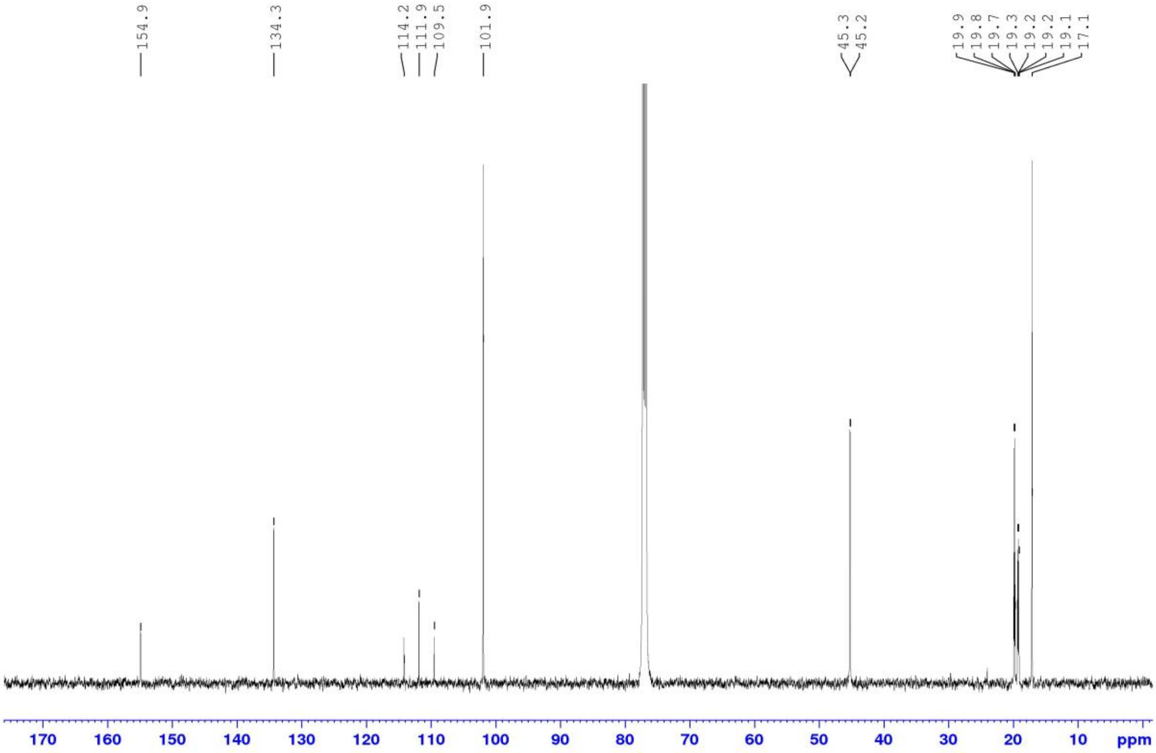

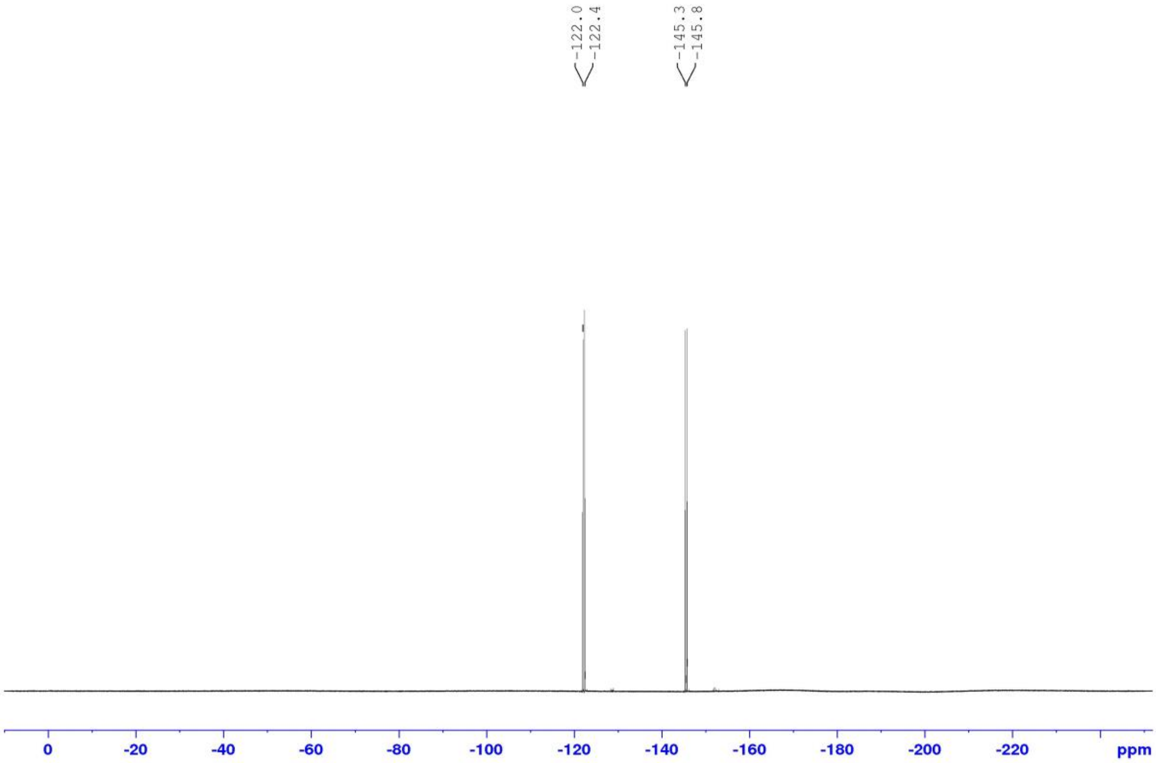

-122.0
-122.4
-145.3
-145.8
0
-20
-40
-60
-80
-100
-120
-140
-160
-180
-200
-220
ppm

trans-5-Bromo-4-hydroxy-2-nitro-4,5,6,7-tetrahydropyrazolo[1,5-*a*]pyridine, **25**

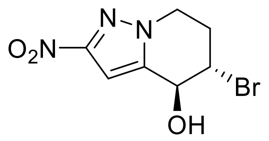

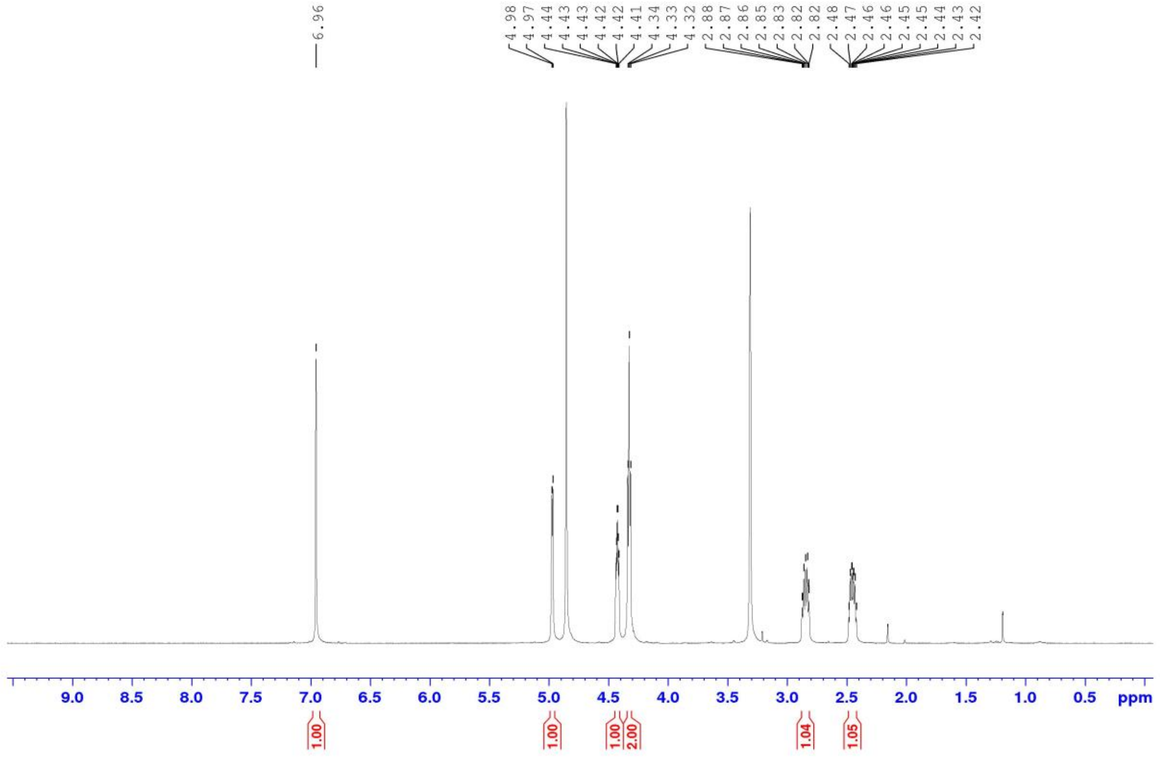

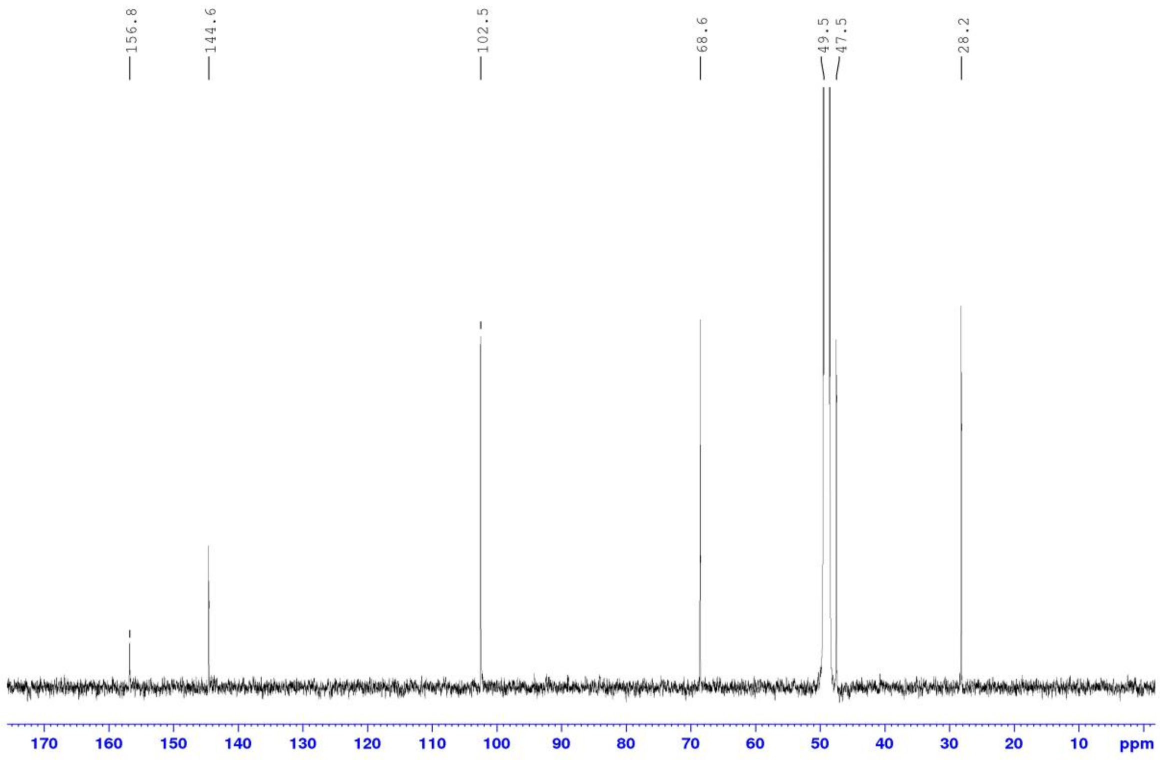

6-Nitro-1a,2,3,7b-tetrahydrooxireno[2,3-*c*]pyrazolo[1,5-*a*]pyridine, **26**

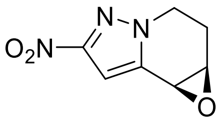

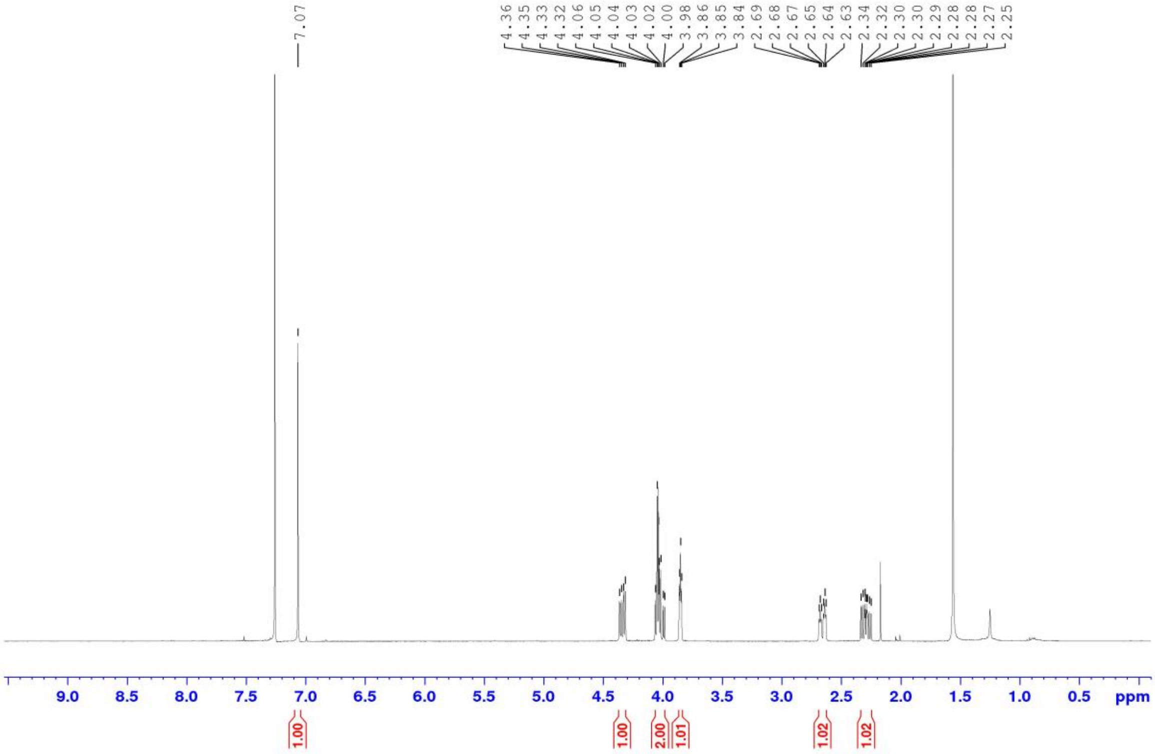

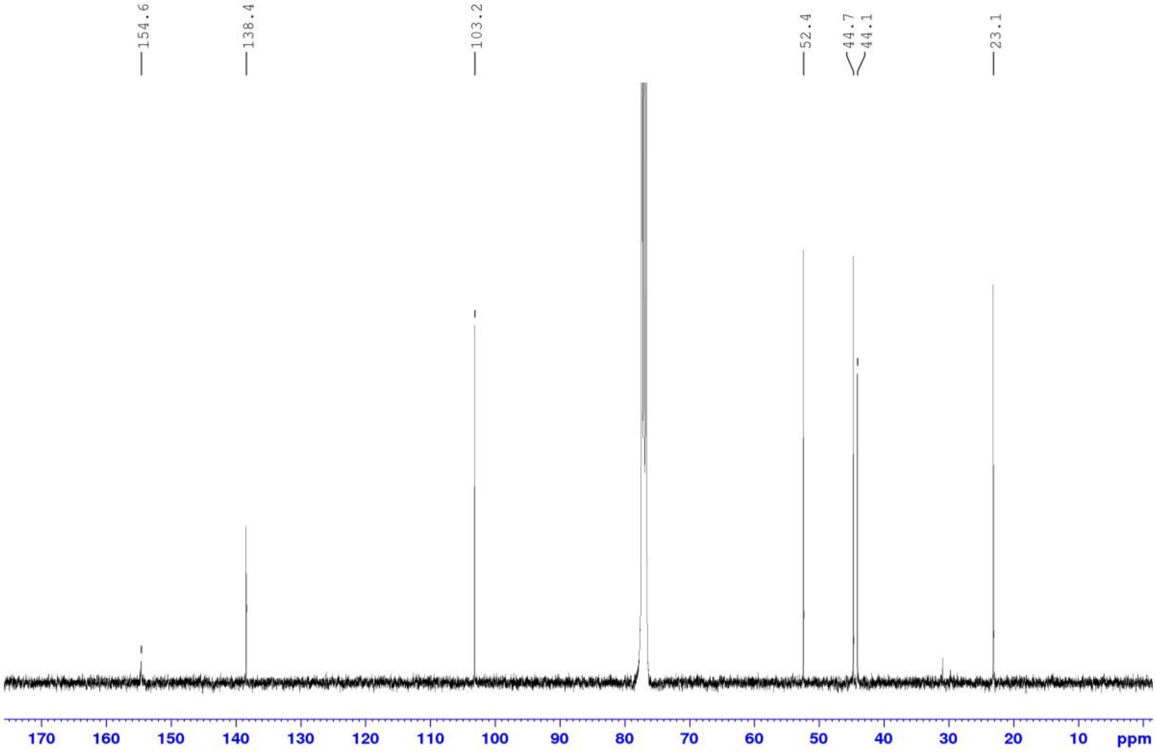

2-Nitro-4,5,6,7-tetrahydropyrazolo[1,5-*a*]pyridin-4-ol, **27**

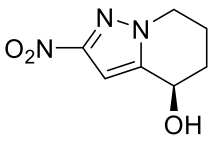

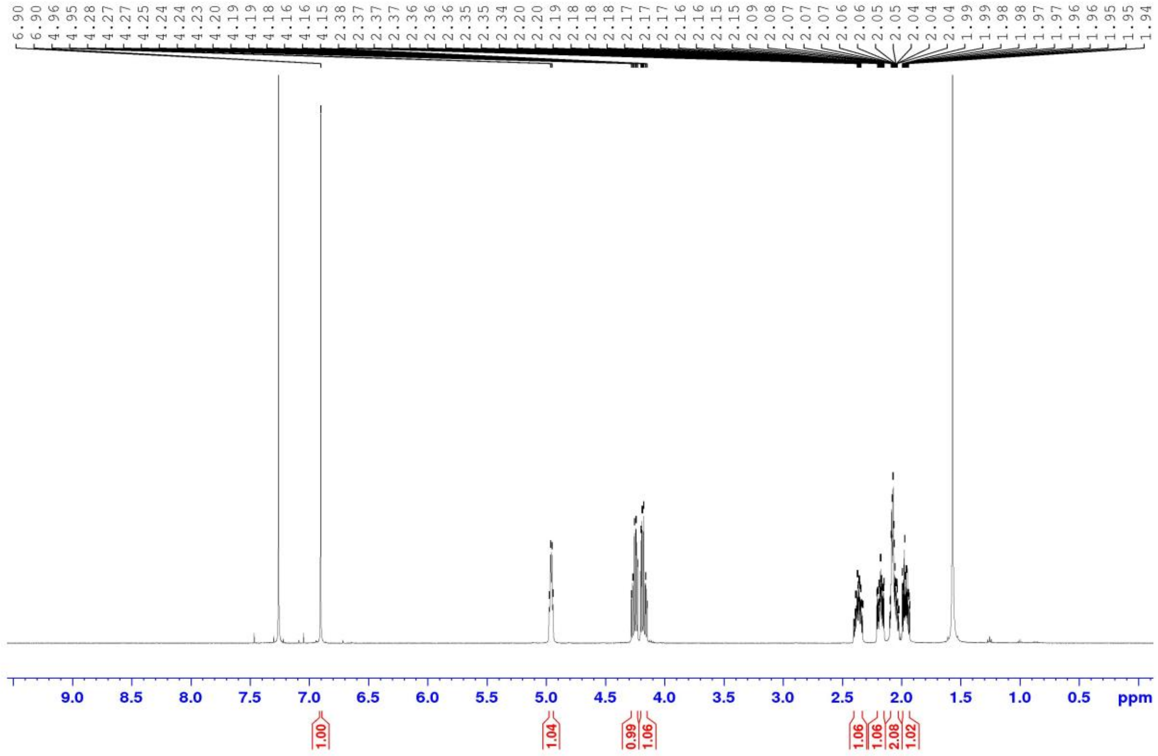

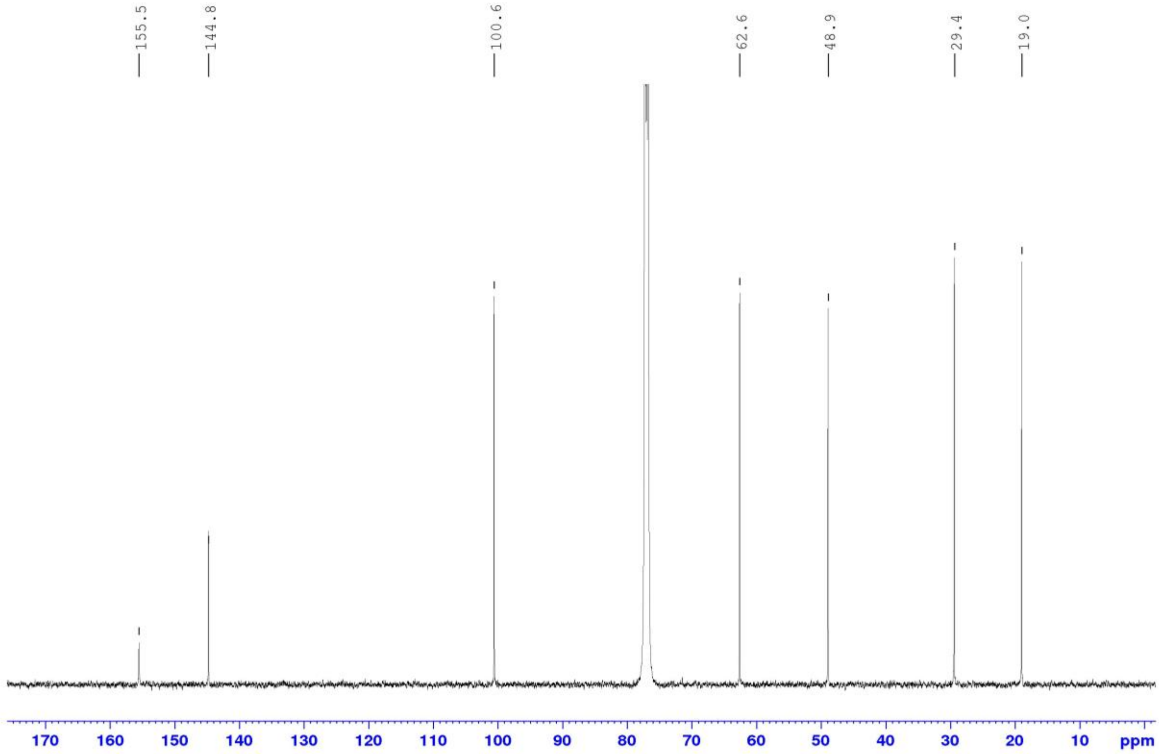

2-Nitro-5,6,7,8-tetrahydro-4*H*-pyrazolo[1,5-*a*]azepin-4-ol, **28**

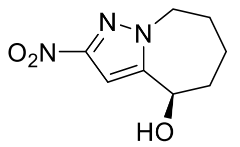

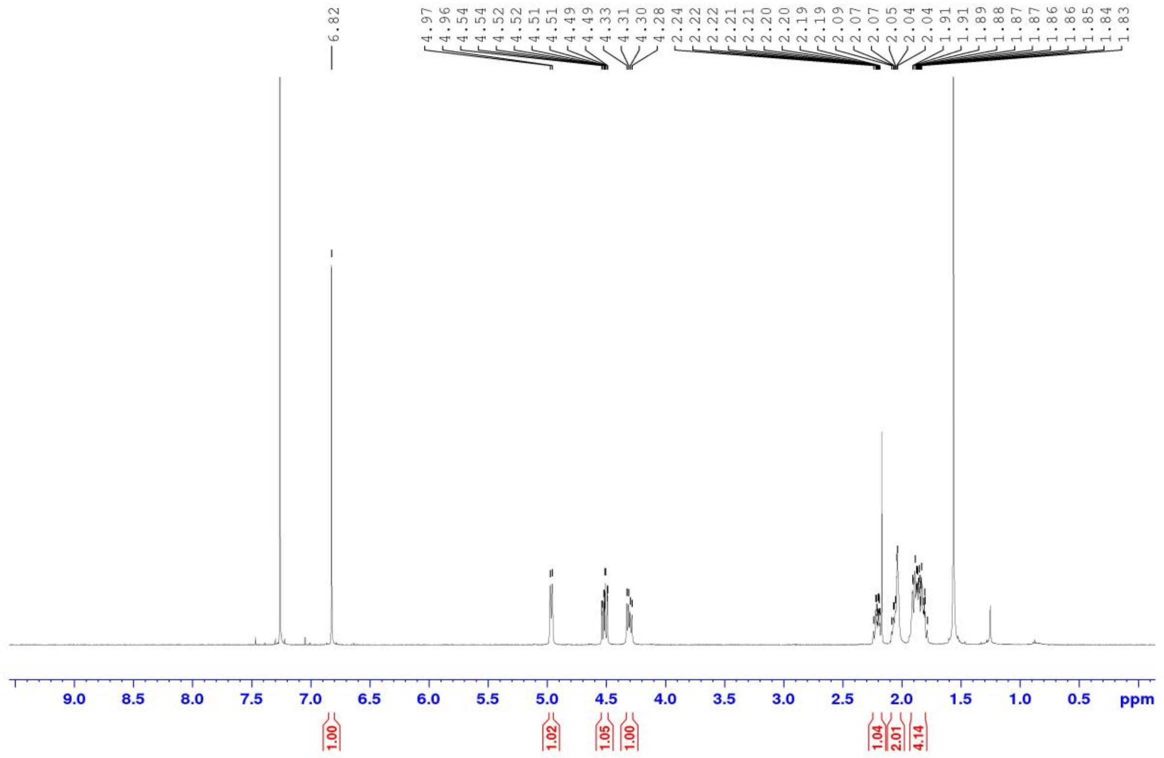

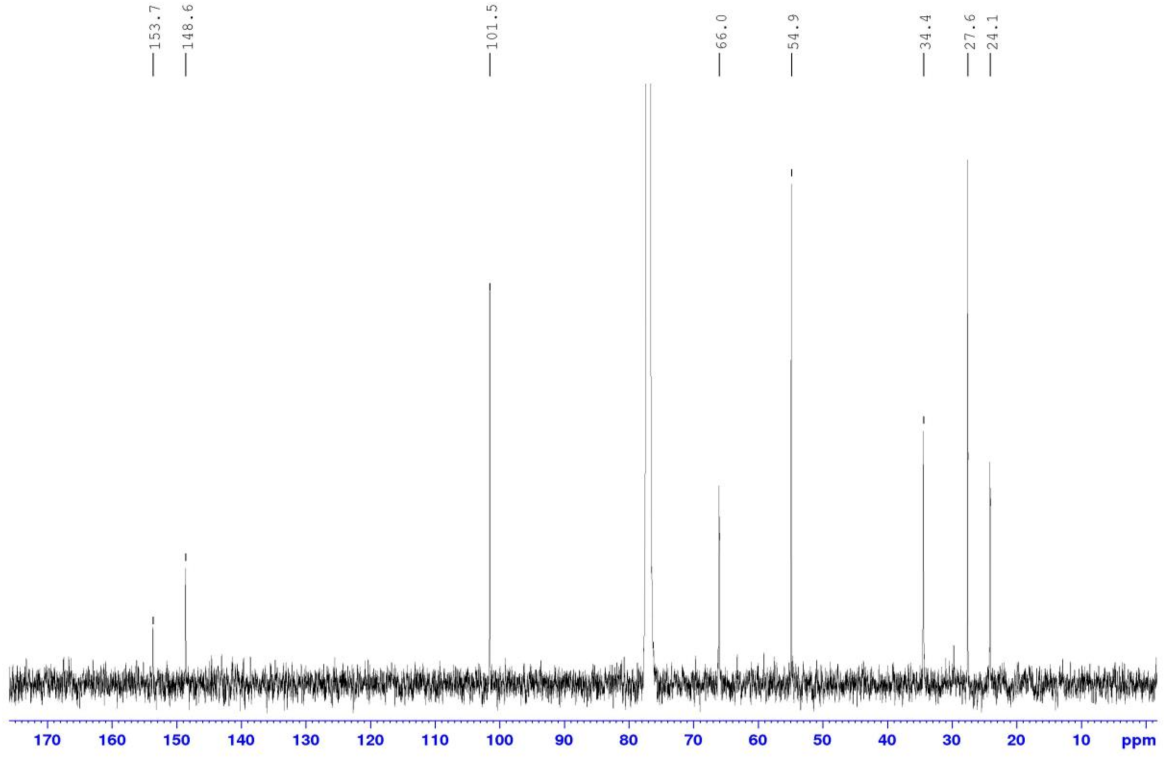

2-Amino-4,5,6,7-tetrahydropyrazolo[1,5-*a*]pyridin-4-ol, **29**

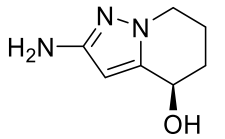

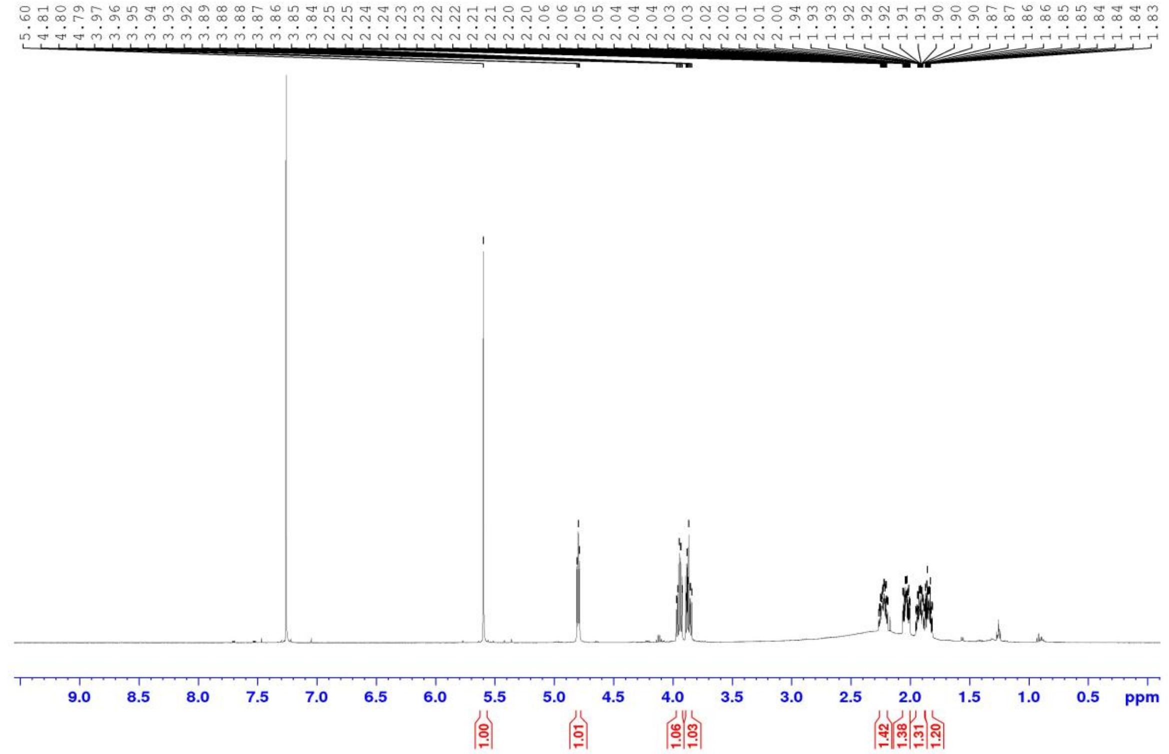

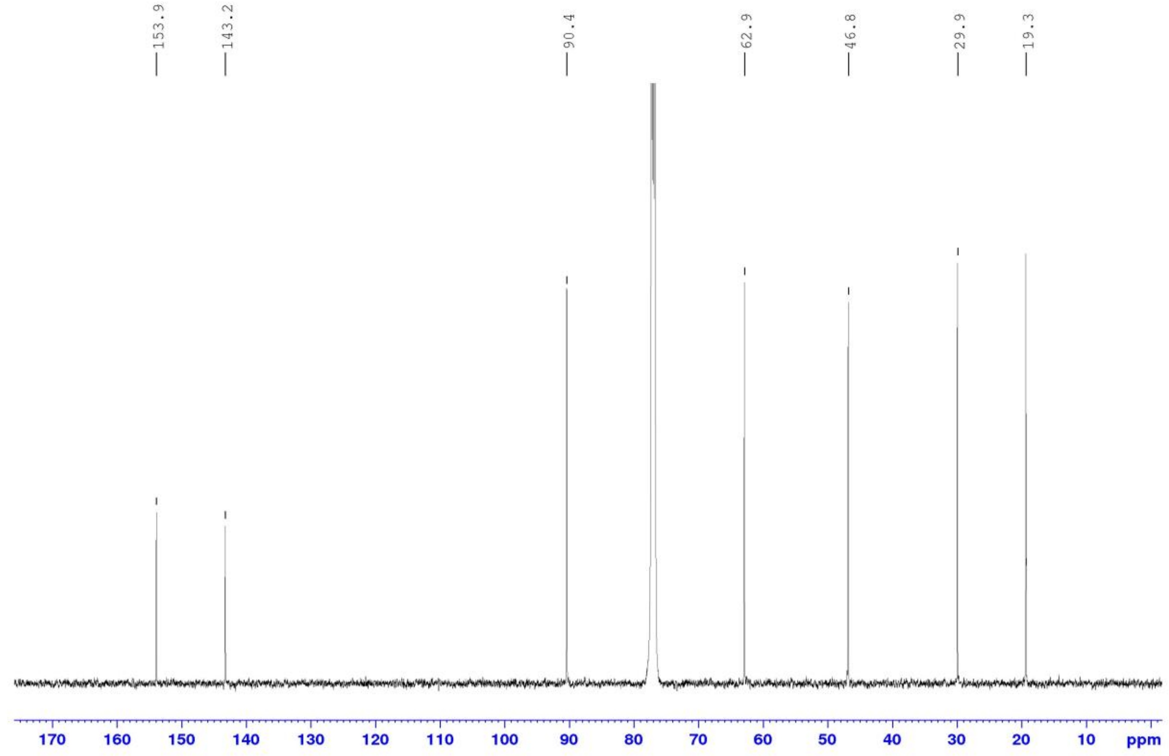

4-Bromo-2-nitro-4,5,6,7-tetrahydropyrazolo[1,5-*a*]pyridine, **30**

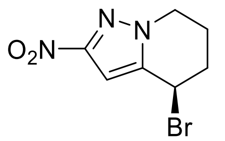

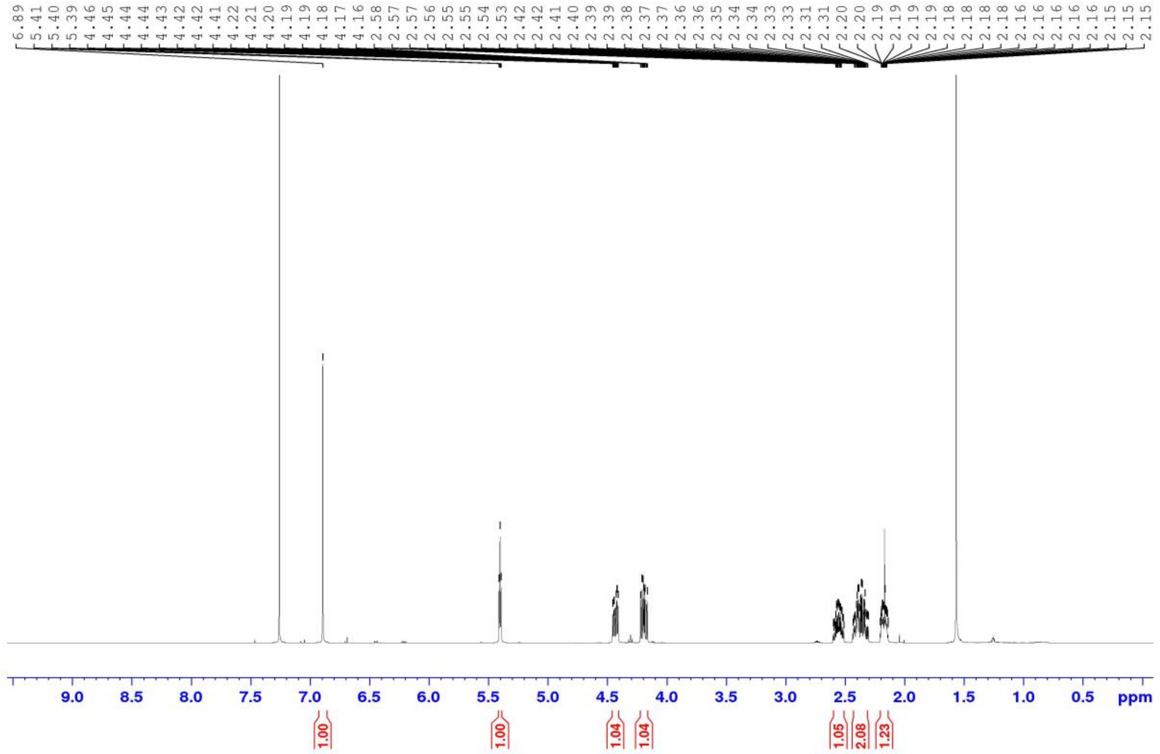

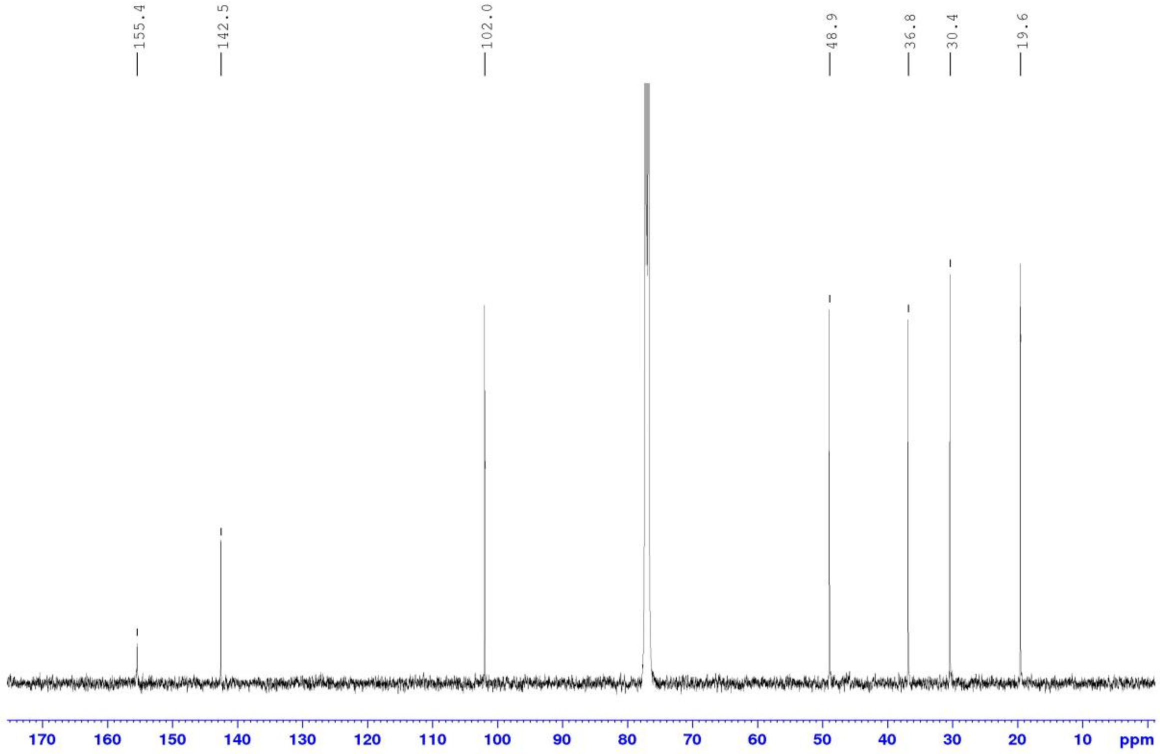

4-Fluoro-2-nitro-4,5,6,7-tetrahydropyrazolo[1,5-*a*]pyridine, **31**

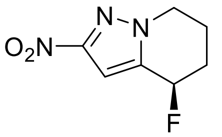

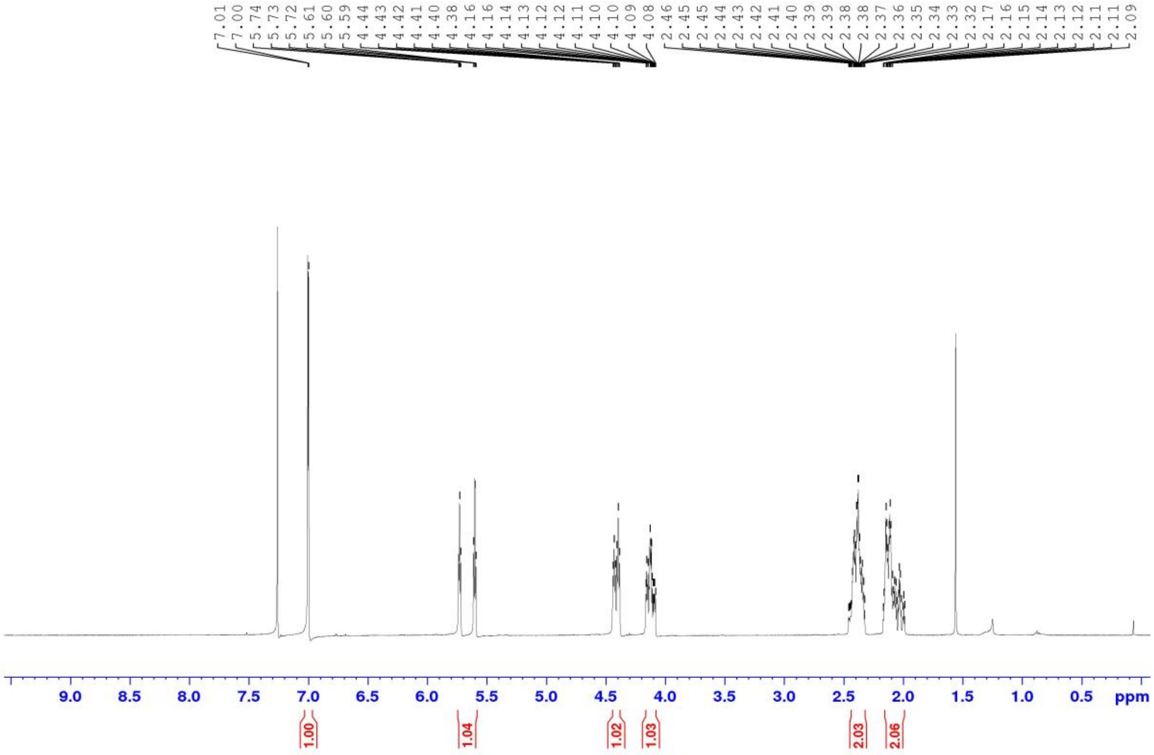

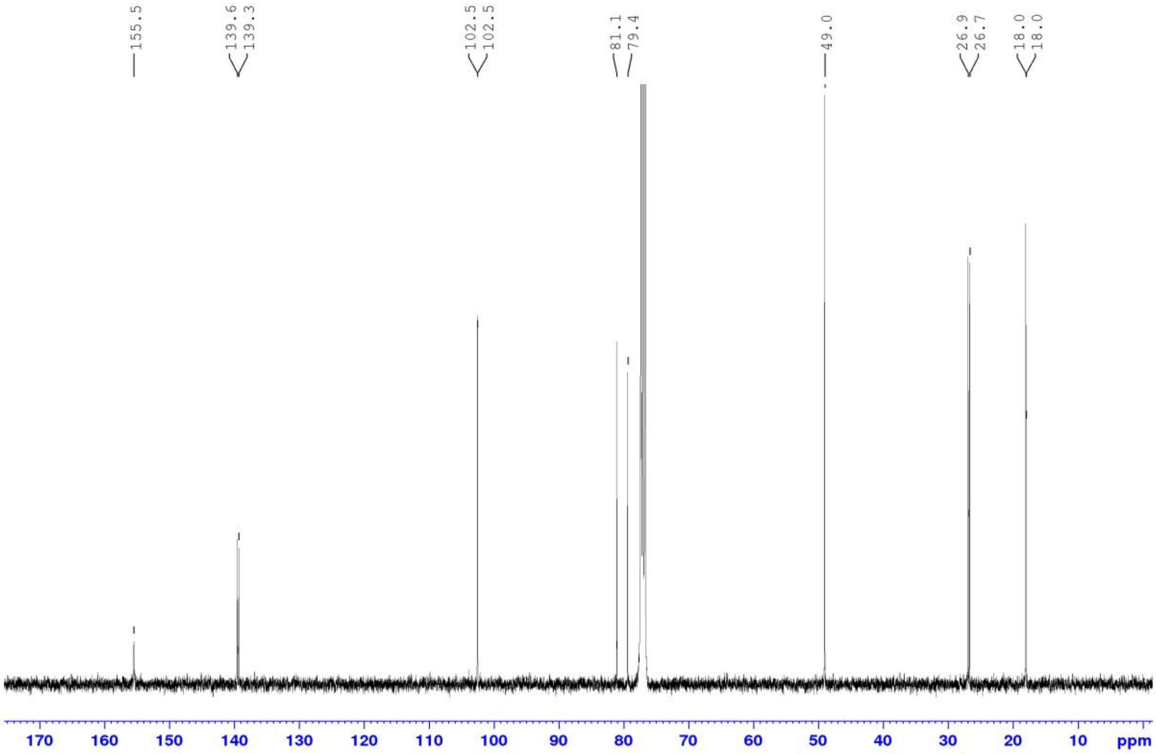

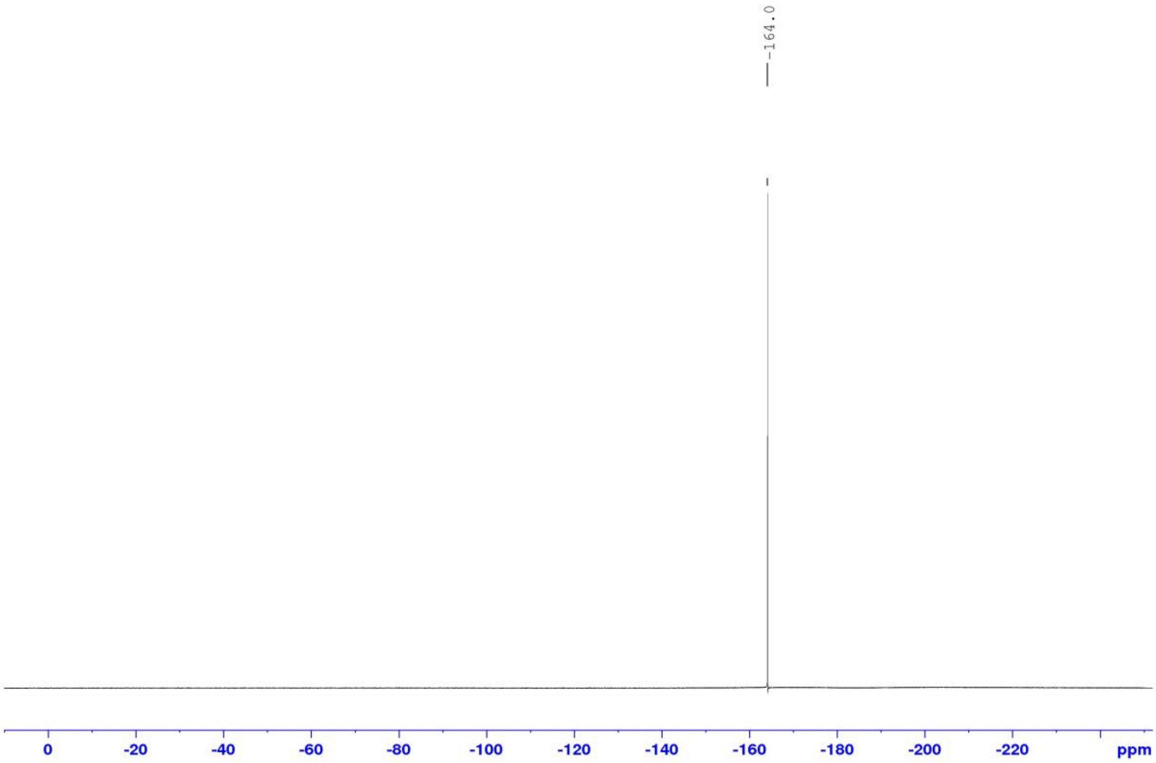

-164.0
0
-20
-40
-60
-80
-100
-120
-140
-160
-180
-200
-220
ppm

4-Azido-2-nitro-4,5,6,7-tetrahydropyrazolo[1,5-*a*]pyridine, **32**

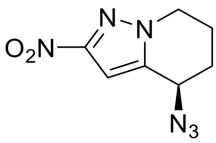

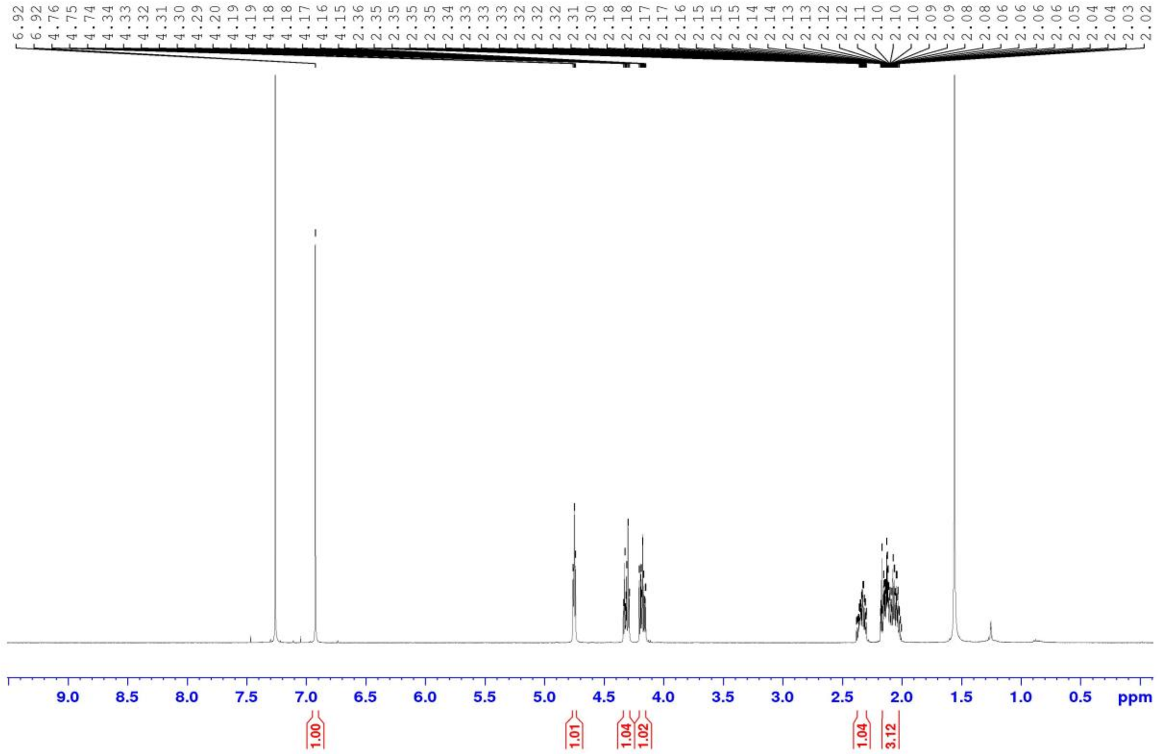

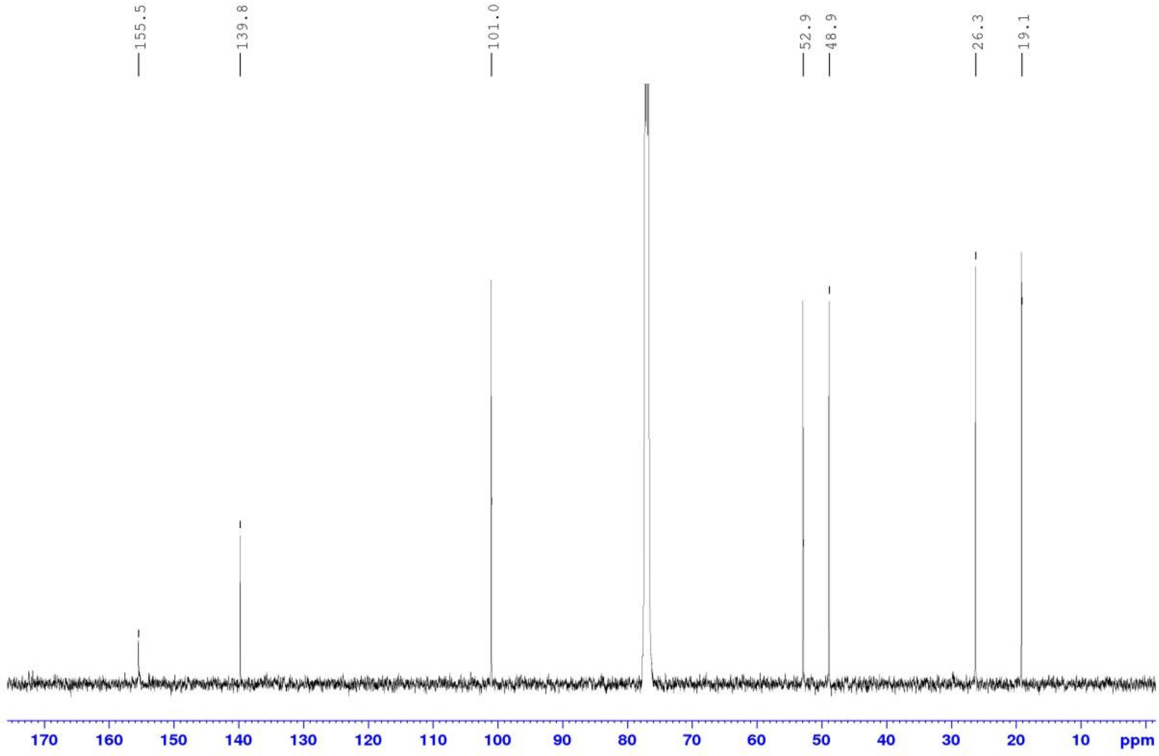

4,5,6,7-Tetrahydropyrazolo[1,5-*a*]pyridine-2,4-diamine, **33**

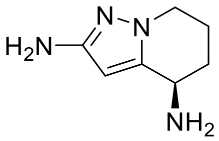

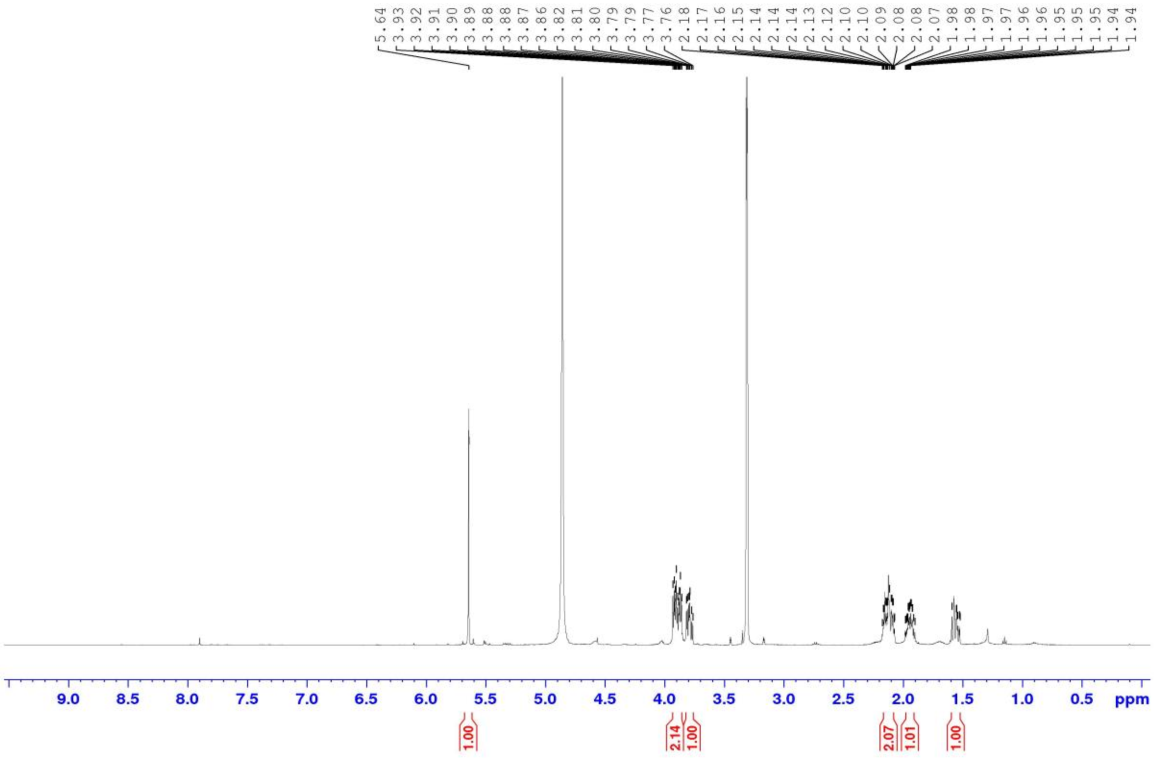

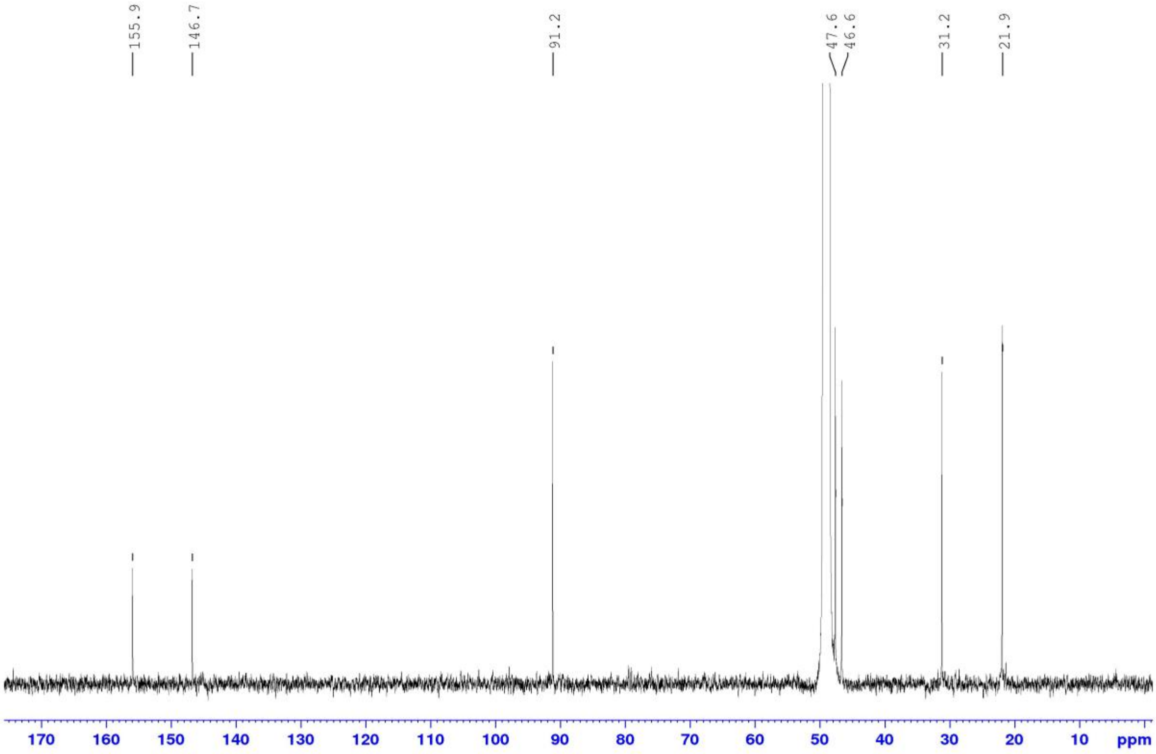

2-Amino-5,6,7,8-tetrahydro-4*H*-pyrazolo[1,5-*a*]azepin-4-ol, **34**

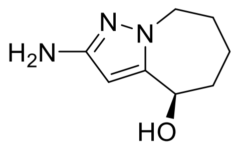

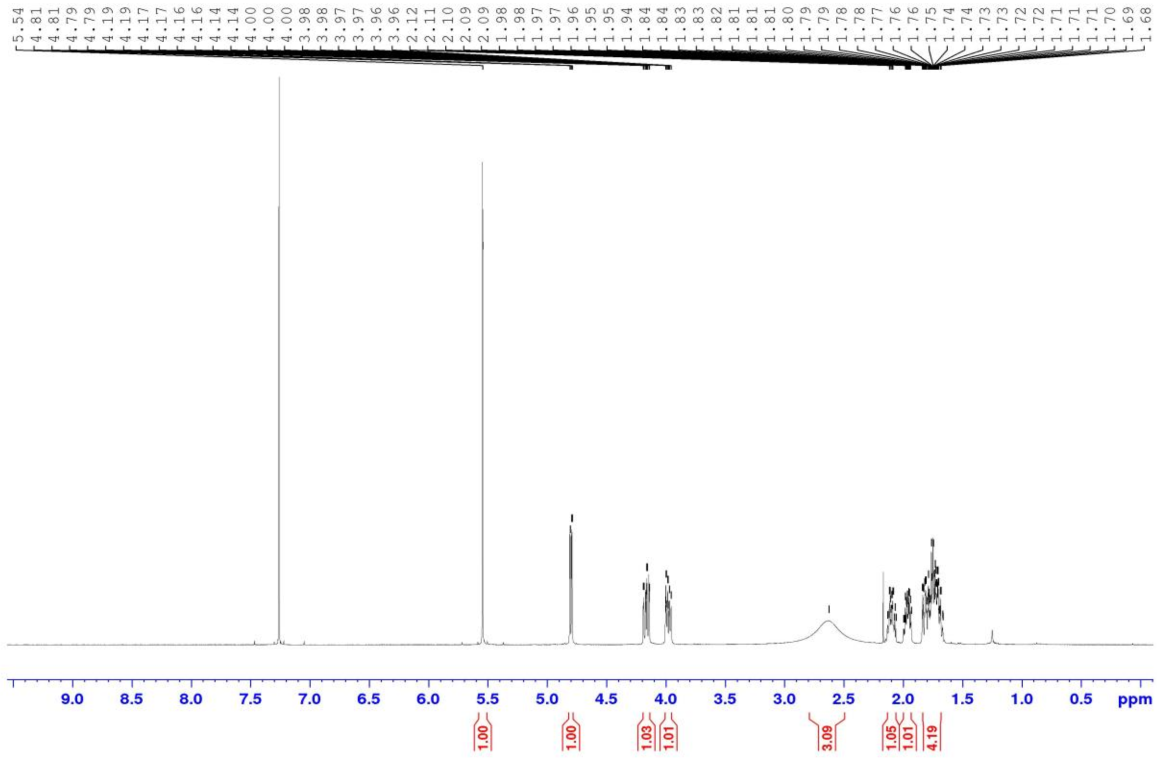

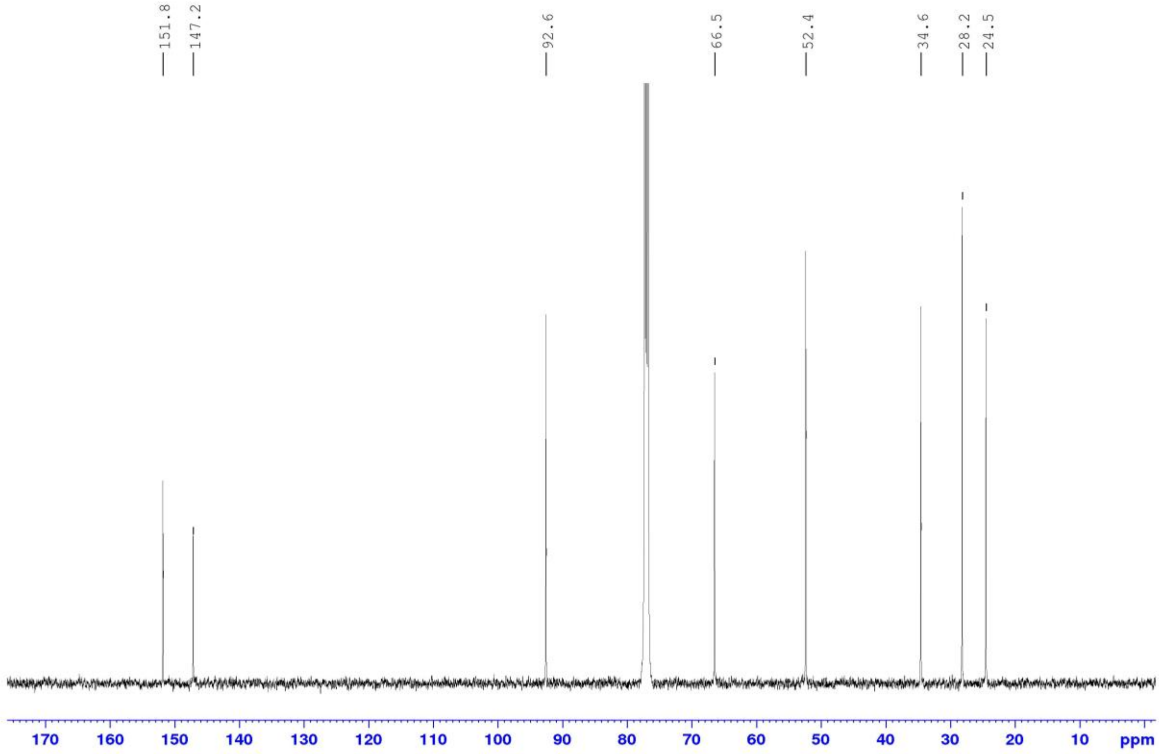

trans-4-Fluoro-2-nitro-4,5,6,7-tetrahydropyrazolo[1,5-*a*]pyridin-5-ol, **35**

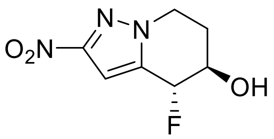

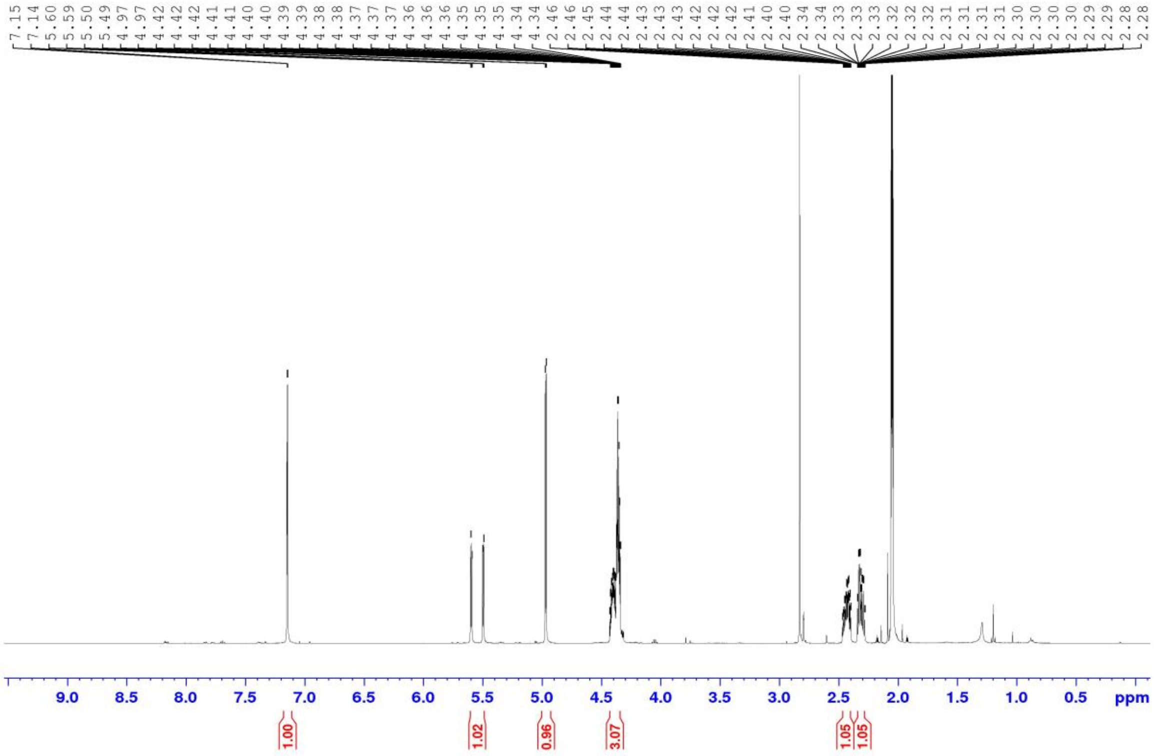

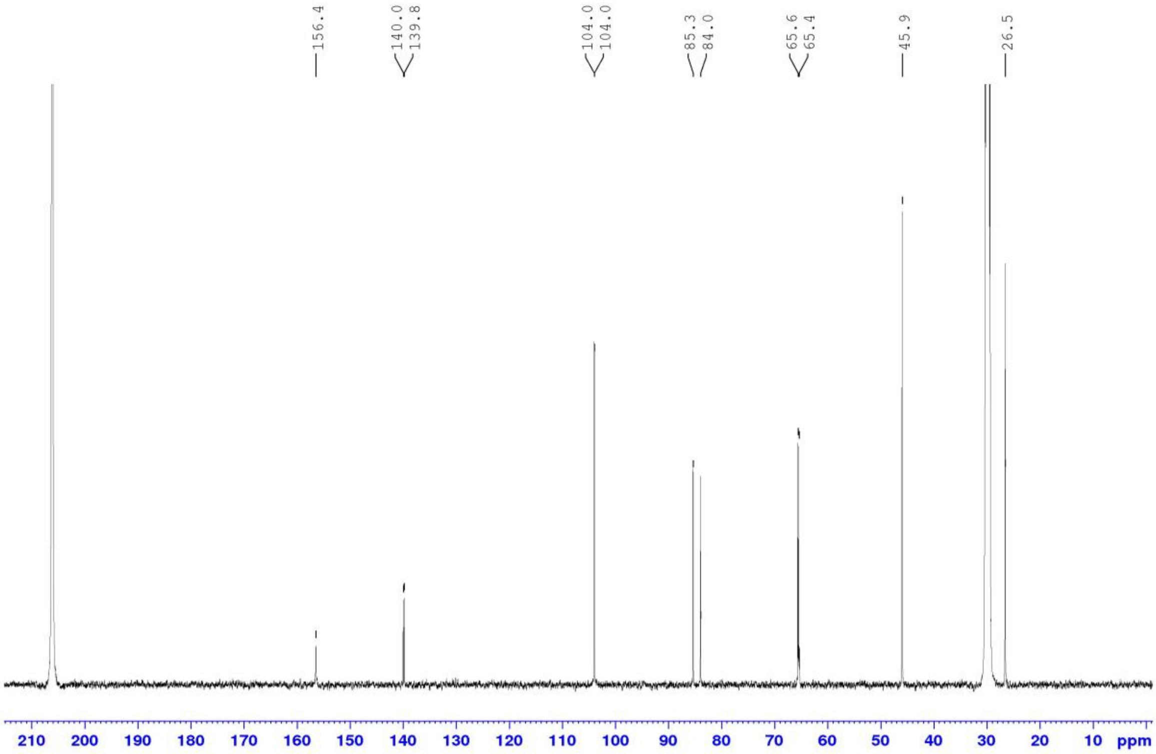

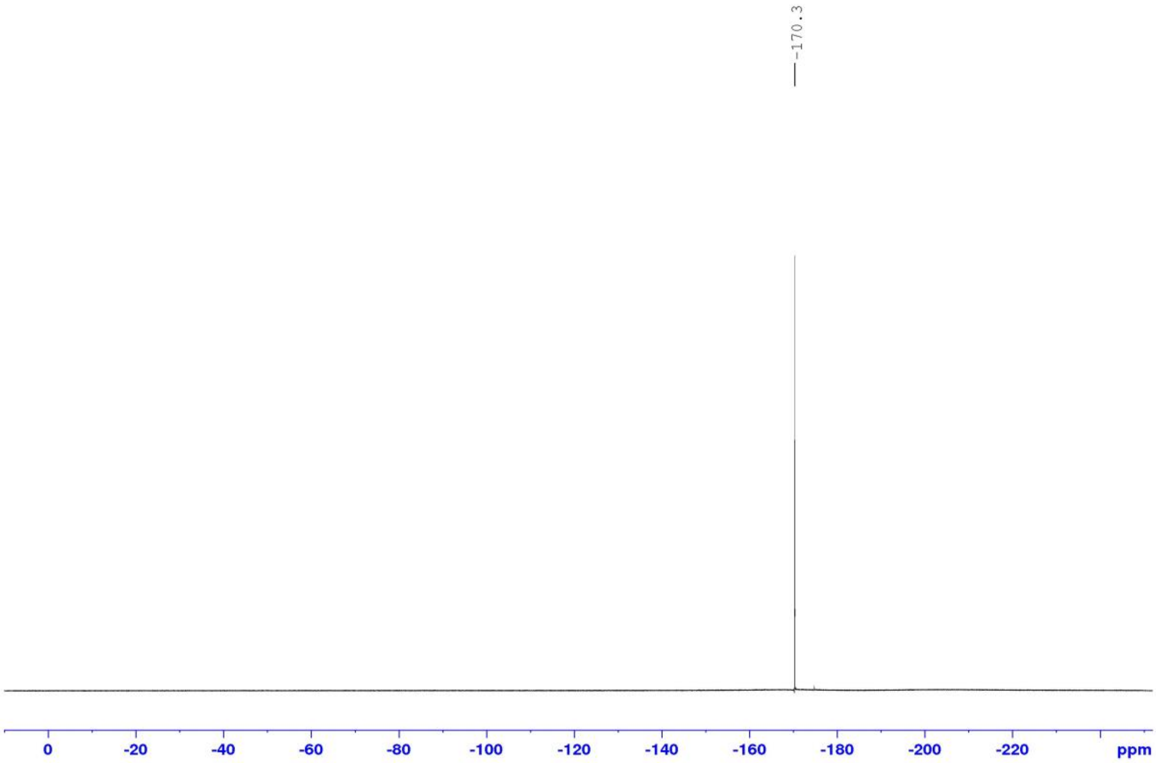
-170.3
0
-20
-40
-60
-80
-100
-120
-140
-160
-180
-200
-220
ppm

2-Nitro-4,5,6,7-tetrahydropyrazolo[1,5-*a*]pyridin-5-ol, **36**

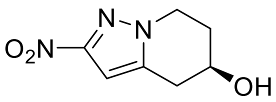

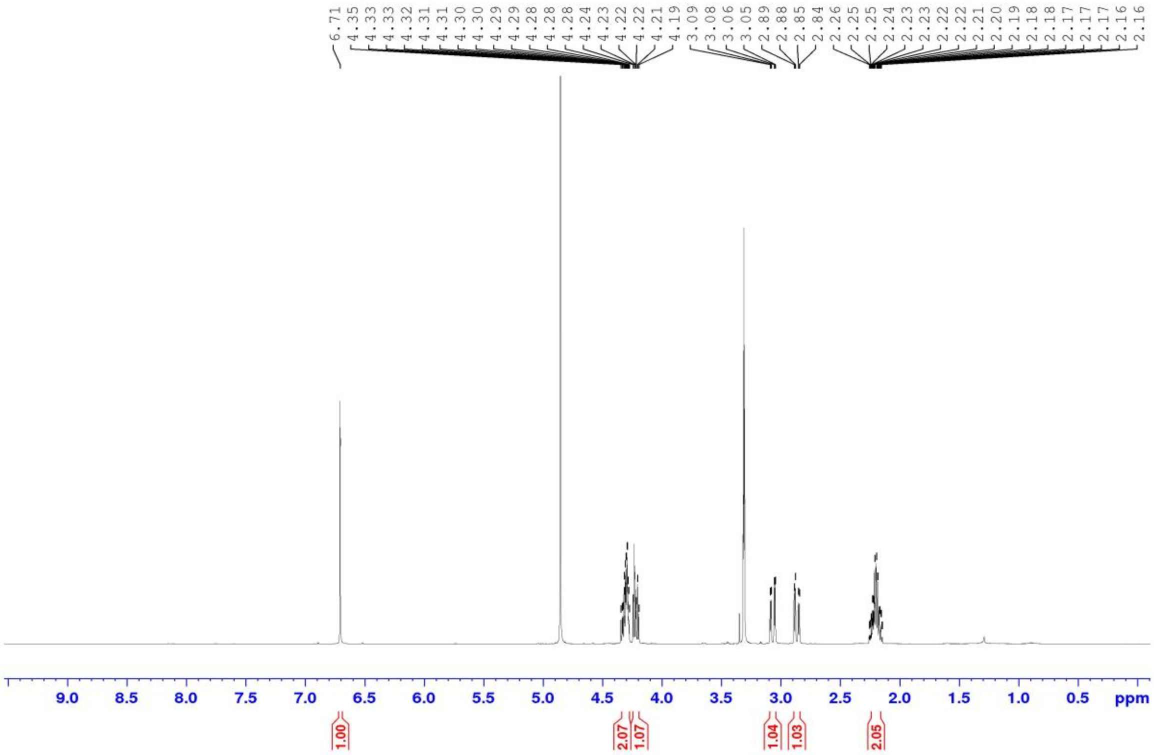

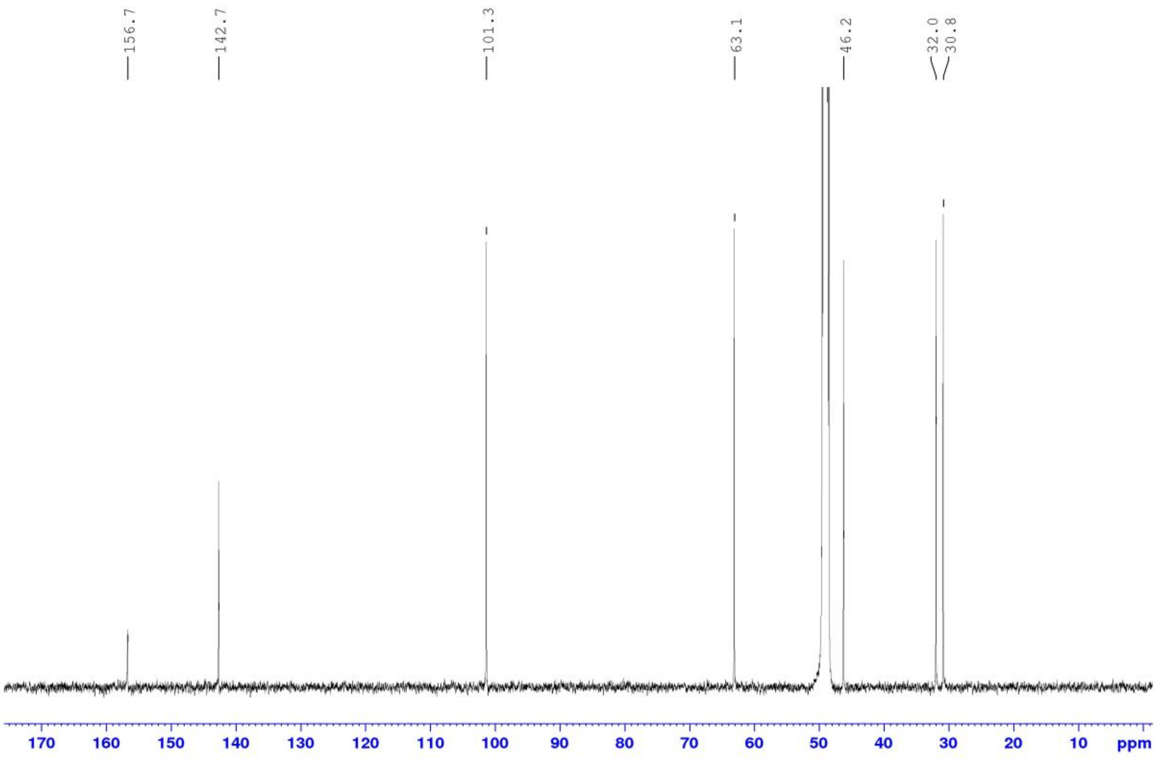

N,N-(bis-Boc)-5,6,7,8-tetrahydroisoquinolin-3-amine, **53a**

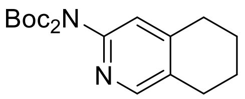

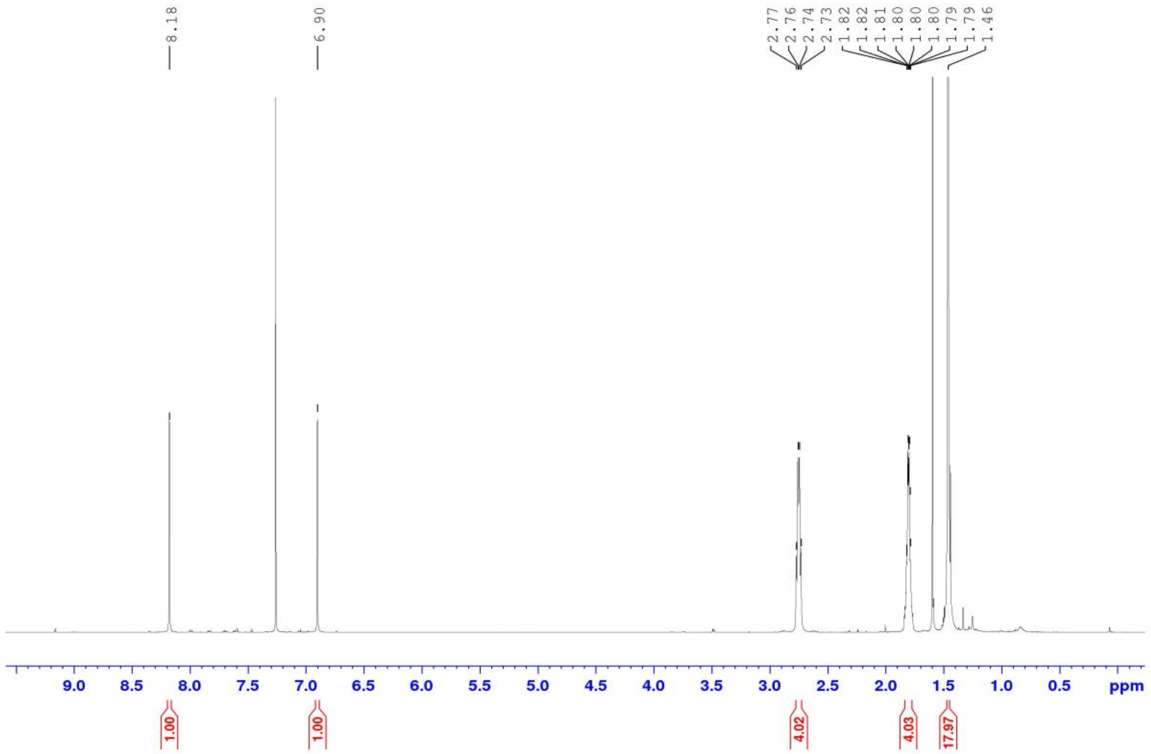

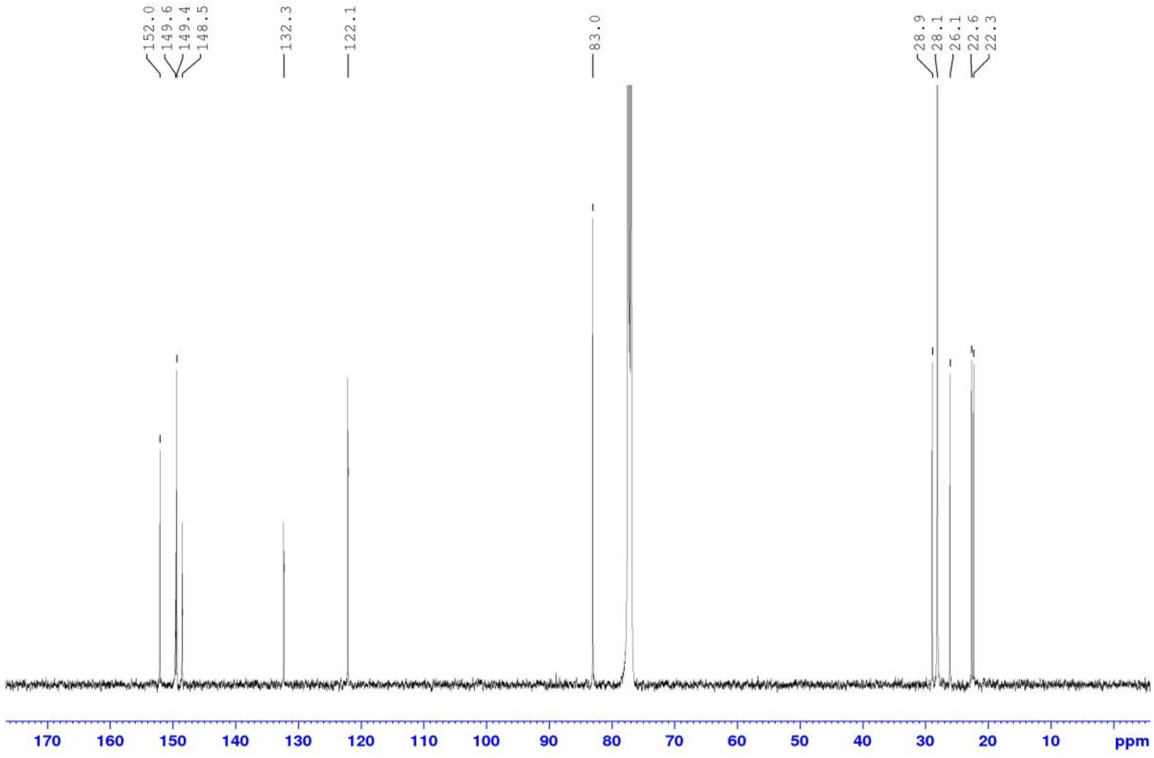

N,N-(bis-Boc)-7-methyl-5,6,7,8-tetrahydroisoquinolin-3-amine, **53b**

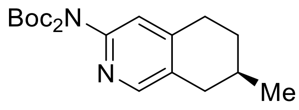

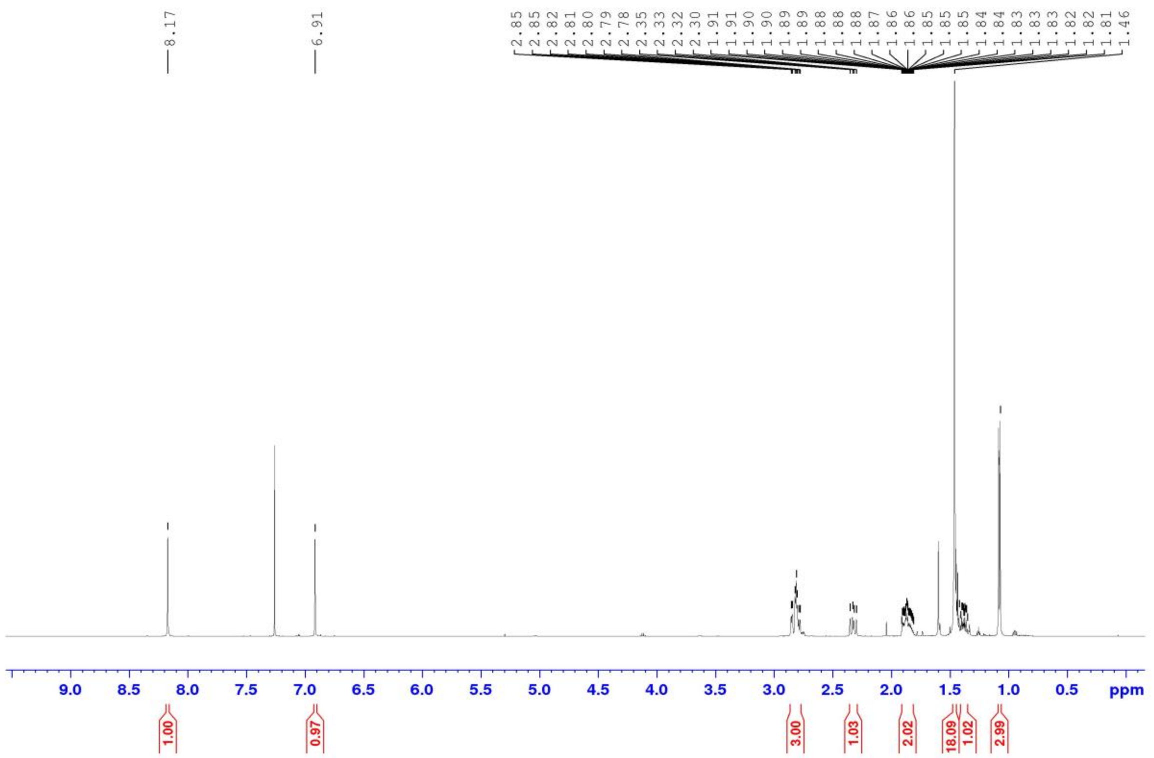

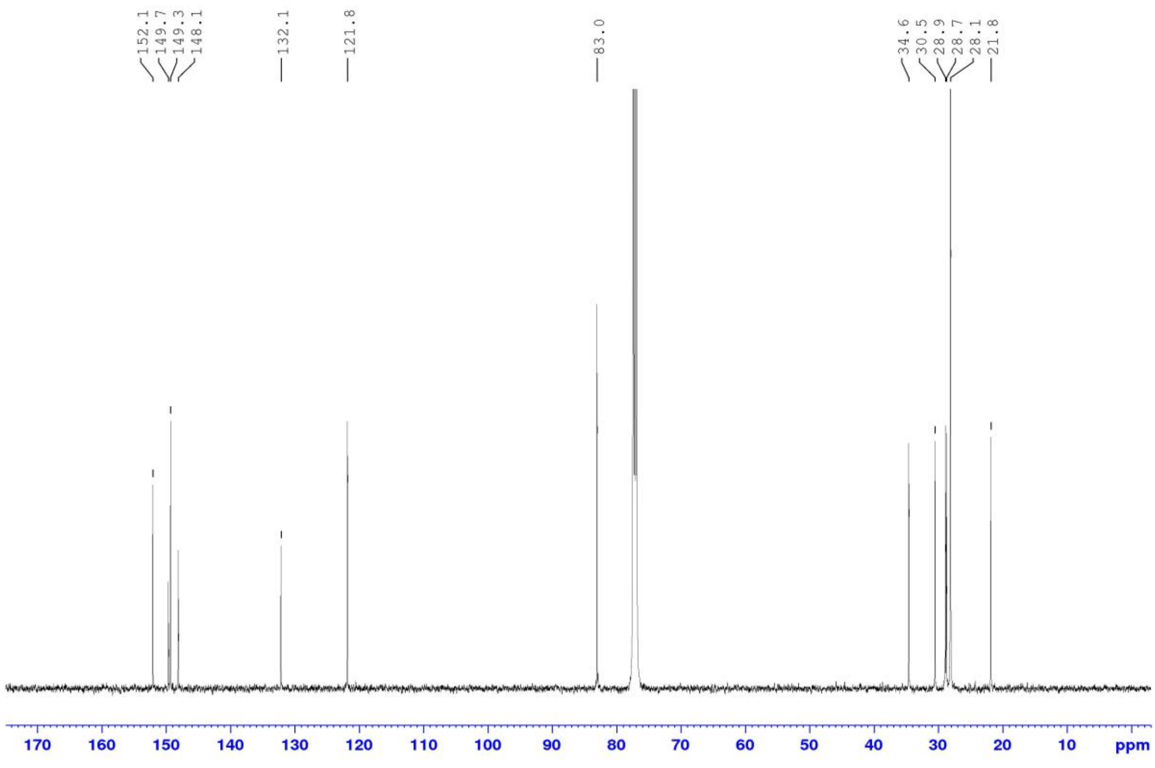

N,N-(bis-Boc)-7-trifluoromethyl-5,6,7,8-tetrahydroisoquinolin-3-amine, **53c**

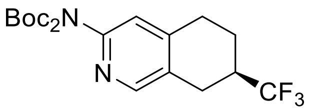

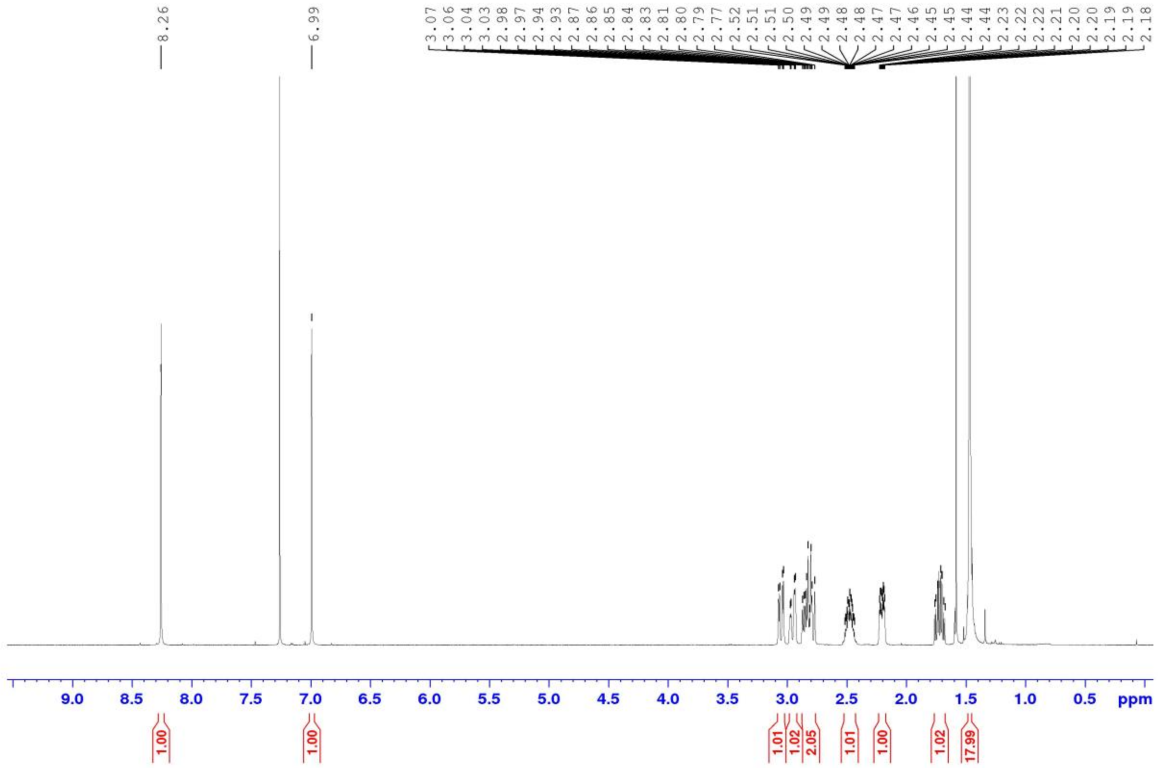

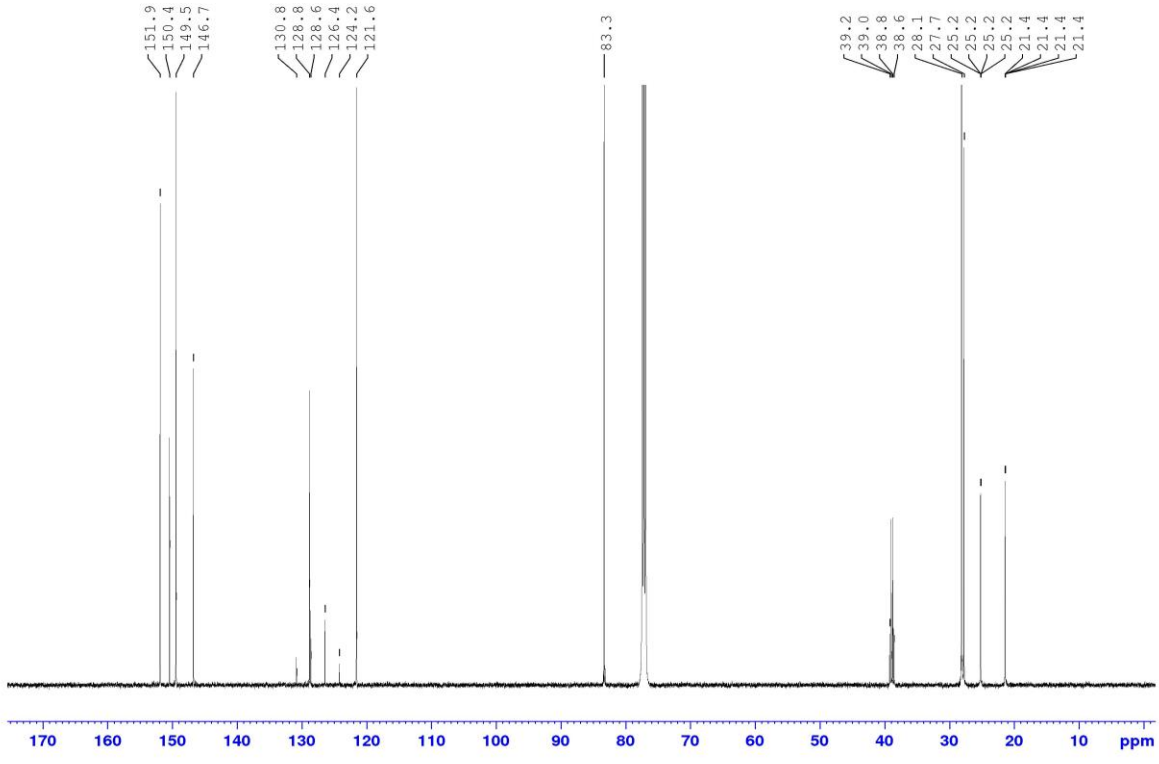

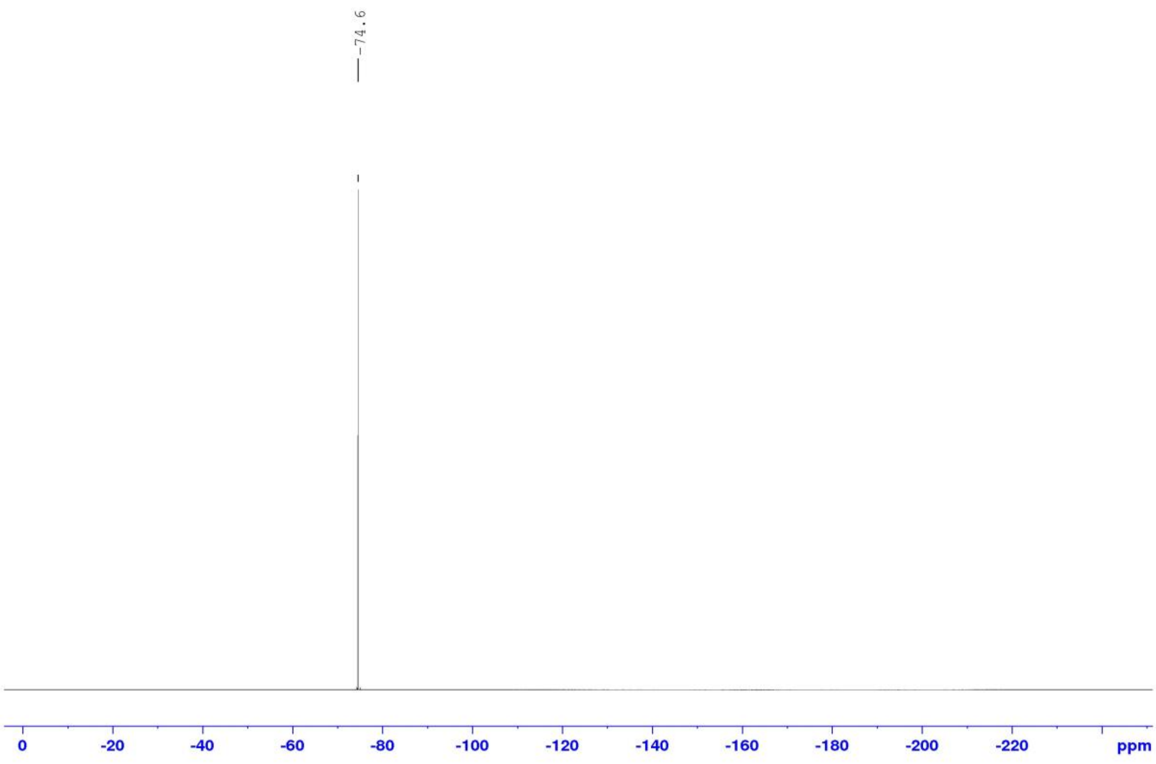
-74.6
0
-20
-40
-60
-80
-100
-120
-140
-160
-180
-200
-220
ppm

N,N-(bis-Boc)-8-methyl-5,6,7,8-tetrahydroisoquinolin-3-amine, **53d**

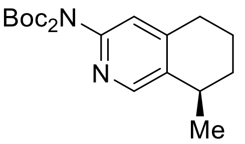

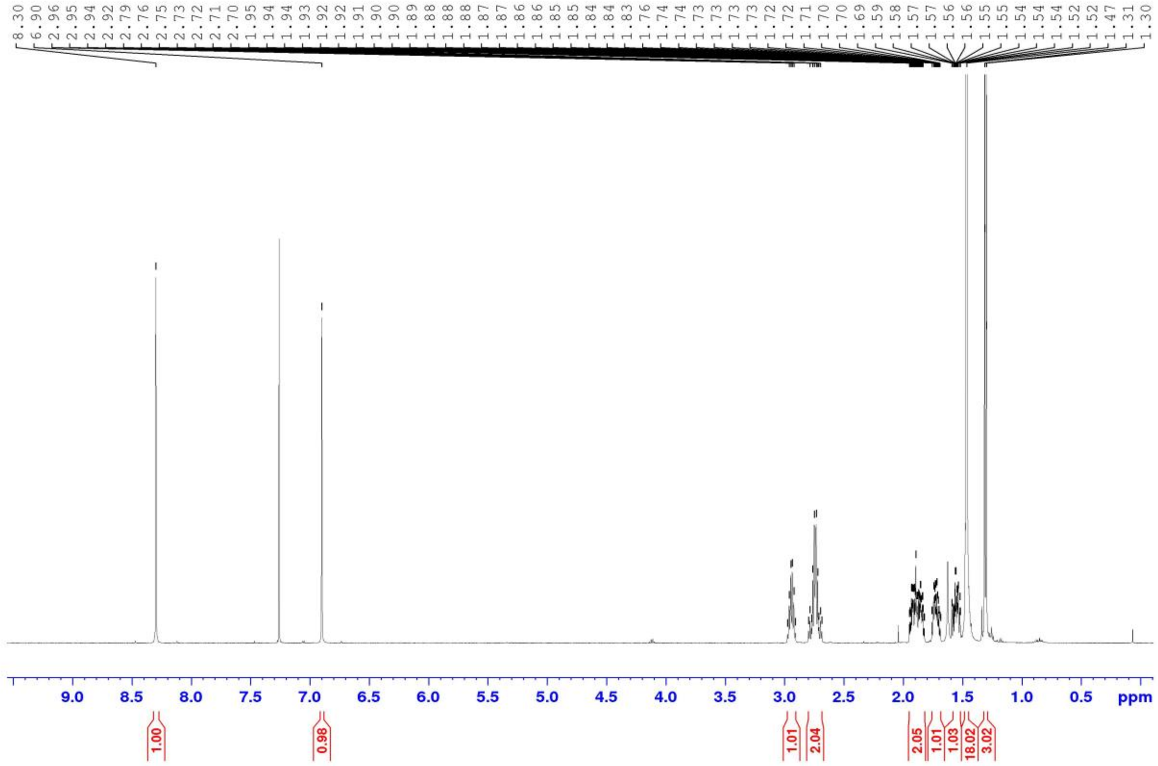

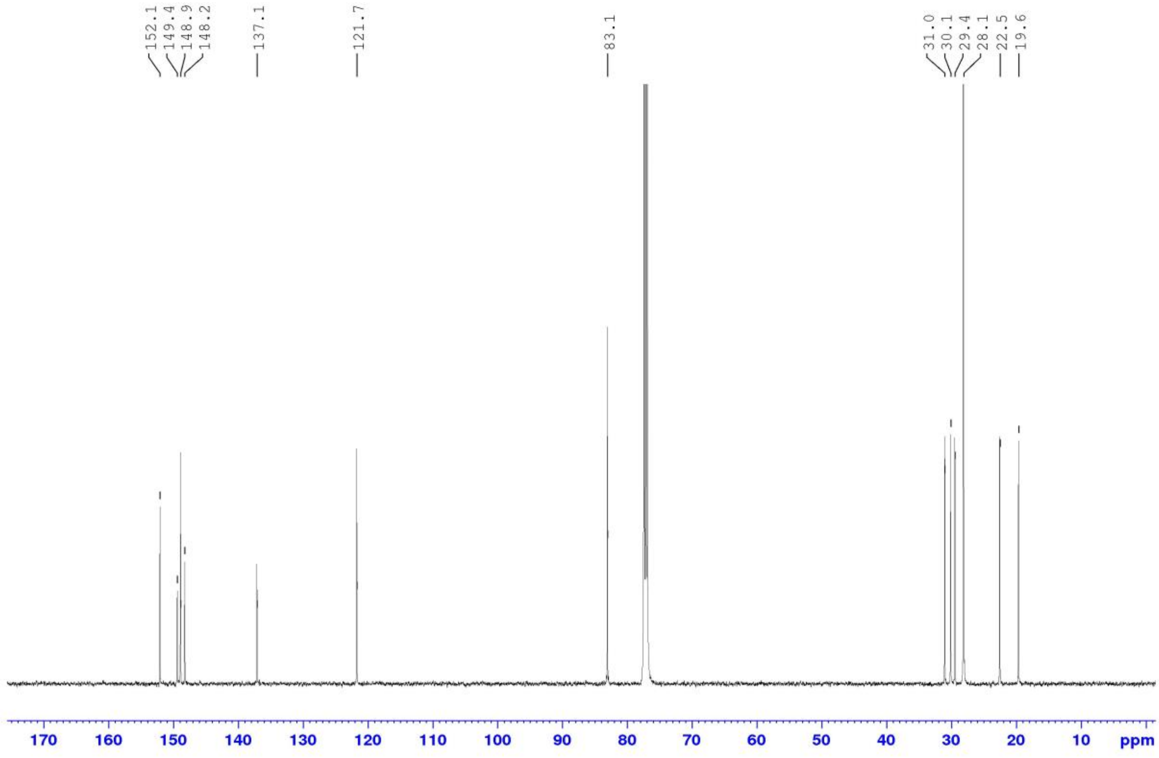

N,N-(bis-Boc)-8-phenyl-5,6,7,8-tetrahydroisoquinolin-3-amine, **53e**

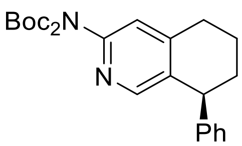

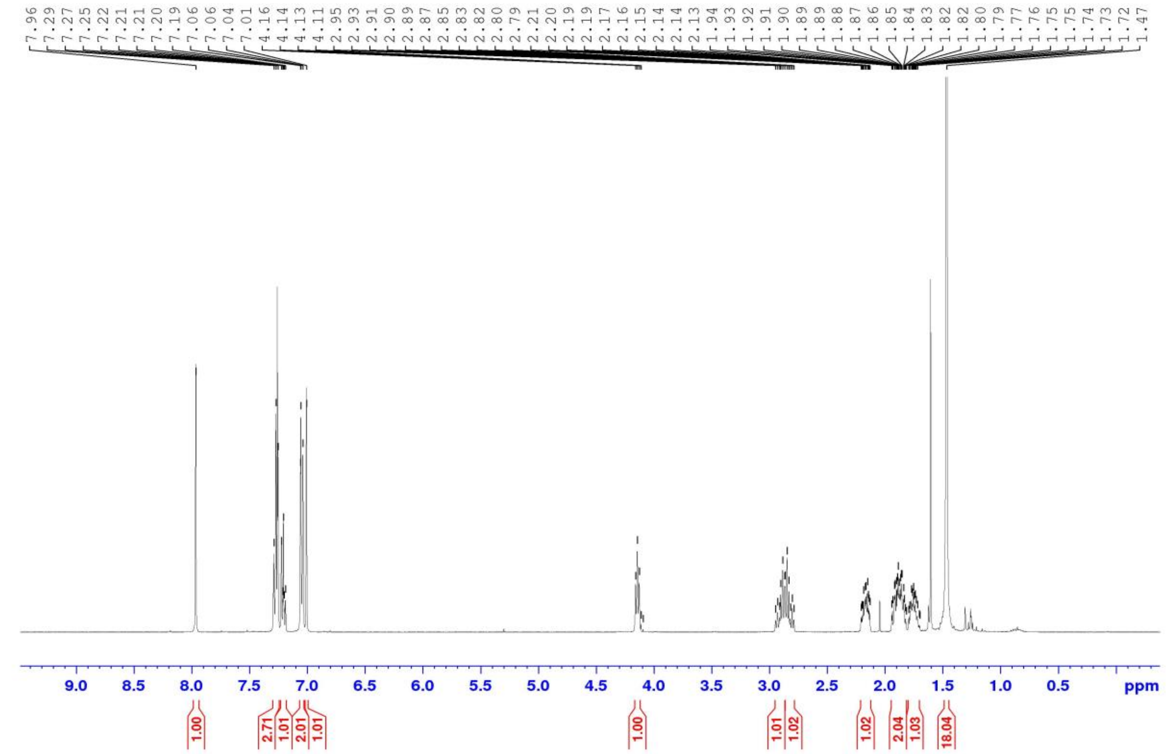

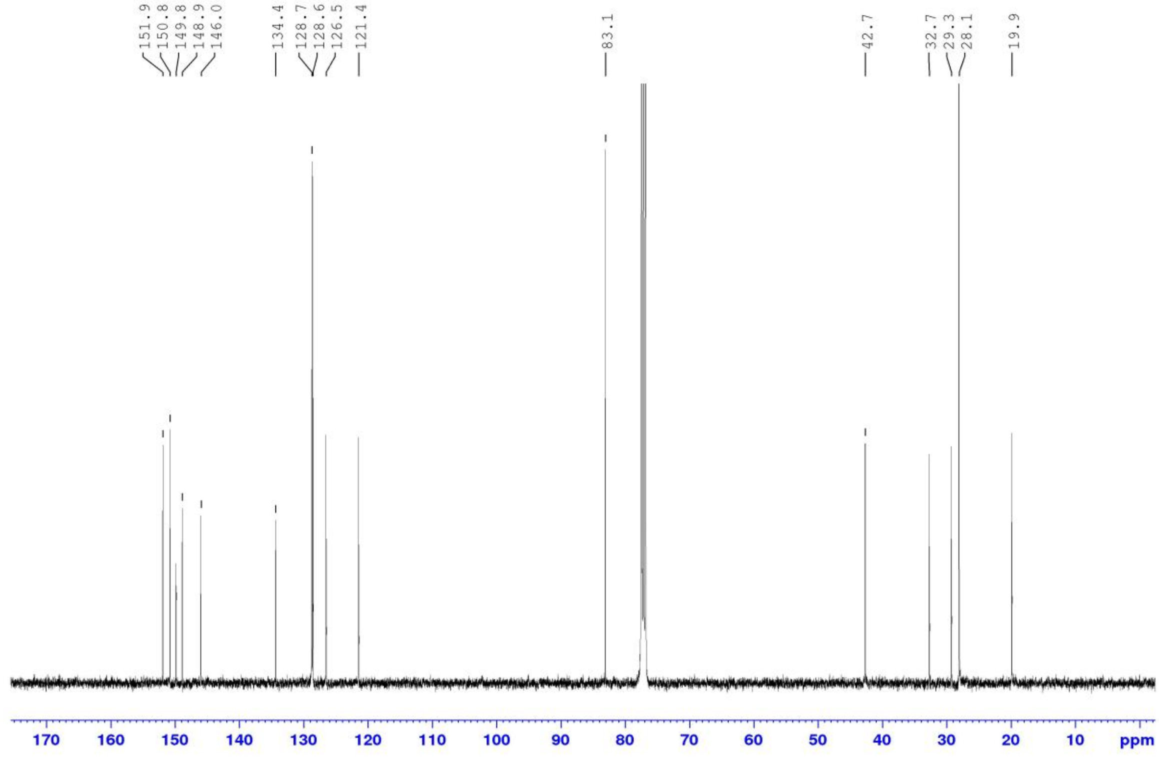

N,N-(bis-Boc)-6,7,8,9-tetrahydro-5*H*-cyclohepta[*c*]pyridin-3-amine, **53f**

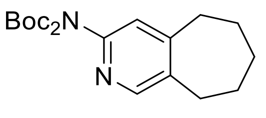

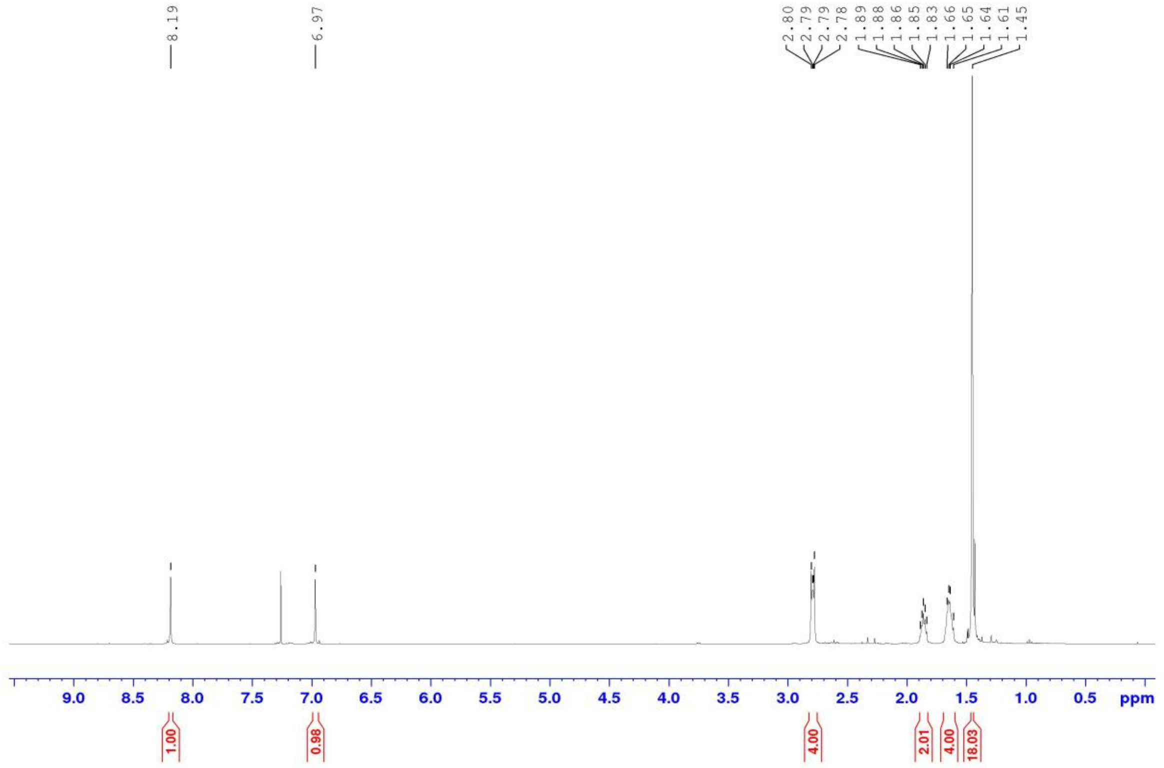

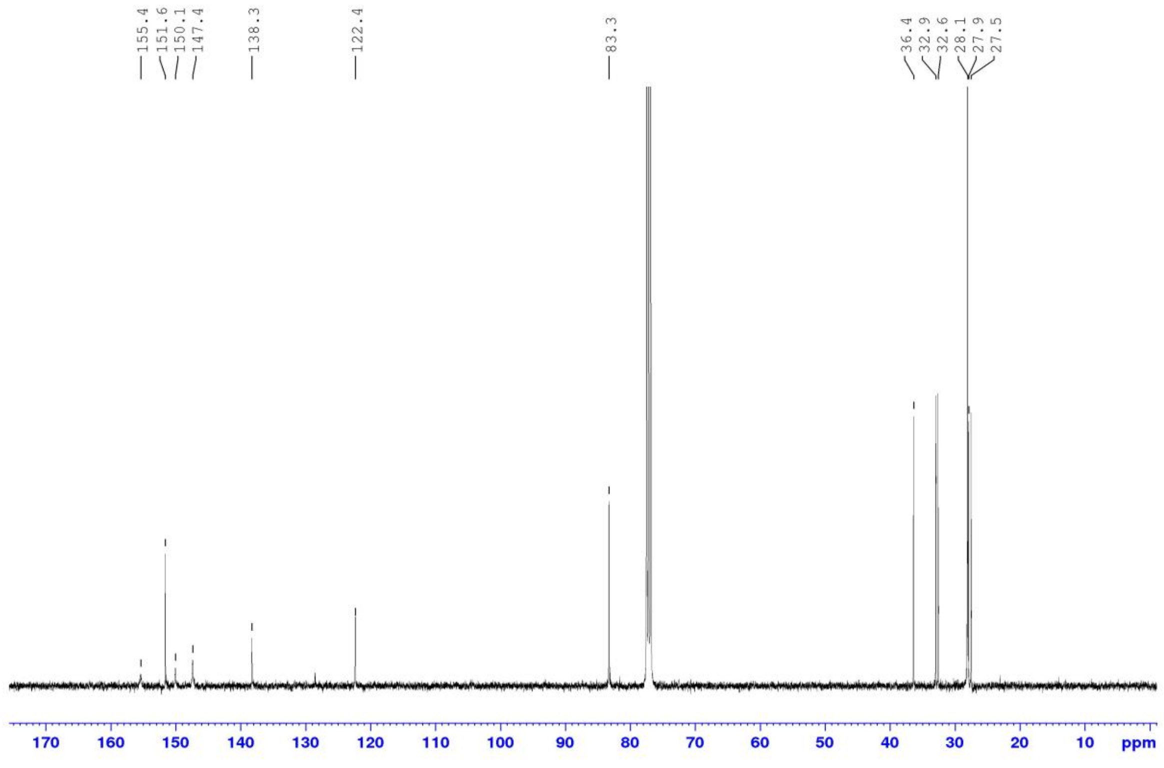

5,6,7,8-tetrahydroisoquinolin-3-amine hydrochloride, **37a**

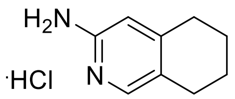

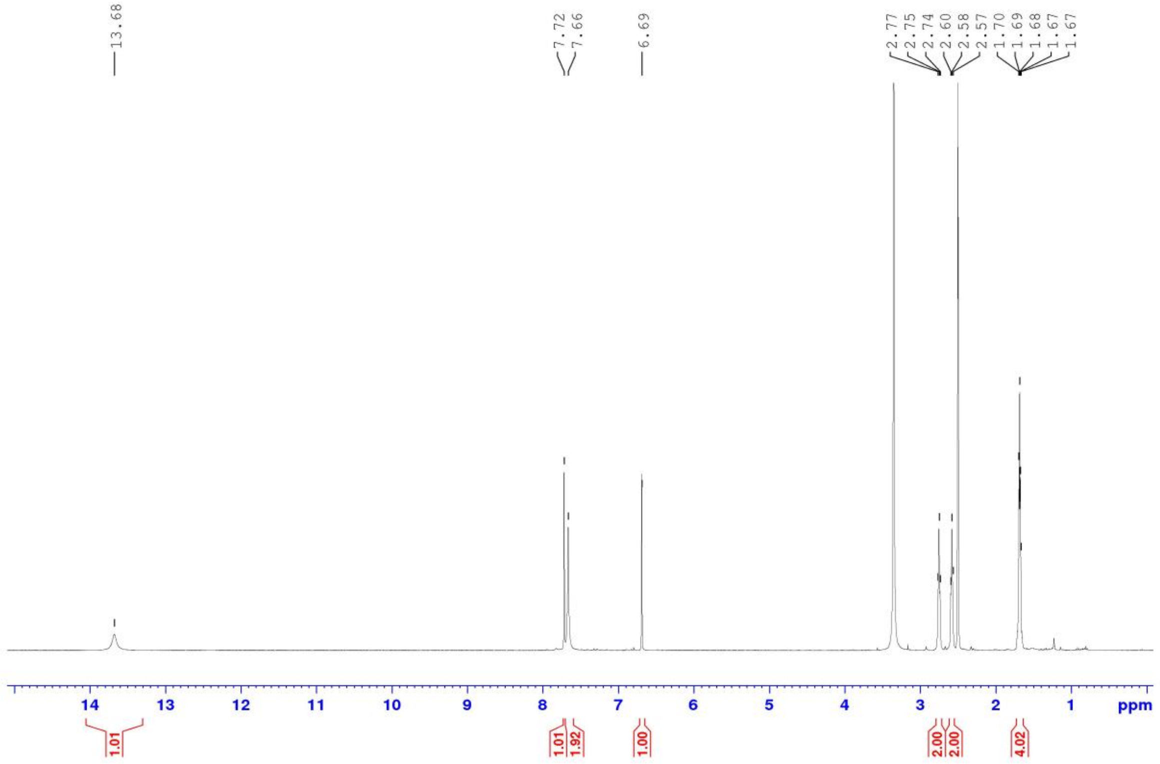

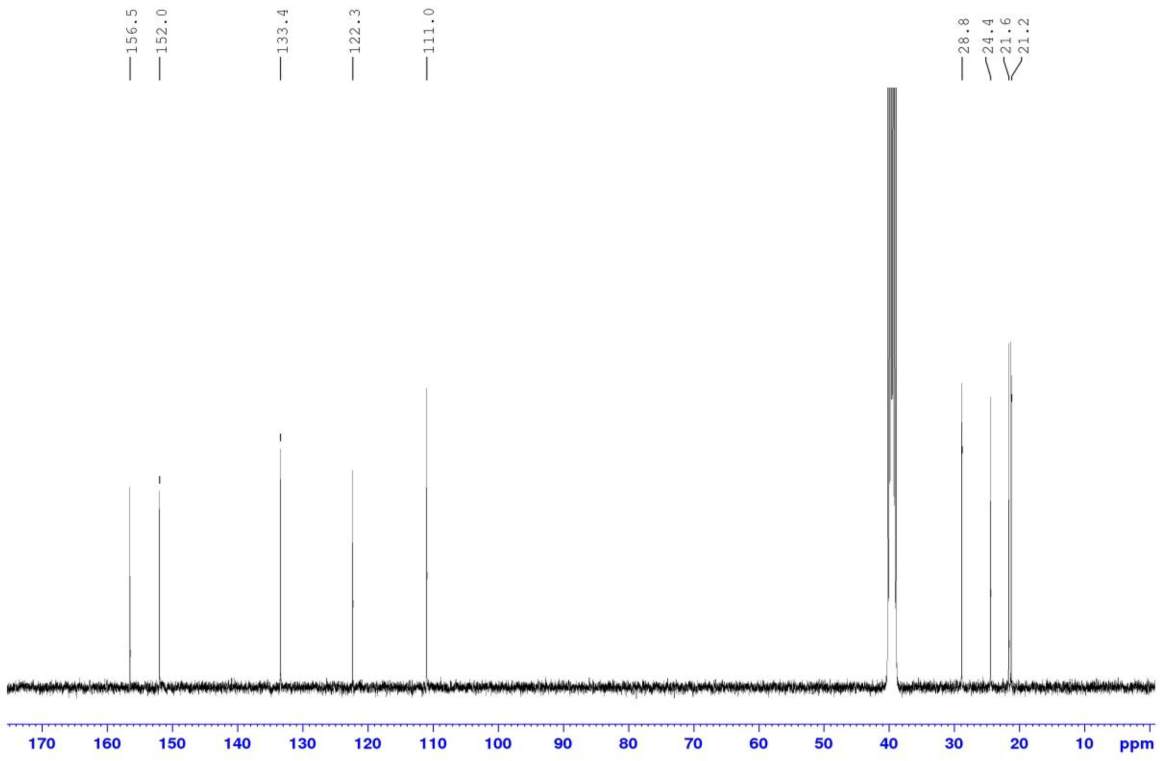

7-Methyl-5,6,7,8-tetrahydroisoquinolin-3-amine hydrochloride, **37b**

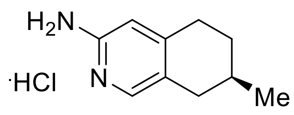

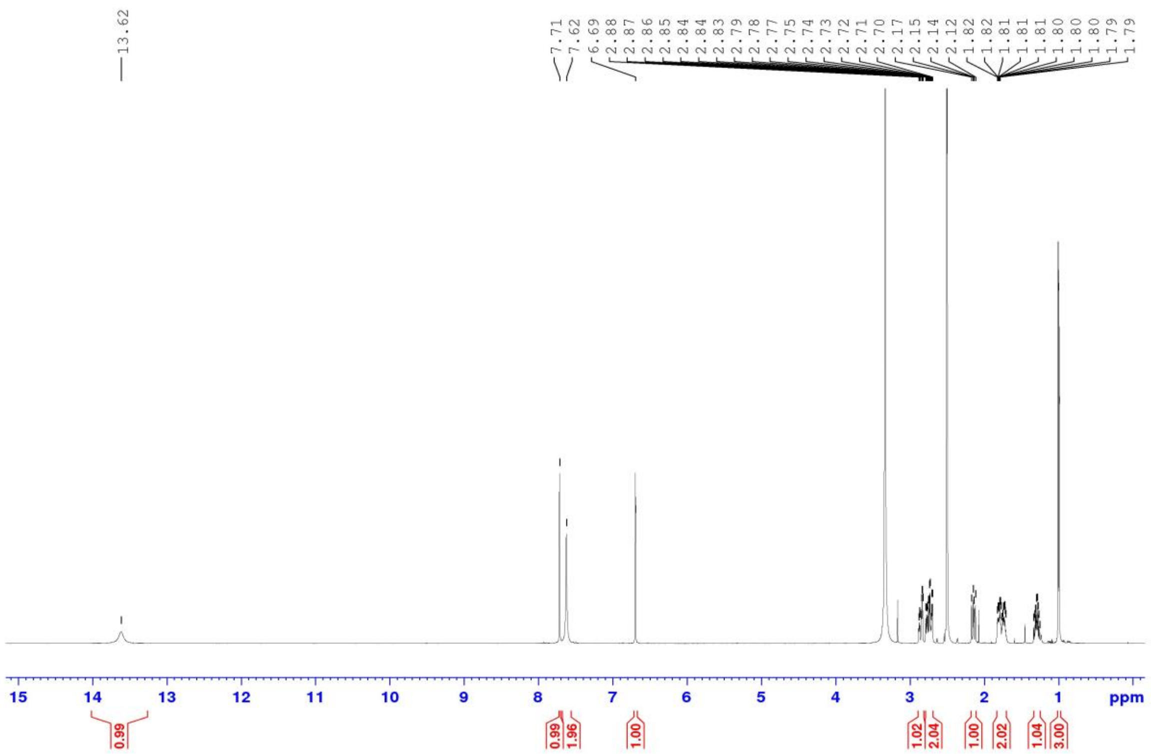

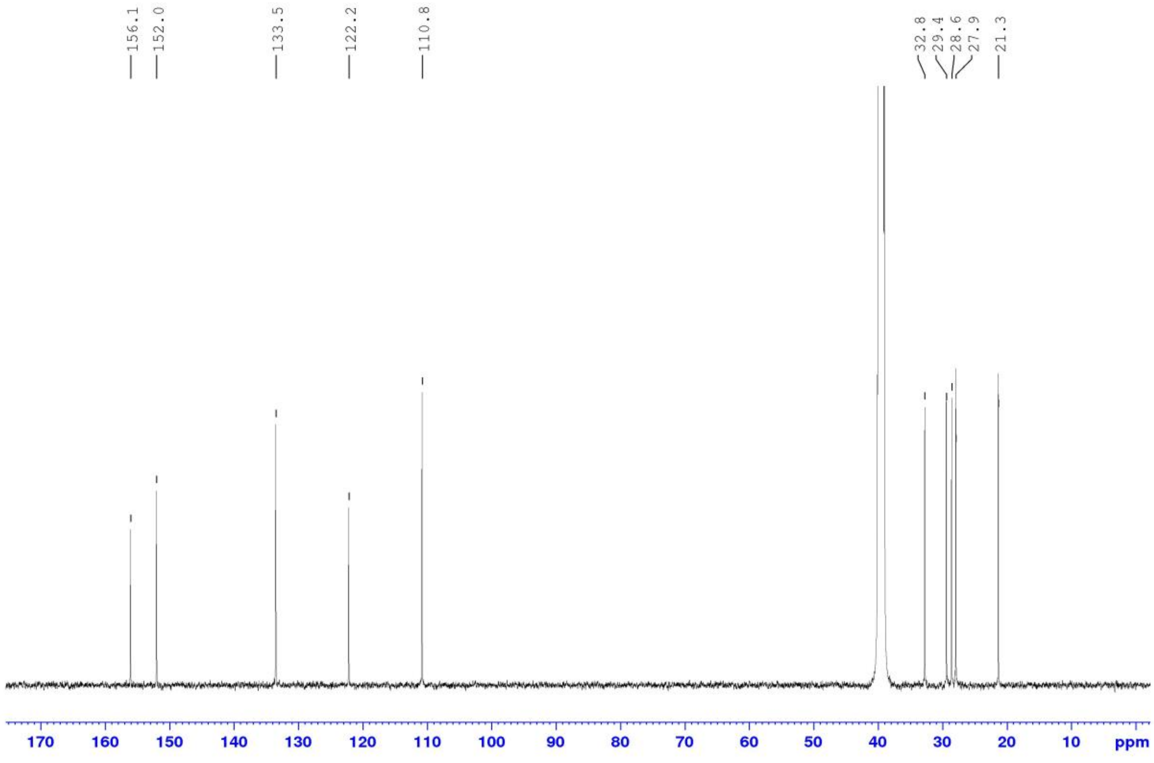

7-Trifluoromethyl-5,6,7,8-tetrahydroisoquinolin-3-amine hydrochloride, **37c**

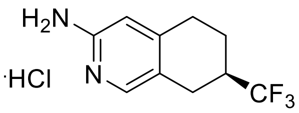

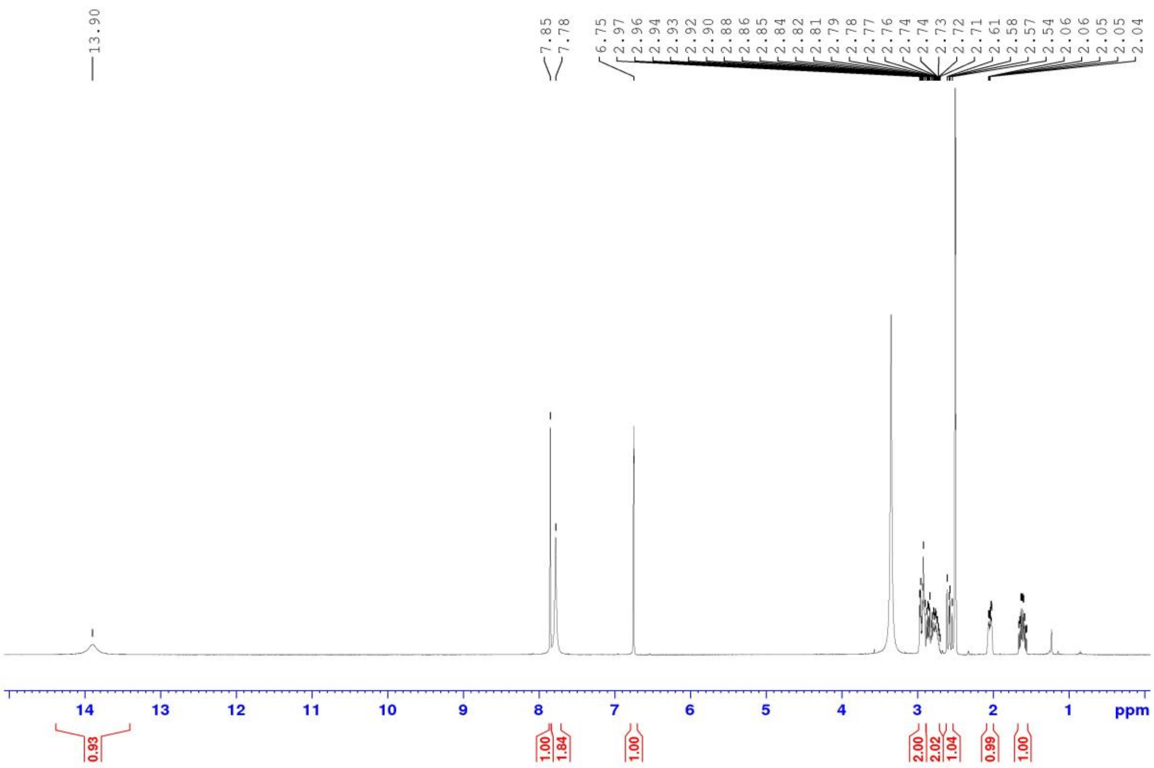

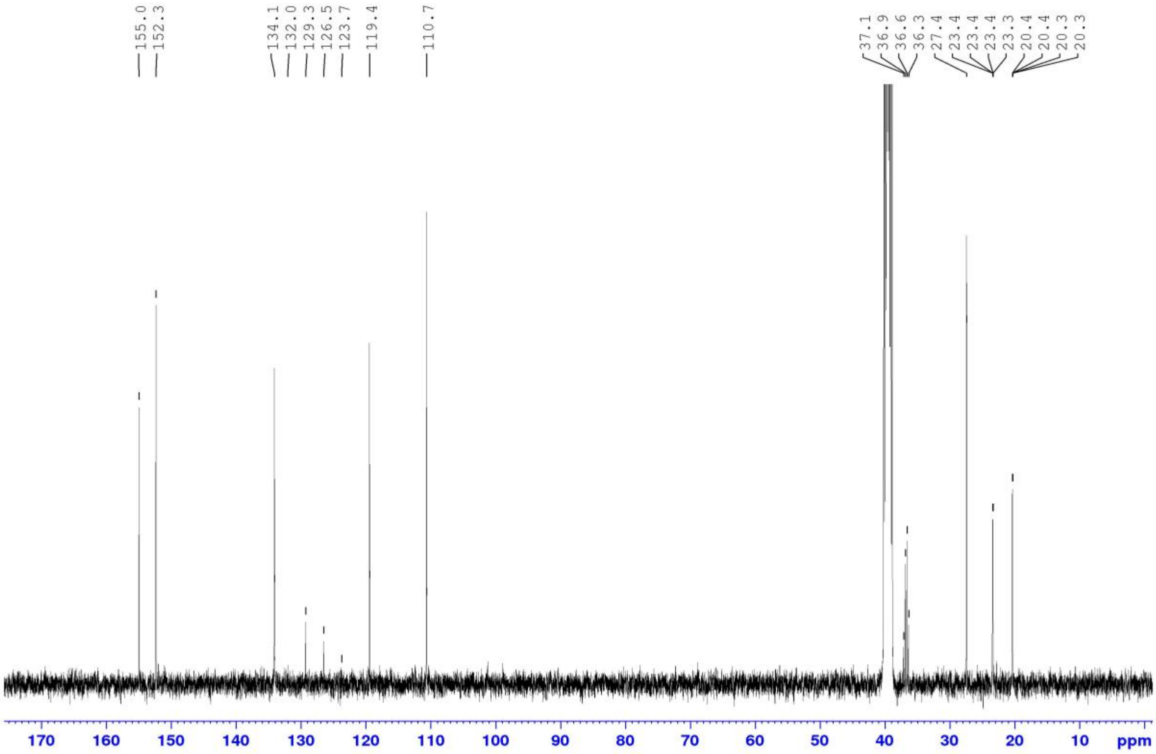

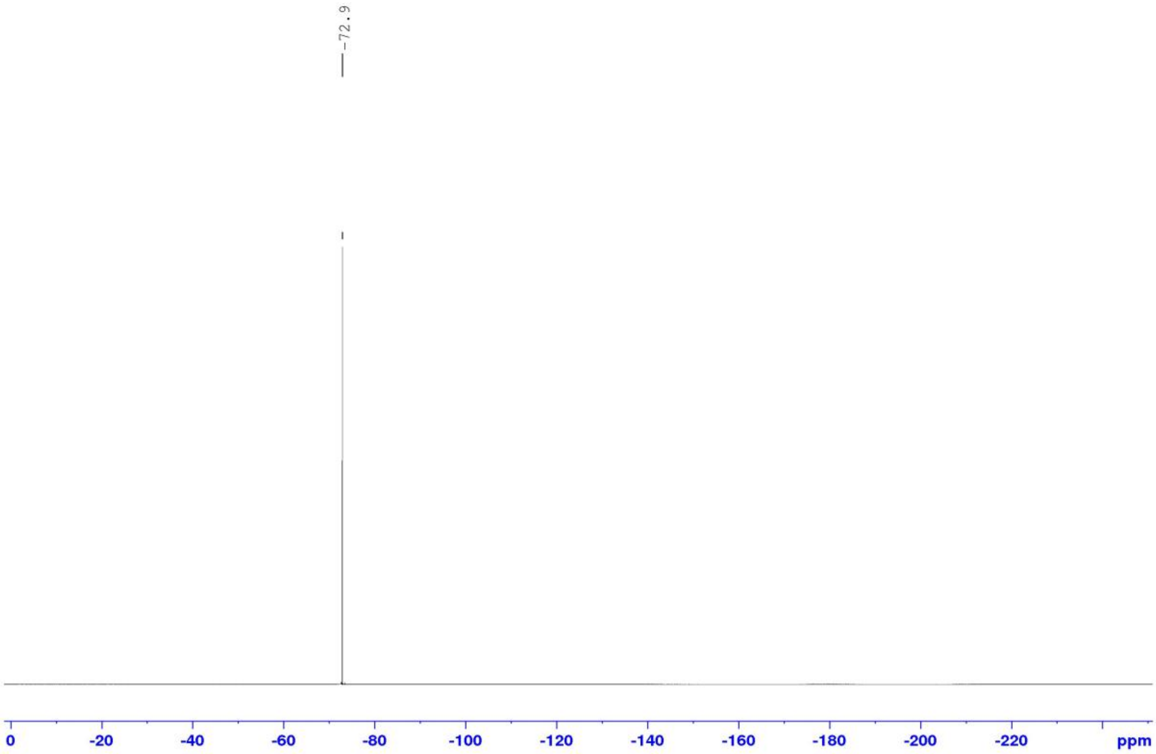

-72.9
0
-20
-40
-60
-80
-100
-120
-140
-160
-180
-200
-220
ppm

8-Methyl-5,6,7,8-tetrahydroisoquinolin-3-amine hydrochloride, **37d**

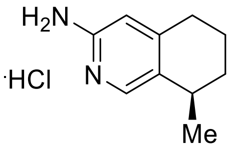

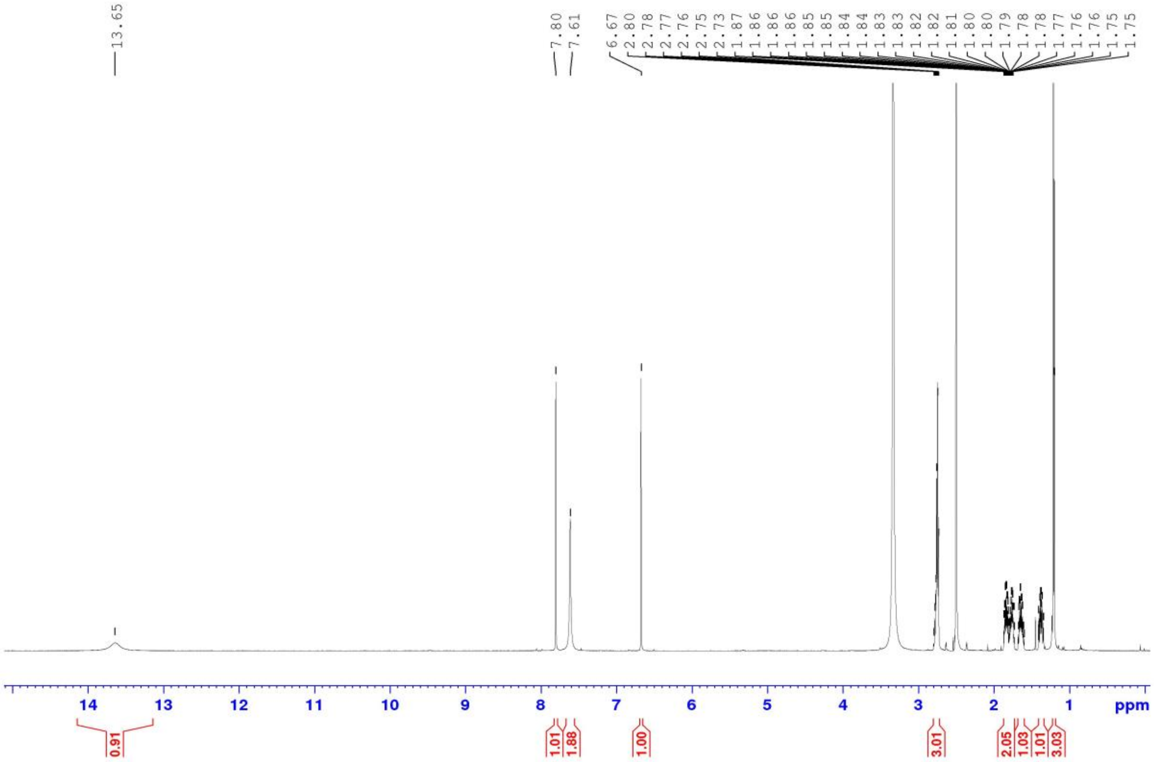

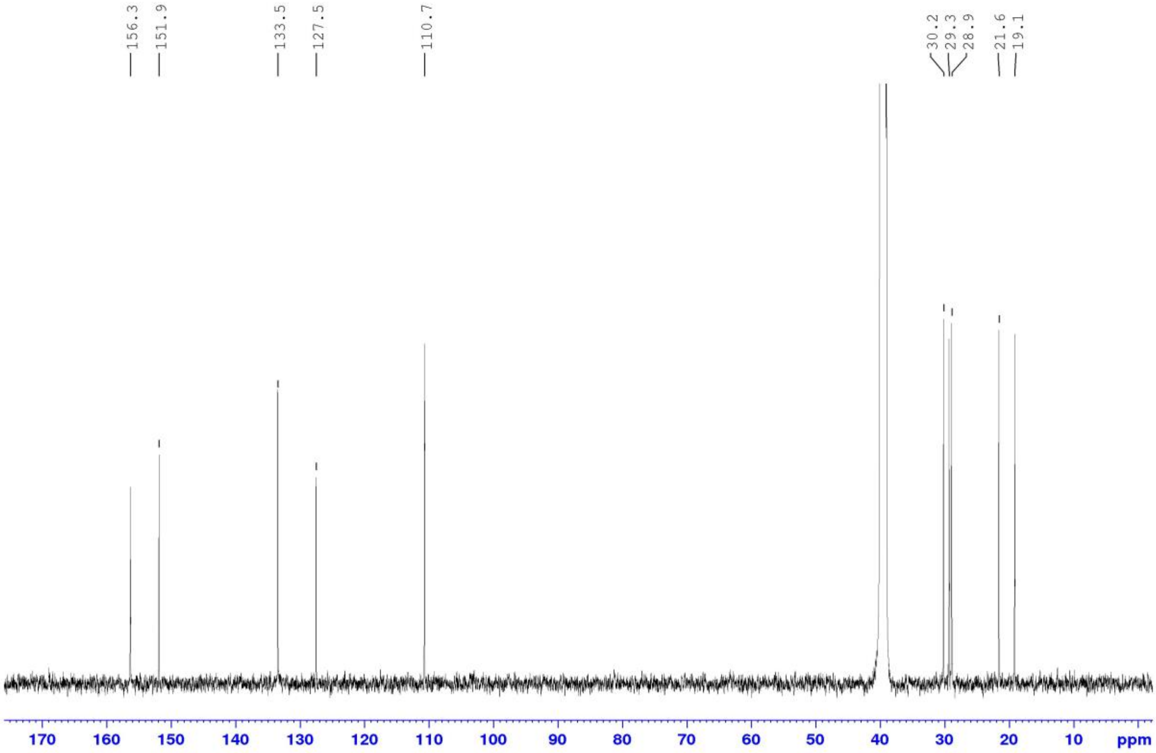

8-Phenyl-5,6,7,8-tetrahydroisoquinolin-3-amine hydrochloride, **37e**

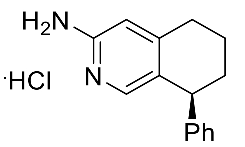

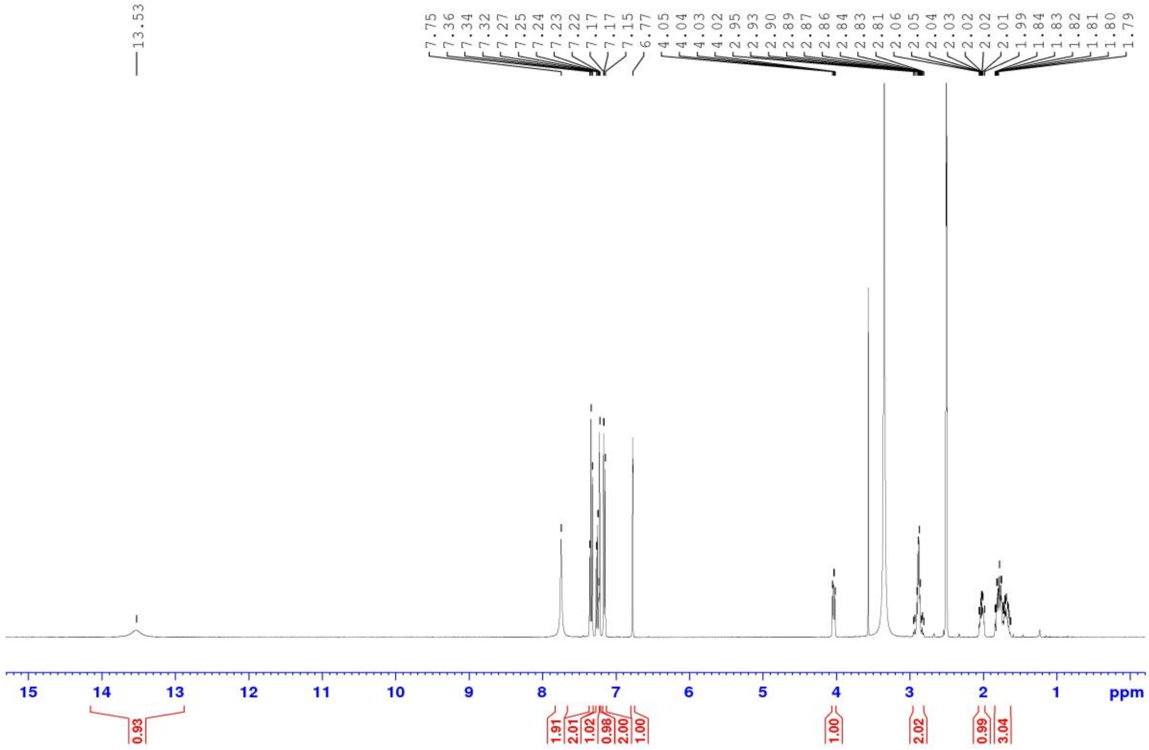

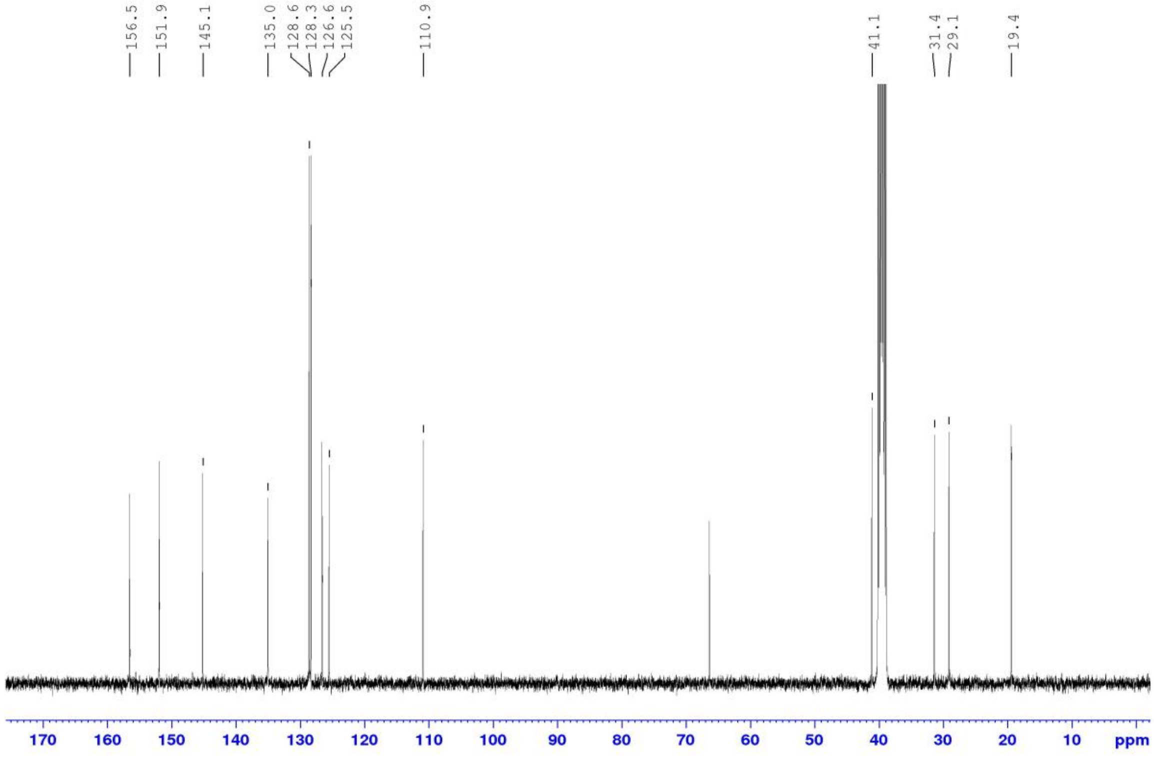

6,7,8,9-Tetrahydro-5*H*-cyclohepta[*c*]pyridin-3-amine hydrochloride, **37f**

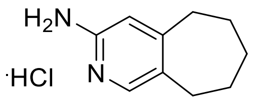

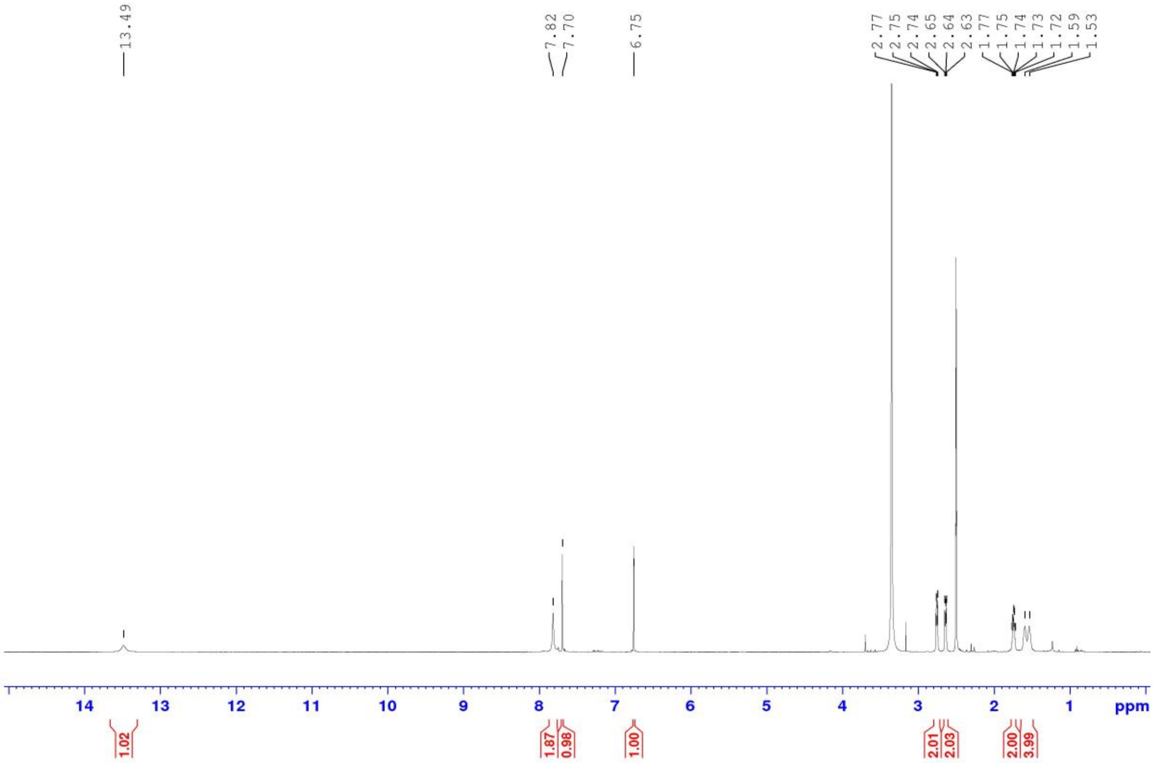

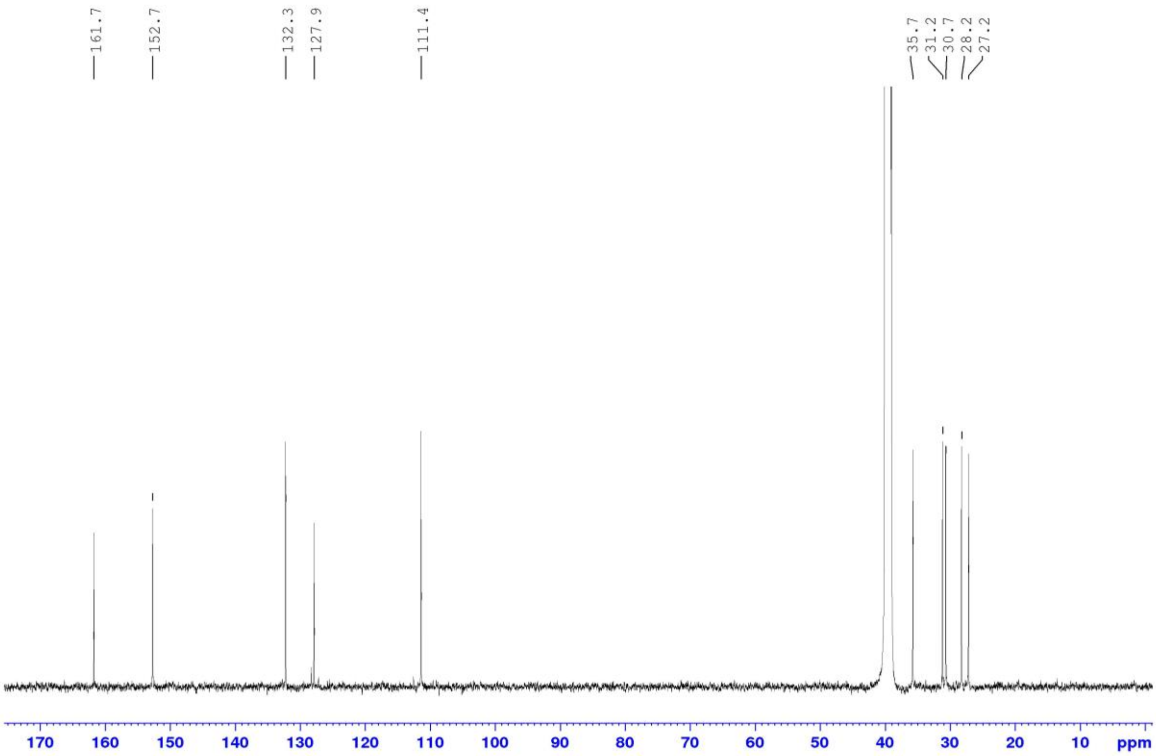

N,N-(bis-Boc)-1-tosyl-1a,2,3,7b-tetrahydro-1H-azirino[2,3-h]isoquinolin-5-amine, **38**

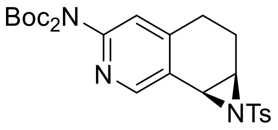

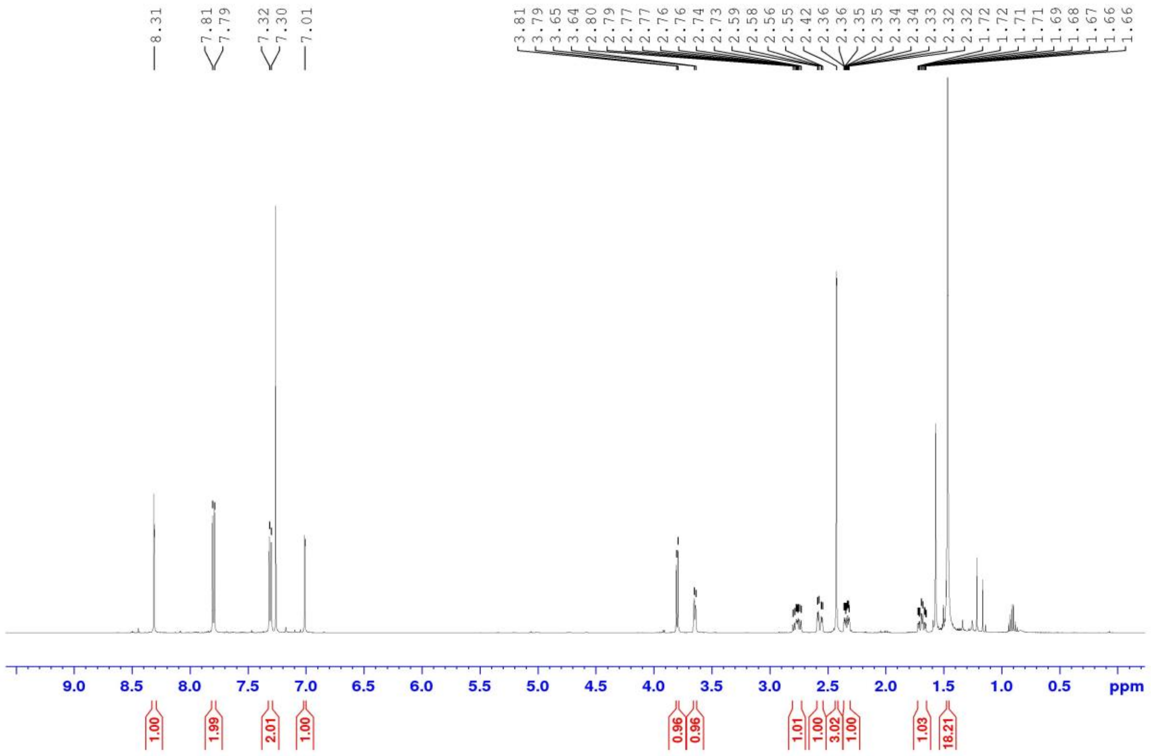

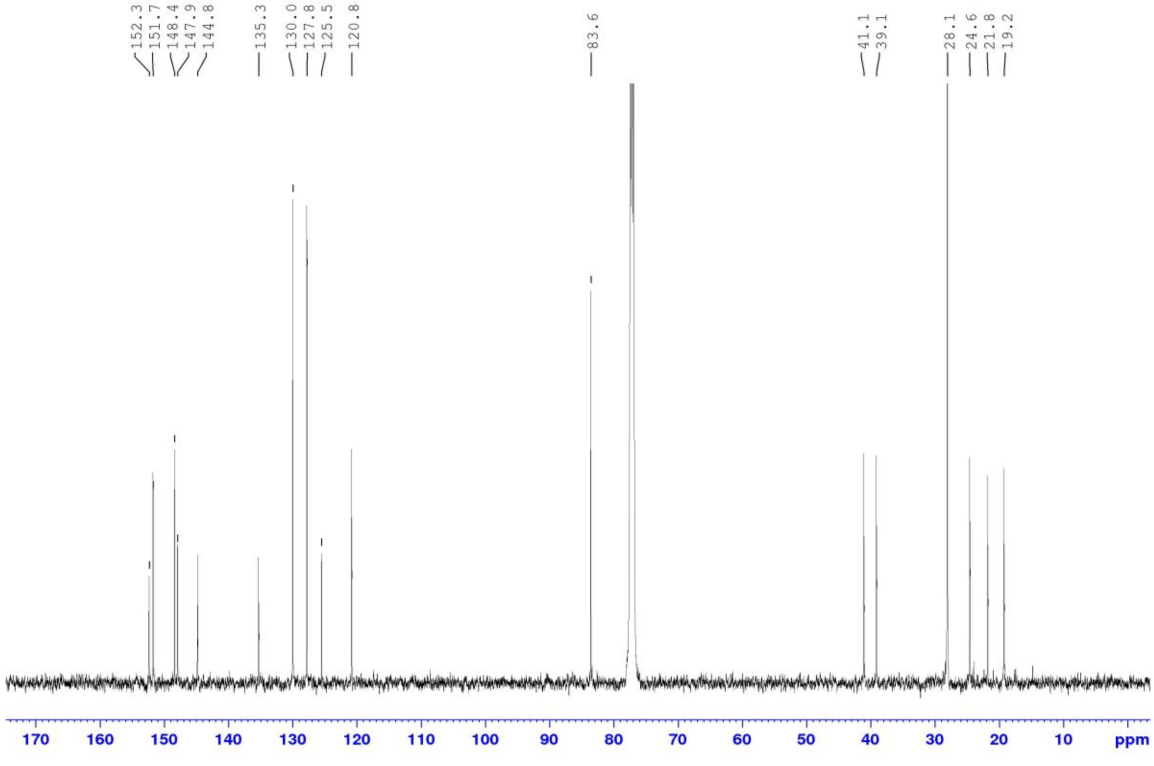

trans-7,8-dibromo-*N,N*-(bis-Boc)-5,6,7,8-tetrahydroisoquinolin-3-amine, **39**

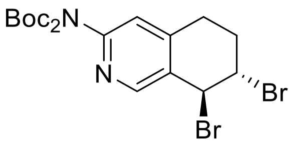

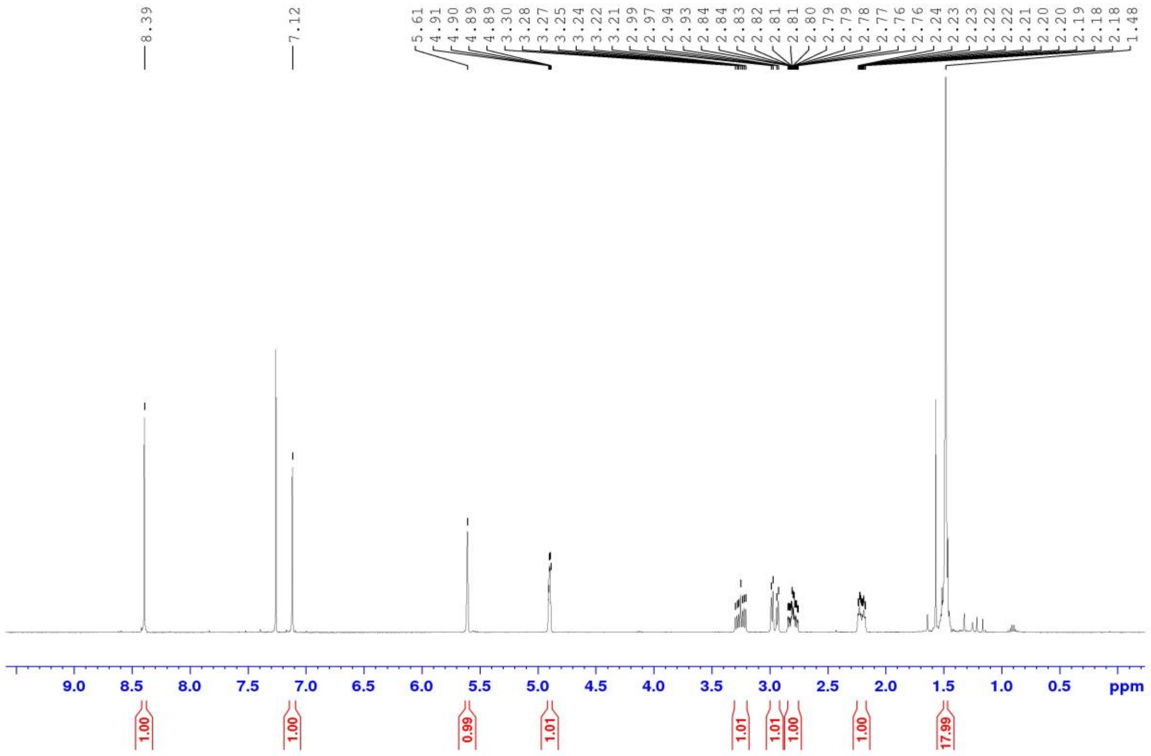

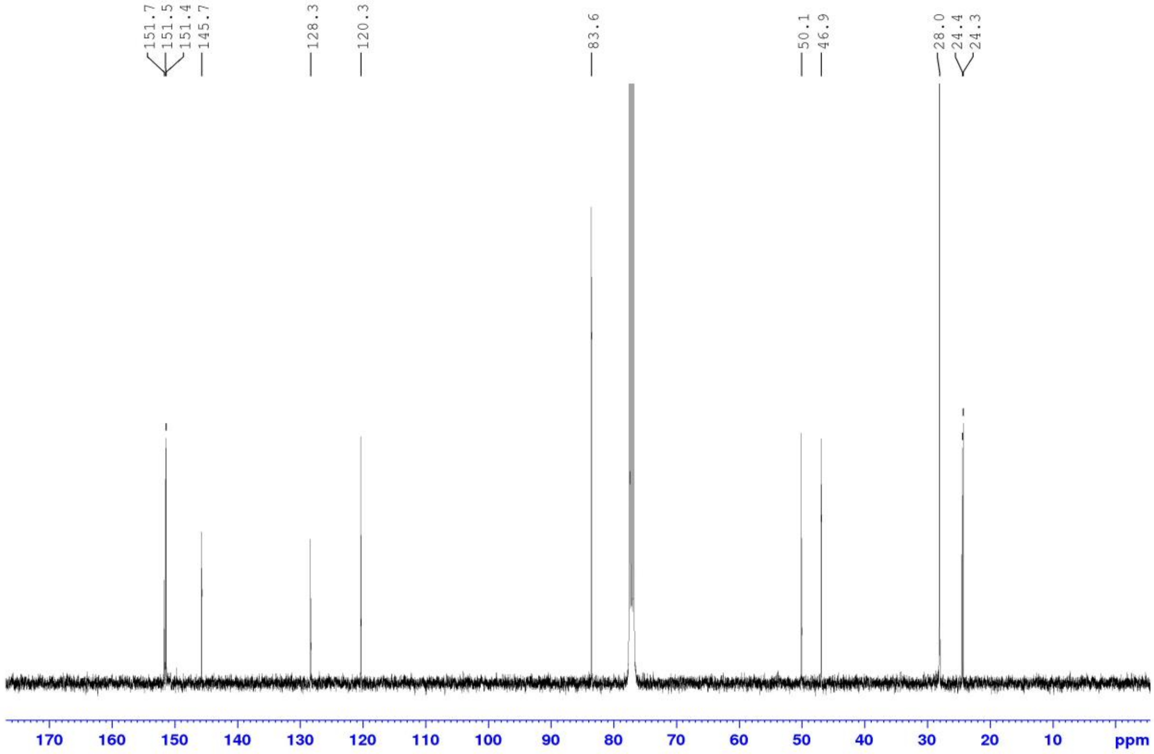

cis-3-(*N,N*-bis-Boc-amino)-5,6,7,8-tetrahydroisoquinoline-7,8-diol, **40**

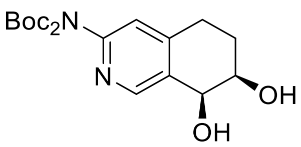

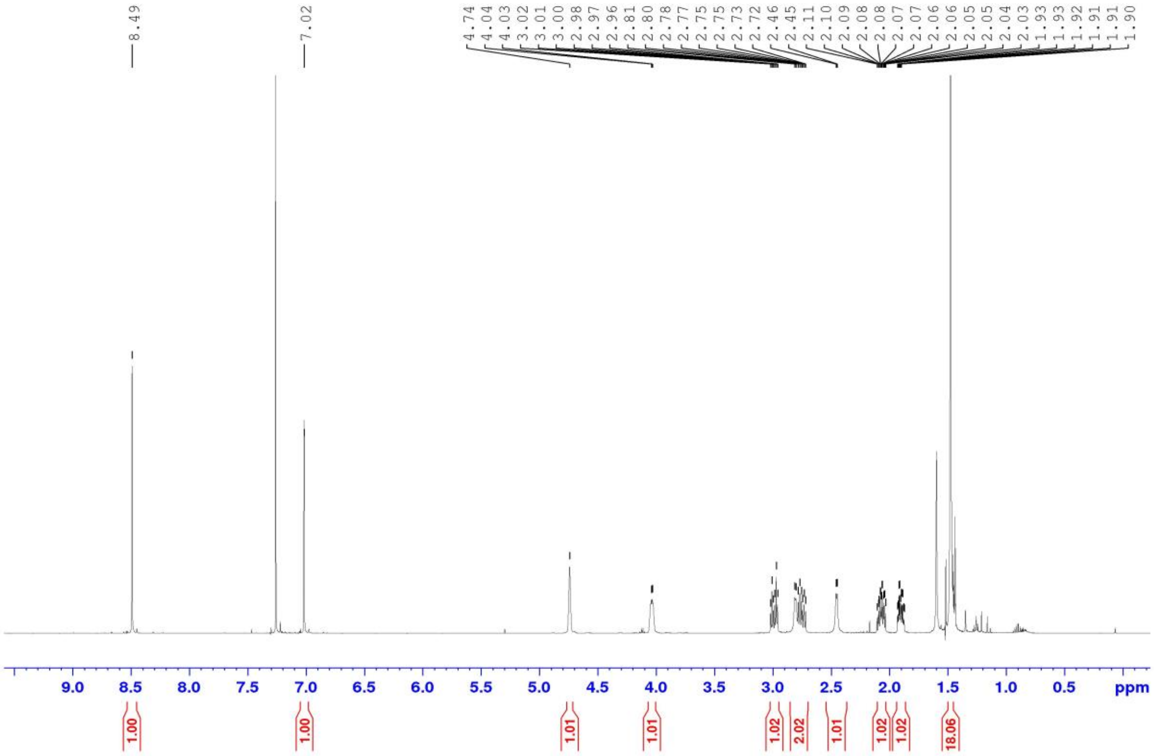

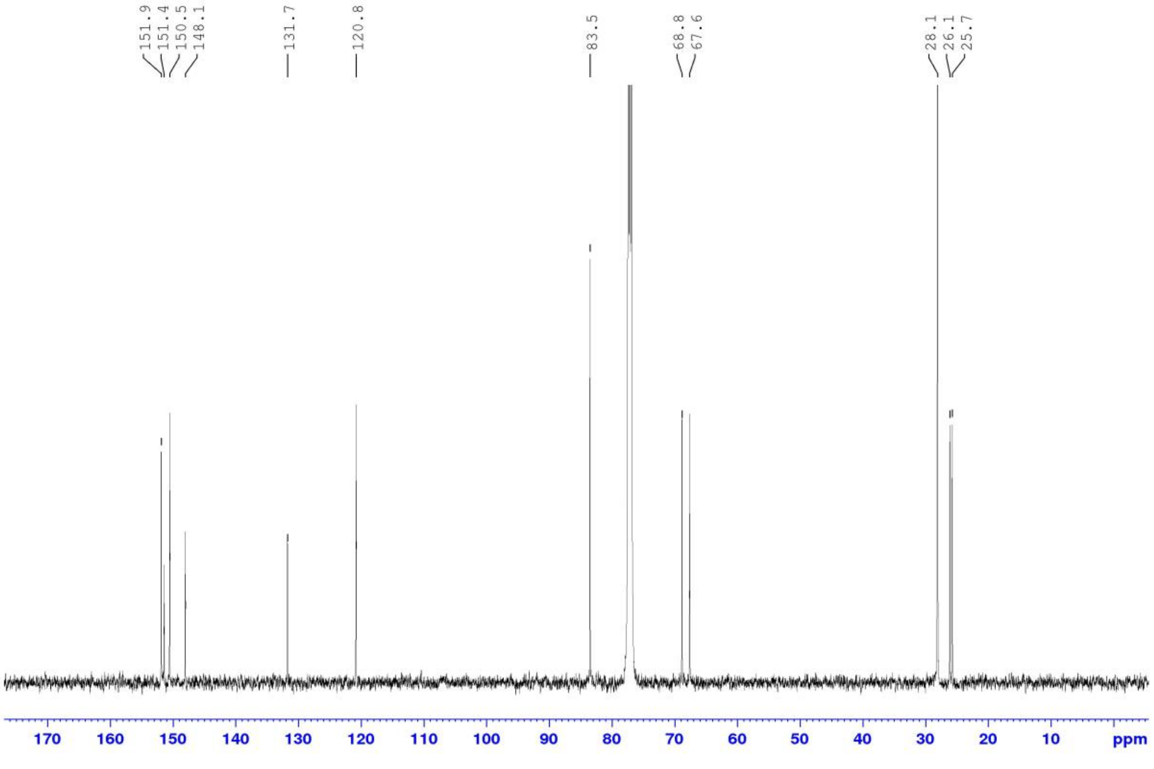

3-(*N,N*-bis-Boc-amino)-5,6,7,8-tetrahydroisoquinolin-8-ol, **41**

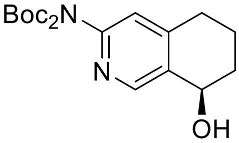

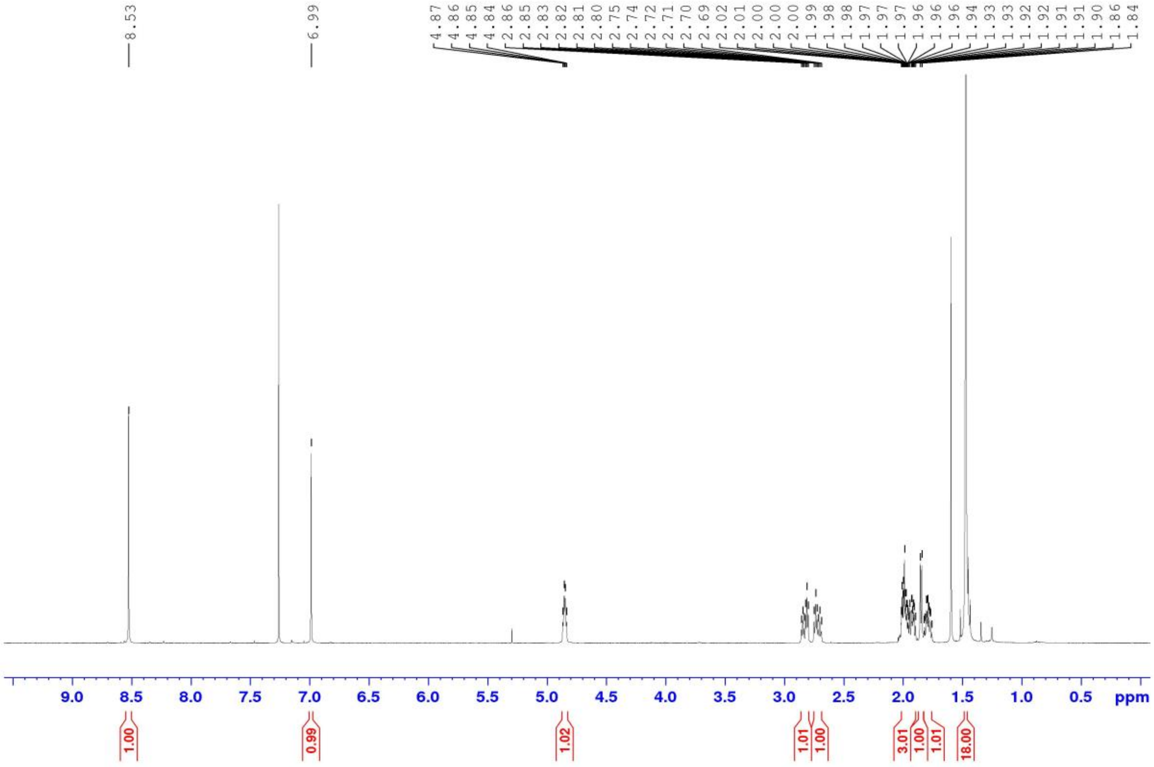

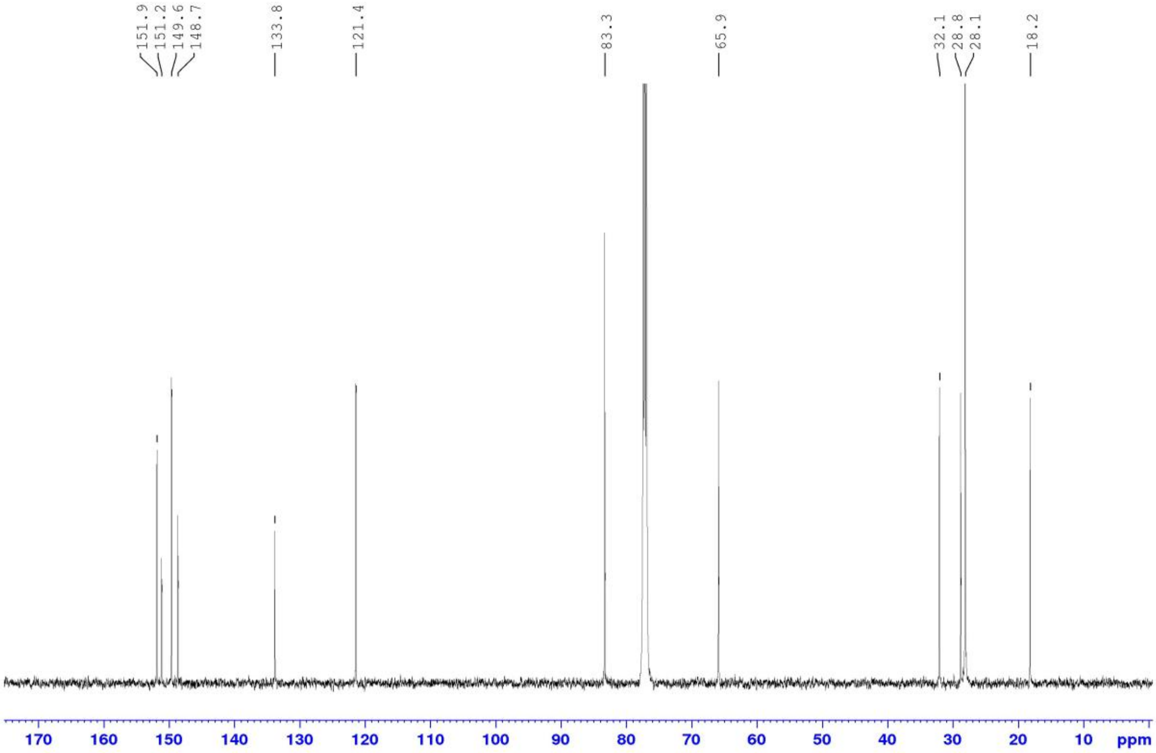

7-bromo-3-(*N,N*-bis-Boc-amino)-6,7-dihydroisoquinolin-8(5H)-one, **42**

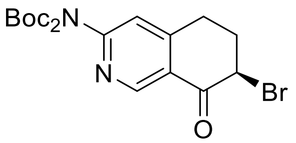

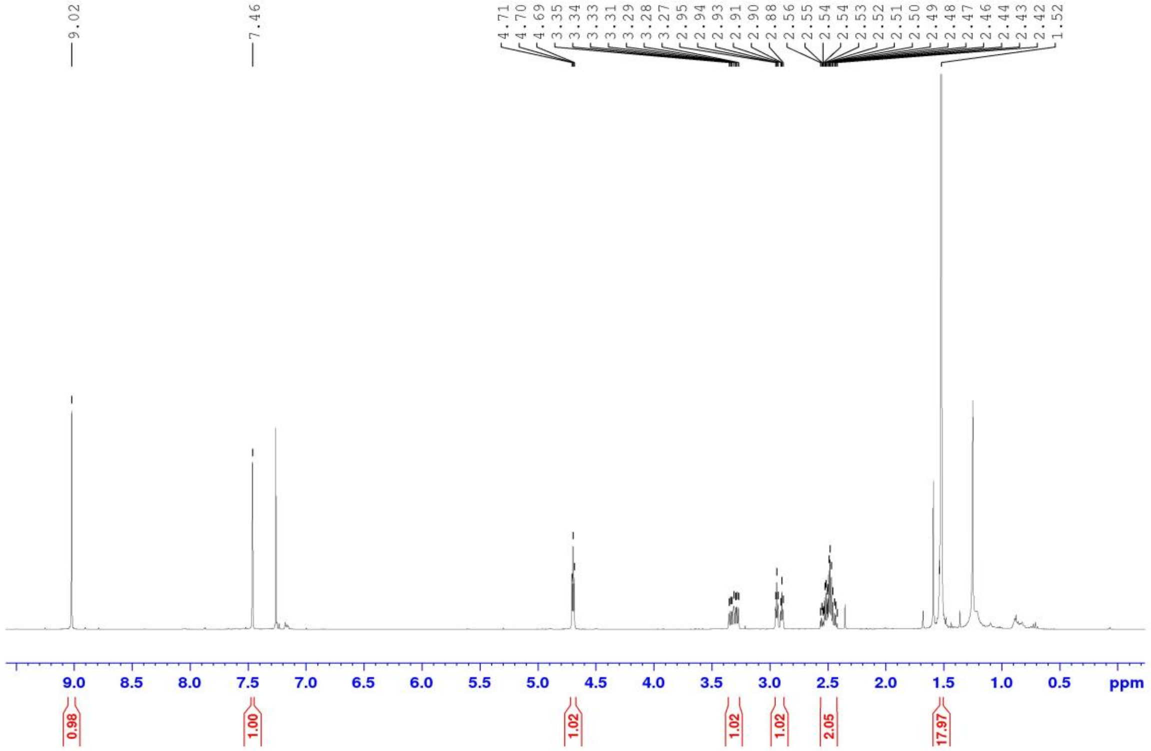

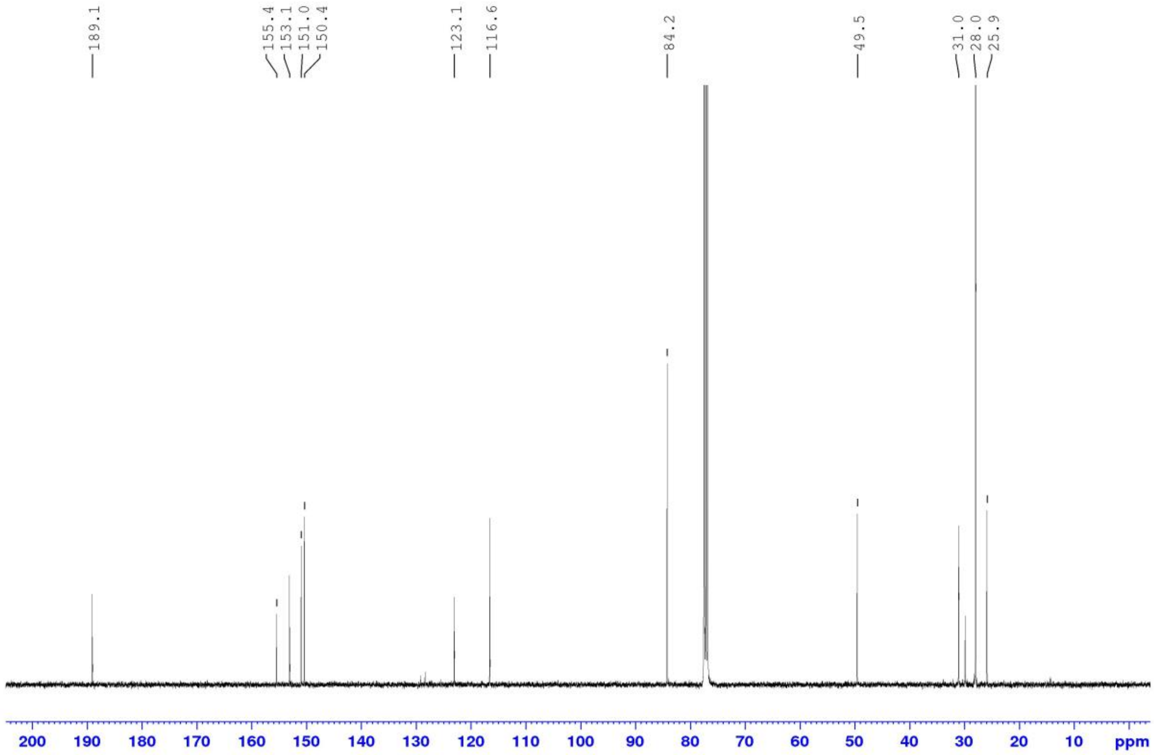

trans-7-Bromo-3-(*N,N*-bis-Boc-amino)-5,6,7,8-tetrahydroisoquinolin-8-ol, **43**

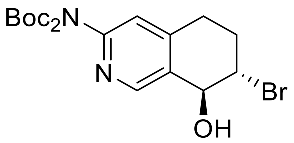

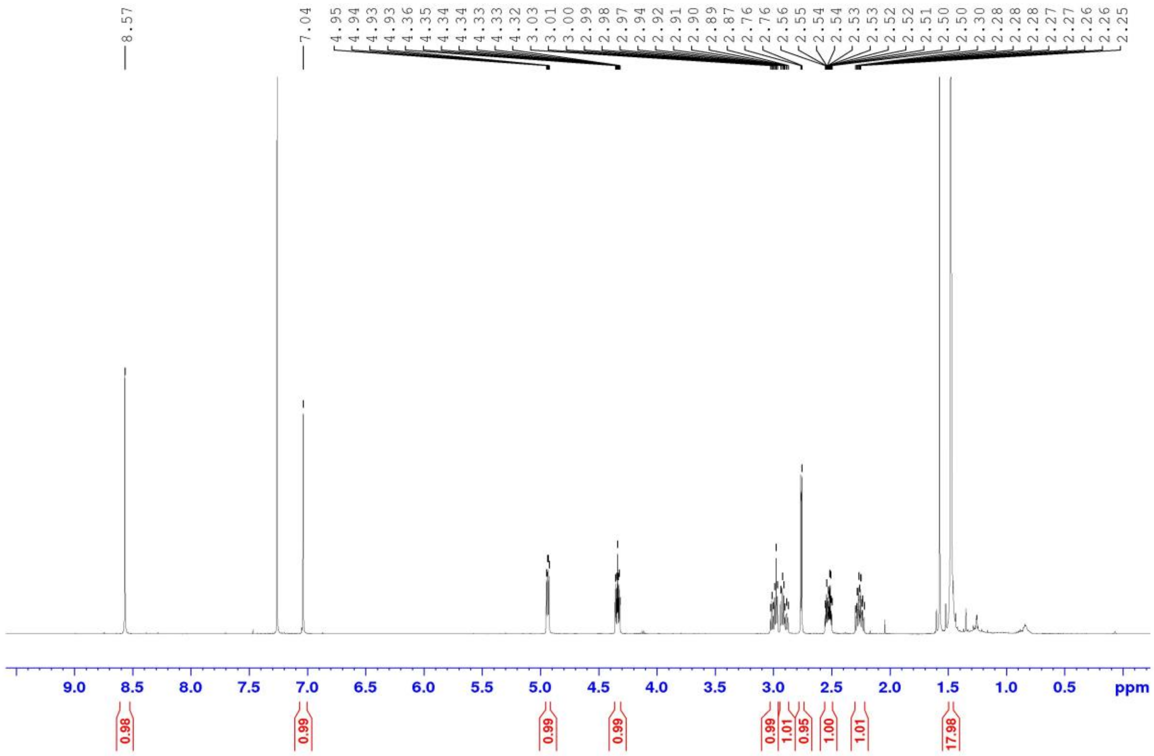

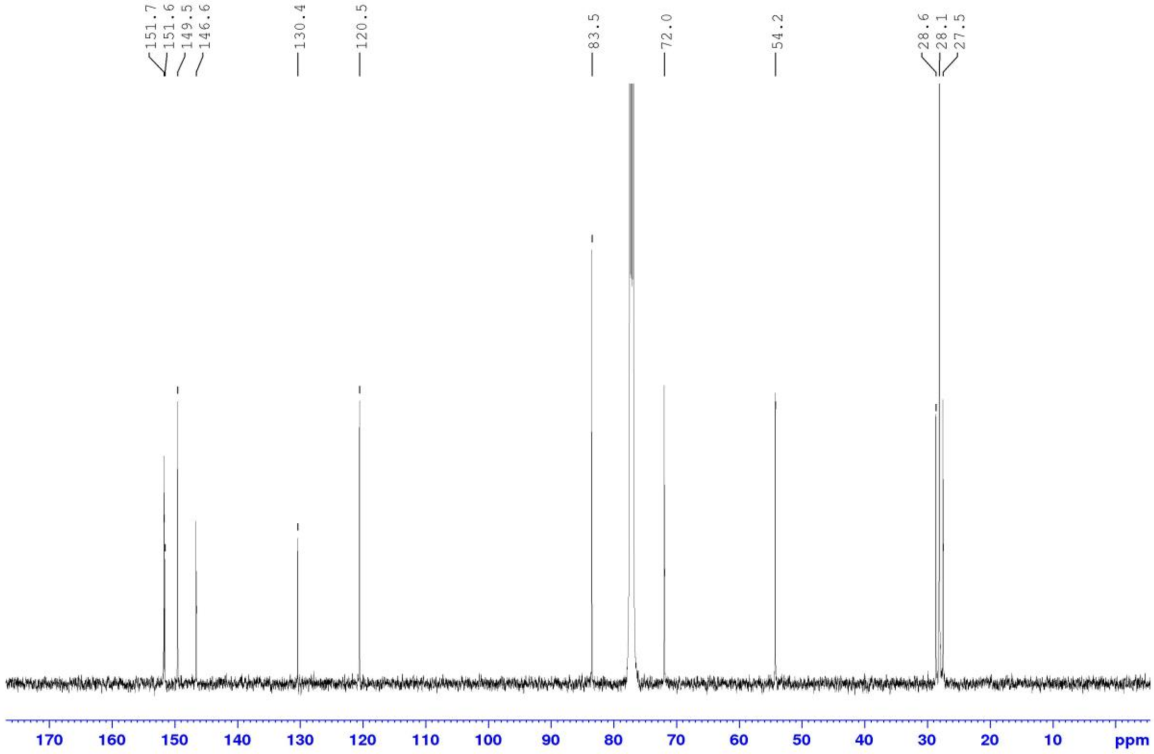

tert-Butyl (8-Amino-7-hydroxy-5,6,7,8-tetrahydroisoquinolin-3-yl) carbamate, **44**

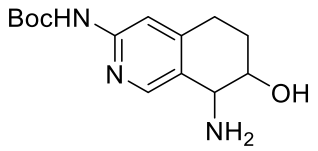

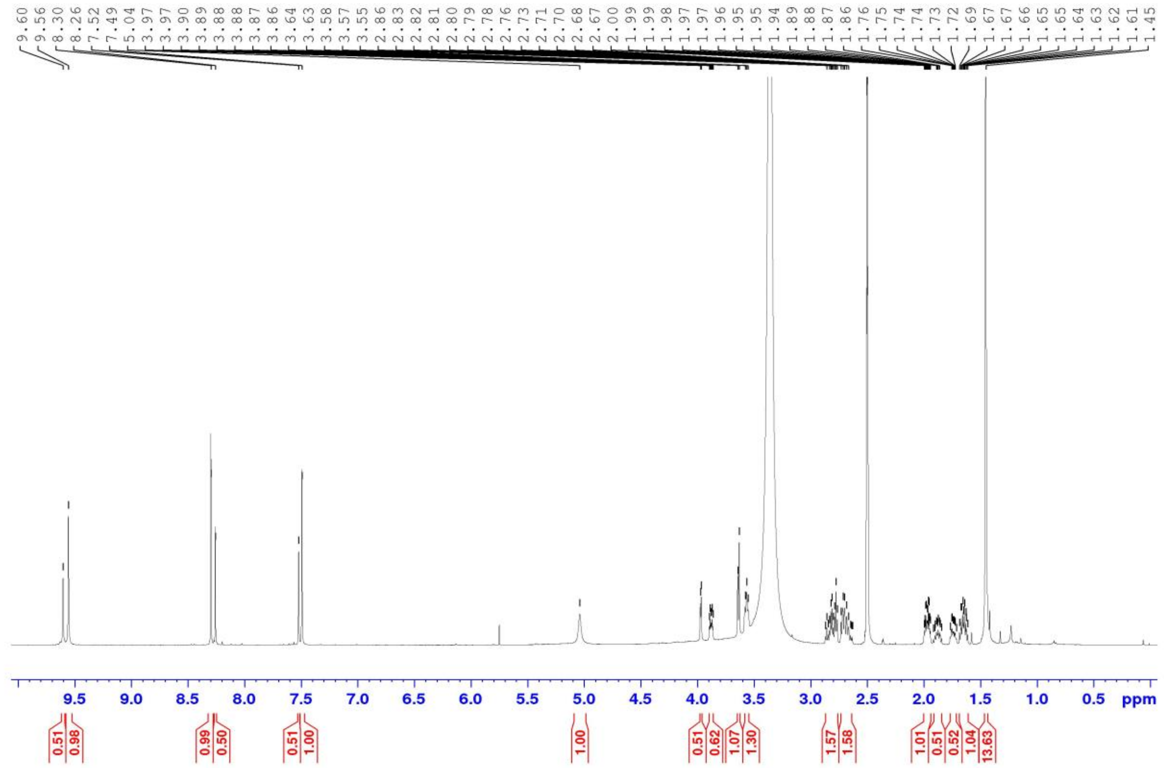

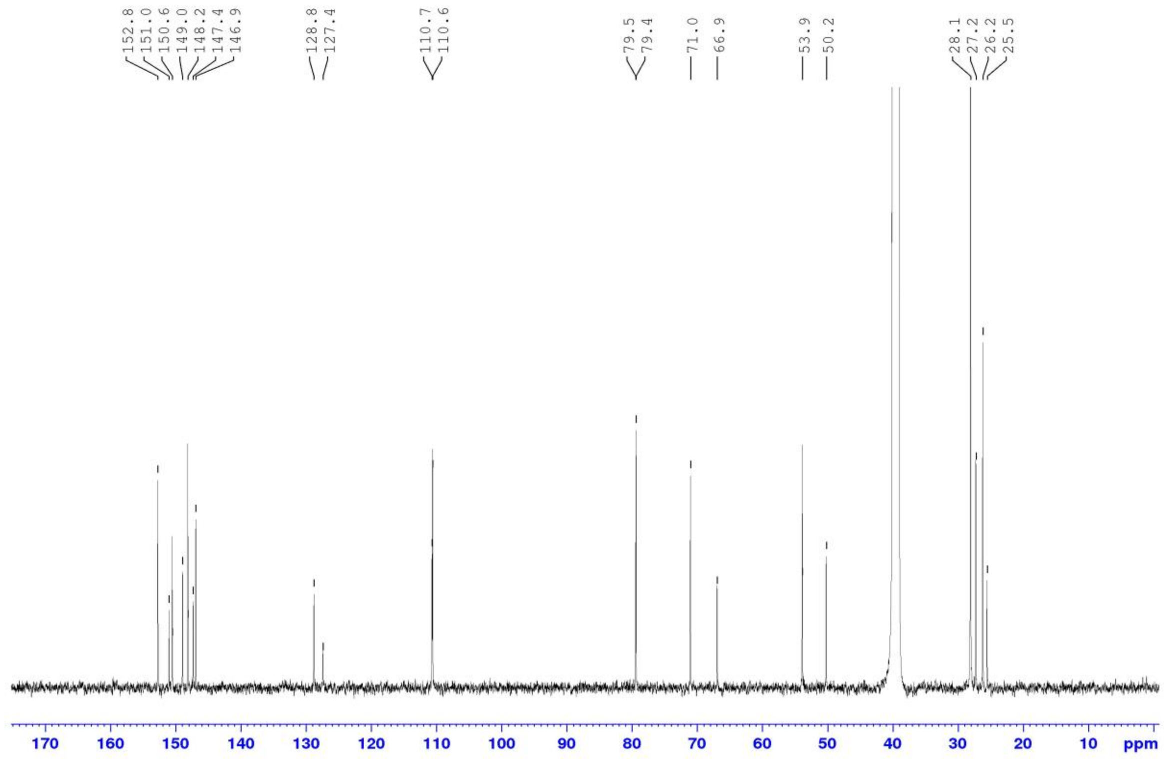

N,N-(bis-Boc)-7,8-epoxy-5,6,7,8-tetrahydroisoquinolin-3-amine, **45**

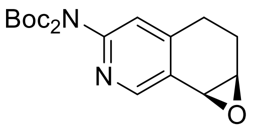

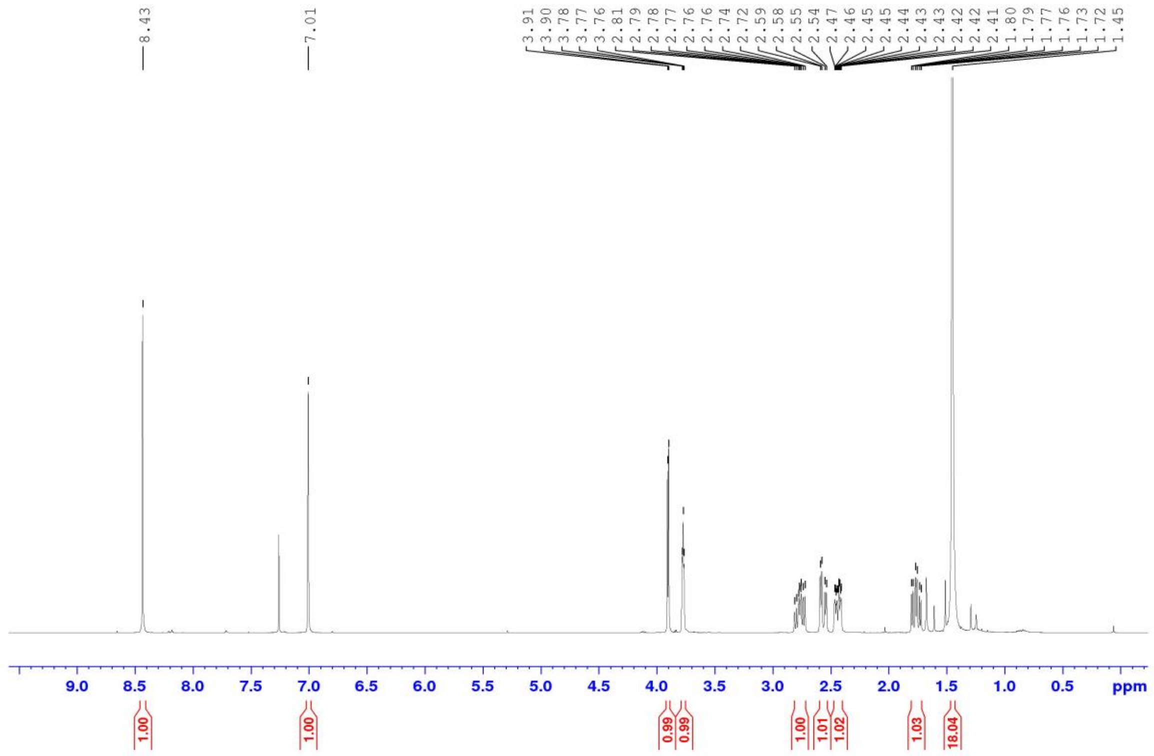

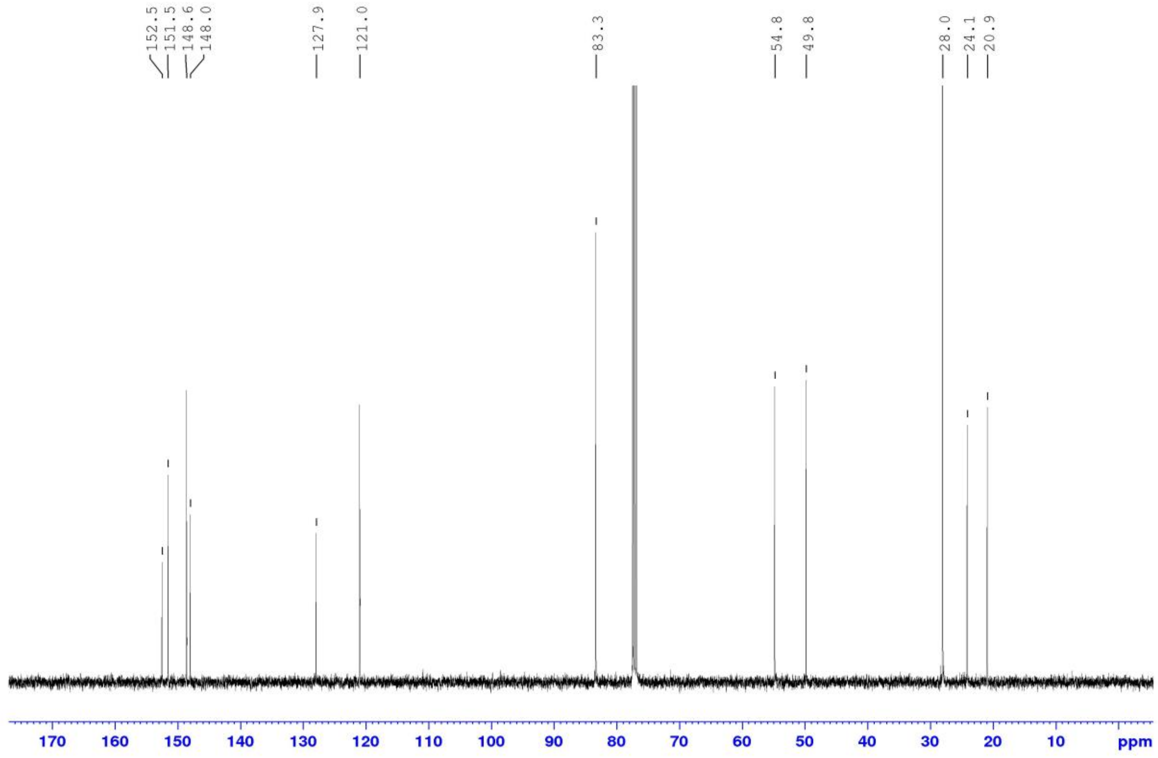

3-Chloro-6,7,8,9-tetrahydro-5*H*-cyclohepta[*c*]pyridine, **46**

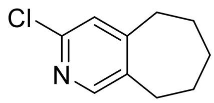

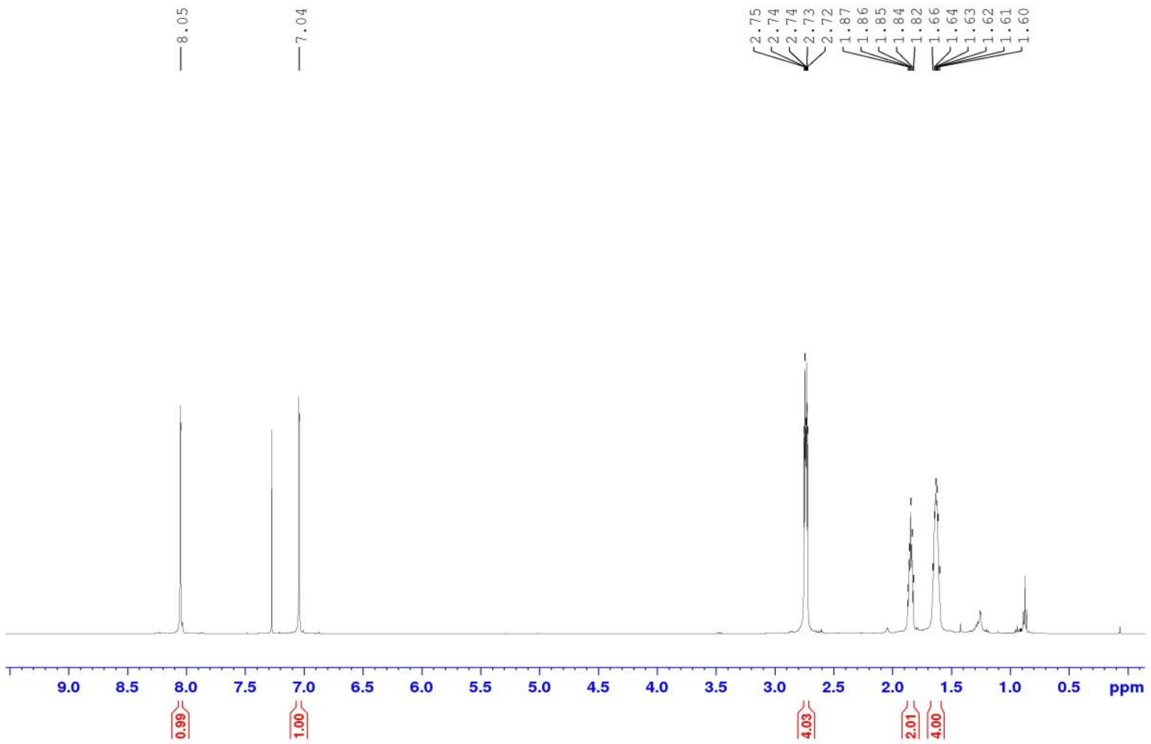

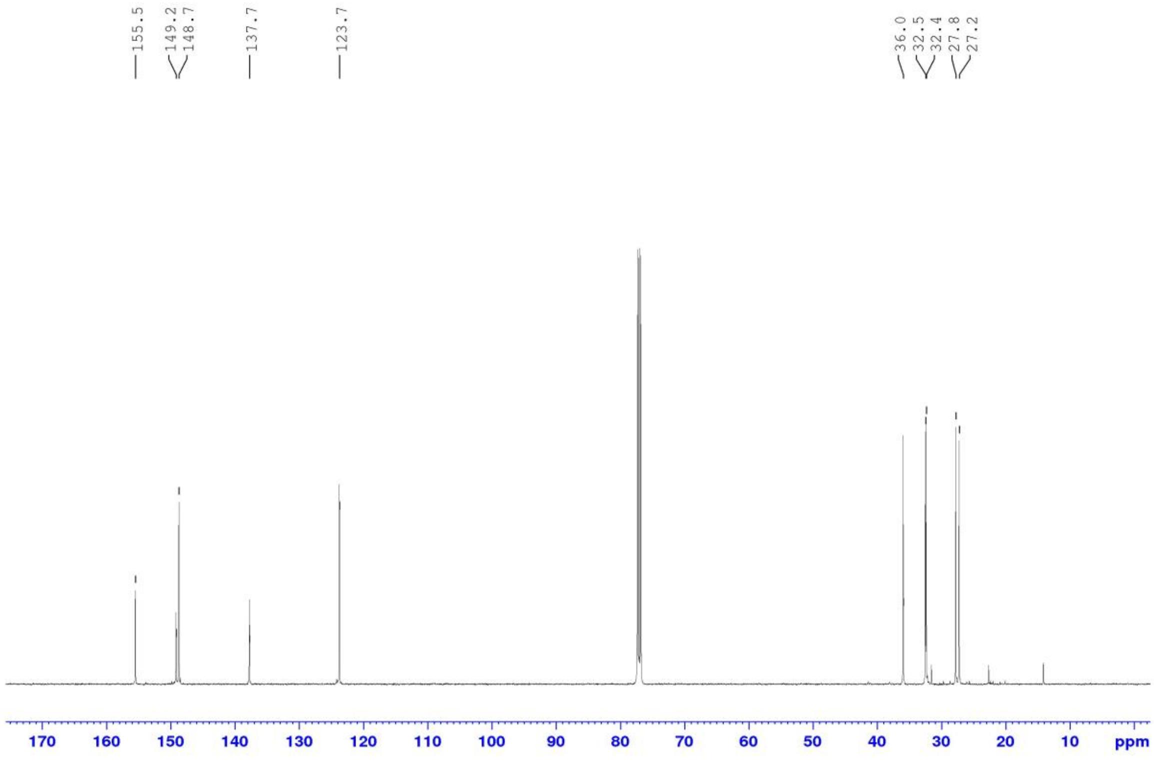

trans-7,8-Dibromo-3-chloro-6,7,8,9-tetrahydro-5*H*-cyclohepta[*c*]pyridine, **47**

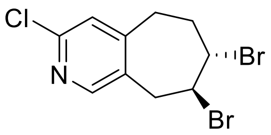

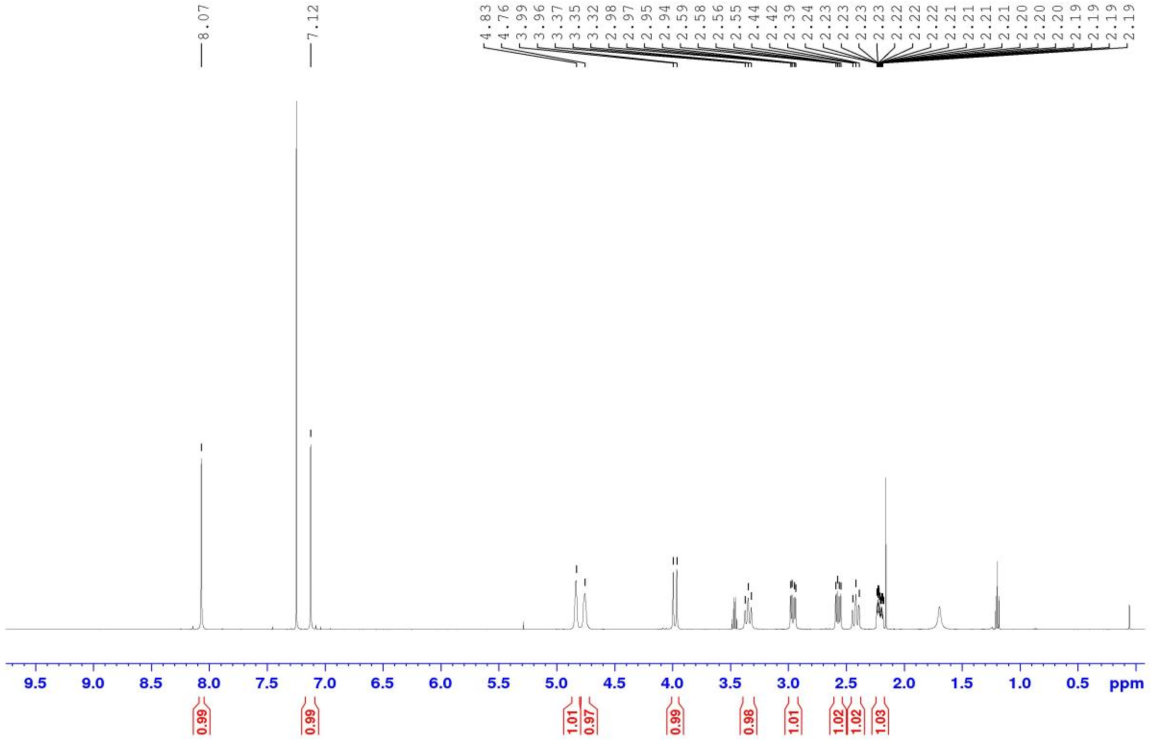

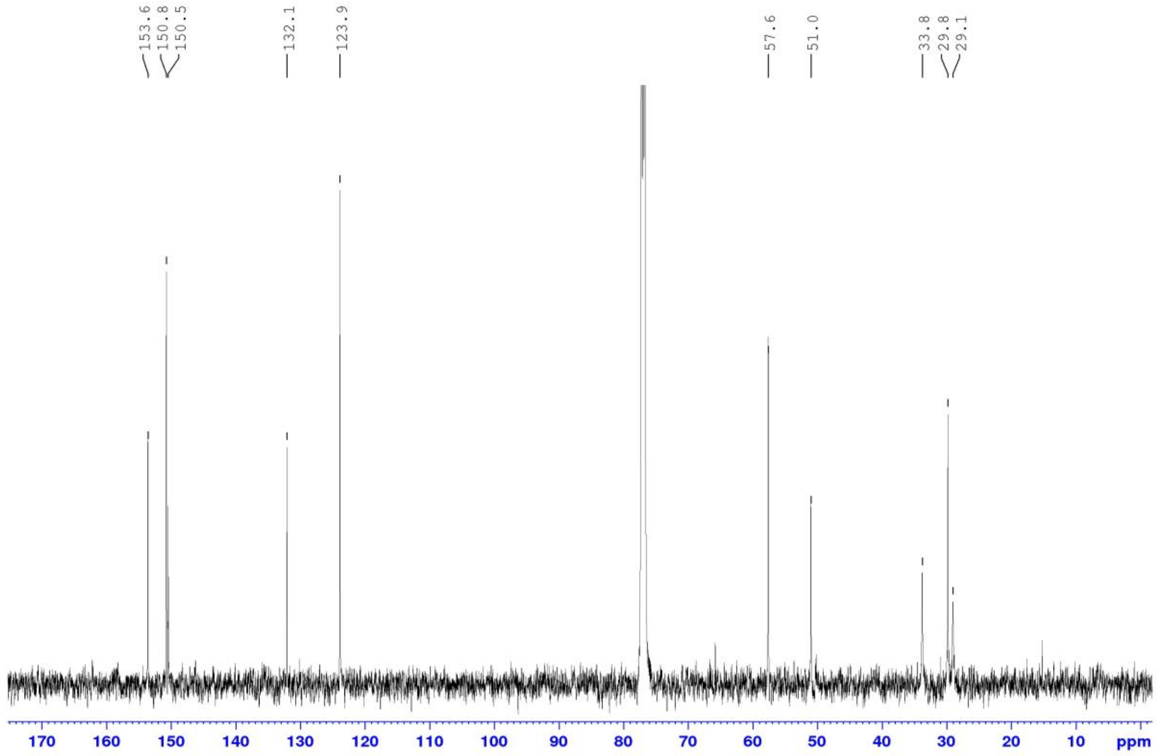

3-Chloro-6,7,8,9-tetrahydro-5*H*-cyclohepta[*c*]pyridin-7-ol, **48**

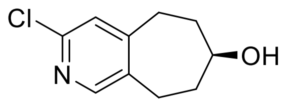

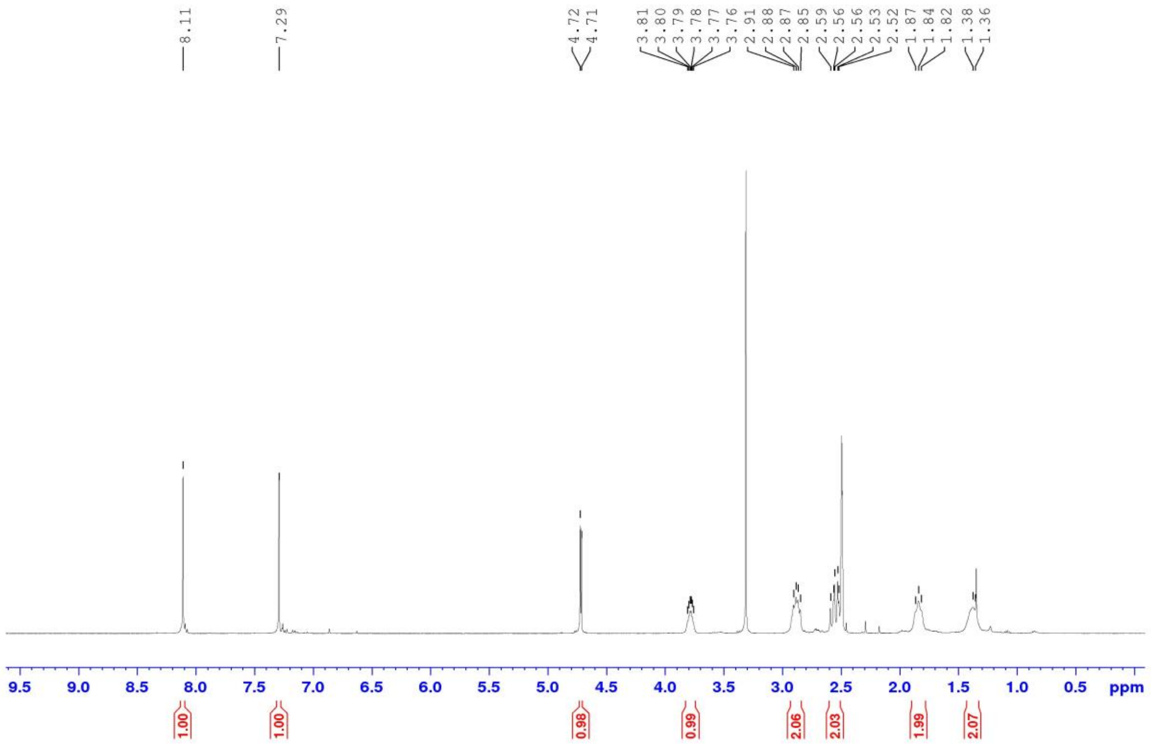

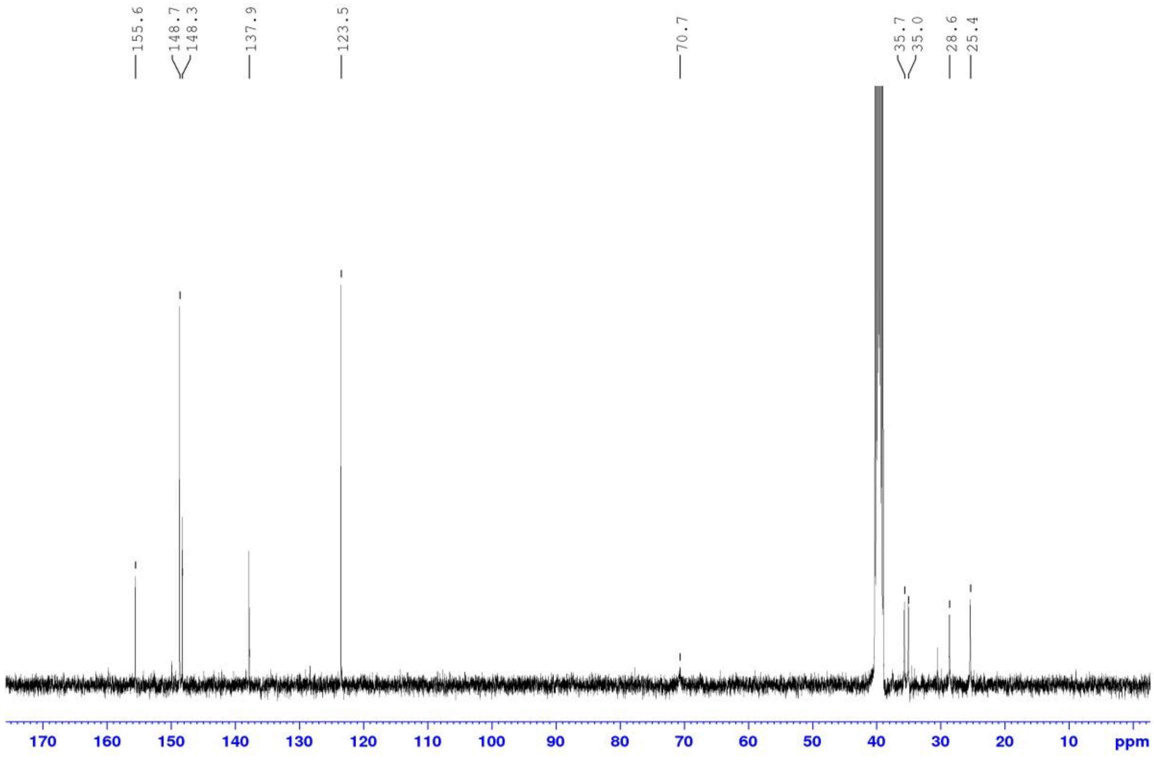

3-Chloro-6,7,8,9-tetrahydro-5*H*-cyclohepta[*c*]pyridin-8-ol, **49**

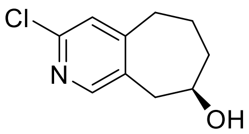

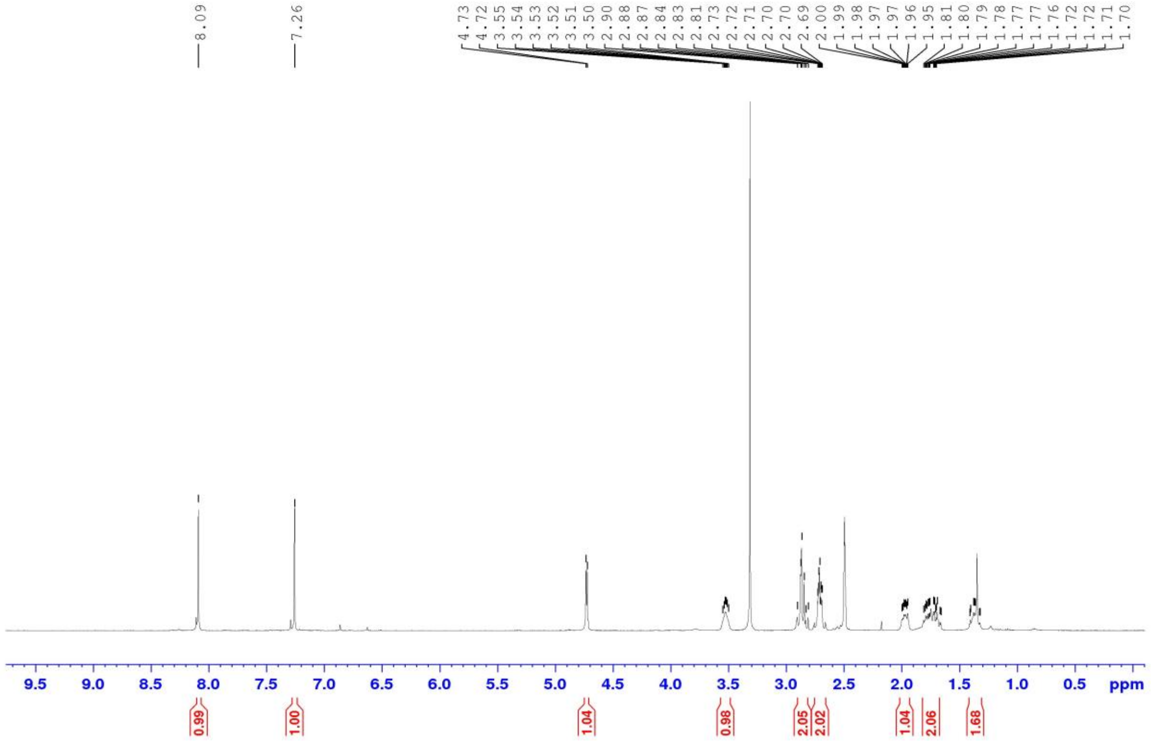

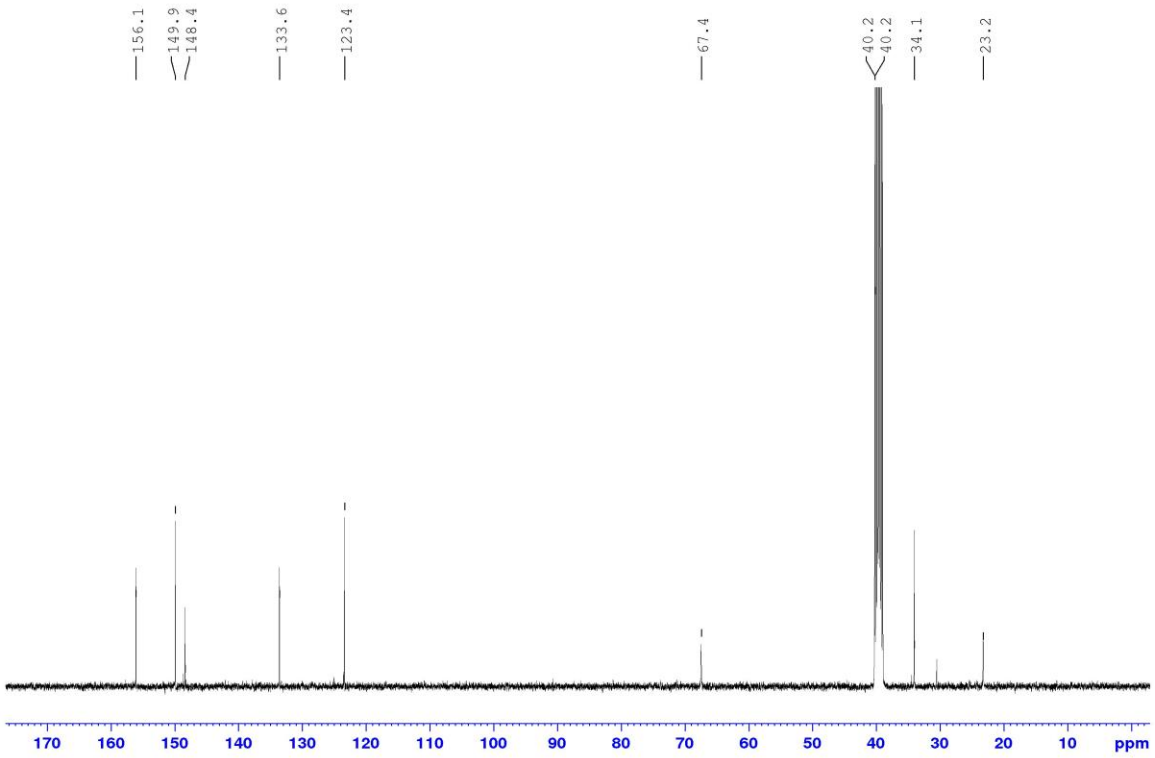

5-Chloro-1a,7,8,8a-tetrahydro-2*H*-oxireno[2',3':5,6]cyclohepta[1,2-*c*]pyridine, **50**

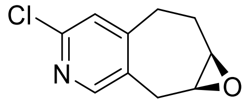

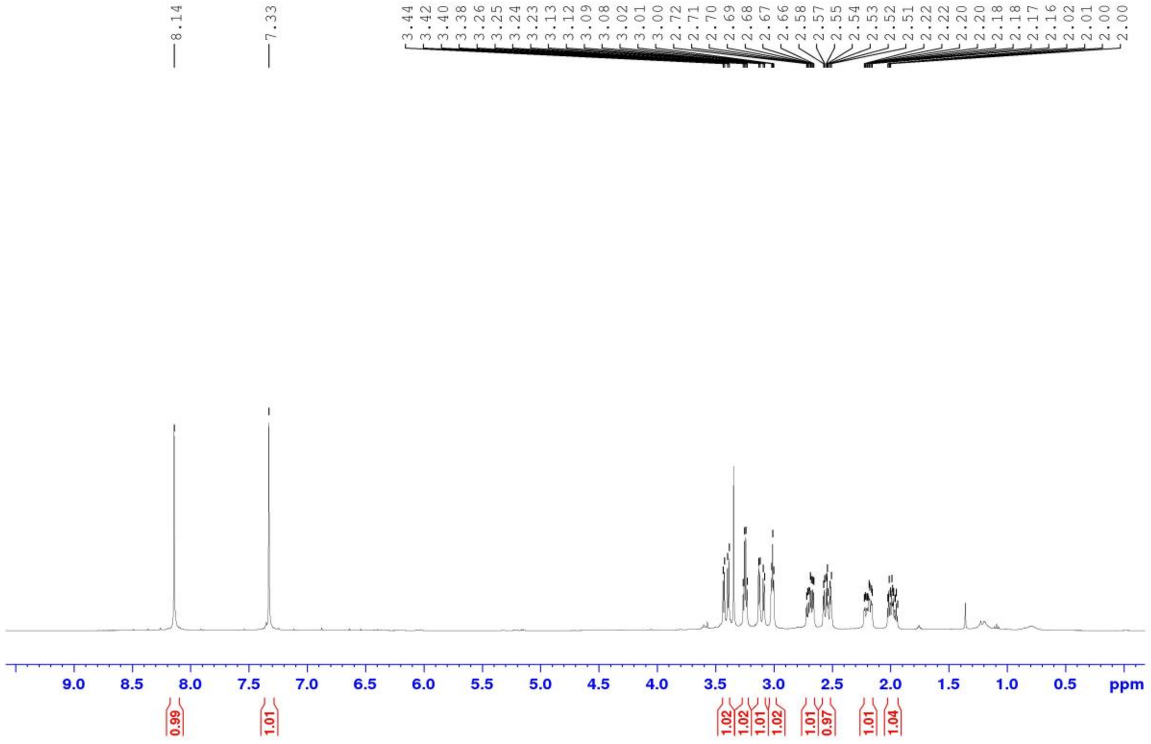

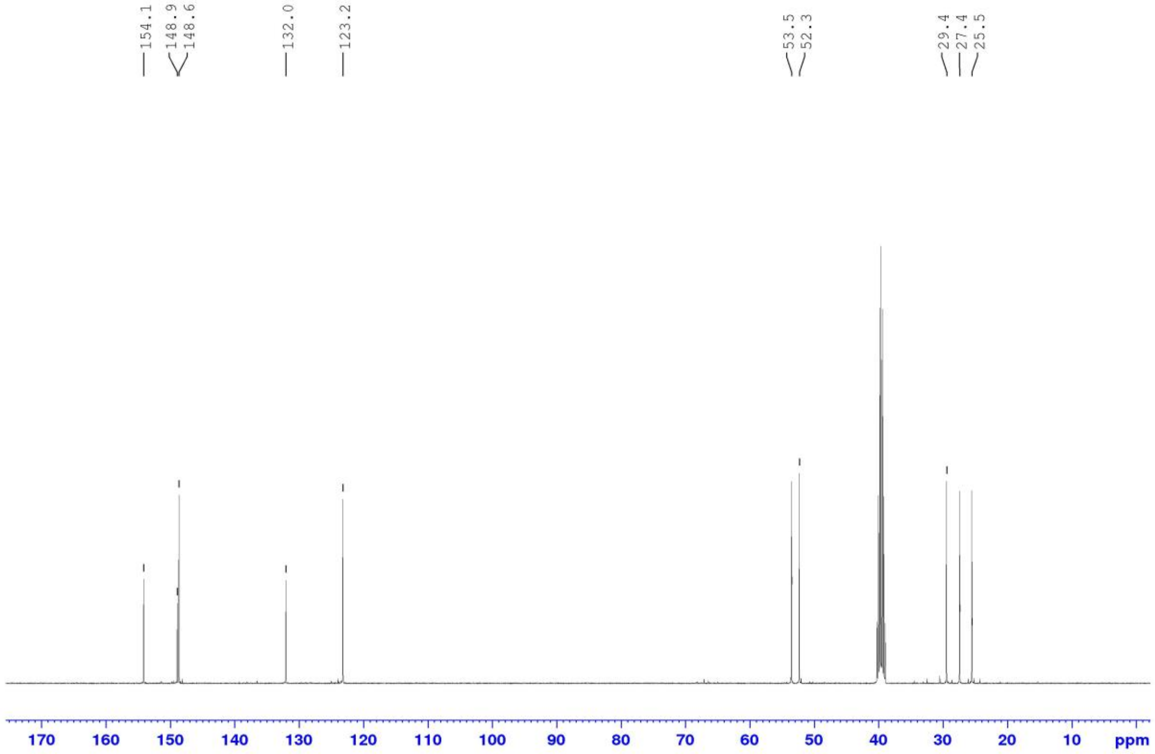

cis-3-chloro-6,7,8,9-tetrahydro-5*H*-cyclohepta[*c*]pyridine-7,8-diol, **51**

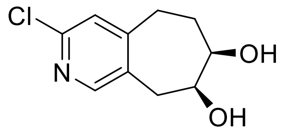

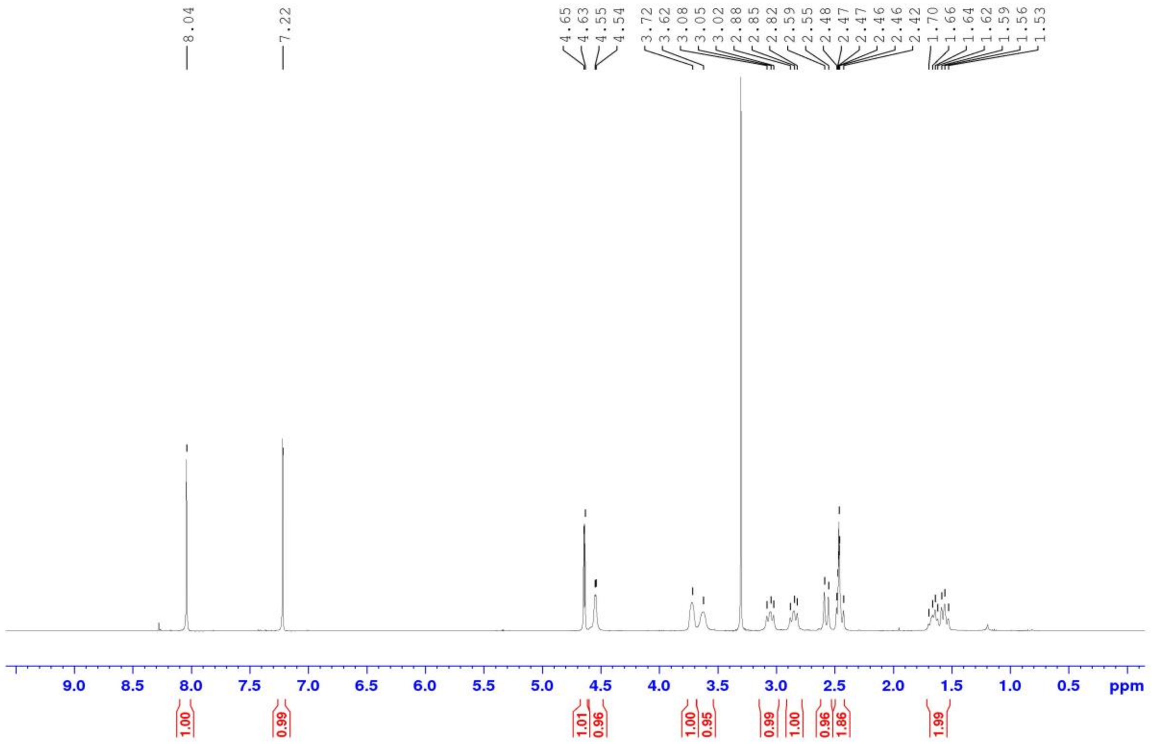

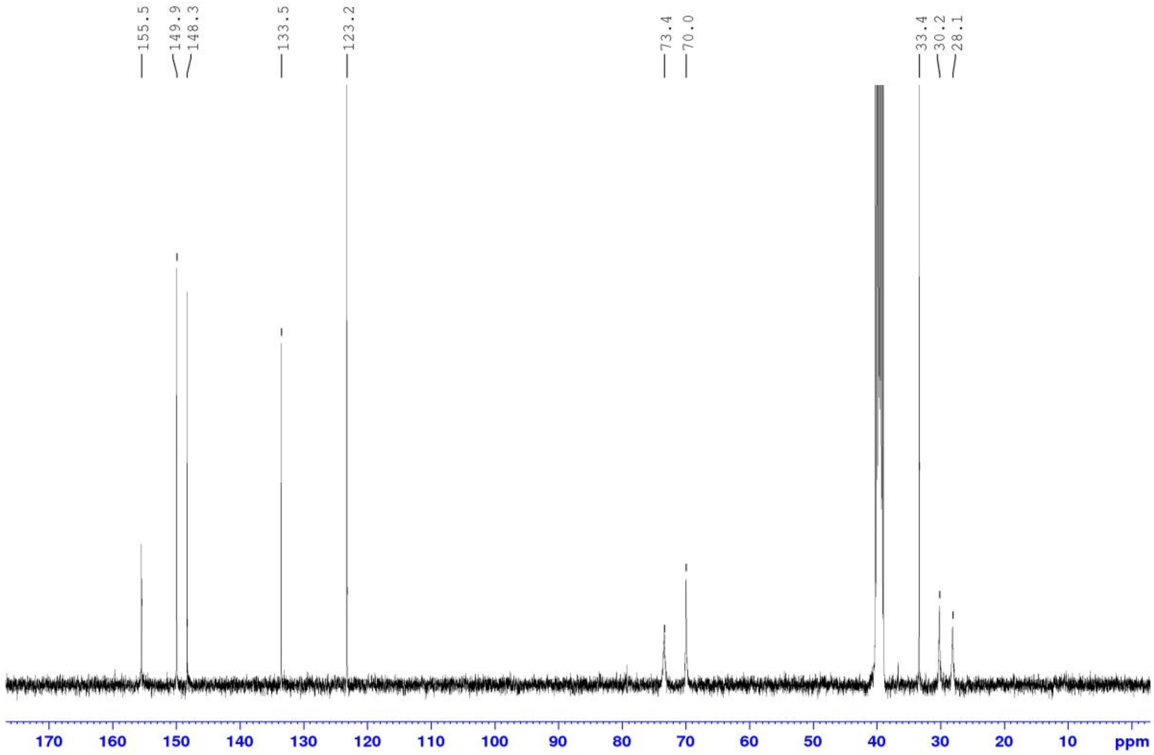

Supplement: Supplementary file 1 — Supplementary [file ANIE-55-12479-s001.pdf]
